# Redox-tunable isoindigos for electrochemically mediated carbon capture

**DOI:** 10.1038/s41467-024-45410-z

**Published:** 2024-02-08

**Authors:** Xing Li, Xunhua Zhao, Lingyu Zhang, Anmol Mathur, Yu Xu, Zhiwei Fang, Luo Gu, Yuanyue Liu, Yayuan Liu

**Affiliations:** 1 Department of Chemical and Biomolecular Engineering, Johns Hopkins University, Baltimore, MD 21218 USA; 2 Department of Mechanical Engineering & Texas Materials Institute, The University of Texas at Austin, Austin, TX 78712 USA; 3 Macao Institute of Materials Science and Engineering (MIMSE), Faculty of Innovation Engineering, Macau University of Science and Technology, Taipa, Macau 999078 China; 4 Department of Materials Science and Engineering, Johns Hopkins University, Baltimore, MD 21218 USA

**Keywords:** Carbon capture and storage, Electrochemistry, Electrocatalysis

## Abstract

Efficient CO_2_ separation technologies are essential for mitigating climate change. Compared to traditional thermochemical methods, electrochemically mediated carbon capture using redox-tunable sorbents emerges as a promising alternative due to its versatility and energy efficiency. However, the undesirable linear free-energy relationship between redox potential and CO_2_ binding affinity in existing chemistry makes it fundamentally challenging to optimise key sorbent properties independently via chemical modifications. Here, we demonstrate a design paradigm for electrochemically mediated carbon capture sorbents, which breaks the undesirable scaling relationship by leveraging intramolecular hydrogen bonding in isoindigo derivatives. The redox potentials of isoindigos can be anodically shifted by >350 mV to impart sorbents with high oxygen stability without compromising CO_2_ binding, culminating in a system with minimised parasitic reactions. With the synthetic space presented, our effort provides a generalisable strategy to finetune interactions between redox-active organic molecules and CO_2_, addressing a longstanding challenge in developing effective carbon capture methods driven by non-conventional stimuli.

## Abstract (web summary)

Electrochemically-mediated carbon capture has good energy efficiency and potential in carbon dioxide separation, but optimisation of these systems is challenging. Here, the authors report the use of isoindigo derivatives to give fine-tuned interactions with CO_2_ for carbon capture methods.

## Introduction

**Fig. 1:**
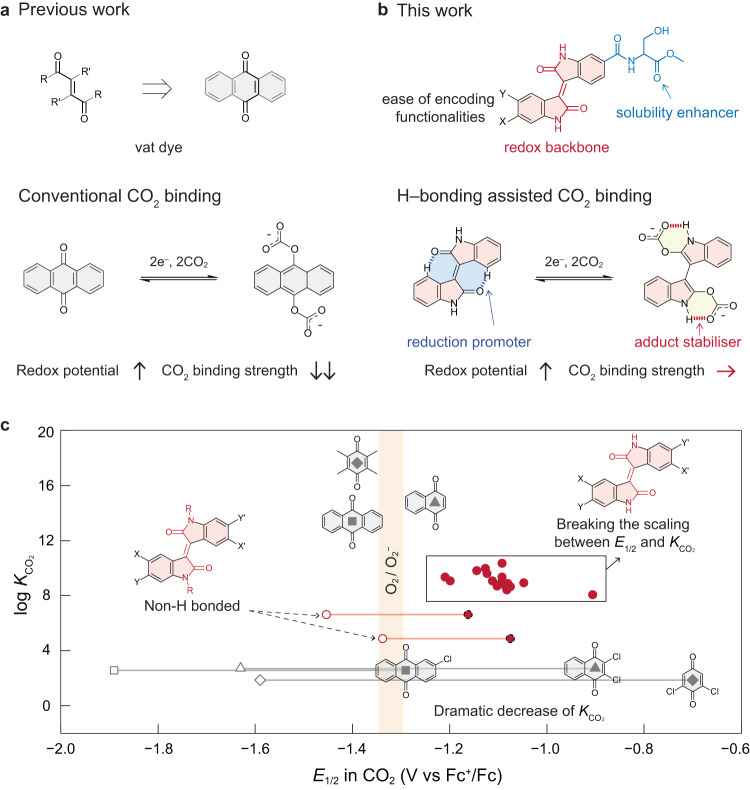
Rational design of bifunctional redox-tunable CO_2_ sorbents based on isoindigo and their derivatives. **a** A universal designing pattern for redox-tunable CO_2_ carriers containing *α,β*-unsaturated 1,4-diketone functionality. In previous designs, CO_2_ is only bonded to the reduced O centre via carbonate formation, where the binding affinity is sensitive to structural modification. **b** Bifunctional redox-tunable CO_2_ carrier design based on isoindigo. The secondary functionality of amide allows intramolecular hydrogen bonding, providing an extra handle to stablise the CO_2_ adduct, thereby enhancing the CO_2_ binding strength. **c** A summary of *E*_1/2_ of typical quinone-based sorbents and isoindigos under CO_2_, and their $\log {K}_{{{{{{{\rm{CO}}}}}}}_{2}}$ in DMF (filled and empty dots represent the first and second *E*_1/2_ under CO_2_, respectively circle, isoindigos; diamond, benzoquinone; triangle, naphthoquinone; square, anthraquinone).

Carbon capture from stationary emitters or directly from the ambient environment, followed by sequestration or utilisation, is critical to mitigating climate change^1–3^. However, the incumbent wet chemical scrubbing methods for carbon dioxide (CO_2_) separation are technically and economically challenged by various inherent limitations, including high energy consumption for sorbent regeneration, thermal degradation, complexity in heat integration when retrofitting existing infrastructures, process equipment corrosion, and fugitive emission of volatile toxic sorbents to the environment^4,5^. Alternatively, electrochemically mediated carbon capture (EMCC) has emerged as a promising technology^6–11^. In EMCC, reversible CO_2_ capture and release is modulated by switching electrochemical potentials. Therefore, they can be operated isothermally at ambient pressure, powered by renewable energy sources, and modularly designed to accommodate the multiscale nature of carbon capture needs. Among the EMCC mechanisms explored to date, one popular strategy is to use redox-active organic compounds as CO_2_ carriers (redox-tunable Lewis bases), with quinones being the most studied class of molecules^12–14^. Electro-reduction of these molecules generates nucleophiles that form adducts with electrophilic CO_2,_ which can be later oxidised to liberate pure CO_2_ while regenerating the sorbents (Fig. 1a). The past two decades have witnessed steady research progress in developing EMCC processes using redox-tunable sorbents^8,10,15–20^. Several bench-scale prototypes have been demonstrated for fixed-bed and flow-based CO_2_ separations attributed to materials and device-level engineering efforts^17–19^. In contrast, molecular-level design principles for precise sorbent property tuning remain largely unestablished beyond the simplistic method of structural substitution with electron-donating and withdrawing groups, despite their central role in EMCC.

The lack of reliable sorbent chemistry has intrinsically hindered existing EMCC processes. A practical EMCC system usually requires chemical modification of redox-tunable sorbents to improve key properties such as solubility, processibility, and stability against impurities. For example, anodically shifting the redox potential will enhance the robustness of activated sorbent against molecular oxygen (O_2_), a common gas stream impurity, for improved carbon capture efficiency. Nevertheless, the CO_2_ binding affinity of existing redox-tunable Lewis base sorbents, such as quinones, is highly susceptible to chemical modifications, which decreases dramatically as the redox potential shifts anodically^21–23^. The seminal work from W. L. Bell et al. introduced a method to calculate the CO_2_ binding constant (${K}_{{{{{{{\rm{CO}}}}}}}_{2}}$) of activated sorbent, which is widely adopted later to evaluate the CO_2_ binding strength^12^. As an example, anthraquinone exhibits a two-electron-transfer half-wave potential (*E*_1/2_) of −1.4 V vs. ferrocenium/ferrocene (Fc^+^/Fc) in *N*,*N*-dimethylformamide (DMF) under CO_2_ and a $\log {K}_{{{{{{{\rm{CO}}}}}}}_{2}}$ of ~13.4 (Fig. 1c). The installation of electron-withdrawing groups (EWGs), such as one chloro group at 2-position, can anodically shift *E*_1/2_ to −1.25 V vs. Fc^+^/Fc, yet the $\log {K}_{{{{{{{\rm{CO}}}}}}}_{2}}$ decreased substantially to 2.73 (Fig. 1c and Supplementary Table 1)^21^. A minimum $\log {K}_{{{{{{{\rm{CO}}}}}}}_{2}}$ of ~3.0 in DMF is required to attain a practical efficiency for point source carbon capture (10% CO_2_), and the $\log {K}_{{{{{{{\rm{CO}}}}}}}_{2}}$ must be >~5.5 for atmospheric CO_2_ concentration (400 ppm)^12^. Importantly, it is challenging to overcome the coupling between *E*_1/2_ and $\log {K}_{{{{{{{\rm{CO}}}}}}}_{2}}$ as it is dictated by the fundamental principle in chemistry that electron deficiency facilitates reduction but in return weakens nucleophilicity^24^. Therefore, to fulfil the practical requirements of EMCC, it is pivotal to develop new classes of redox-tunable sorbents that can break the linear free-energy relationship between redox potential and CO_2_ binding strength to enable aerobic stability and high CO_2_ capacity (Supplementary Fig. 1).

Here, through analysing the structures of existing EMCC sorbents, we observe that the most representative quinoid species share a common pattern of *α*,*β*-unsaturated 1,4-diketone (Fig. 1a). Inspired by this structural pattern, we present a class of redox-tunable CO_2_ carriers based on isoindigo compounds, which can successfully overcome the undesirable coupling between redox potential and CO_2_ binding strength (Fig. 1b, c). With a molecular library of 21 examples and a combined experimental and computational effort, we show that the *α*,*β*-unsaturated 1,4-diketone in isoindigos plays the role of redox backbone for CO_2_ binding and the amide groups act as extra docking sites for CO_2_ complexation via intramolecular hydrogen bonding. This unique bifunctional structural design allows a wide range of chemical modifications to independently optimise key sorbent properties without sacrificing their abilities for CO_2_ binding. The isoindigo family culminates in an EMCC system that can operate at mild potentials (around −1 V vs. Fc^+^/Fc) with $\log {K}_{{{{{{{\rm{CO}}}}}}}_{2}}$ maintained at ~9. The value is five orders of magnitude higher than tetrachloroquinone with alcohol additives^22^, which is the state-of-the-art sorbent chemistry with an attempt to break the linear free-energy relationship. Flow-based separation prototypes have also been demonstrated to evaluate the EMCC performance of the isoindigo sorbents, which can achieve CO_2_ capacity utilisation efficiencies up to ~80% and energy consumptions as low as 127.3 kJ mol^−1^ per CO_2_ capture/release cycle. With intrinsic O_2_ stability, high structural tunability, and synthetic feasibility, isoindigos are promising to serve as the next-generation EMCC sorbents. Moreover, this work demonstrates a generalisable strategy to overcome the intrinsic linear free-energy limits in redox-active organic species that can be broadly applied to EMCC and beyond.

## Results

### Redox-tunable CO_2_ absorption of isoindigo

**Fig. 2:**
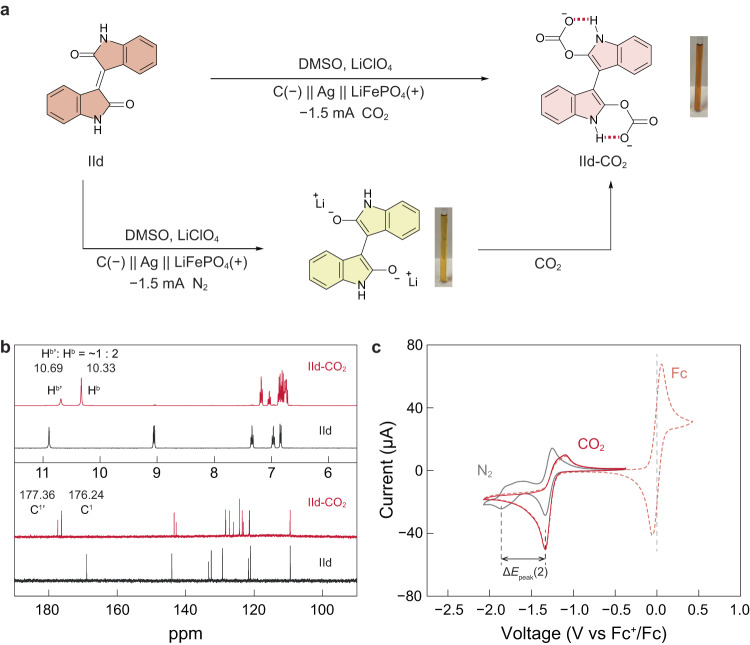
Validating the redox-tunable CO_2_ absorption of isoindigo. **a** Bulk electrolysis of isoindigo (IId) under N_2_ or CO_2_ atmosphere. The CO_2_ adduct can be obtained either by directly reducing IId (40 mM) in DMSO with 0.25 M LiClO_4_ under CO_2_ or by reducing IId under N_2_ followed by purging with CO_2_. **b** ^1^H NMR and ^13^C NMR spectra of the crude solution after bulk electrolysis under CO_2_. The minor isomer peaks are marked with an asterisk. **c** CV curves of IId (2.5 mM) in DMF using 0.1 M Nbu_4_PF_6_ as the supporting salt under N_2_ (grey) and CO_2_ (red) at a scan rate of −50 mV s^−1^ (5 mM ferrocene as the internal reference). ∆*E*_peak_(2) is labelled for the calculation of ${K}_{{{{{{{\rm{CO}}}}}}}_{2}}$.

Isoindigo and its derivatives have been extensively utilised as core building blocks in organic semiconductors^25–28^, but have rarely been explored as redox molecules for organic electrodes. We envisage isoindigos bearing *α*,*β*-unsaturated 1,4-diketone functionalities to be redox-active and can complex with CO_2_ at the oxygen centres in the reduced state. A series of electrochemical experiments was conducted to validate the redox-driven interaction between isoindigo (IId) and CO_2_. First, we performed bulk electrolysis of IId using 0.25 M lithium perchlorate (LiClO_4_) in dimethyl sulfoxide (DMSO) as the supporting electrolyte under N_2_ or CO_2_ atmosphere (Fig. 2a). Under N_2_, the dark dispersion of IId turned greenish at the beginning of the reduction and became a clear yellow solution when the reaction was completed. This suggests the formation of the bisindolidenolate intermediate, which is expected to absorb CO_2_ as a Lewis base. Interestingly, the yellow solution quickly turned red when purged with CO_2_, implying the formation of the IId-CO_2_ adduct. To our delight, ^1^H and ^13^C nuclear magnetic resonance (NMR) spectra of the crude solution confirm the full conversion of IId into IId-CO_2_ with negligible side products (Fig. 2b). Characteristic peaks for the carbonate carbon were observed at 176 ~ 177 pm in ^13^C NMR. Besides, the proton on the lactam N shifts upfield to 10.33 ppm in IId-CO_2_ compared to that of 10.89 ppm in IId, strongly evidencing the formation of intramolecular hydrogen bonding with complexed CO_2_ (highlighted in red in Fig. 2a). The NMR spectra exhibit two types of amide hydrogens and carbonate carbons, which is probably due to the rotational isomerisation of IId-CO_2_ (Supplementary Fig. 2). The presence of intramolecular hydrogen bonding in IId-CO_2_ was further verified by variable-temperature (VT) ^1^H NMR, 2D ^1^H-^13^C Heteronuclear Single Quantum Coherence (HSQC) NMR, and Fourier transform infra-red (FT-IR) experiments (Supplementary Figs. 3–5, see Supplementary Note 1 for detailed analysis). The full NMR peak assignment of IId and IId-CO_2_ is given in Supplementary Fig. 2.

Since the discovery of the EMCC mechanism using quinones in 1988^12,13^, to our best knowledge, the widely accepted electrochemically generated quinone-CO_2_ carbonate adduct is still a proposed structure and has not been confirmed in an EMCC process by non-ambiguous characterisations. This is probably due to the poor stability of the adducts and the transient bonding nature between the reduced sorbent and CO_2_. On the contrary, crude NMR spectra suggest bulk electro-reduction of IId under CO_2_ can yield the proposed adduct with full conversion (IId was fully consumed), indicating the high selectivity of this chemistry and the sufficient stability of the adduct, which we postulate to be the result of intramolecular hydrogen bonding. Noticeably, the IId-CO_2_ solution obtained from bulk electrolysis can be stably stored in ambient air with high water content. After 53 days, <9 mol% of IId-CO_2_ oxidised back to the neutral IId form (Supplementary Fig. 6).

To confirm the redox activity of IId, we measured its cyclic voltammetry (CV) using 0.1 M tetrabutylammonium hexafluorophosphate (NBu_4_PF_6_) in DMF as the supporting electrolyte (Fig. 2c). Under an inert N_2_ atmosphere, IId exhibits two major redox waves typical to stepwise two-electron transfer. Like quinoid species^13^, the two electron transfer steps correspond to the formation of anionic radicals (IId^⋅−^) and dianions (IId^2−^), respectively. The two reduction peaks under N_2_ emerge into one in the presence of CO_2_ with a nearly doubled peak current, indicating chemical interactions between reduced IId and CO_2_. This behaviour is analogous to the other redox-tunable CO_2_ sorbents reported previously based on oxygen or nitrogen binding centres, whose reaction with CO_2_ proceeds via an ECEC mechanism (E, electron transfer; C, chemical reaction)^12,13,20^. As a Lewis acid, CO_2_ can withdraw the electron density from IId^⋅−^ to promote the second electron transfer, giving rise to an anodically shifted second reduction wave. Besides, the oxidation peak also shifts anodically and becomes quasi-reversible, corroborating the formation of the IId-CO_2_ adduct, which requires more energy for CO_2_ desorption.

**Fig. 3:**
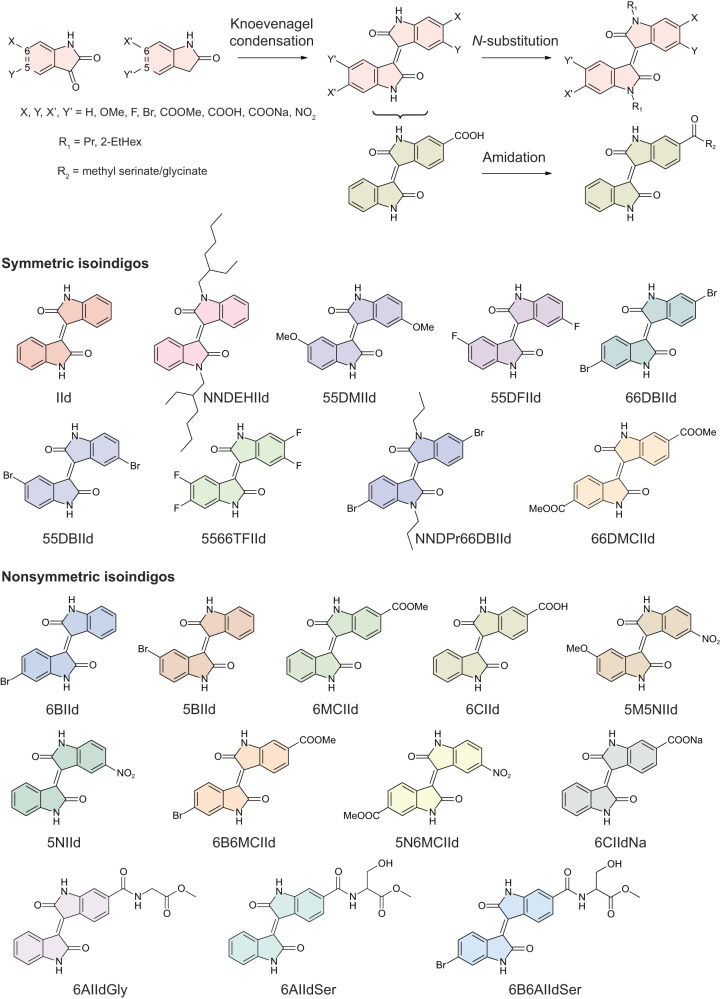
Modular synthesis of redox-tunable isoindigo sorbents for EMCC. Functional groups can be pre-installed onto the precursors of isoindigos and various isoindigos can be obtained through Knoevenagel condensation of the precursors. Amino acid ester can be further installed through amidation reaction with carboxylic acid modified isoindigos.

**Fig. 4:**
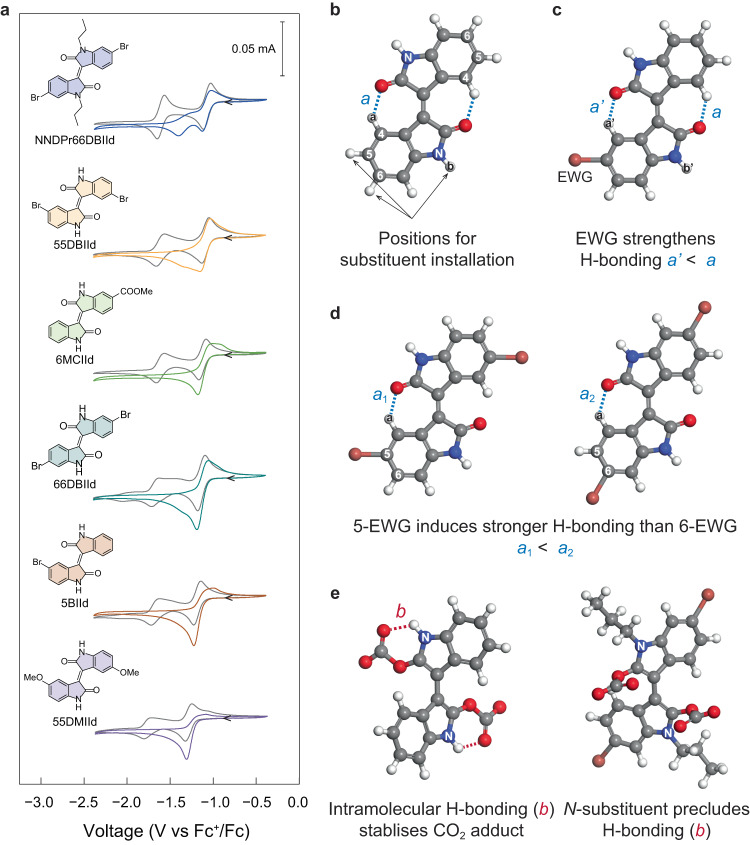
Structure-property relationships of redox-tunable isoindigo-based CO_2_ sorbents. **a** CV of various isoindigos using 2.5 mM compound in DMF with 0.1 M NBu_4_PF_6_ under N_2_ (grey) or CO_2_ (coloured). The CV curves were recorded at a scan rate of −50 mV s^−1^ at 298 K. **b** DFT-optimised structure of IId; positions for introducing substituent groups are labelled, which are at 5, 6, and lactam *N*-position; intramolecular hydrogen bonding (***a*** indicates bond length) in the neutral state is shown as blue dashed lines. **c** DFT-optimised structure of 5BIId suggests EWG can strengthen the hydrogen bonding (***a***). **d** DFT-optimised structures of 55BIId (left) and 66DBIId (right) showing that EWG substituent at 5-position is more effective in strengthening the hydrogen bonding (***a***). **e** DFT simulation showing that intramolecular hydrogen bonding (***b***) indicates bond length, red dashed lines) occurs in the CO_2_ adduct, stabilising the CO_2_ binding. Colour of atoms: grey: C; red: O; blue: N; brown: Br; white: H.

This preliminary finding encouraged us to expand the chemical scope of isoindigos and search for more qualified EMCC compounds. Isoindigos can be synthesised through the condensation reaction between 2-oxindoles and isatins. This allows the modular design of redox sorbents, where the two building units can be modified separately and integrated into isoindigos in the last step, thereby addressing the synthetic barriers when engineering EMCC sorbents (Supplementary Note 2). Here, we demonstrate the structural modification of isoindigo at 5, 6, and *N*-positions with 21 examples (Fig. 3) and examine in detail the interplay between substituent groups and hydrogen bonding on the redox and CO_2_ binding behaviours of isoindigos to establish the underlying structure-property relationships. The main conclusions are summarised in Fig. 4 and will be discussed in detail in the following sections.

### Tuning the redox potential of isoindigos

We first examine the redox behaviours of derivatised isoindigos under N_2_ via CV. Delightfully, all the isoindigos tested display redox couples typical to stepwise two-electron transfer, underscoring the good electrochemical reversibility of these molecules (Fig. 4a, Supplementary Figs. 7–9).

Among the 21 examples of isoindigos, only IId, 6BIId, and 66DBIId exhibit less well-defined shapes for the second redox wave. This is probably due to the rotational isomerisation of the one-electron reduced isoindigo radical anions. As shown in Supplementary Fig. 10, the C = C bond connecting the two oxindole rings becomes a single bond in the radical anion, allowing free rotation of the two rings. This creates rotational isomers and gives rise to shoulder peaks in the second redox process. Adding strong EWGs or substitution groups at the 5-position of isoindigo can create dipole-dipole repulsion or steric hindrance, inhibiting rotational isomerisation and resulting in a more reversible second redox process. Similarly, adding CO_2_ onto isoindigos also creates dipole and steric hindrance, impeding the rotational isomerisation and leading to more defined CV curves.

**Fig. 5:**
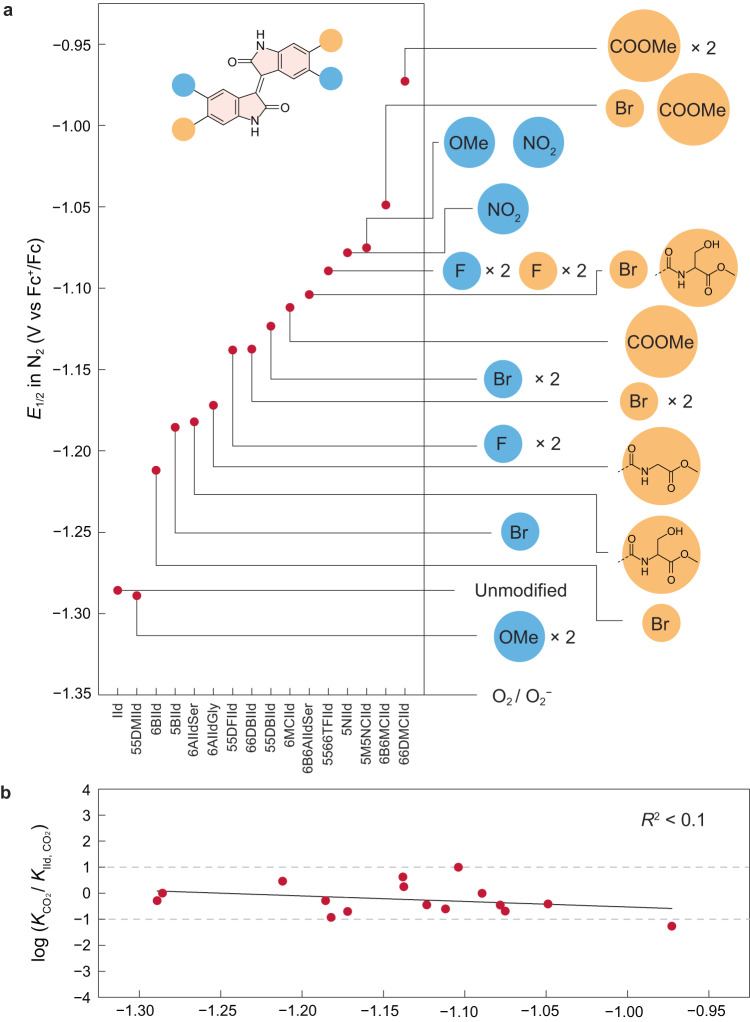
The substituent effect on the redox potentials and CO_2_ binding abilities of isoindigos. **a** The effect of substituent group on the first electron-transfer half-wave potentials under N_2_. **b** The plot between the first electron transfer half-wave potential under N_2_ and the change in CO_2_ binding constant relative to unmodified isoindigo. There is no linear correlation (*R*^2^ < 0.1) between the relative CO_2_ binding constants and the half-wave potentials.

Ideal EMCC sorbents shall have redox potentials more positive than the oxygen reduction potential (−1.35 V vs. Fc^+^/Fc in DMF, Supplementary Fig. 11) to minimise sorbent sensitivity towards O_2_. With stronger or increasing numbers of EWG introduced to the isoindigo rings, the redox potential exhibits an increasing anodic shift in the sequence of -F, -Br, -COOMe, -CONHR, and -NO_2_ substituent groups from mono- to tetra-substitution (Fig. 5a and Supplementary Table 2). Through a close examination of the structure-property relationship, we hypothesise that the anodic shift is attributed to not only the commonly expected electronic state tuning from the EWG substituents but also the intramolecular hydrogen bonding effect (***a*** shown in Fig. 4b and c). This is because hydrogen bonding can decrease the electron density of the redox-active oxygen centre to facilitate reduction. The downfield shift of proton at 4-position (H^a^) in isoindigos with EWG substituents suggests the formation of stronger hydrogen bonding (***a***) (Table 1 and Supplementary Table 2).

**Table 1:** The interplay between substituent groups and intramolecular hydrogen bondings in isoindigos and the corresponding impacts on redox potentials (V vs. Fc^+^/Fc) and CO_2_ binding


Isoindigos	^1^HNMR (H^a^) (ppm)^A^	^1^HNMR (H^b^) (ppm)^A^	Bond length of *a* (Å)^B^	Bond length of *b* (Å)^B^	Bond length of *c* (Å)^B^	*E*_1/2_(IId/IId^●−^) in N_2_ (V vs Fc^+^/Fc)	$\log {K}_{{{{{{{\rm{CO}}}}}}}_{2}}$
IId	9.06	10.89	1.963	1.954	1.461	−1.29	9.34
55DMIId	8.85	10.69	1.953	1.97	1.463	−1.29	9.05
5BIId	9.07	10.96	1.962	1.957	1.463	−1.19	9.05
5BIId (EWG)	9.31	11.05	1.936	1.943	1.467	−1.19	9.05
55DBIId	9.32	11.11	1.939	1.946	1.469	−1.09	9.33
6MCIId	9.07	10.94	1.960	1.954	1.954	−1.12	8.89
6MCIId (EWG)	9.15	11.08	1.956	1.944	1.476	−1.12	8.89
66DBIId	8.99	11.1	1.961	1.942	1.468	−1.14	9.59
NNDPr66DBIId	9.05	NA	1.948	NA	1.486	−1.07	4.87

^A 1^H NMR were recorded on the neutral molecules in DMSO-d_6_ using solvent residual peak as the internal reference for calibration. ^B^ Bond length was obtained from DFT-optimised structures. ^C^ Results from the non-symmetric oxindole ring with the EWG-substituent.

To verify the above hypothesis, we show that EWG substituent at 5-position is more effective in facilitating electro-reduction than that at 6-position. This is because the former is in the ortho-position of H^a^ and more effective in pulling away the electron density, thereby inducing a stronger hydrogen bonding (Fig. 4d). For instance, 55DBIId and 66DBIId exhibit very close ^1^H NMR peaks for H^b^ (11.11 and 11.10 ppm, Supplementary Fig. 12), suggesting similar degrees of electron deficiency in these two molecules. In contrast, the chemical shift of H^a^ is 9.32 and 8.99 ppm for 55DBIId and 66DBIId, respectively, clearly indicating a stronger hydrogen bonding (***a***) in the 5-substituted species. Therefore, *E*_1/2_(IId/IId^⋅−^) of 55DBIId (−1.09 V vs. Fc^+^/Fc) is more positive compared to 66DBIId (−1.12 V vs. Fc^+^/Fc). This trend is consistent for all examples in our isoindigo family (Supplementary Table 2, e.g., 5BIId vs. 6BIId and 6MCIId vs. 5NIId vs. 5N6MCIId) and is further confirmed with DFT calculations (details *vide post*).

Counterintuitively, adding electron-donating groups (EDGs) such as methoxy shows a negligible influence on *E*_1/2_(IId/IId^⋅−^), as evidenced by IId vs. 55DMIId (both show *E*_1/2_ of −1.29 V vs. Fc^+^/Fc, Table 1), and 5NIId vs. 5M5NIId (both show *E*_1/2_ of −1.08 V vs. Fc^+^/Fc, Supplementary Table 2). This is likely due to the charge-transfer effect that lowers the energy level of the molecule, offsetting the electronic effect from EDGs. Specifically, isoindigo species are electron acceptors (n-type organic semiconductors)^28^, where charge transfer can be induced between the electron-deficient isoindigo rings and the electron-rich methoxy group. UV-vis absorption spectra suggest an optical bandgap of 1.90 ~ 1.98 eV for most isoindigos with or without chemical modification (Supplementary Table 2 and Supplementary Figs. 13–15). However, the bandgaps drop to 1.78 and 1.71 eV for methoxy substituted 55DMIId and 5M5NIId, manifesting charge transfer in these two compounds.

### Breaking the scaling relationship between redox potential and CO_2_ affinity

After understanding the effect of molecular structures on the redox potentials of isoindigos, we further study their CO_2_ binding behaviours. All isoindigos in this work exhibit anodically shifted potential for the second electron transfer process under CO_2_, confirming their ability to form CO_2_ adducts upon electro-reduction. Importantly, through the combined effect of EWGs substitution and hydrogen bonding (***a***) discussed above, all isoindigos with unsubstituted H^b^ display *E*_1/2_ values anodic to oxygen reduction under CO_2_, implying favourable stability of isoindigo sorbents against O_2_.

In all previous reports on redox-tunable CO_2_ sorbents, anodically shifted redox potential always comes with significantly diminished CO_2_ binding affinity (Supplementary Table 1). However, another key finding of this work is that hydrogen atom (H^b^) on the lactam-N of isoindigos can induce intermolecular hydrogen bonding with the complexed CO_2_ molecule to thermodynamically stabilise the CO_2_ adduct (Fig. 4e). As a result, regardless of *E*_1/2_, the $\log {K}_{{{{{{{\rm{CO}}}}}}}_{2}}$ values of isoindigos with unsubstituted H^b^ were found to be relatively constant (Table 1 and Supplementary Table 2, see Supplementary Note 3 for details on ${K}_{{{{{{{\rm{CO}}}}}}}_{2}}$ calculation), suggesting the high tolerance of such redox carriers to a wide range of chemical modifications.

To further demonstrate the role of hydrogen bonding (***b***) on CO_2_ adduct stabilisation, we synthesised two examples with *N*-substitutions to eliminate this hydrogen bonding (NNDPr66DBIId and NNDEHIId). Compared with 6,6’-dibromo substituted 66DBIId ($\log {K}_{{{{{{{\rm{CO}}}}}}}_{2}}$ = 9.59), the CO_2_ binding constant of *N*-alkylated NNDPr66DBIId ($\log {K}_{{{{{{{\rm{CO}}}}}}}_{2}}$ = 4.48) shows a dramatic decrease of five orders of magnitude (Table 1). A similar phenomenon was observed between NNDEHIId ($\log {K}_{{{{{{{\rm{CO}}}}}}}_{2}}$ = 6.62, Supplementary Table 2) and IId ($\log {K}_{{{{{{{\rm{CO}}}}}}}_{2}}$ = 9.34). Besides, we designed an isoindigo bearing a carboxylic acid group (6CIId), which serves as a free proton donor to disrupt the intramolecular hydrogen bonding (***b***). As a result, the CO_2_ binding ability of reduced 6CIId almost diminished with a low $\log {K}_{{{{{{{\rm{CO}}}}}}}_{2}}$ of 2.87 (Supplementary Table 2).

To visualise our success in breaking the scaling relationship between redox potential and CO_2_ binding affinity, we plot the change in ${K}_{{{{{{{\rm{CO}}}}}}}_{2}}$ relative to unmodified IId against *E*_1/2_ of the first electron transfer under N_2_ (Fig. 5b). Upon installing EWGs, *E*_1/2_ can be effectively shifted from −1.29 V to −0.97 V vs. Fc^+^/Fc. Nevertheless, the ${K}_{{{{{{{\rm{CO}}}}}}}_{2}}$ values exhibit minimal changes within only one order of magnitude, as long as the lactam-N is unsubstituted to facilitate intramolecular hydrogen bonding. Furthermore, linear fitting shows a negligible correlation (*R*^2^ < 0.1), underlining that our molecular design strategy can indeed break the scaling relationship between redox potential and CO_2_ binding affinity.

We further tested the bimolecular rate constant (*k*_*bimolecular*_) for the reaction between isoindigo radical anion and CO_2_ (Supplementary Figs. 16–19, Supplementary Table 3, see Supplementary Note 3 for details in measurement). With intramolecular hydrogen bonding (***b***) in the CO_2_ adduct, 6MCIId, 6AIIdSer, and 66DBIId display a similar rate constant of 22.5–18.6 M^−1^ s^−1^. However, the *N*,*N*-disubstituted NNDPr66DBIId exhibits a decreased rate constant of 3.3 M^−1^ s^−1^, manifesting that intramolecular hydrogen bonding (***b***) is also conducive to CO_2_ complexation kinetics.

At a CO_2_ concentration of 20% or 10%, the CV curves of isoindigos usually exhibit a positively shifted second reduction peak compared to that under N_2_; however, most do not completely emerge into the first (Supplementary Fig. 7 and 8). The phenomenon is attributed to the kinetic competition between the chemical transformation of IId^⋅−^ into [IId-CO_2_]^⋅−^ (*r* = *k*_*bimolecular*_[CO_2_][IId^⋅−^]^29^, where [CO_2_] and [IId^⋅−^] are the concentrations of CO_2_ and IId^⋅−^) and the electrochemical reduction of [IId-CO_2_]^⋅−^. Higher CO_2_ concentrations lead to higher *r*, which facilitates the formation of [IId-CO_2_]^⋅−^ and ultimately leads to a single, merged reduction peak. Correspondingly, we tested the CV of 6MCIId at different scan rates and indeed observed the gradual merging of the two reduction peaks with slower scans (Supplementary Fig. 20). Therefore, the separation between the two cathodic peaks under low CO_2_ concentrations can serve as a qualitative indicator for CO_2_ complexation kinetics.

As a final note, CO_2_ complexation can be frustrated when introducing highly strong EWGs or strong hydrogen bonding acceptors to isoindigo (5N6MCIId and 6CIIdNa). Detailed analysis is included in Supplementary Note 4 and Supplementary Fig. 21.

### Detailed investigation on the role of hydrogen bonding

Distinct from previously reported redox-tunable CO_2_ carriers, isoindigos possess intramolecular hydrogen bondings in both inactivated (neutral) and activated (reduced) forms, accounting for their unique EMCC properties. Therefore, we selected the seven most representative structures from our library and carefully compared various parameters such as NMR spectra, density functional theory (DFT)-optimised bond lengths, redox potentials, and CO_2_ binding constants to investigate the interplay between substituent groups and hydrogen bonding on the thermodynamic properties of isoindigos. Key data are summarised in Table 1 (also see Supplementary Figs. 22 and 23 for DFT-optimised structures). DFT calculation shows that the theoretical first electron transfer potential shifts anodically in the order of IId, 5BIId, 66DBIId, and 55DBIId, consistent with experimental observation (Supplementary Table 4). Besides, DFT-calculated CO_2_ binding constants ($\log {K}_{{{{{{{\rm{CO}}}}}}}_{2}}$) of the isoindigos agree well with the trend of our experimental results (Supplementary Table 5). DFT-optimised structures further confirm the formation of intramolecular hydrogen bonding (***a***) and (***b***).

The electron density of H^a^ at 4-position and H^b^ at *N*-position can be regarded as indicators of the bond strength of ***a*** and ***b***, respectively. Specifically, ^1^H NMR spectra reveal a chemical shift of 9.06 ppm for H^a^ and 10.89 ppm for H^b^ in unmodified IId, corresponding to a DFT-optimised bond length of 1.963 Å for ***a*** and 1.954 Å for ***b***. Introducing electron-withdrawing bromo groups at 5-position (55DBIId) downfield shifts H^a^ to 9.32 ppm and H^b^ to 11.11 ppm, resulting in an enhanced hydrogen bonding of 1.939 Å for ***a*** and 1.946 Å for ***b***, respectively. Interestingly, the electronics and hydrogen bonding of each oxindole ring can be independently tuned in nonsymmetric isoindigos. Using the nonsymmetric 5BIId as an example, the chemical shifts of H^a^ and H^b^ are 9.07 and 10.96 ppm at the non-substituted side, corresponding to a DFT-optimised bond length of 1.962 Å for ***a*** and 1.957 Å for ***b***, which is very close to IId. In contrast, H^a^ and H^b^ shift downfield to 9.31 and 11.05 ppm at the bromo-substituted side, corresponding to an enhanced hydrogen bonding of 1.936 Å for ***a*** and 1.943 Å for ***b***.

Moreover, introducing the same EWG at different positions modulates the strength of hydrogen bonding (***a***) differently. For example, with the same dibromo-substitution, the chemical shift of H^a^ in 66DBIId (8.99 ppm) is upfield to that of 55DBIId (9.32 ppm). This results in a weakened hydrogen bonding (***a***) of 1.961 Å in 66DBIId than that of 1.939 Å in 55DBIId, explaining the more negative reduction potential of 66DBIId (−1.14 V vs. Fc^+^/Fc) than 55DBIId (−1.09 V vs. Fc^+^/Fc). The above results strongly suggest that, in addition to the electronic effect of substituent groups, intramolecular hydrogen bonding (***a***) is also vital in facilitating the reduction of isoindigo.

Although decreasing the electron density of isoindigos by introducing EWGs at 5,6-positions can effectively facilitate their reduction, counterintuitively, the reduced isoindigos exhibit negligible decay in CO_2_ affinities, underscoring the importance of intramolecular hydrogen bonding (***b***). Using DFT calculation, we found that the nucleophilicity of the oxygen centre indeed weakens when EWG is introduced, as suggested by the increased length of the carbonate C–O bond (***c***). For instance, the bond length of ***c*** on the oxindole ring with –COOMe group is increased by 1.2 pm compared to that on the non-substituted ring in 6MCIId. However, as mentioned above, hydrogen bonding (***b***) strengthens with stronger or increasing number of EWG substituents to keep the bond length of ***c*** nearly constant. Moreover, the DFT-optimised structure suggests that hydrogen bonding (***b***) is precluded in NNDPr66DBIId, and the carbonate bends out-of-plane to the reduced isoindigo rings due to steric repulsion. Thus, the bond length of ***c*** in NNDPr66DBIId is increased by 1.8 pm compared to 66DBIId, giving rise to a significant drop in ${K}_{{{{{{{\rm{CO}}}}}}}_{2}}$ by almost five orders of magnitude.

The collective information above confirms our key conclusions. First, hydrogen bonding (***a***) can reduce the electron density at the redox centre and facilitate reduction. Second, hydrogen bonding (***a***) can be tuned by substituent groups, where EWG at 5-position is more effective than 6-position to shorten the bond length of ***a*** and hence facilitate reduction. Third, hydrogen bonding (***b***) stabilises the complexed CO_2_ when EWGs are introduced.

It is important to note that the effect of intramolecular hydrogen bonding on CO_2_ complexation has been briefly studied in prior works using quinones. However, it was observed that hydrogen bonding occupies the CO_2_ binding sites of quinones and diminishes the ability for CO_2_ capture^14,30^. Therefore, our work presents the first demonstration that intramolecular hydrogen bonding can facilitate CO_2_ adduct formation and break the intrinsic linear free-energy relationship of EMCC chemistries. This is attributed to the unique chemical structure of isoindigo that allows free rotation of the oxindole rings in the reduced state as supported by DFT simulation, breaking the intramolecular hydrogen bonding (***a***) to create space for CO_2_ complexation, which is further enhanced by the intramolecular hydrogen bonding (***b***) through the amide functionality.

### Finetuning the properties of isoindigos

Breaking the correlation between chemical modification and CO_2_ binding affinity greatly enhances the degree of freedom in finetuning sorbent properties. For instance, by installing EWGs such as methyl carboxylate, *E*_1/2_ of 66DMCIId under CO_2_ can be positively shifted by 300 mV compared to that of unmodified IId to impart O_2_ stability, while the $\log {K}_{{{{{{{\rm{CO}}}}}}}_{2}}$ only drops slightly (from 9.34 to 8.07). Besides, 66DMCIId is almost insoluble in organic solvents such as DMF (solubility < 2.5 mM), suggesting its potential as absorbent electrodes in fixed-bed EMCC devices.

Unmodified IId has a moderate solubility in DMF ( ~ 230 mM), which needs to be improved for practical use in flow-based EMCC systems^7^. To facilitate chemical functionalisation, we introduced a carboxyl group to the 6-position of isoindigos, which can be easily connected with amino acids through amidation reactions. As a proof of concept, we utilised glycine methyl ester and serine methyl ester as solubility enhancers and three nonsymmetric isoindigos were prepared (6AIIdGly, 6AIIdSer, and 6B6AIIdSer). 6B6AIIdSer features halogen substitution on one oxindole ring to tune redox potentials and amino acid ester functionalisation on the other oxindole ring to enhance solubility. To our delight, the solubilities of 6AIIdGly, 6AIIdSer, and 6B6AIIdSer increase to 606, 830, and 568 mM in DMF, respectively (Supplementary Table 6), likely due to the enhanced molecular interaction between the polar functional groups (amide and carboxylate) and DMF solvent. This is supported by the fact that 6AIIdSer is more soluble than 6AIIdGly due to the additional hydroxyl group from the serine moiety.

Conventional amine scrubbing sorbents have raised environmental concerns due to their biotoxicity^31,32^. Here, we show that introducing amino ester functionalities into isoindigos substantially improves their biocompatibility with mammalian cells. Unmodified IId shows a LC_50_ (lethal concentration that causes 50% cell death) of 11.2, 50.4, and 15.8 μg ml^−1^ for NIH3T3/GFP mouse fibroblasts, U2OS.EGFP human osteosarcoma cells, and MCF10A human breast epithelial cells, respectively, after 48 h cell culture (Supplementary Fig. 24 and 25). In comparison, the serinate-modified counterpart does not display clear toxicity under concentrations up to 100 μg ml^−1^ for NIH3T3/GFP and U2OS.EGFP, and has a significantly improved LC_50_ of 89.4 μg ml^−1^ for MCF10A.

### Effects of electrolytes, oxygen, and water

Before evaluating the CO_2_ capture performance of isoindigos in EMCC devices, we assessed the influence of electrolytes and common gas stream impurities such as water and O_2_ on their CO_2_ binding properties. Using 55DBIId as an example, the electrochemical behaviours remained almost unaffected up to a high O_2_ content (16% CO_2_ and 20% O_2_, Supplementary Fig. 26). Under N_2_, the CV curves of 55DBIId remain reversible even at a high water content of 10 vol% (Supplementary Fig. 27). The declining peak current is caused by the decreasing isoindigo solubility with increasing water content. However, the expected CO_2_ release at approximately −1 V vs. Fc^+^/Fc gets suppressed in the presence of 10 vol% water, possibly due to the involvement of pH-swing process under high water content that requires higher energy input for CO_2_ release.

We also studied the influence of supporting salt on the redox and CO_2_ binding behaviours of isoindigos (Supplementary Fig. 28). The most prominent effect comes from the choice of cation, where reducing the size of cation leads to anodically shifted reduction potential, as is explained by the electrostatic interaction between cation and reduced isoindigo. Smaller alkaline cations exhibit stronger Lewis acidity, allowing tighter binding with reduced isoindigo to facilitate electro-reduction. Therefore, a more acidic supporting salt cation can further enhance the robustness of isoindigo against O_2_. Nevertheless, it may also slow down the CO_2_ complexation kinetics due to the competition between cation and CO_2_ for binding with reduced isoindigo.

### Evaluating the isoindigo sorbents in flow-based EMCC prototypes

**Fig. 6:**
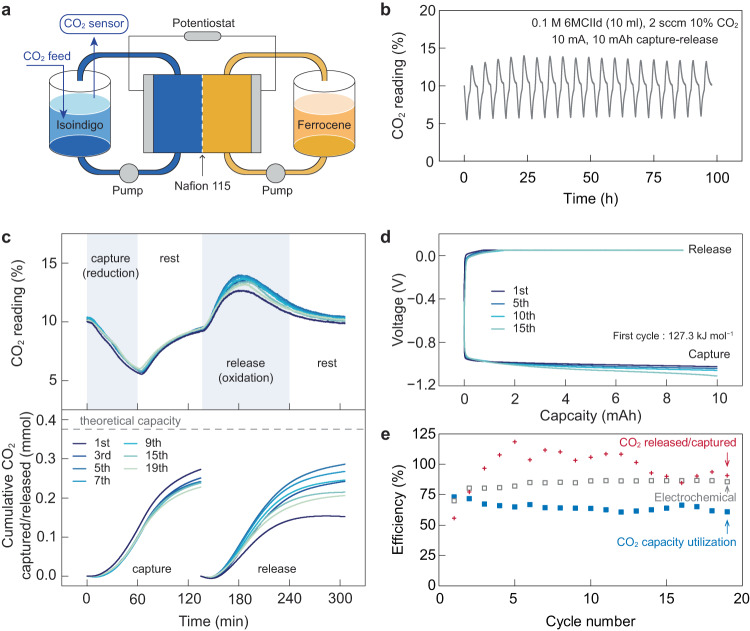
Evaluating the performance of 6MCIId in the flow-based EMCC prototype. **a** Schematic of the flow-based EMCC prototype. **b** CO_2_ reading at the exit of the sorbent tank over 19 repeating capture/release cycles for ~100 h of operation. **c**, The CO_2_ reading of selected capture/release cycles overlaid, with the cumulative amount of CO_2_ captured/released in each cycle relative to the theoretical capacity. Lighter colours represent later cycles. The shaded regions indicate the capture, rest, release, and rest steps. For CO_2_ capture, 6MCIId was reduced at 10 mA for 60 min followed by a 75 min rest. For CO_2_ release, the adducts were oxidised at 10 mA to 0.05 mV followed by a ~ 120 min constant voltage hold, and finally rested for another 75 min. **d** Selected voltage-capacity curves for the 1^st^, 5^th^, 10^th^, and 15^th^ capture/release cycle. **e** The CO_2_ capacity utilisation efficiency (blue squares), release/capture efficiency (red crosses), and electrochemical efficiency (empty grey squares) of the system. The liquid sorbent was composed of 10 ml 0.1 M 6MCIId in DMF with 0.25 M NaClO_4_ as the supporting salt. The sorbent tank was filled with plastic beads and purged with 10% CO_2_ at a flow rate of 2 standard cubic centimetres per minute (sccm). On the opposite side, a Fc tank was used to balance the charge, which was filled with 20 ml 0.1 M Fc in DMF with 0.25 M NaClO_4_ as the supporting salt, 4 mM ferrocenium tetrafluoroboronate (FcBF_4_) to facilitate Fc oxidation, and 10 mM 6MCIId to mitigate sorbent crossover.

Based on the CV peak potentials for CO_2_ capture and release, we estimated the theoretical minimum energy requirement for CO_2_ separation using isoindigo sorbents, which ranges from 9.8 to 27.6 kJ mol^−1^ CO_2_ (Supplementary Fig. 29). It is noteworthy that these molecules show very close onset potentials for CO_2_ capture and release such that the theoretical energetics calculated from CV peak potentials can be overestimated compared to previous calculations using onset values^17^. As a proof of concept, we evaluated their intrinsic capability for reversible CO_2_ capture and release in a flow-based EMCC prototype reported by us previously (Fig. 6a)^17^. Detailed testing conditions are provided in the Supplementary Information.

Figure 6b shows the cyclic capture-release performance of 6MCIId using 10% CO_2_ (balanced by N_2_) as the gas feed. The CO_2_ reading curves of each cycle are overlaid in Fig. 6c, where the cumulative CO_2_ captured/released is obtained by integrating these curves. For each cycle, 6MCIId was reduced at 10 mA for 60 min, and the decrease in CO_2_ concentration at the gas outlet confirmed carbon capture. The current was then stopped for 75 min, allowing the CO_2_ reading to gradually return to the baseline as the reduced isoindigo fully reacted with CO_2_. The CO_2_ adduct was subsequently oxidised following a constant current-constant voltage (CC-CV) protocol, and the continuous increase in CO_2_ concentration above 10% indicated CO_2_ desorption. Afterward, the current was set to zero again to ensure the complete release of the oversaturated CO_2_ from the sorbent electrolyte. The oxidation-reduction profiles of the flow system are shown in Fig. 6d. By integrating the voltage-capacity curves, the electrical energy consumption under 10% CO_2_ is estimated as 127.3 kJ mol^−1^ CO_2_ in the first cycle and 142.5 ± 8.2 kJ mol^−1^ CO_2_ over the first 16 cycles (Supplementary Fig. 30), which is comparable to other carbon capture technologies^18–20,33–38^.

Figure 6e summarises the three key metrics commonly used for evaluating EMCC performance. CO_2_ capacity utilisation, defined as the amount of CO_2_ captured relative to the theoretical value (one CO_2_ per electron), shows an average of 65% over 19 cycles, which is competitive against the state-of-the-art quinone-based sorbent reported recently^19^. The release/capture efficiency, defined as the ratio of the total amount of CO_2_ released and captured in each cycle, is averaged to be 97%, indicating the good reversibility of the sorbent. Considering a relatively constant electrochemical (Coulombic) efficiency of the EMCC prototype at ~84%, the major loss should be attributed to two factors: (1) our CC-CV protocol where the CO_2_ adduct was not fully oxidised; (2) the crossover of sorbents and counter electrolytes caused by membrane swelling, which limits all current nonaqueous redox-flow electrochemical systems. 6MCIId was also evaluated at a higher percentage of CO_2_ removal (Supplementary Fig. 31).

In addition to 6MCIId, we evaluated the performance of other isoindigo sorbents such as 55DBIId (Supplementary Fig. 32), 66DBIId (Supplementary Fig. 33), and 6B6AIIdSer (Supplementary Fig. 34). 55DBIId achieved an average CO_2_ utilisation efficiency of up to ~80% and an average release/capture efficiency of ~80%. Using 66DBIId, we studied ^1^H NMR of the crude sorbent electrolyte after 11 capture/release cycles over 50+ hours (Supplementary Fig. 35). The spectrum suggests the high stability of 66DBIId after cycling and also the severe crossover issue of the ferrocene counter electrolyte (three times the concentration of 66DBIId in the sorbent tank), which explains the decay in electrochemical capacity of current EMCC prototypes. Besides, we can recover 66DBIId with 87% yield from the sorbent electrolyte after cycling, corroborating the robustness of the sorbent. In addition, we found ~24% of 66DBIId isomerised into *cis*−66DBIId, supporting our hypothesis on the rotational isomerisation of reduced 66DBIId discussed earlier (Supplementary Fig. 10). Nevertheless, the reduction of both 66DBIId and its *cis*-isomer yield the same activated CO_2_ sorbent, which we believe does not affect the long-term stability of the EMCC prototype.

To our delight, 6B6AIIdSer exhibits a much lower oxidation potential for CO_2_ release, likely due to its higher solubility. Moreover, 6B6AIIdSer shows excellent cycling stability with negligible voltage decay over >40 cycles and ~200 h of operation with a capacity degradation rate of 2% (Supplementary Note 5).

We further tested the EMCC performance of 6MCIId using simulated flue gas (10% CO_2_ + 3% O_2_ balanced in N_2_) (Supplementary Fig. 36). The cell can run stably over ~90 h with reduction voltage maintained above −1.3 V, which minimised the parasitic oxygen reduction reaction. A CO_2_ capacity utilisation efficiency of ~50% was achieved with a near unity CO_2_ release/capture efficiency.

Finally, we evaluated the CO_2_ capture capability of 6MCIId under low CO_2_ concentration and its CO_2_ release capability under pure CO_2_. Using 1% CO_2_ with 0.3% O_2_ as the feed, we observe an early-stage energy consumption of 224.2 kJ mol^−1^ CO_2_ and a single pass CO_2_ removal of >90% (Supplementary Fig. 37). This suggests that our intramolecular hydrogen bonding strategy is effective in improving CO_2_ affinity for low-concentration CO_2_ capture. In another experiment, the CO_2_ capture-release behaviour under 100% CO_2_ headspace was quantified using mass flow metre (Supplementary Fig. 38). Under conditions similar to low-concentration CO_2_ capture, we show an early-stage energy consumption of 143.7 kJ mol^−1^ CO_2_ captured and 13.6 kJ mol^−1^ CO_2_ released, respectively.

In this study, we focus on exploring the fundamental chemistry of isoindigos as redox-active CO_2_ carriers and their potential to overcome the linear free-energy relationship that limits the structural modification of EMCC sorbents. The flow-based prototype in this work is, however, not an ultimate design for practical systems but a proof-of-concept demonstration to evaluate the performance of isoindigo at the lab scale. We believe future efforts can substantially improve the performance by optimising the electrolytes, electrodes, and membranes of EMCC devices.

### Comparison of methods for estimating CO_2_ binding constants

As a final note, in this manuscript, we estimated the ${K}_{{{{{{{\rm{CO}}}}}}}_{2}}$ of isoindigos using the prevalent method adopted for quinones, bipyridines, and benzyl thiolate^12,21,22^, providing an equitable comparison with previously reported redox-active CO_2_ carriers. Accordingly, we assume that the two-electron-reduced isoindigo binds to one molecule of CO_2_ and calculate the ${K}_{{{{{{{\rm{CO}}}}}}}_{2}}$ based on ∆*E*_peak_(2) under pure N_2_ and CO_2_ atmosphere, respectively (Supplementary Note 3). This method, therefore, eliminates the kinetic effects in CV under lower CO_2_ concentrations. Alternatively, we recorded the CV of IId and 6MCIId under various CO_2_ concentrations (Supplementary Fig. 39) and fitted the relationship between ∆*E*_peak_(2) and CO_2_ concentration (Supplementary Fig. 40 and Supplementary Note 7). Similar ${K}_{{{{{{{\rm{CO}}}}}}}_{2}}$ values were obtained for IId and 6MCIId on the order of 10^12^, further confirming the high tolerance of EWG in isoindigo structural motifs for strong CO_2_ binding. However, the fitting quality was unsatisfactory, with low *R*^2^ values and unreasonable number of binding sites. Therefore, we took the former method to calculate ${K}_{{{{{{{\rm{CO}}}}}}}_{2}}$ for all isoindigos reported in this work.

## Discussion

In summary, we demonstrate the rational design of a class of bifunctional redox-tunable CO_2_ carriers based on isoindigo and their derivatives. The unique intramolecular hydrogen bonding in isoindigo moieties enables a wide range of chemical modifications to facilitate electro-reduction, tune solubility, and preclude parasitic reactions without compromising their CO_2_ binding ability. With coupled experimental and computational studies, we provide an in-depth analysis of the structure-function relationships of isoindigos as EMCC sorbents. Compared to existing EMCC sorbents, isoindigo compounds have the following advantages: 1) a nearly constant $\log {K}_{{{{{{{\rm{CO}}}}}}}_{2}}$ of ~9 with high tolerance to chemical modifications; 2) facile synthesis with the ease of encoding functionalities; 3) highly tunable redox potentials and solubilities; 4) improvable biocompatibility. In addition to flow-based EMCC, we envisage that isoindigos can also find applications as solid adsorbents in fixed-bed systems, due to their descent charge mobility and the abundant methods in synthesising and processing isoindigo-based polymers developed by the community of organic semiconductors. The work paves the way for engineering more reliable EMCC systems by breaking the fundamental barriers of the scaling relationship between redox potential and CO_2_ binding strength when designing redox-tunable CO_2_ sorbents.

## Methods

### Synthesis of 6MCIId

To a mixture of isatin (1.47 g, 10 mmol) and methyl 2-oxindole-6-carboxylate (1.91 g, 10 mmol) in acetic acid (50 ml) was added 37% HCl solution (0.5 ml). The mixture was heated at reflux for 1 day under Argon atmosphere. The mixture was cooled to room temperature, filtered, and washed with water, ethanol, and ethyl acetate. The solid was dried in the vacuum oven at 60 °C for 15 h to afford a dark red powder (2.93 g, 92%). ^1^H NMR (400 MHz, DMSO) δ 11.08 (s, 1H), 10.94 (s, 1H), 9.15 (d, *J* = 8.4 Hz, 1H), 9.07 (d, *J* = 8.0 Hz, 1H), 7.57 (dd, *J* = 8.4, 1.7 Hz, 1H), 7.38 (td, *J* = 7.7, 1.2 Hz, 1H), 7.34 (d, *J* = 1.6 Hz, 1H), 7.02 – 6.94 (m, 1H), 6.85 (d, *J* = 7.3 Hz, 1H), 3.87 (s, 3H). ^13^C NMR (101 MHz, DMSO) δ 168.68, 168.62, 165.57, 144.70, 143.91, 135.73, 133.47, 131.95, 131.79, 129.83, 129.06, 125.68, 121.98, 121.51, 121.28, 109.69, 109.29, 52.29.

### Electrochemical measurements

Electrochemical measurements were performed with a BioLogic VSP potentiostat from BioLogic Science Instruments. Cyclic voltammetry (CV) utilised a glassy carbon electrode (3 mm diameter) as the working electrode, a platinum wire as the counter electrode, and a silver wire as the quasi-reference electrode, with ferrocene as the internal reference. In a standard CV test, isoindigo (2.5 mM) was dissolved in anhydrous DMF with 100 mM NBu_4_PF_6_ as the supporting electrolyte. CV were typically recorded at a scanning rate of −50 mV s^–1^ with a cut-off potential from −1.5 to 0.5 V (prior to ferrocene calibration). To examine the effects of ionic species on the redox behaviour of the sorbent molecules, supporting electrolyte salts including 100 mM LiClO_4_, NaClO_4_, KClO_4_, NBu_4_ClO_4_, sodium triflate (NaOTf), or sodium bis(trifluoromethanesulfonyl)imide (NaTFSI) was employed, respectively. The electrochemical data were gathered and analysed by EC-lab V11.50.

### Flow-based EMCC prototype

In a standard setup, a scintillation vial (20 ml) with a septum cap, serving as the sorbent tank, was continuously purged with CO_2_ feed gas (balanced with N_2_) at a controlled flow rate using an Alicat mass flow controller. Simulated flue gas conditions were mimicked using a 10% CO_2_ and 3% O_2_ mix, balanced by N_2_. CO_2_ levels were continuously monitored at the gas outlet using an infra-red-based CO_2_ sensor (SprintIR-W 100%), with data recorded via Labview 2021. To minimise mixing time in the overhead space, the tank was filled with plastic beads from McMaster-Carr. The Fc tank was maintained without air exposure. Sorbent and Fc electrolytes were circulated at a flow rate of 10 ml min^–1^ through a commercial flow cell (Scribner) by a two-channel peristaltic pump (Masterflex). The flow cell incorporated two graphite plates with 5 cm^2^ interdigitated flow fields, pressing against two pieces of carbon paper electrodes (Sigracet 28 AA) on each side of the graphite electrodes. A Nafion 115 membrane, flanked by polypropylene sheets, was placed between the carbon electrodes. The cell was sealed by Kalrez fluoropolymer elastomer gaskets (0.02-inch thick). CO_2_ capture was conducted in constant current mode, while release followed a constant current/constant voltage protocol: the adduct was first oxidised at a constant current until reaching a cut-off voltage and then held at that voltage until the current value fell below 5% of the original constant current). For experiments using 5 cm^2^ flow fields, the CO_2_ capture-release was cycled at a current of 10 mA with a cut-off potential of 0.05 V in the release process. For experiments using 25 cm^2^ flow fields, the CO_2_ capture-release was conducted at a current of 50 mA with a cut-off potential of 0.3 V in the release process. The cycling protocols of each experiment are provided in the corresponding figure captions. For experiments using 6MCIId sorbent, we added 10 mM 6MCIId and 4 mM ferrocenium tetrafluoroboronate (FcBF_4_) into the CE tank to mitigate sorbent crossover and facilitate the reduction of the oxidised ferrocenium in the counter electrolyte, respectively. For less soluble 55DBIId and 66DBIId, the sorbent was dispersed in dimethylacetamide (DMAc) to form a slurry catholyte.

### Reporting summary

Further information on research design is available in the Nature Portfolio Reporting Summary linked to this article.

### Supplementary information

Supplementary Information

Peer Review File

Reporting Summary

### Source data

Source Data

## Data Availability

The data generated or analysed during this study are included in the manuscript and its Supplementary Information. The main data generated in this study are provided in the Supplementary Information/Source Data file. Data are also available from the corresponding author upon request. Source data are provided with this paper.
