# Supplementary material for: Redox-tunable isoindigos for electrochemically mediated carbon capture
Source: Nat Commun. 2024 Feb 8;15:1175. doi: 10.1038/s41467-024-45410-z (PMC10853560; doi:10.1038/s41467-024-45410-z)
Supplement: Supplementary file 1 — Supplementary Information [file 41467_2024_45410_MOESM1_ESM.pdf]

Supplementary Information

Redox-Tunable Isoindigos for Electrochemically Mediated Carbon Capture

Xing Li[1], Xunhua Zhao[2,3], Lingyu Zhang[1], Anmol Mathur[1], Yu Xu[1], Zhiwei Fang[4], Luo Gu[4], Yuanyue Liu[2], Yayuan Liu[1*]

[1] Department of Chemical and Biomolecular Engineering, Johns Hopkins University, Baltimore, Maryland, 21218, United States

[2] Department of Mechanical Engineering & Texas Materials Institute, The University of Texas at Austin, Austin, Texas, 78712, United States

[3] Macao Institute of Materials Science and Engineering (MIMSE), Faculty of Innovation Engineering, Macau University of Science and Technology, Taipa, Macau, 999078, China

[4] Department of Materials Science and Engineering, Johns Hopkins University, Baltimore, Maryland, 21218, United States

*Correspondence to: yayuanliu@jhu.edu

Table of Contents

Supplementary Figures 3
Supplementary Tables 43
Supplementary Methods 49
Synthesis of isoindigos 50
Bulk electrolysis 86
Nafion membrane pretreatment 88
DFT calculations 88
Cell culture 88
Cytotoxicity assay and cell morphology 88
Supplementary Notes 90
Supplementary References 97

Supplementary Figures

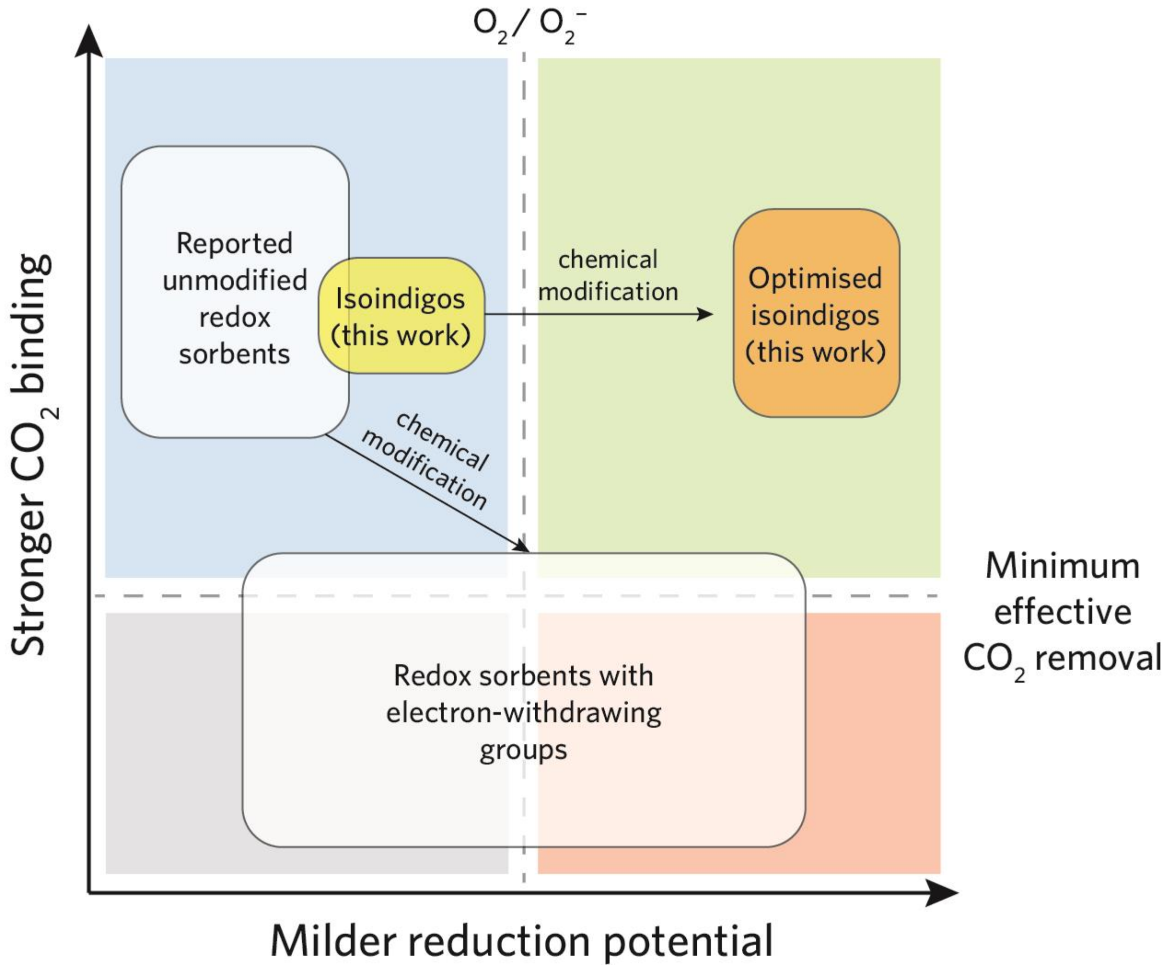

Supplementary Fig. 1 | A diagram showing the current limitations of organic redox-active CO_2 sorbents for EMCC and how the bifunctional isoindigos overcome this constraint.

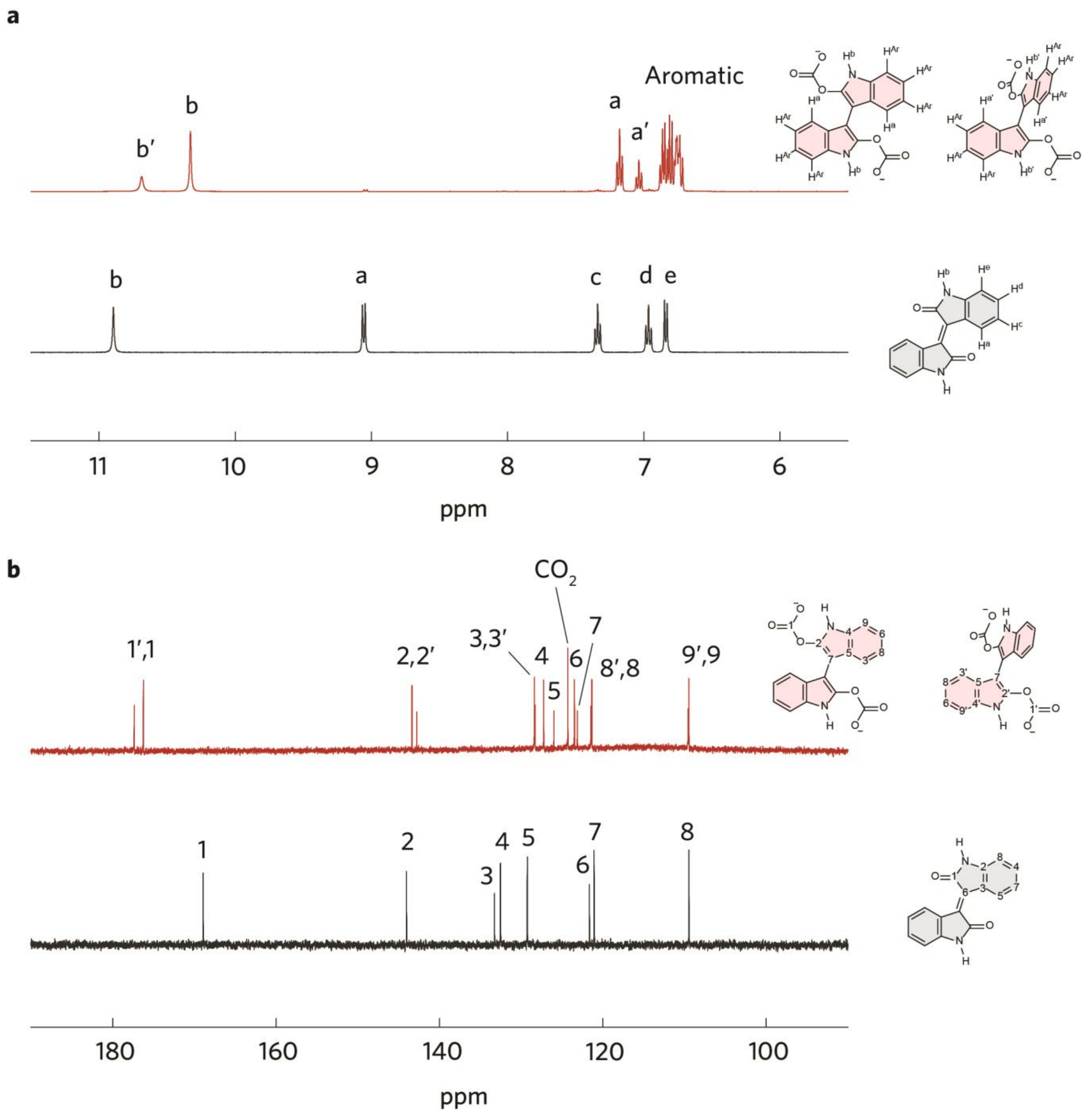

Supplementary Fig. 2 | Full assignment of the NMR spectra of IId (black) and IId-CO_2 (red). a, ^{1}H NMR. **b**, ^{13}C NMR.

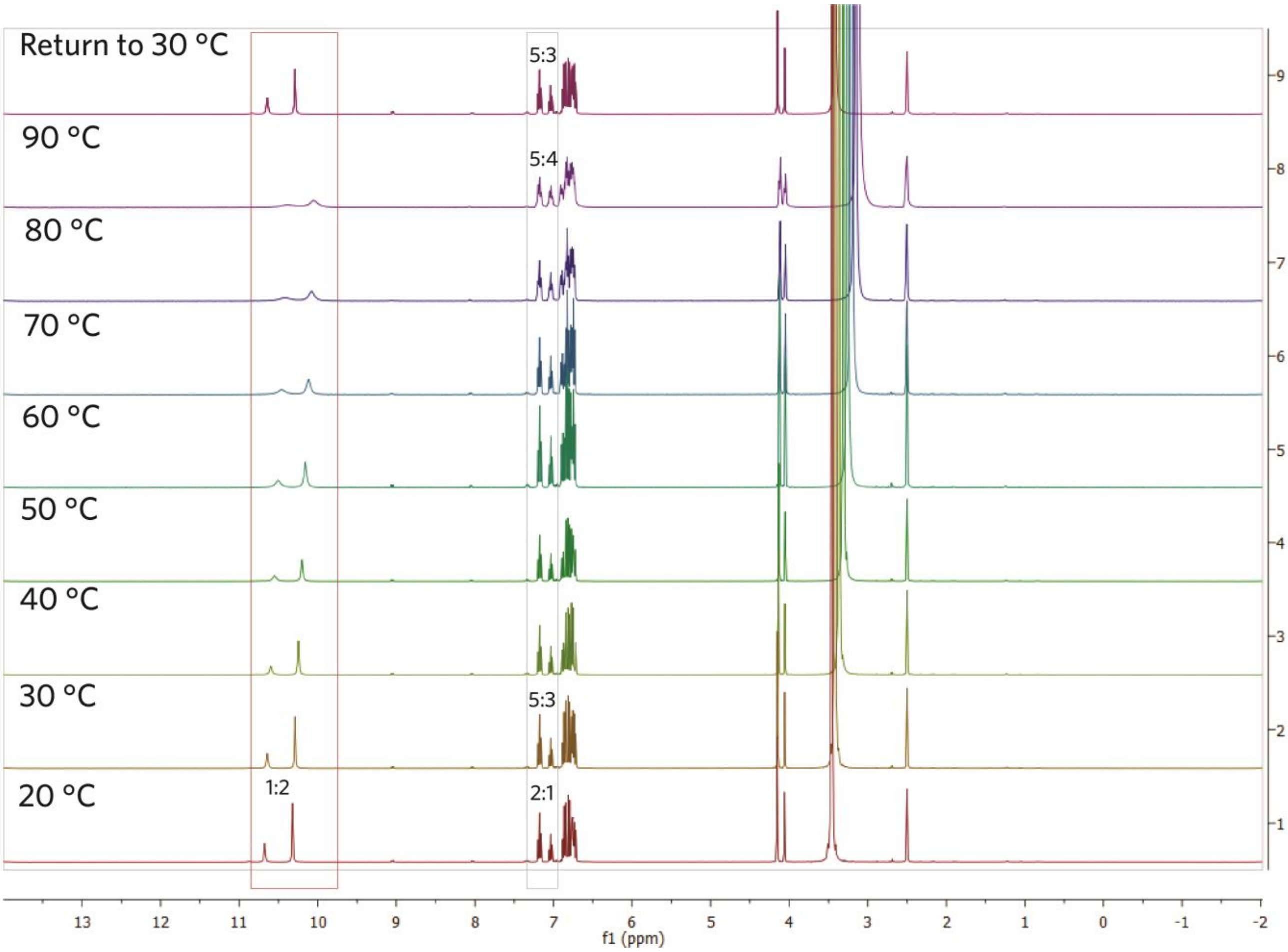

Supplementary Fig. 3 | Variable-Temperature ^{1}H NMR spectra of the crude solution of IId after the bulk reduction in CO_2.

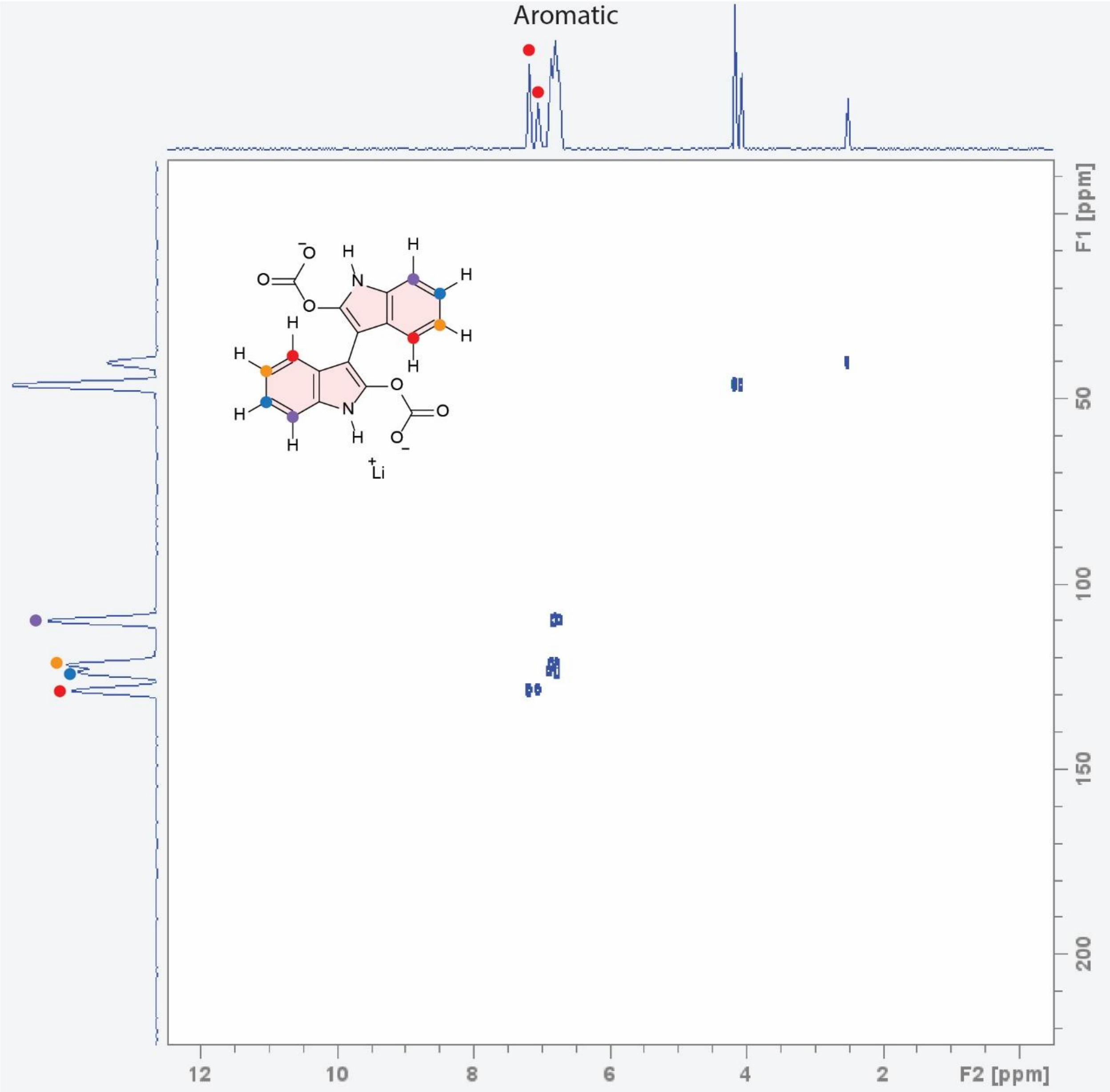

Supplementary Fig. 4 | 2D ^{1}H-^{13}C Heteronuclear Single Quantum Coherence (HSQC) NMR of the crude solution after bulk reduction of IId in CO_2.

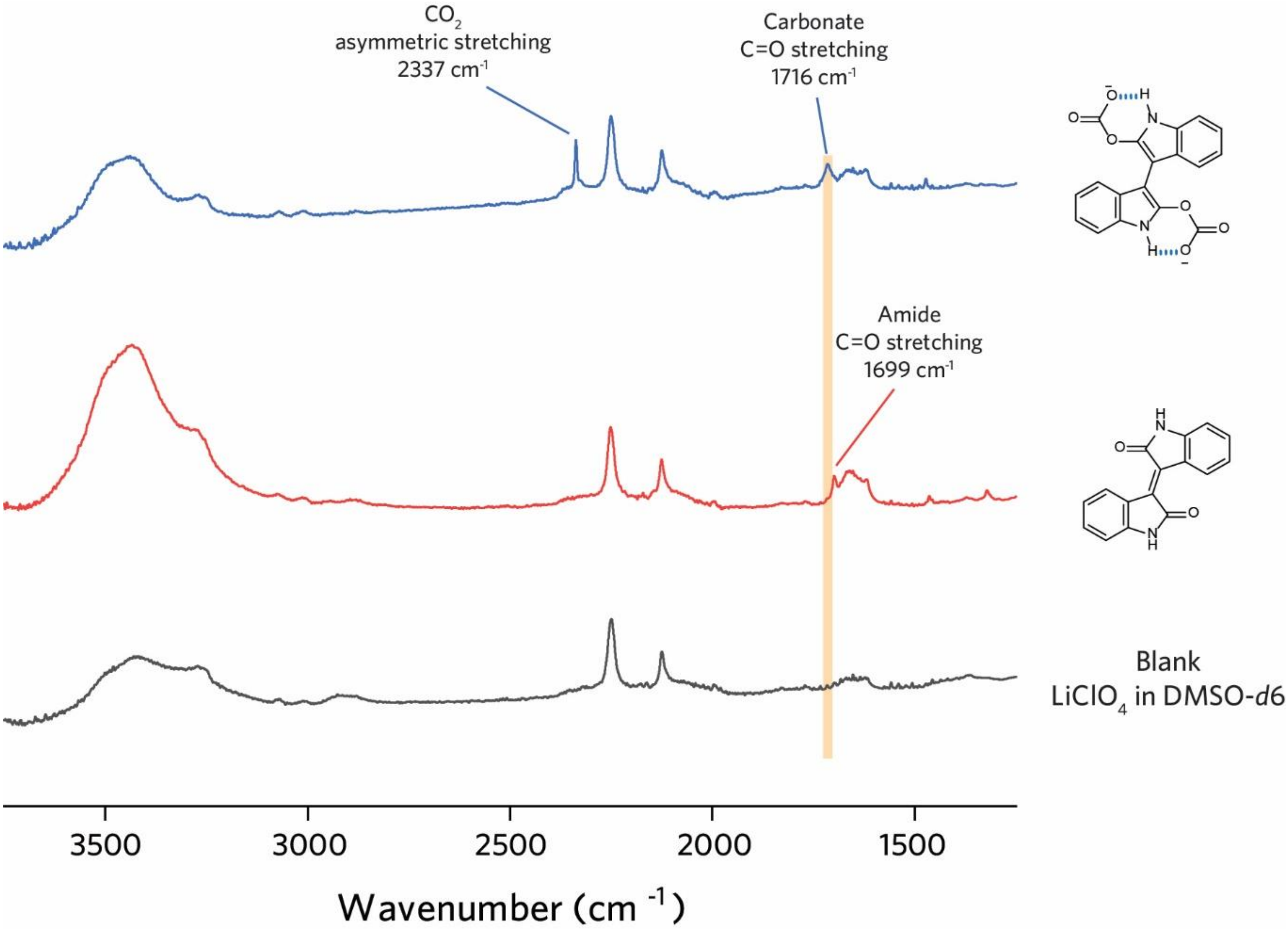

Supplementary Fig. 5 | FT-IR spectra of IId and the IId-CO_2 adduct after bulk electrolysis in DMSO with $LiClO_4$ as the supporting salt.

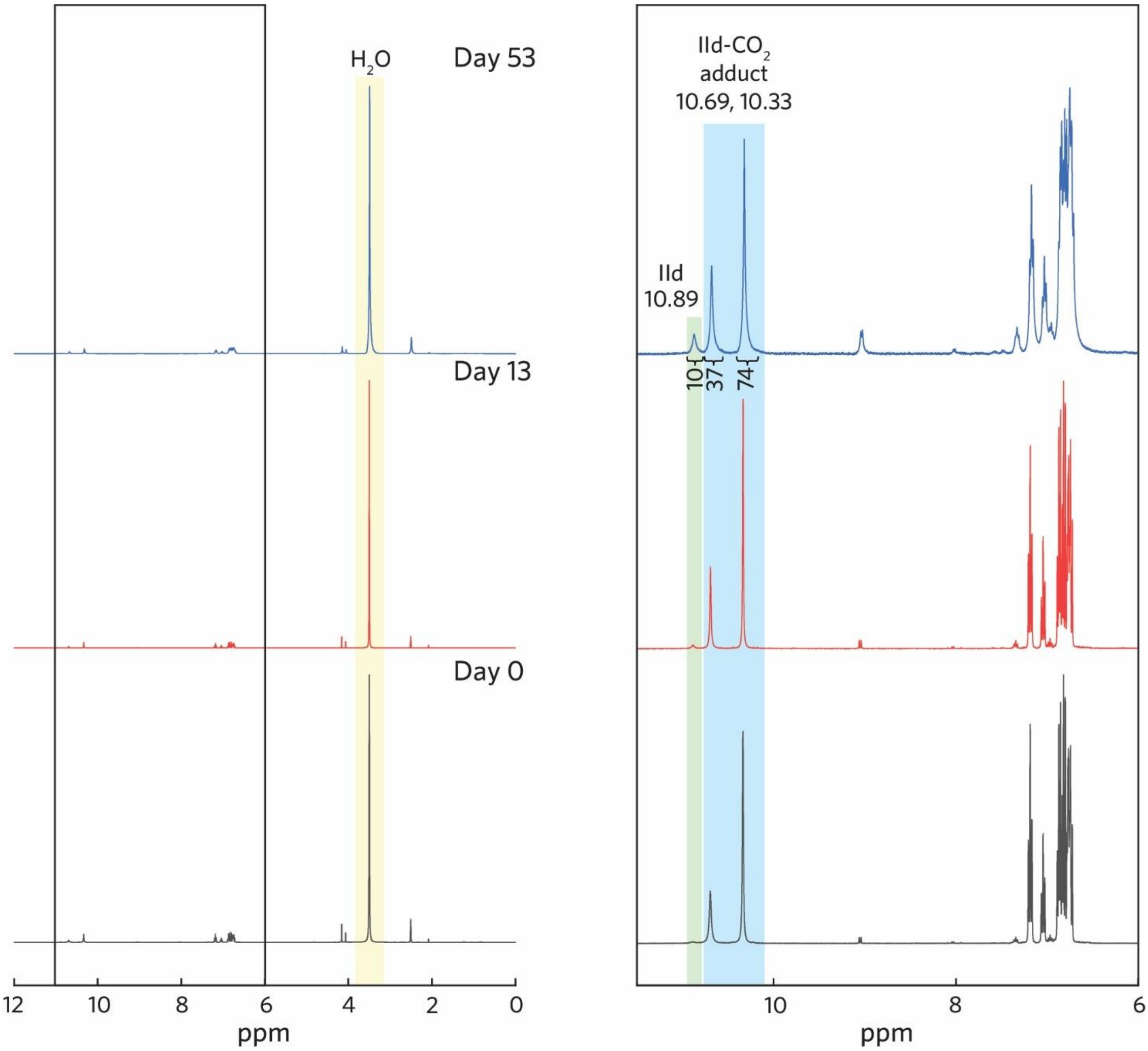

Supplementary Fig. 6 | Stability test of the isoindigo-CO_2 adduct. Less than 9 mol% of the IId-CO_2 adduct was oxidised back to IId after the solution was stored in the air in the presence of high water content for 53 days (based on ^{1}H NMR relative integrals).

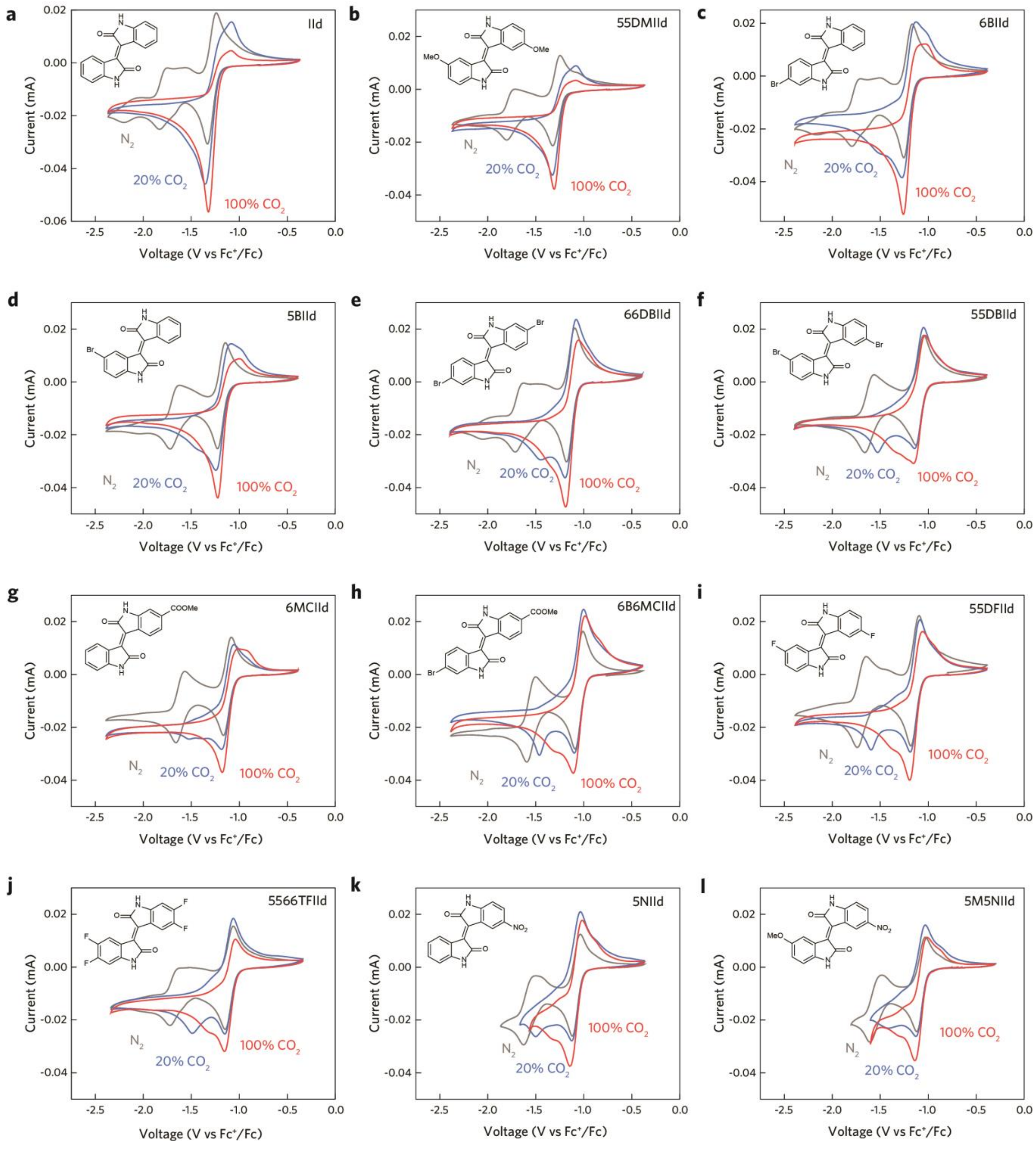

Supplementary Fig. 7 | CVs of isoindigos with various chemical modifications under different atmospheres. **a**, IId. **b**, 55DMIId. **c**, 6BIId. **d**, 5BIId. **e**, 66DBIId. **f**, 55DBIId. **g**, 6MCIId. **h**, 6B6MCIId. **i**, 55DFIId. **j**, 5566TFIId. **k**, 5NIId. **l**, 5M5NIId. The CV was recorded using 2.5 mM compound in anhydrous DMF with 0.1 M NBu_4PF_6 saturated by N_2 (grey), 20% CO_2 (balanced with N_2, blue), and CO_2 (red), respectively, at a scan rate of −50 mV s^{-1} at 298 K.

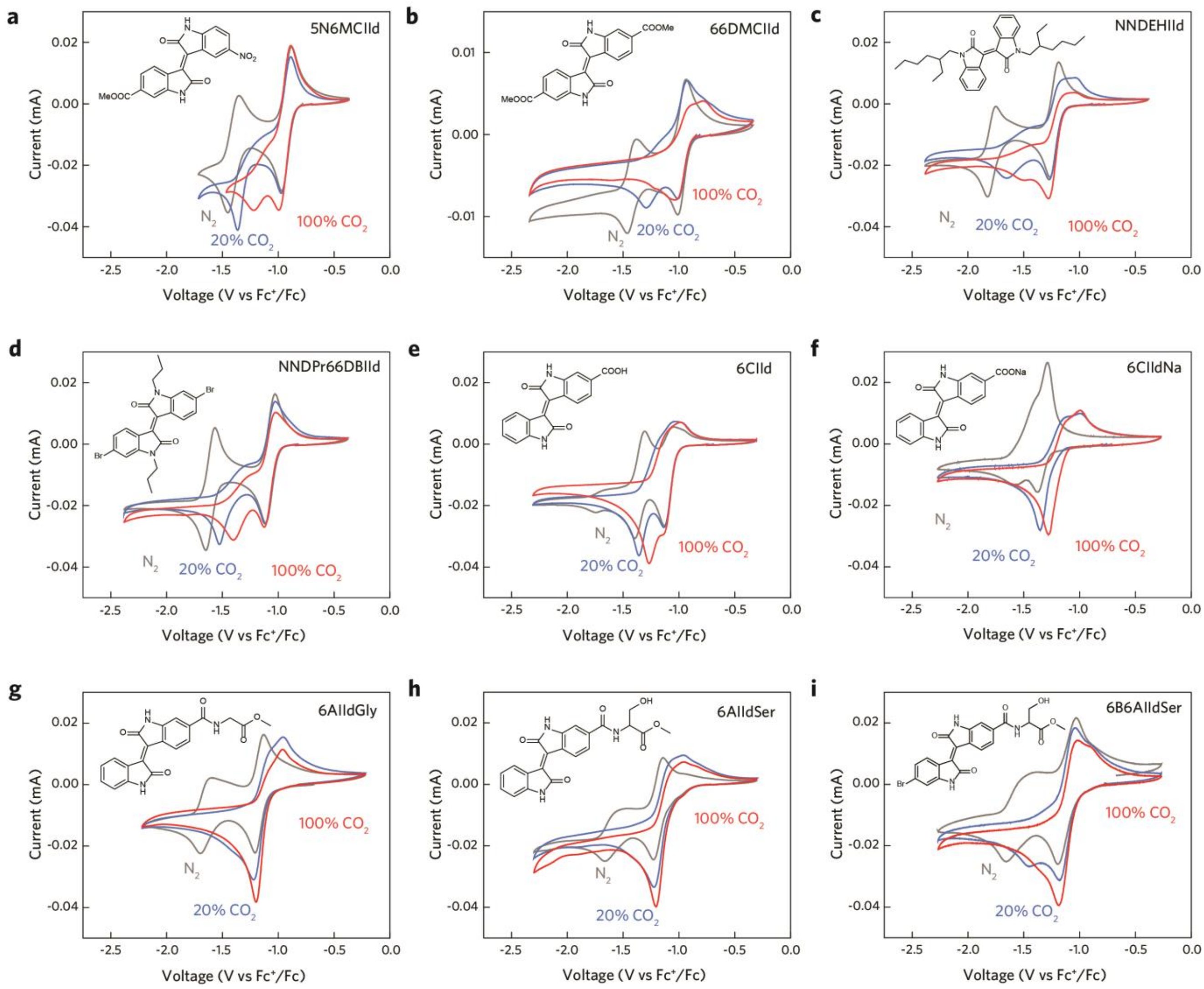

Supplementary Fig. 8 | CVs of isoindigos with various chemical modifications under different atmospheres. **a**, 5N6MCIId. **b**, 66DMCIId. **c**, NNDEHIId. **d**, NNDPr66DBIId. **e**, 6CIId. **f**, 6CIIdNa. **g**, 6AIIdGly. **h**, 6AIIdSer. **i**, 6B6AIIdSer. The CV was recorded using 2.5 mM compound in anhydrous DMF with 0.1 M NBu_4PF_6 saturated by N_2 (grey), 20% CO_2 (balanced with N_2, blue), and CO_2 (red), respectively, at a scan rate of -50 mV s^{-1} at 298 K.

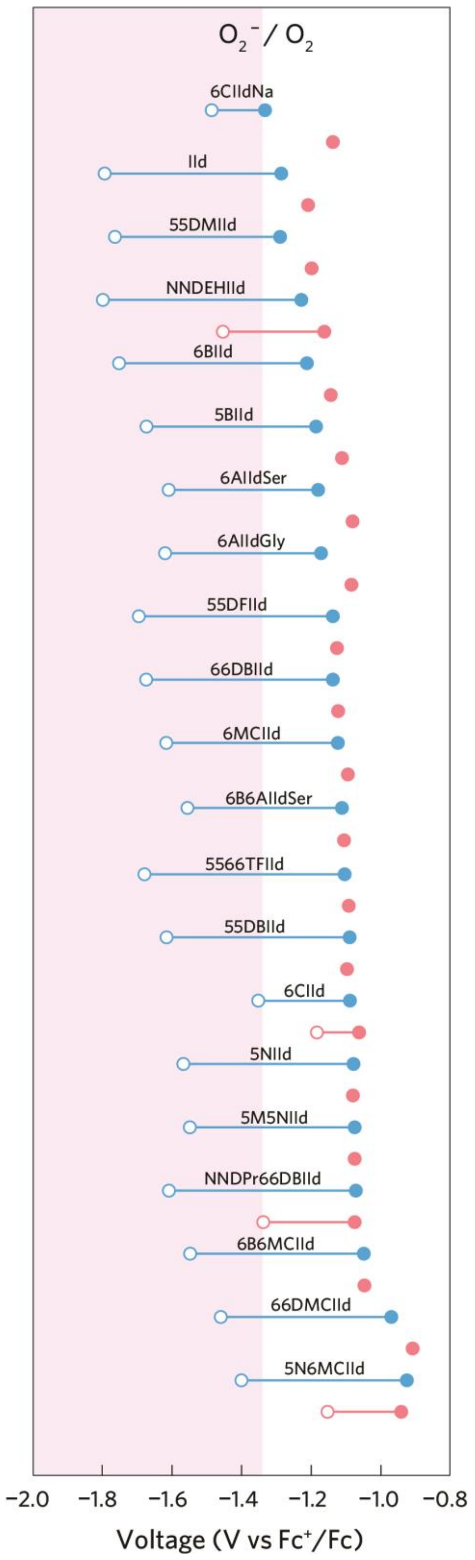

Supplementary Fig. 9 | Tabulated half-wave potentials of isoindigos under N_2 or CO_2. Half-wave potentials are summarised from the CVs of various isoindigos using 2.5 mM compound in DMF with 0.1 M NBu_4PF_6 under N_2 (blue) or CO_2 (pink) (filled circle: first redox potential; empty circle: second redox potential). The oxygen reduction reaction to superoxide occurs at −1.35 V vs. Fc^+/Fc in DMF.

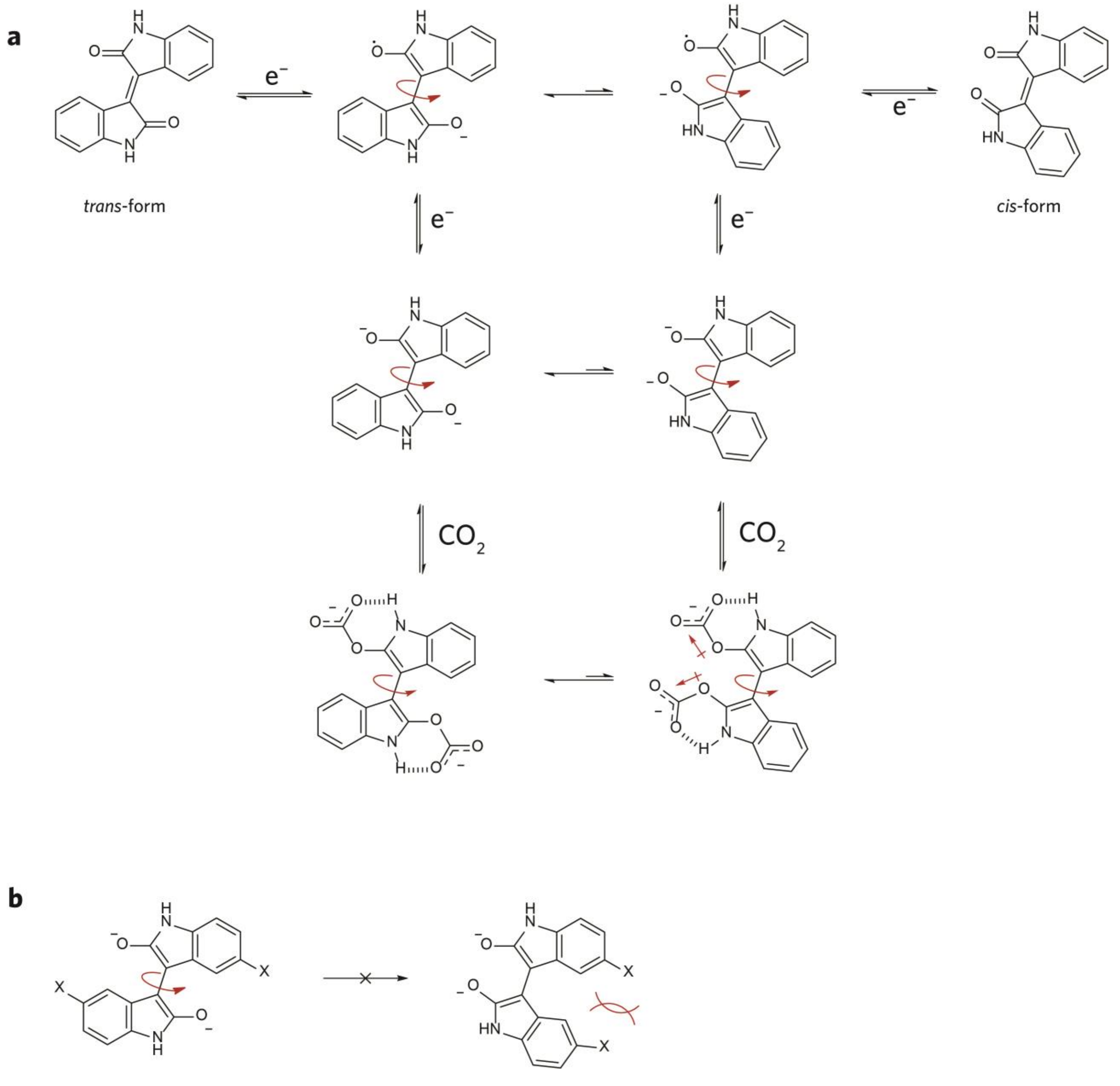

Supplementary Fig. 10 | Proposed mechanism for the rotational isomerisation of isoindigo radical anion.

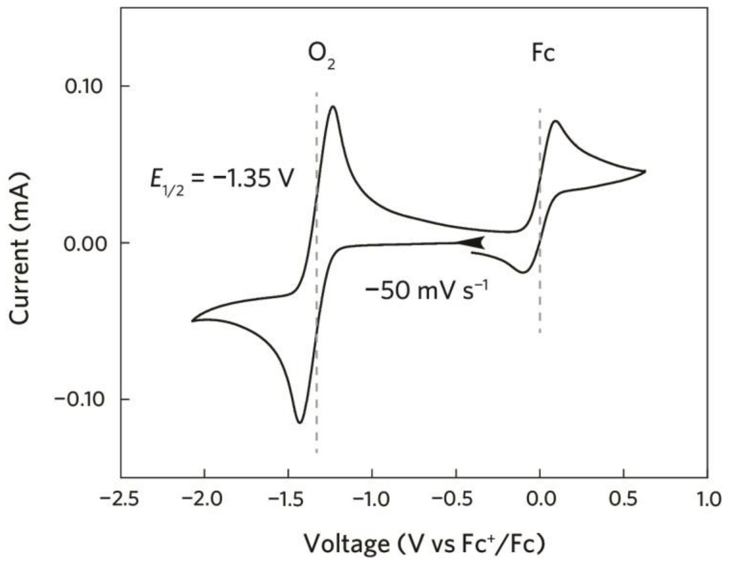

Supplementary Fig. 11 | CV of O_2 saturated DMF with 0.1 M NBu_4PF_6 at a scan rate of −50 mV s^{-1} with a cathodic sweeping direction initially at 298 K.

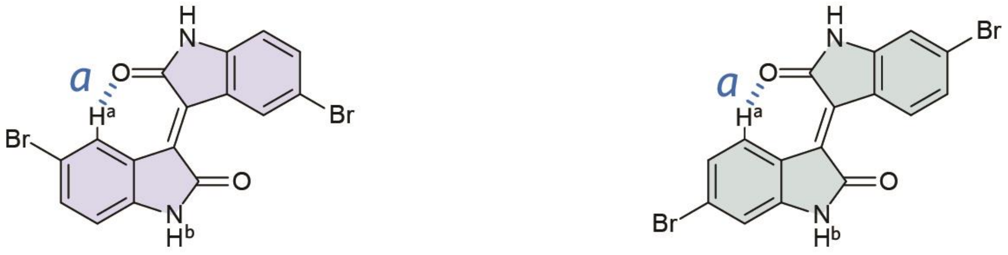

55DBIId

H^a = 9.32 ppm

H^b = 11.11 ppm

$E_{1/2}$(IId/IId•⁻) = −1.09 V

66DBIId

H^a = 8.99 ppm

H^b = 11.10 ppm

$E_{1/2}$(IId/IId•⁻) = −1.12 V

Supplementary Fig. 12 | A summary of characteristic ^{1}H NMR for H^a and H^b and half-wave redox potential of 55DBIId and 66DBIId under CO_2.

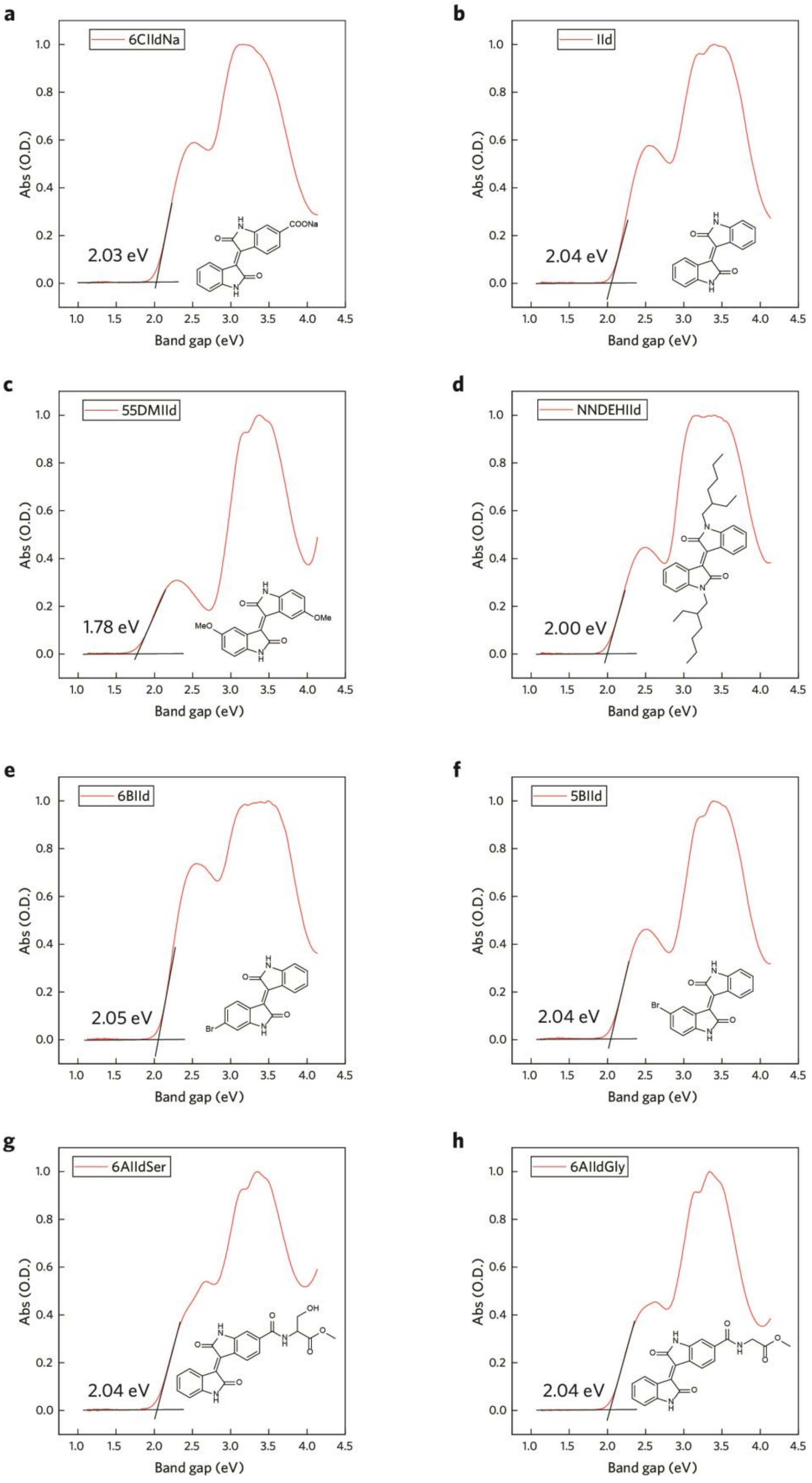

Supplementary Fig. 13 | Kubelka-Munk spectra of various isoindigos.

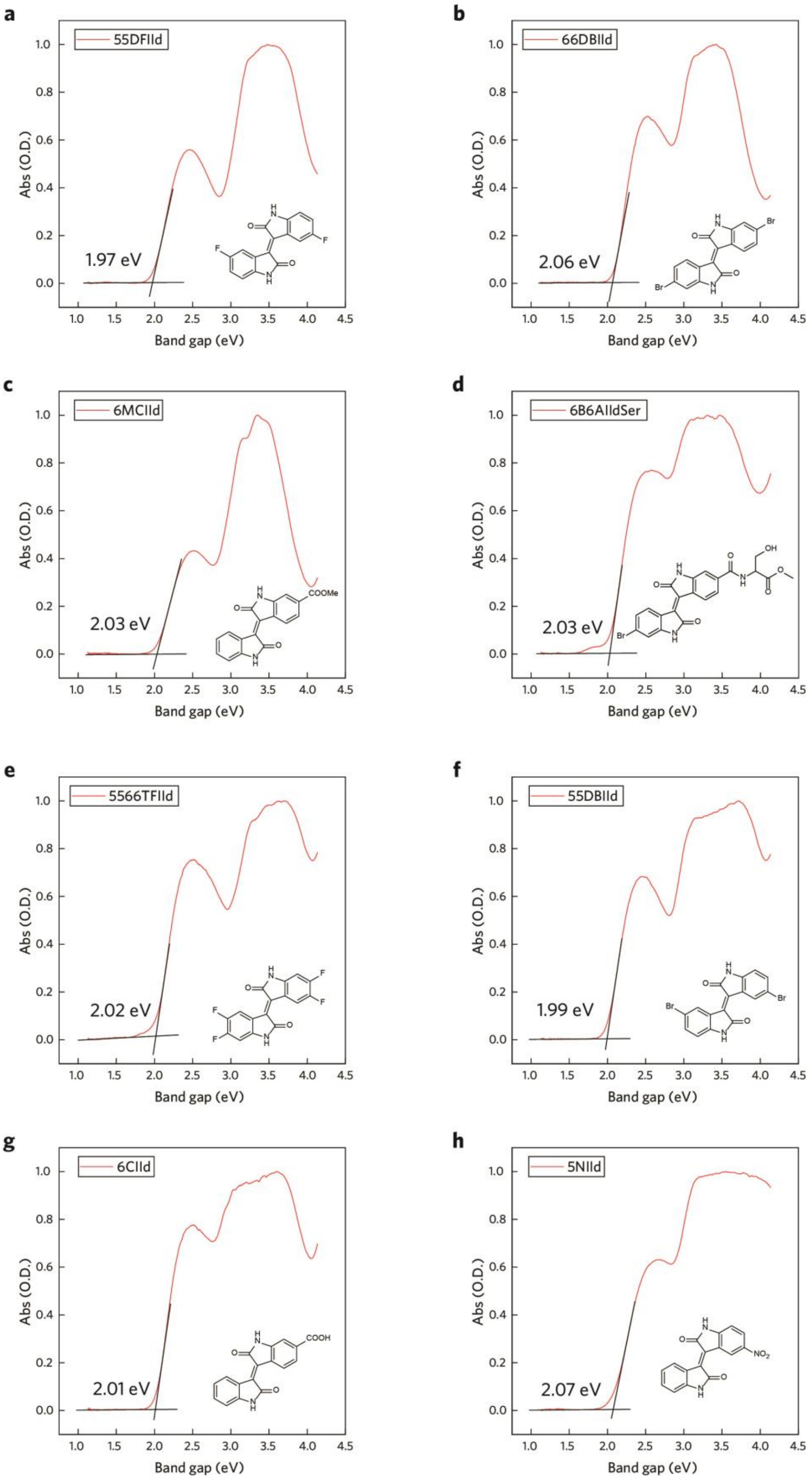

Supplementary Fig. 14 | Kubelka-Munk spectra of various isoindigos.

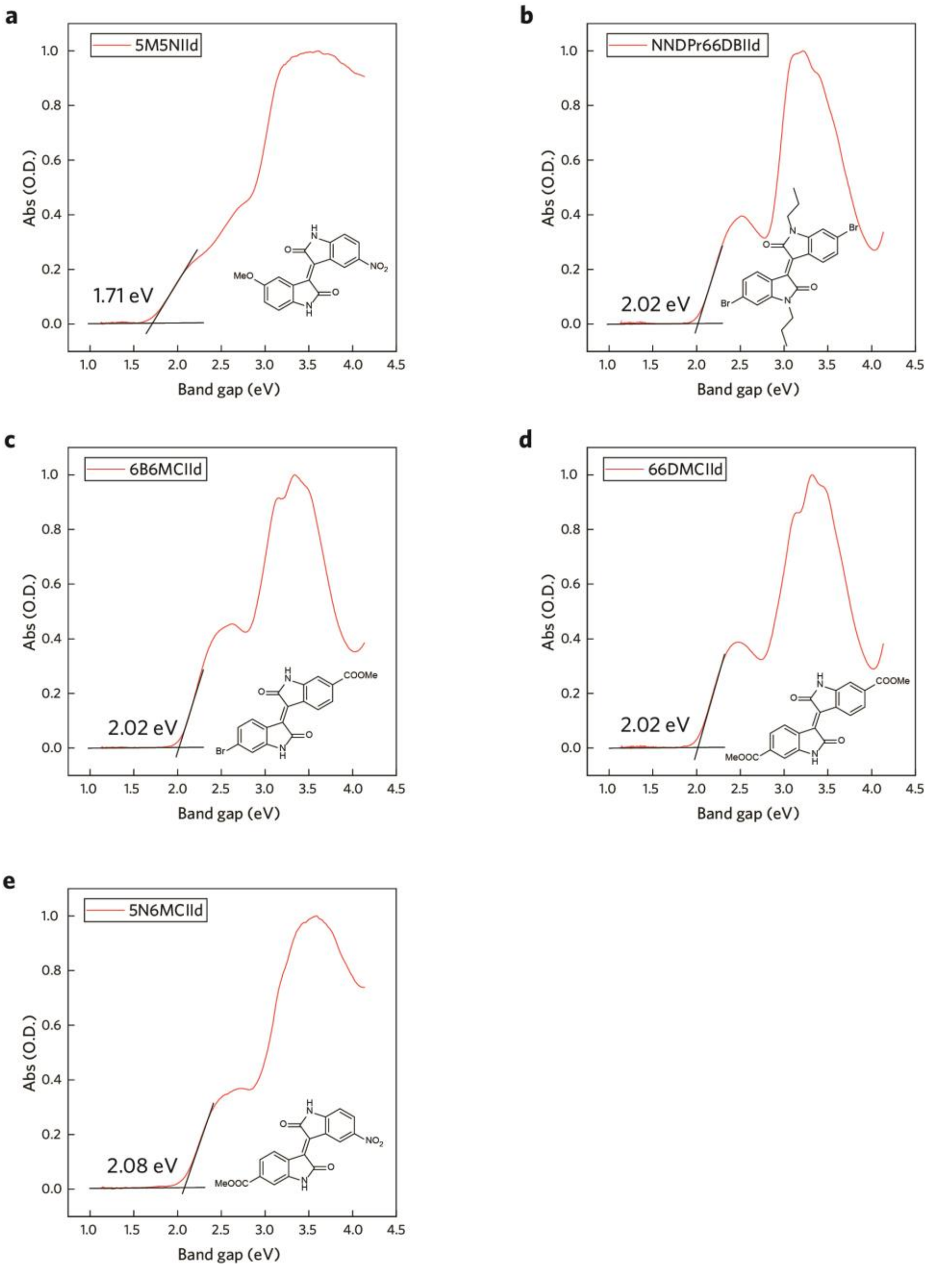

Supplementary Fig. 15 | Kubelka-Munk spectra of various isoindigos.

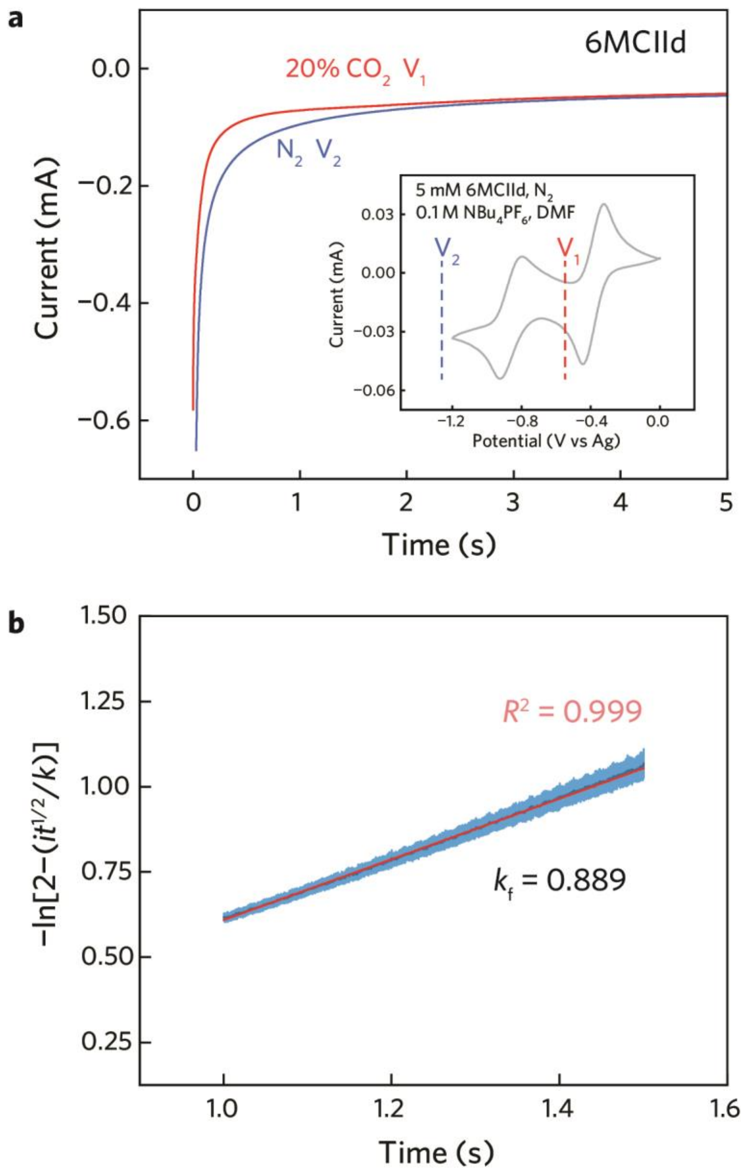

Supplementary Fig. 16 | Calculation of the reaction rate constant between 6MCIId radical anion and CO_2. a, Plots of current vs. time for 5 mM 6MCIId in DMF with 0.1 M NBu_4PF_6 as the supporting salt for potential steps from open circuit voltage to –0.55 V vs. Ag (V_1) with 20% CO_2, and from open circuit voltage to –1.26 V vs. Ag (V_2) without CO_2. The inset is the CV of the electrolyte under N_2 with the corresponding voltage steps (V_1 and V_2). **b**, Determination of the forward rate constant (k_f) for the addition of CO_2 to 6MCIId$^{\bullet-}$. The measurements were done in triplicate, and the data is shown for the time interval where the current is between one- and two-electron transfer processes. The slope of the plot gives k_f.

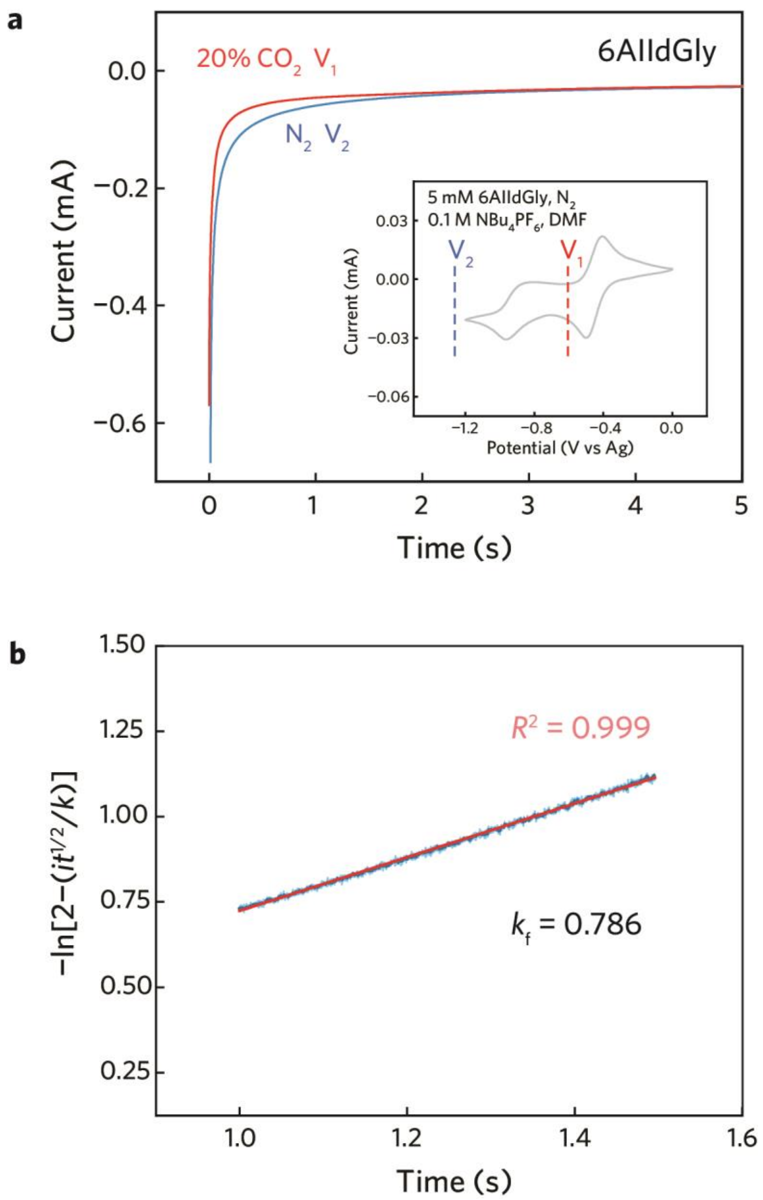

Supplementary Fig. 17 | Calculation of the reaction rate constant between 6AIIdGly radical anion and CO_2. a, Plots of current vs. time for 5 mM 6AIIdGly in DMF with 0.1 M NBu_4PF_6 as the supporting salt for potential steps from open circuit voltage to –0.6 V vs. Ag (V_1) with 20% CO_2, and from open circuit voltage to –1.282 V vs. Ag (V_2) without CO_2. The inset is the CV of the electrolyte under N_2 with the corresponding voltage steps (V_1 and V_2). **b**, Determination of the forward rate constant (k_f) for the addition of CO_2 to 6AIIdGly$^{\bullet-}$. The measurements were done in triplicate, and the data is shown for the time interval where the current is between one- and two-electron transfer processes. The slope of the plot gives k_f.

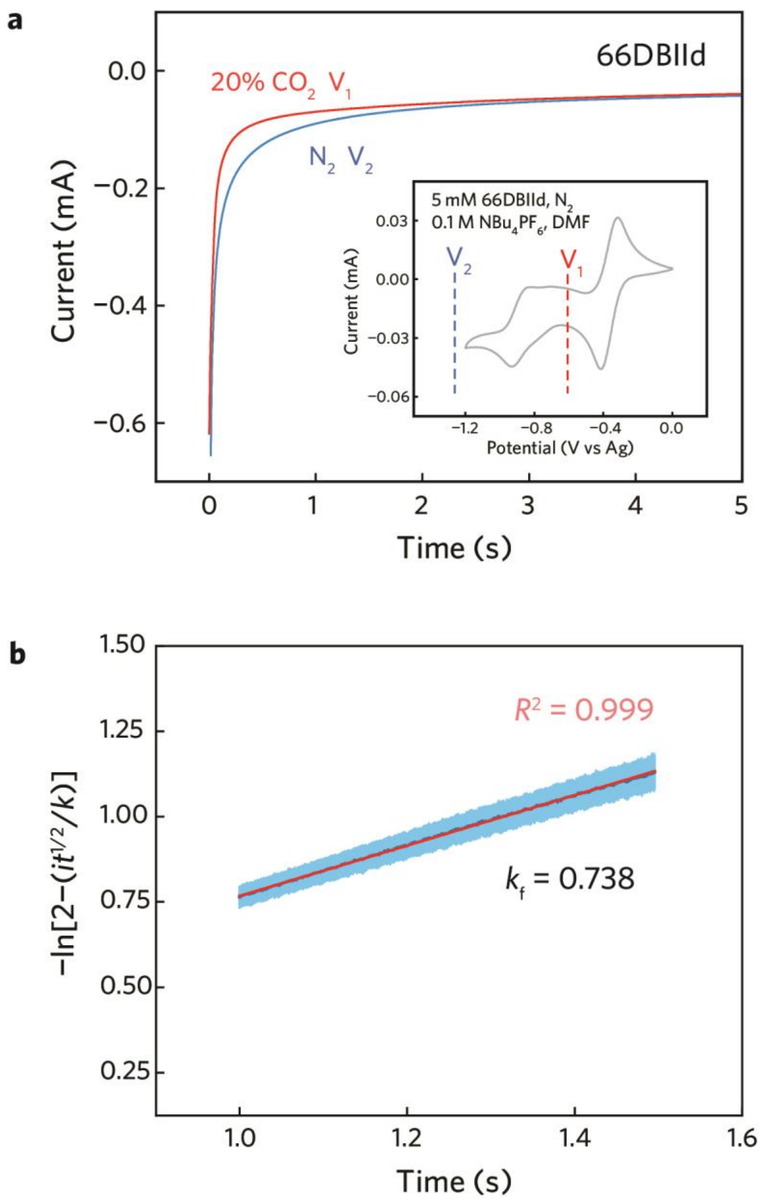

Supplementary Fig. 18 | Calculation of the reaction rate constant between 66DBIId radical anion and CO_2. a, Plots of current vs time for 5 mM 66DBIId in DMF with 0.1 M NBu_4PF_6 as the supporting salt for potential steps from open circuit voltage to –0.6 V vs Ag (V_1) with 20% CO_2, and from open circuit voltage to –1.275 V vs Ag (V_2) without CO_2. The inset is the cyclic voltammogram of the electrolyte under N_2 with the corresponding voltage steps (V_1 and V_2). **b**, Determination of the forward rate constant (k_f) for the addition of CO_2 to 66DBIId$^{\bullet-}$. The measurements were done in triplicate, and the data is shown for the time interval where the current is between that of one- and two-electron transfer processes. The slope of the plot gives k_f.

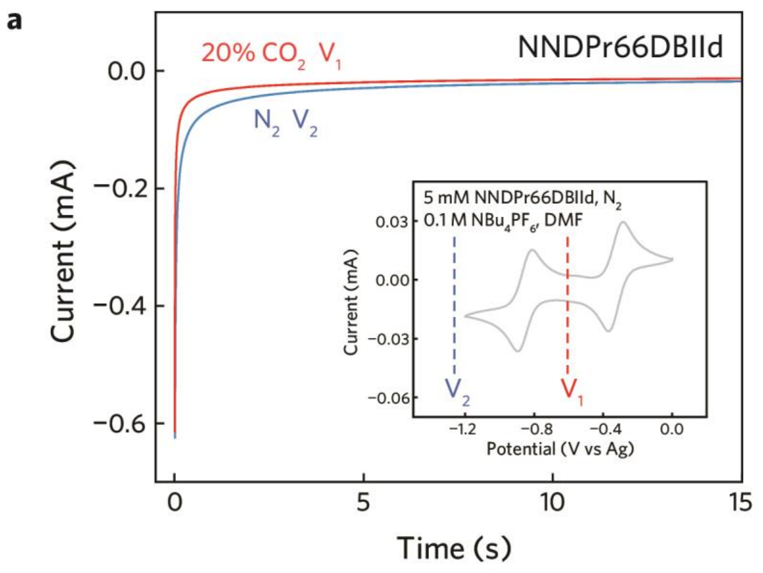

Supplementary Fig. 19 | Calculation of the reaction rate constant between NNDPr66DBIId radical anion and CO_2. a, Plots of current vs time for 5 mM NNDPr66DBIId in DMF with 0.1 M NBu_4PF_6 as the supporting salt for potential steps from open circuit voltage to –0.6 V vs Ag (V_1) with 20% CO_2, and from open circuit voltage to –1.252 V vs Ag (V_2) without CO_2. The inset is the cyclic voltammogram of the electrolyte under N_2 with the corresponding voltage steps (V_1 and V_2). **b**, Determination of the forward rate constant (k_f) for the addition of CO_2 to NNDPr66DBIId$^{\bullet-}$. The measurements were done in triplicate, and the data is shown for the time interval where the current is between that of one- and two-electron transfer processes. The slope of the plot gives k_f.

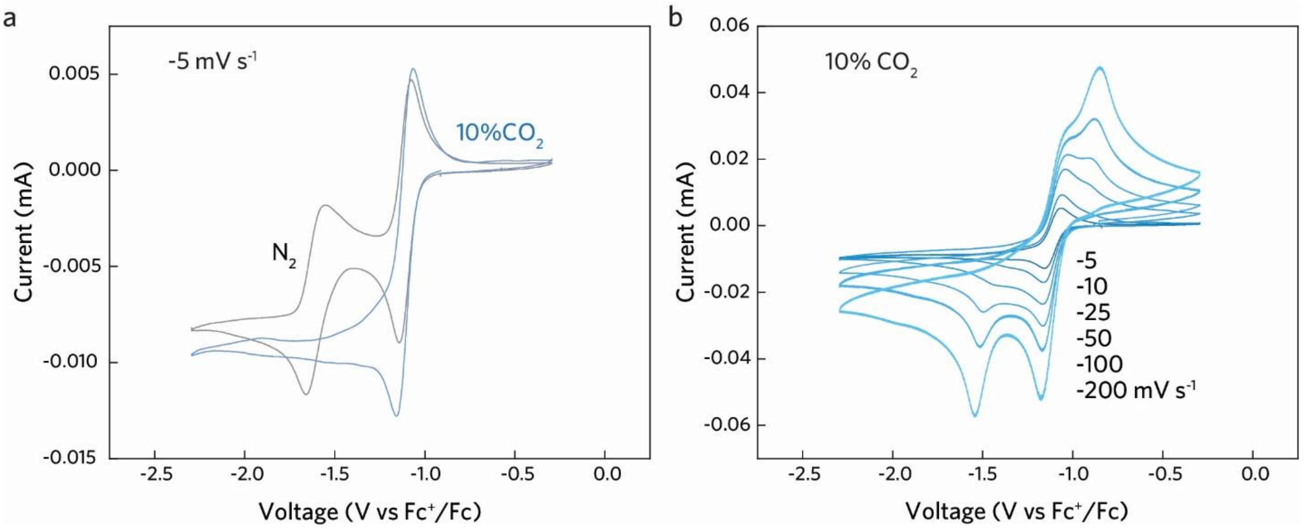

Supplementary Fig. 20 | CV of 6MCIId under different atmospheres or at different scan rates. **a**, CV curves under N_2 or 10% CO_2 at a cathodic scan rate of −5 mV s^{-1}. **b**, CV curves under 10% CO_2 at various scan rates from −5 to −200 mV s^{-1}. The CV curves were recorded using 2.5 mM 6MCIId in anhydrous DMF with 0.1 M NBu_4PF_6.

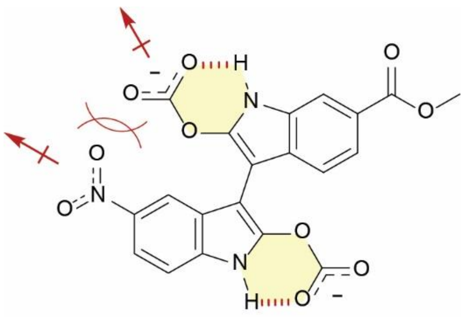

Supplementary Fig. 21 | Proposed mechanism of CO_2 binding inhibition in 5N6MCIId.

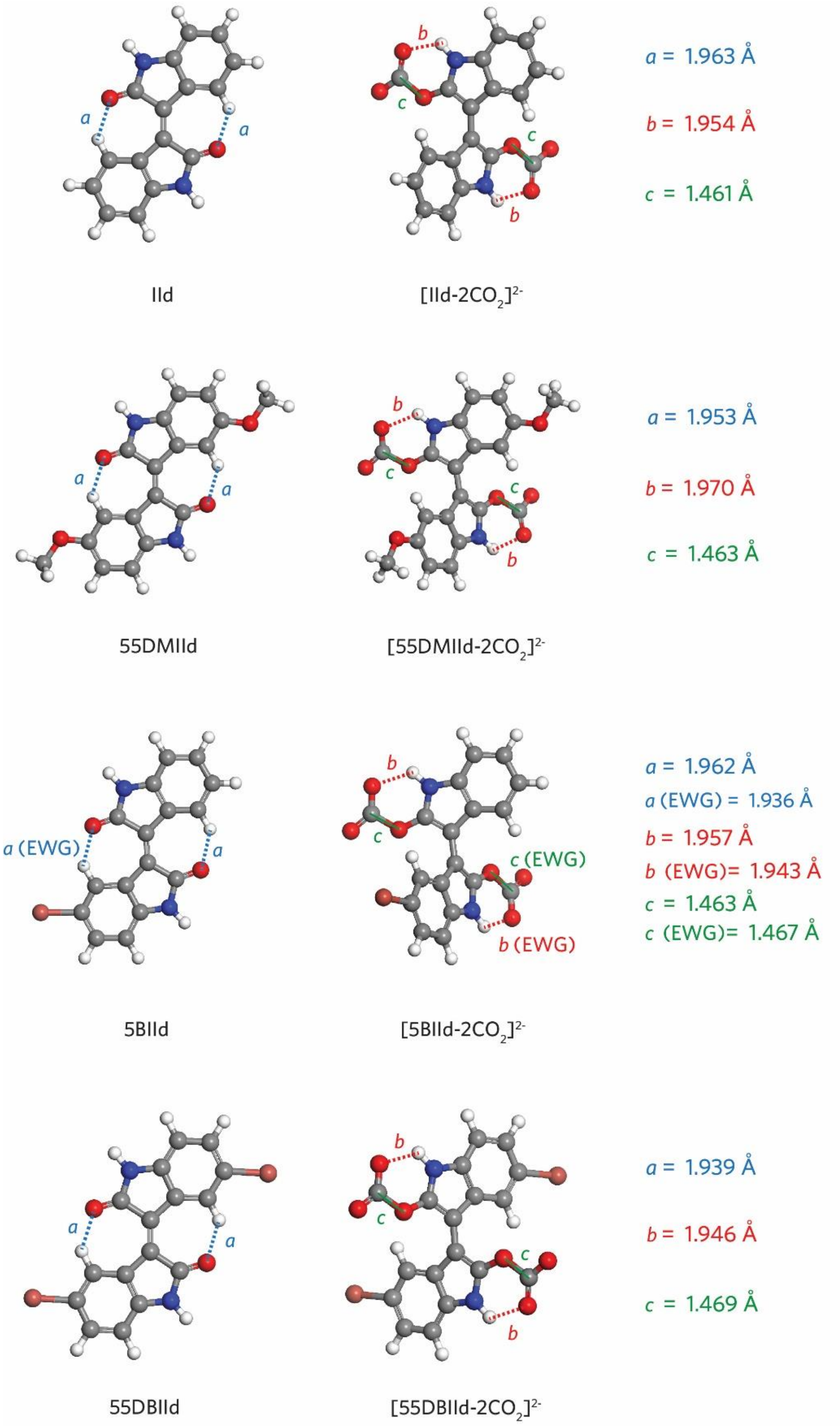

Supplementary Fig. 22 | DFT-optimised structures of isoindigos in the neutral state and CO_2-complexed state (colour of atoms: grey: C; red: O; blue: N; brown: Br; white: H).

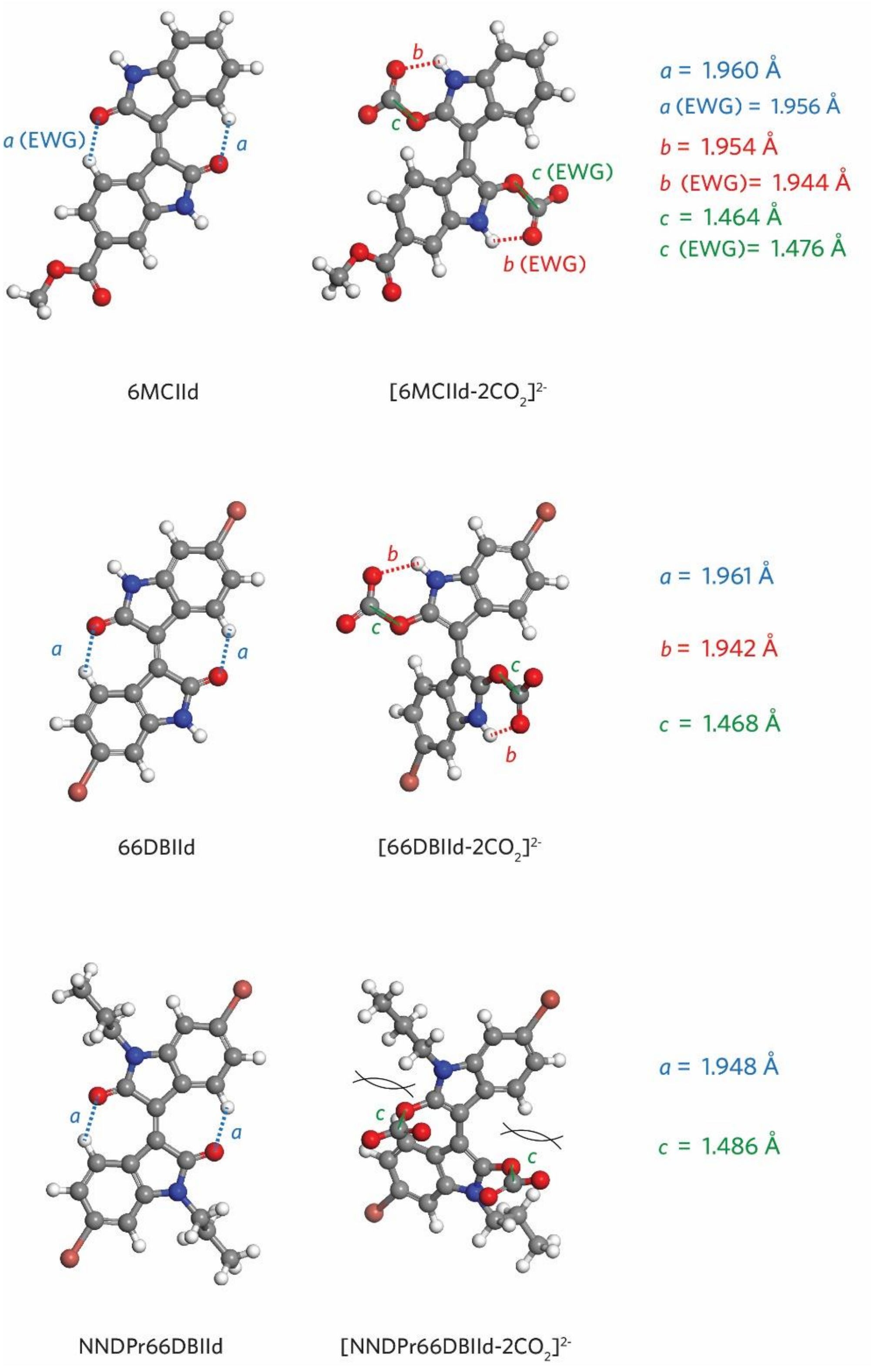

Supplementary Fig. 23 | DFT-optimised structures of isoindigos in the neutral state and CO_2-complexed state (colour of atoms: grey: C; red: O; blue: N; brown: Br; white: H).

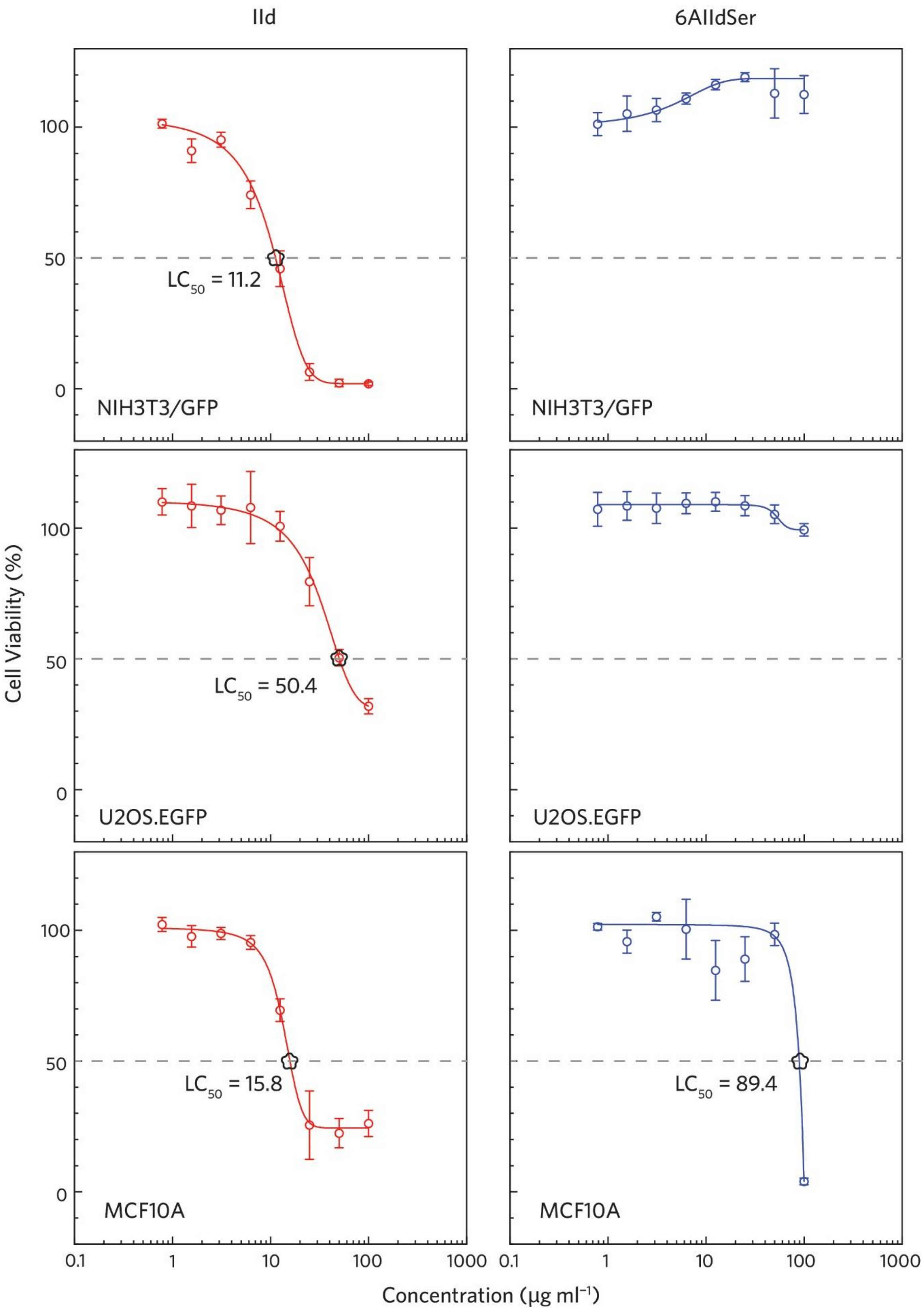

Supplementary Fig. 24 | Cytotoxicity test of IId and 6AIIdSer using different mammalian cells. Cells were cultured in the absence or presence of isoindigos under various concentrations from 0.78 to 100 μg ml^{-1} for 48 h. The number of replicates (n=3). The amino ester modified 6AIIdSer exhibits much better biocompatibility than its unmodified counterparts.

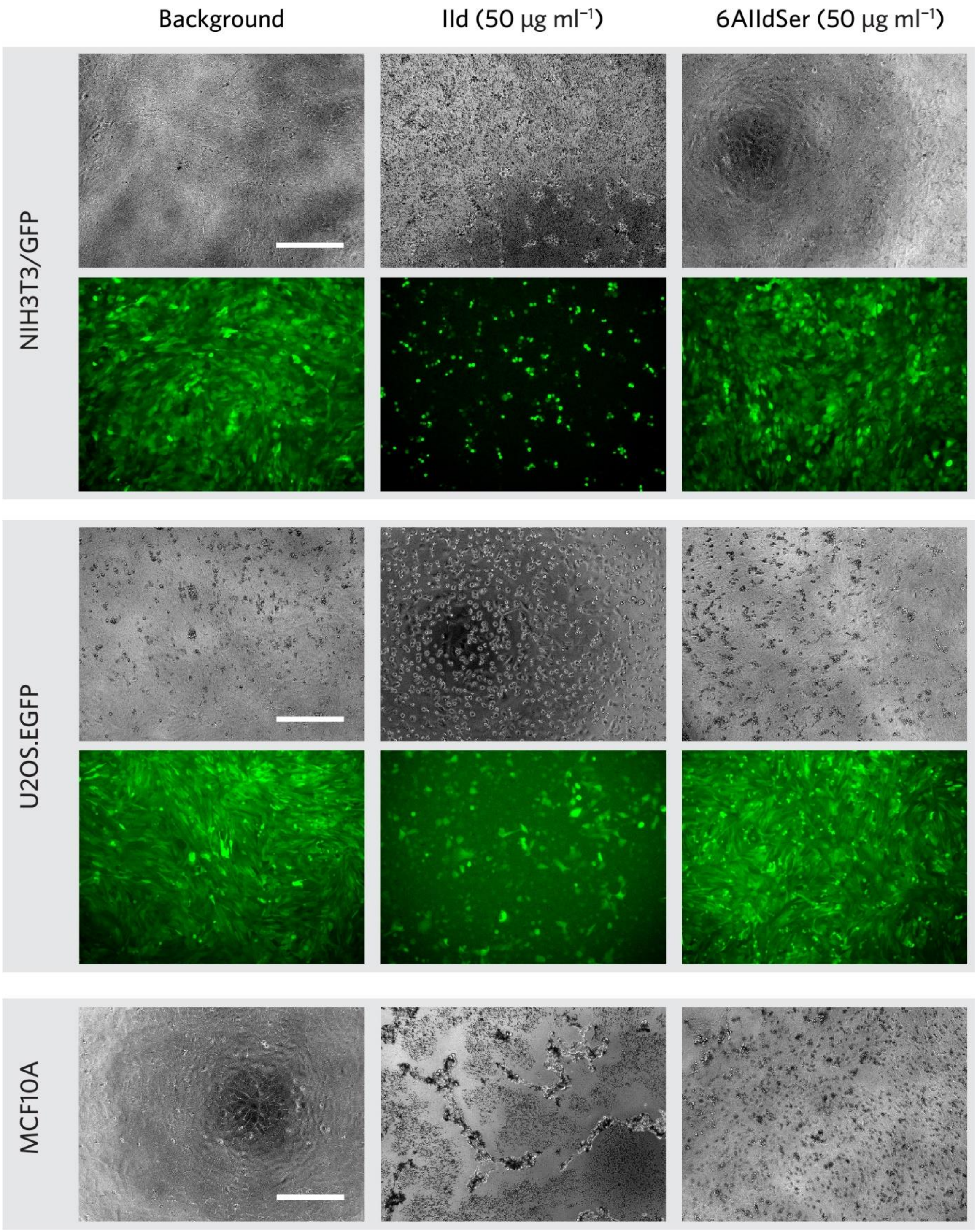

Supplementary Fig. 25 | Optical microscopic images of mammalian cells after 48 h culture with isoindigos. Different types of cells were cultured in the presence of 50 μg ml^{-1} of IId or 6AIIdSer, and cells remained much healthier under the 6AIIdSer condition. The solvent DMF (1 vol%) was used as the background reference/control. The scale bars are 300 μm. NIH3T3/GFP and U2OS.EGFP express green fluorescent protein (GFP).

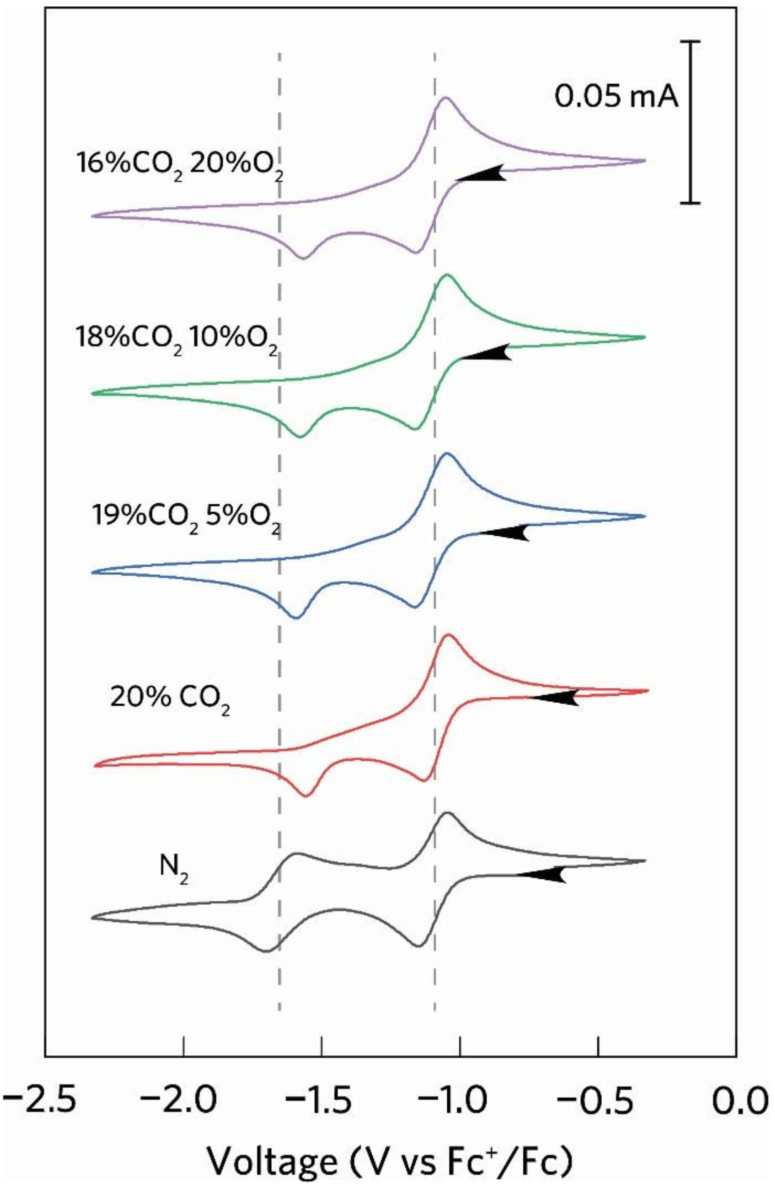

Supplementary Fig. 26 | The impact of O_2 on the redox properties and CO_2 binding behaviours of 55DBIId. The CV curves were recorded using 2.5 mM 55DBIId in DMF with 0.1 M NBu_4PF_6 under mixtures of gases at a scan rate of −50 mV s^{-1} with a cathodic sweeping direction initially at 298 K.

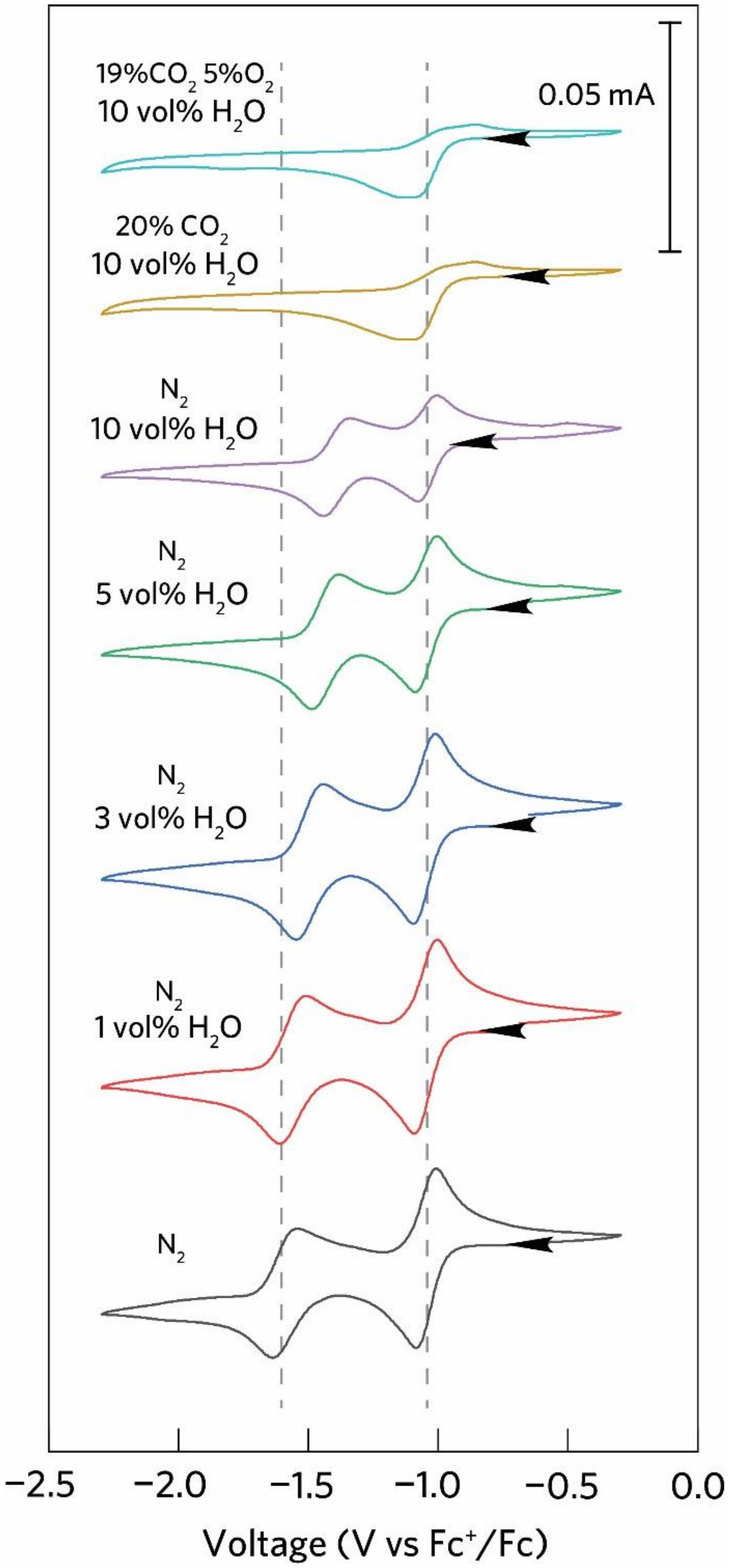

Supplementary Fig. 27 | The impact of water on the redox properties and CO_2 binding behaviours of 55DBIId. The CV was recorded using 2.5 mM 55DBIId in DMF with 0.1 M NBu_4PF_6 with various water contents under various atmospheres at a scan rate of −50 mV s^{-1} with an initial cathodic sweeping direction at 298 K.

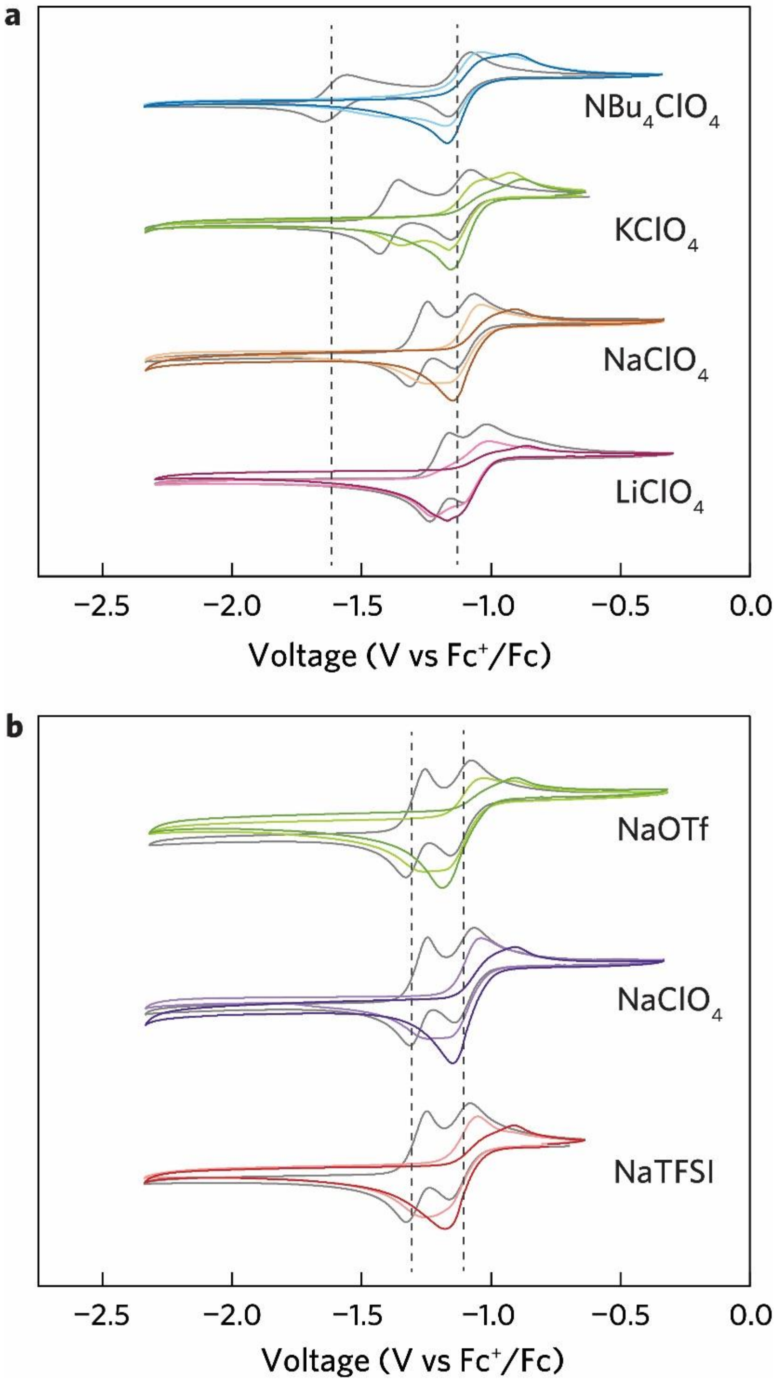

Supplementary Fig. 28 | The effect of supporting salts on the redox properties and CO_2 binding behaviours of 6MCIId. a, CV curves of 6MCIId in electrolytes with various supporting salt cations under N_2, 20% CO_2, and CO_2, respectively. **b**, CV curves of 6MCIId in electrolytes with various counter anions under N_2, 20% CO_2, and 100% CO_2. The CV was recorded using 2.5 mM 6MCIId in DMF with 0.1 M supporting salt at a scan rate of −50 mV s^{-1} with an initial cathodic sweeping direction at 298 K. (Grey: N_2; lighter colour: 20% CO_2; colour: 100% CO_2).

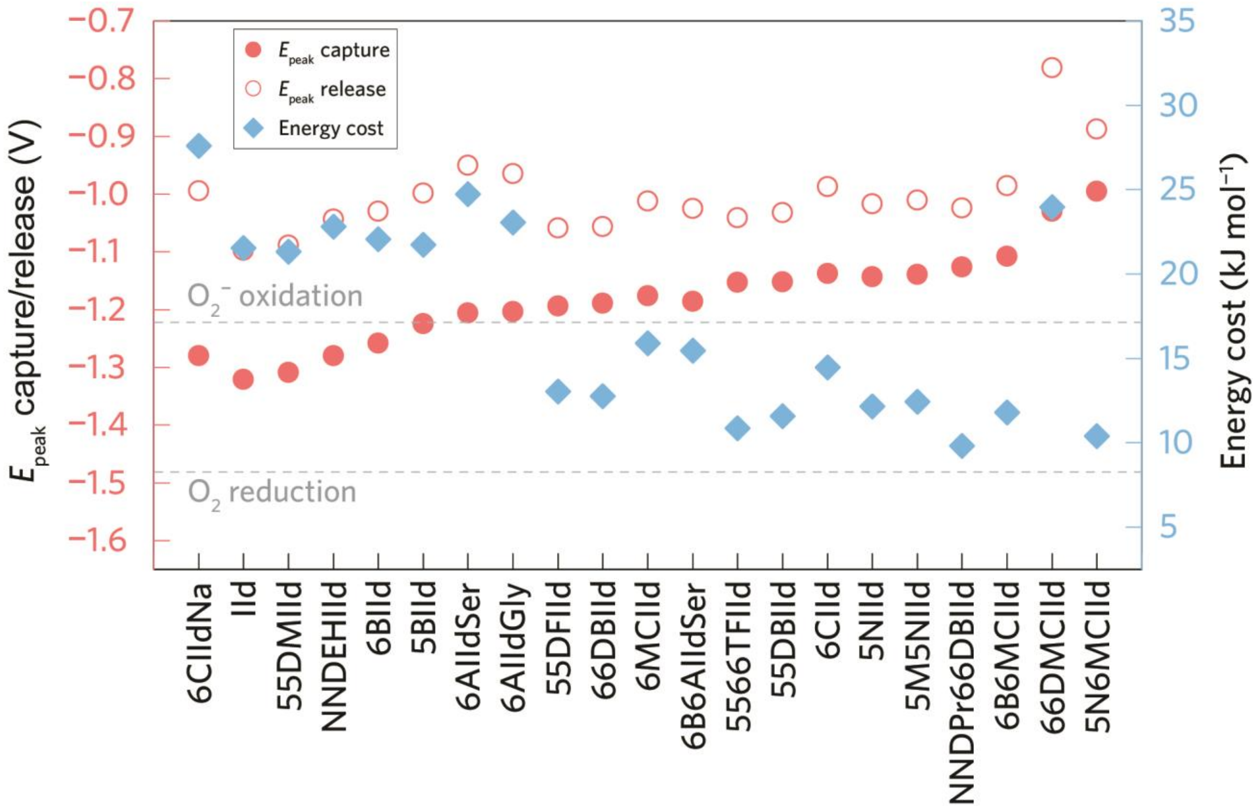

Supplementary Fig. 29 | Summary of the peak potentials for CO_2 capture (filled symbols) and release (empty symbols) of isoindigos from Supplementary Fig. 2 and 3. The theoretical energy costs for CO_2 capture and release were estimated based on the voltage gap (E_{peak} release–E_{peak} capture). The peak potentials for O_2 reduction and oxidation are shown as grey dashed lines.

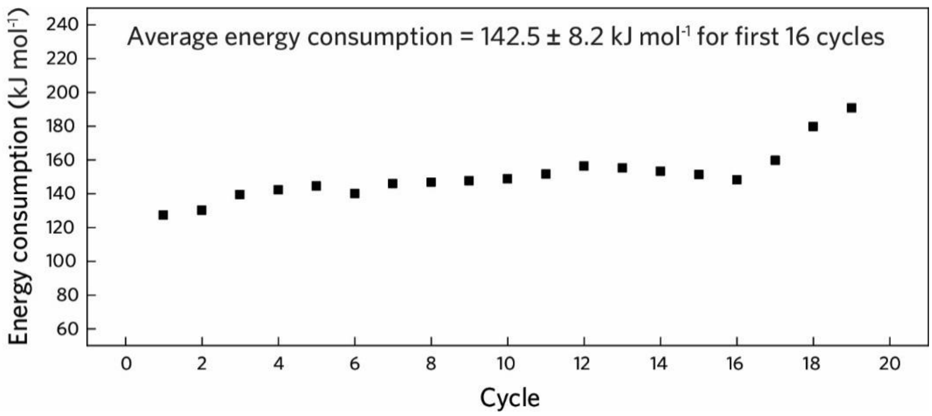

Supplementary Fig. 30 | Energy consumption of CO_2 concentration for each cycle in Fig. 6.

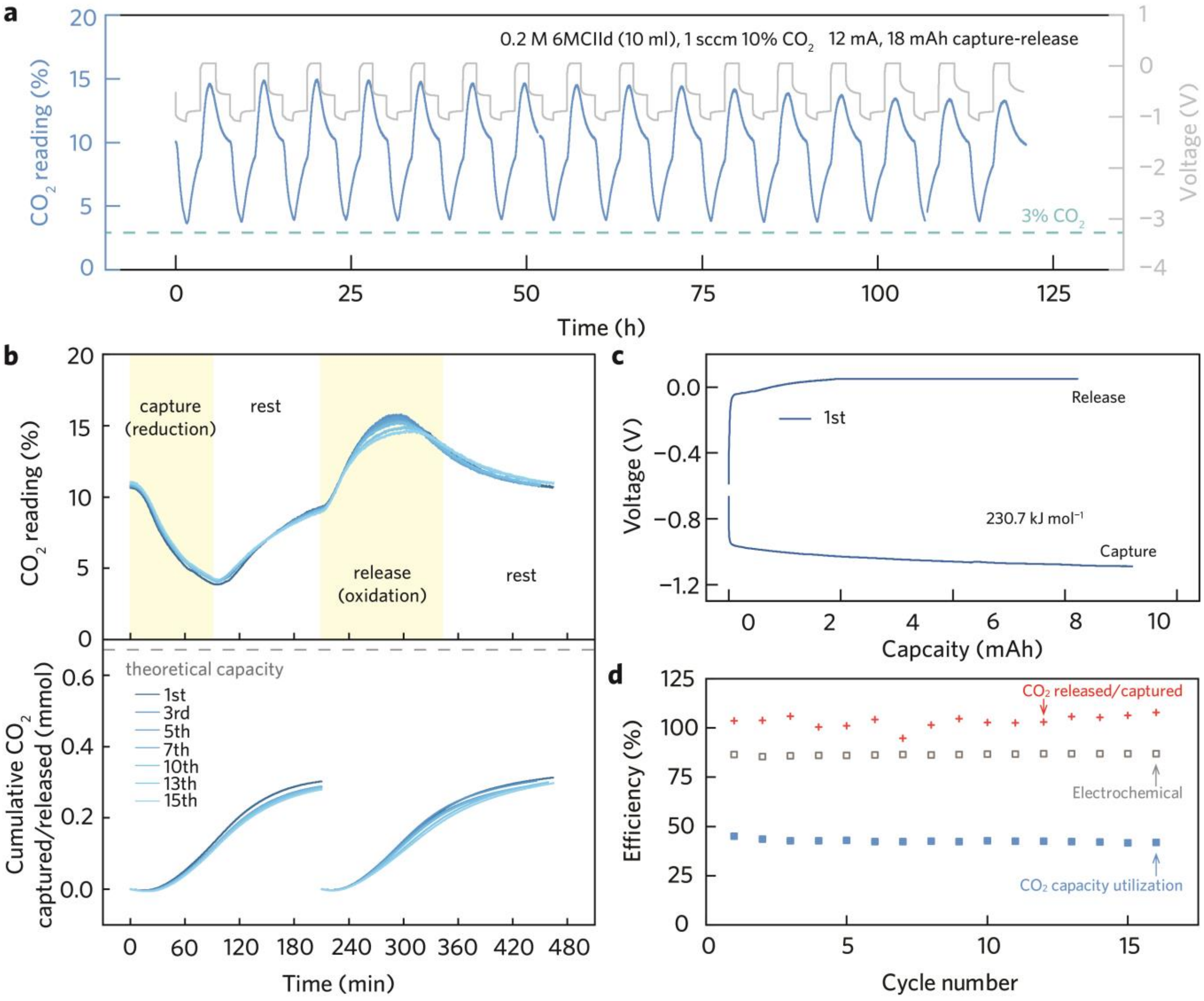

Supplementary Fig. 31 | Evaluating the performance of 6MCIId at a higher percentage of CO_2 removal. **a**, CO_2 reading at the exit of the sorbent tank and voltage curve over 16 repeating capture/release cycles for ~120 h operation. **b**, CO_2 reading for selected capture/release cycles overlaid, with the cumulative amount of CO_2 captured/released in each cycle relative to the theoretical capacity. Lighter colours represent later cycles. Capture, rest, release, and rest steps are indicated by the shaded regions. For CO_2 capture, 6MCIId was reduced at 12 mA for 90 min followed by a 120 min rest. For CO_2 release, the adducts were oxidised at 12 mA to 0.05 mV followed by a ~120 min constant voltage hold, and finally rested for another 120 min. **c**, The voltage-capacity curve for the 2nd capture/release cycle, indicating an early-stage energy consumption of 230.7 kJ per mol CO_2 concentrated. **d**, The CO_2 capacity utilisation efficiency (blue squares), release/capture efficiency (red crosses), and electrochemical efficiency (grey empty squares) of the system. The liquid sorbent was composed of 10 ml 0.2 M 6MCIId in DMF with 0.5 M NaTFSI as the supporting salt. The sorbent tank was filled with plastic beads and purged with 10% CO_2 balanced in N_2 at a flow rate of 1 sccm. On the opposite side, a Fc tank was used to balance the charge, which was filled with 20 ml 0.2 M Fc in DMF with 0.5 M NaTFSI as the supporting salt, 4 mM $FcBF_4$ to facilitate electro-reduction, and 10 mM 6MCIId to mitigate sorbent crossover.

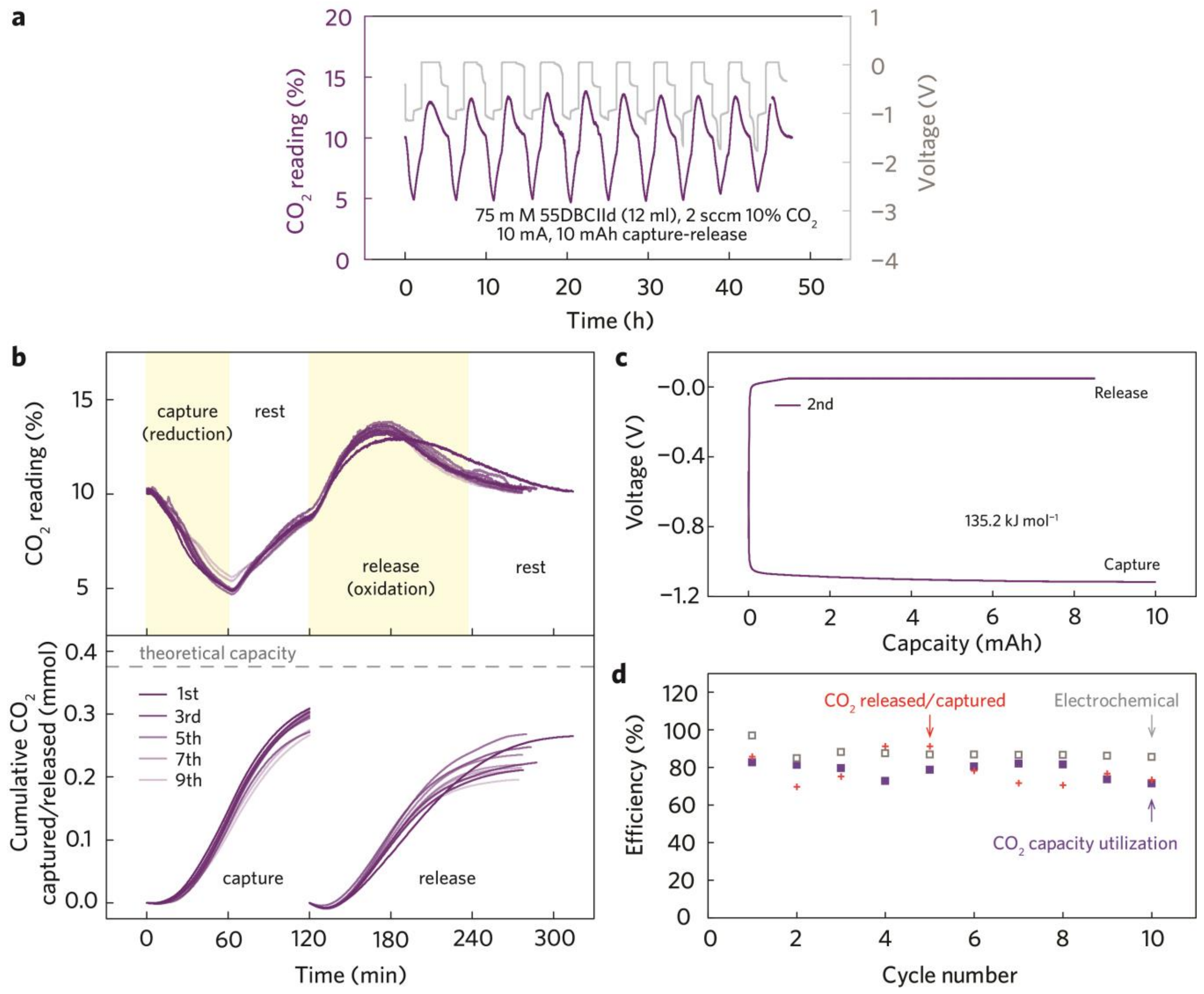

Supplementary Fig. 32 | Evaluating the performance of 55DBIId in the flow-based EMCC prototype. a, CO_2 reading at the exit of the sorbent tank and voltage curve over repeating capture/release cycles for ~50 hours of operation. **b**, CO_2 reading of selected capture/release cycles overlaid, with the cumulative amount of CO_2 captured/released in each cycle relative to the theoretical capacity. Lighter colours represent later cycles. The capture, rest, release, and rest steps are indicated by the shaded regions. For CO_2 capture, 55DBIId was reduced at 10 mA for 60 min followed by a 60 min rest. For CO_2 release, the adducts were oxidised at 10 mA to 0.05 mV followed by a ~120 min constant voltage hold, and finally rested for another 60 min. **c**, The voltage-capacity curve for the 2nd capture/release cycle, indicating an energy consumption of 135.2 kJ per mole of CO_2 concentrated. **d**, The CO_2 capacity utilisation efficiency (purple squares), release/capture efficiency (red crosses), and Coulombic efficiency (empty grey squares) of the system. The liquid sorbent was composed of 12 ml 75 mM 55DBIId slurry in DMAc with 0.25 M $NaClO_4$ as the supporting salt. On the opposite side, a Fc tank was used to balance the charge, which was filled with 30 ml 0.1 M Fc in DMAc with 0.25 M $NaClO_4$ as the supporting salt.

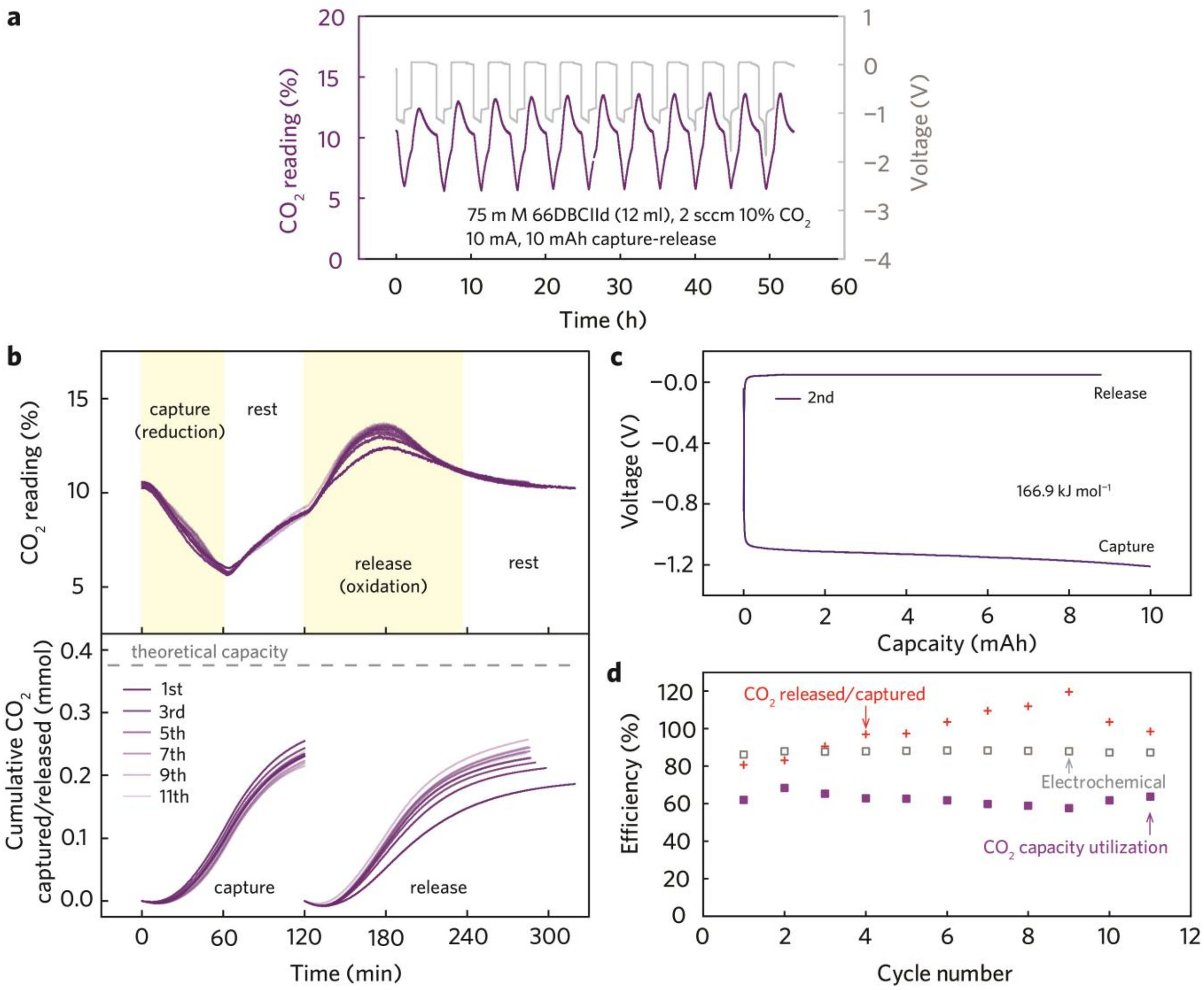

Supplementary Fig. 33 | Evaluating the performance of 66DBIId in the flow-based EMCC prototype. **a**, CO_2 reading at the exit of the sorbent tank and voltage curve over repeating capture/release cycles for ~50 h operation. **b**, The CO_2 reading for selected capture/release cycles overlaid, with the cumulative amount of CO_2 captured/released in each cycle relative to the theoretical capacity. Lighter colours represent later cycles. The capture, rest, release, and rest steps are indicated by the shaded regions. For CO_2 capture, 66DBIId was reduced at 10 mA for 60 min followed by a 60 min rest. For CO_2 release, the adducts were oxidised at 10 mA to 0.05 mV followed by a ~120 min constant voltage hold, and finally rested for another 60 min. **c**, The voltage-capacity curve for the 2nd capture/release cycle, indicating an early-stage energy consumption of 166.9 kJ per mol CO_2 concentrated. **d**, The CO_2 capacity utilisation efficiency (purple squares), release/capture efficiency (red crosses), and Coulombic efficiency (grey empty squares) of the system. The liquid sorbent was composed of 12 ml 75 mM 66DBIId slurry in DMAc with 0.25 M $NaClO_4$ as the supporting salt. On the opposite side, a Fc tank was used to balance the charge, which was filled with 30 ml 0.1 M Fc in DMAc with 0.25 M $NaClO_4$ as the supporting salt.

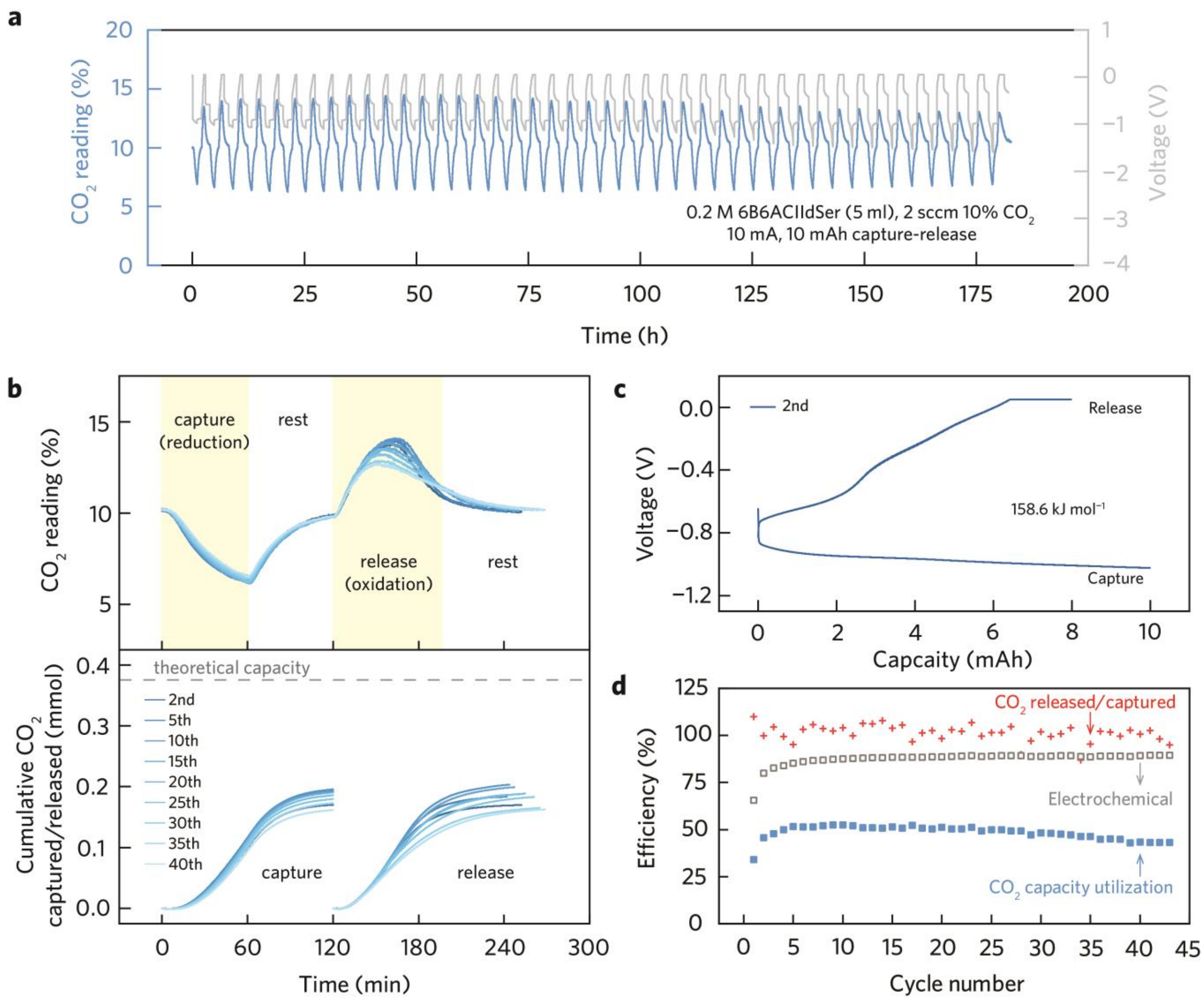

Supplementary Fig. 34 | Evaluating the performance of 6B6AIIdSer in the flow-based EMCC prototype. a, CO_2 reading at the exit of the sorbent tank and voltage curve over 43 repeating capture/release cycles for ~190 h operation. **b**, CO_2 reading for selected capture/release cycles overlaid, with the cumulative amount of CO_2 captured/released in each cycle relative to the theoretical capacity. Lighter colours represent later cycles. The capture, rest, release, and rest steps are indicated by the shaded regions. For CO_2 capture, 6B6AIIdSer was reduced at 10 mA for 60 min followed by a 60 min rest. For CO_2 release, the adducts were oxidised at 10 mA to 0.05 mV followed by a ~80 min constant voltage hold, and finally rested for another 60 min. **c**, The voltage-capacity curve for the 2nd capture/release cycle, indicating an early-stage energy consumption of 158.6 kJ per mol CO_2 concentrated. **d**, The CO_2 capacity utilisation efficiency (blue squares), release/capture efficiency (red crosses), and electrochemical efficiency (grey empty squares) of the system. The liquid sorbent was composed of 5 ml 0.2 M 6B6AIIdSer in DMF with 0.5 M NaTFSI as the supporting salt. On the opposite side, a Fc tank was used to balance the charge, which was filled with 10 ml 0.22 M Fc in DMF with 0.5 M NaTFSI as the supporting salt, and 10 mM 6B6AIIdSer to mitigate sorbent crossover.

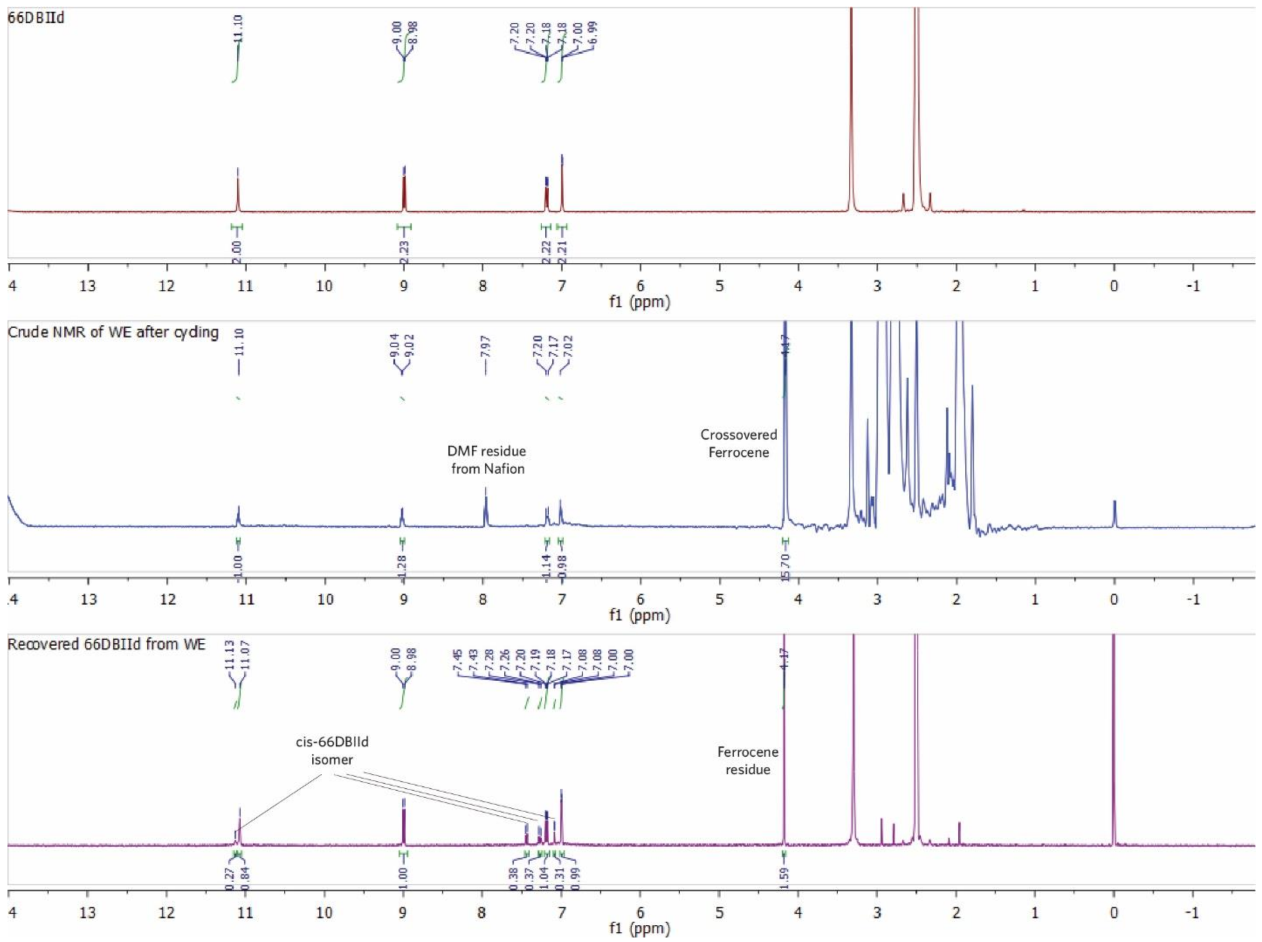

Supplementary Fig. 35 | NMR analysis of the sorbent electrolyte after CO_2 capture-release cycling of 66DBIId. The corresponding cycling data are presented in Supplementary Fig. 33. ^{1}H NMR of 66DBIId (up), crude ^{1}H NMR of sorbent electrolyte after 11 capture-release cycles (middle), and ^{1}H NMR of recovered 66DBIId from the sorbent electrolyte.

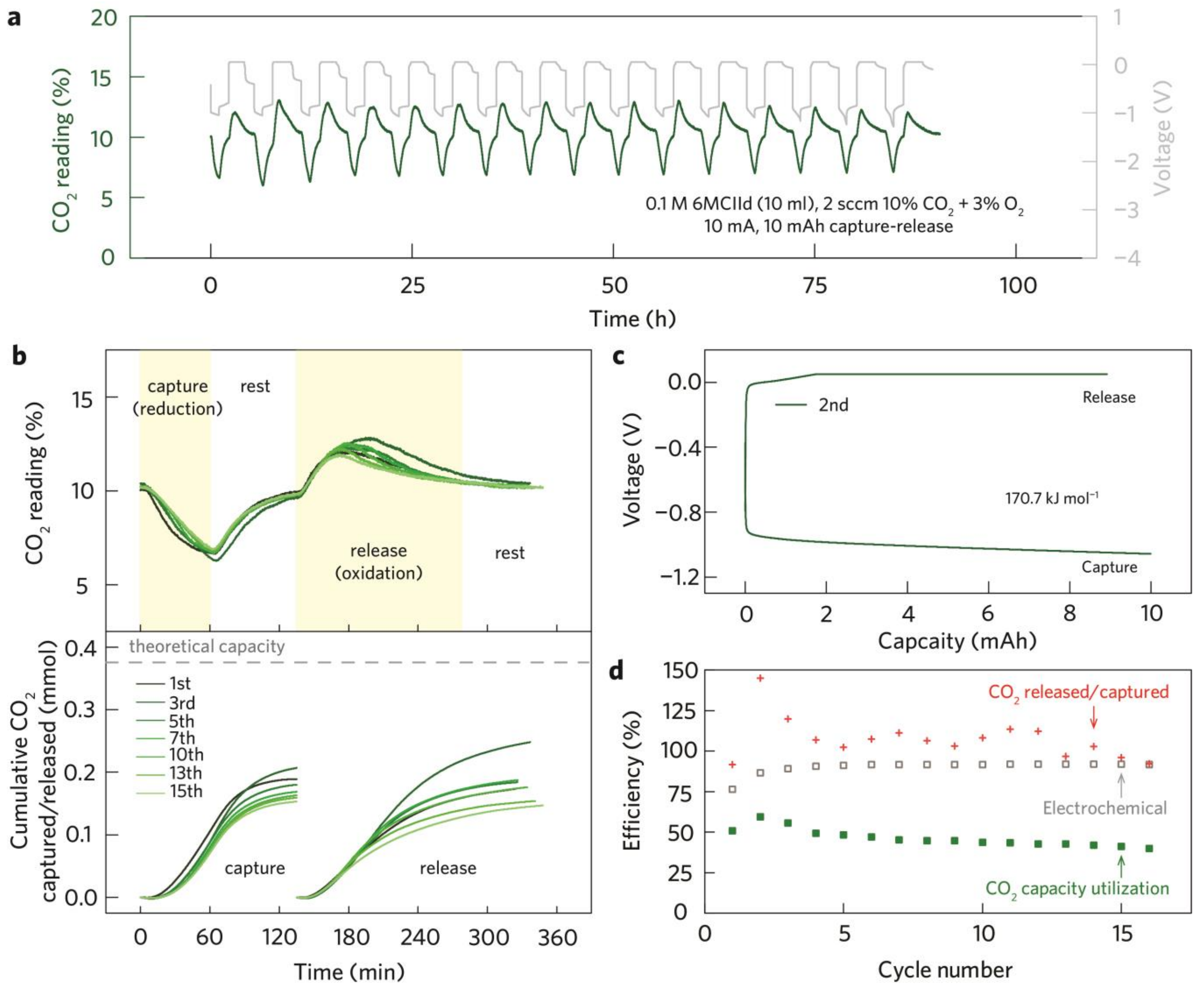

Supplementary Fig. 36 | Evaluating the performance of 6MCIId in simulated flue gas condition. **a**, CO_2 reading at the exit of the sorbent tank and voltage curve over 16 repeating capture/release cycles for ~90 h operation. **b**, CO_2 reading of selected capture/release cycles overlaid, with the cumulative amount of CO_2 captured/released in each cycle relative to the theoretical capacity. Lighter colours represent later cycles. Capture, rest, release, and rest steps are indicated by the shaded regions. For CO_2 capture, 6MCIId was reduced at 10 mA for 60 min followed by a 75 min rest. For CO_2 release, the adducts were oxidised at 10 mA to 0.05 mV followed by a ~120 min constant voltage hold, and finally rested for another 75 min. **c**, The voltage-capacity curve for the 2nd capture/release cycle, indicating an energy consumption of 170.7 kJ per mole of CO_2 concentrated. **d**, The CO_2 capacity utilisation efficiency (green squares), release/capture efficiency (red crosses), and electrochemical efficiency (empty grey squares) of the system. The liquid sorbent was composed of 10 ml 0.1 M 6MCIId in DMF with 0.5 M NaTFSI as the supporting salt. The sorbent tank was filled with plastic beads and purged with 10% CO_2 and 3% O_2 balanced in N_2 at a flow rate of 2 sccm. On the opposite side, a Fc tank was used to balance the charge, which was filled with 20 ml 0.1 M Fc in DMF with 0.5 M NaTFSI as the supporting salt, 4 mM $FcBF_4$ to facilitate Fc oxidation, and 10 mM 6MCIId to mitigate sorbent crossover.

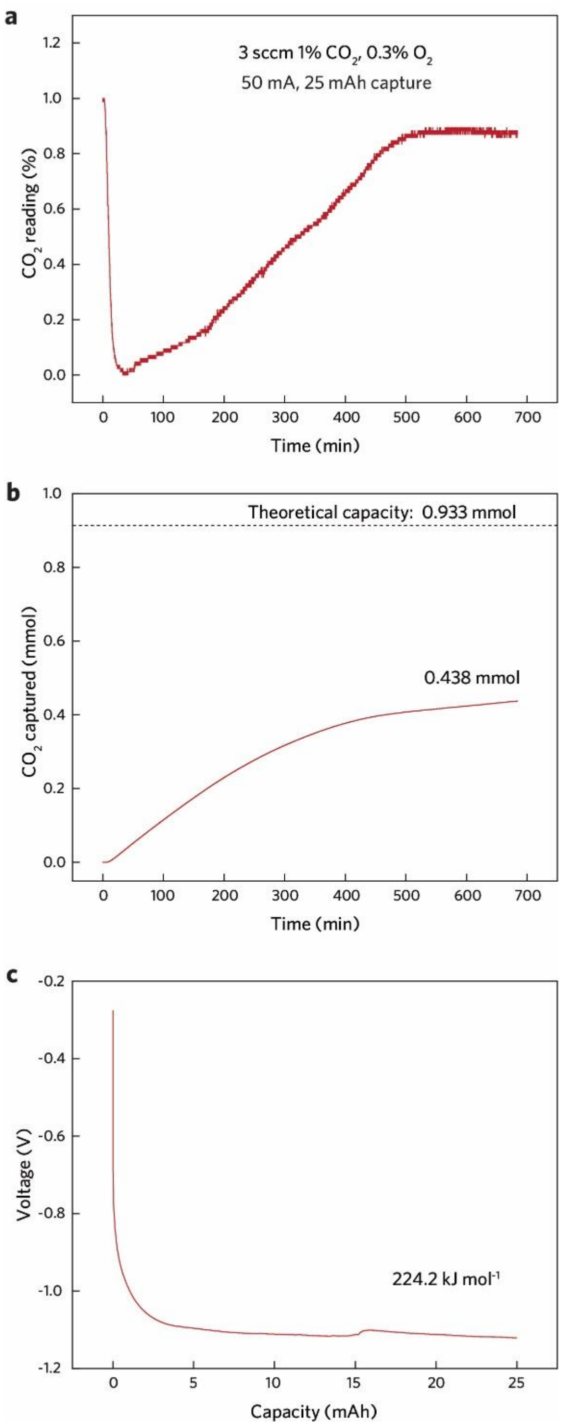

Supplementary Fig. 37 | Evaluating CO_2 capture performance of 6MCIId in flow-based EMCC prototype with low-concentration CO_2 feed. 3 sccm 1% CO_2 with 0.3% O_2 was used as the feed gas. **a**, CO_2 reading from an IR-based sensor at the exit of the sorbent tank. **b**, Cumulative amount of CO_2 captured over time. **c**, The voltage-capacity curve of the capture process, indicating an early-stage energy consumption of 224.2 kJ per mole of CO_2 captured. The area of the flow field and the carbon paper electrode was 25 cm^2. The liquid sorbent was composed of 10 ml 0.25 M 6MCIId in DMF with 0.5 M NaTFSI as the supporting salt. On the opposite side, a Fc tank was used to balance the charge, which was filled with 20 ml 0.25 M Fc in DMF with 0.5 M NaTFSI as the supporting salt and 12.5 mM 6MCIId to mitigate sorbent crossover. We observed faster redox molecule crossover in this system as the membrane area was increased from 5 to 25 cm^2, resulting in lower CO_2 capacity utilization efficiency.

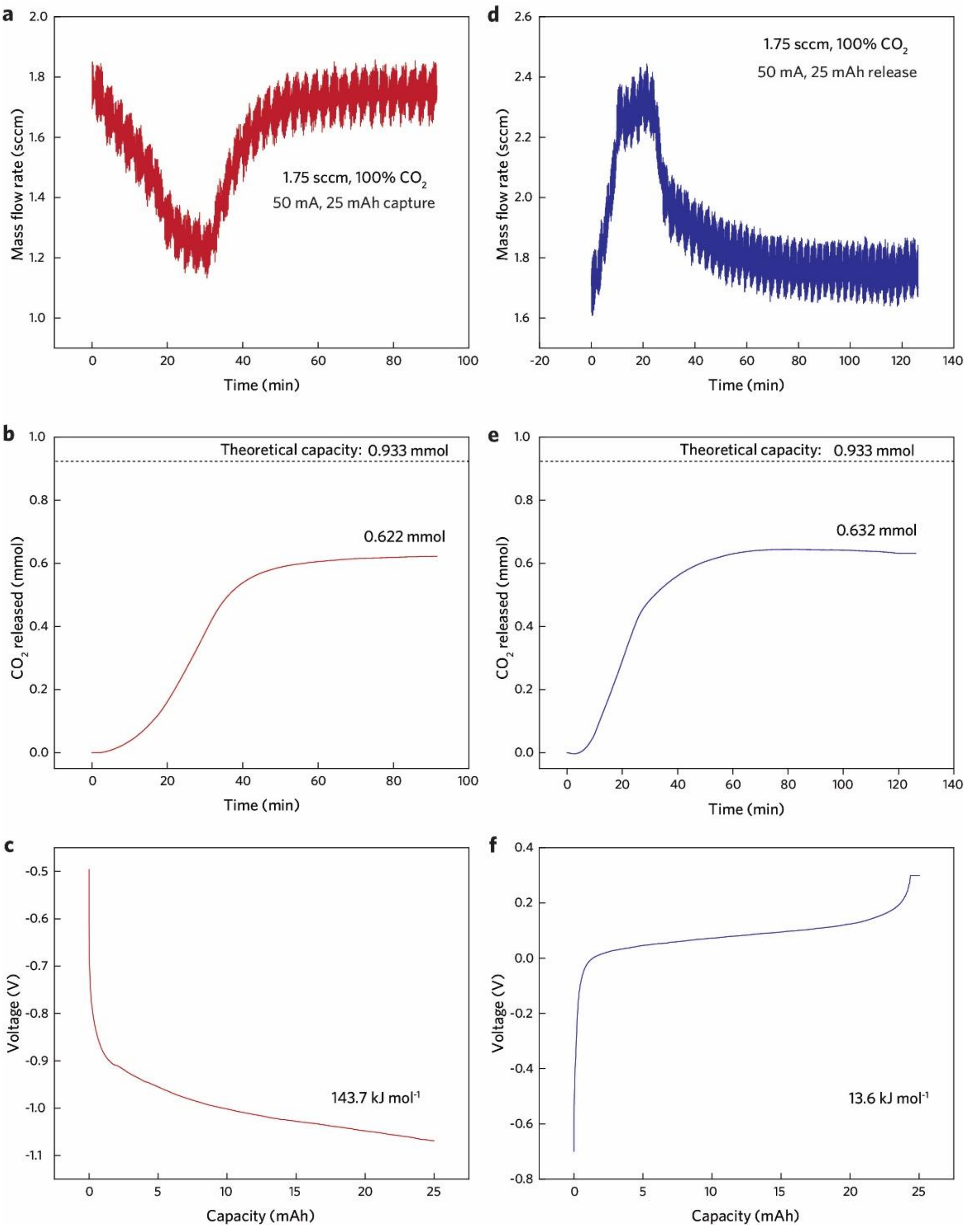

Supplementary Fig. 38 | Evaluating CO_2 capture-release performance of 6MCIId in flow-based EMCC prototype under 100% CO_2. a and **d**, CO_2 flow rate on a mass flow metre at the exit of the sorbent tank. **b** and **e**, Cumulative amount of CO_2 captured and released over time, respectively. **c** and **f**, The voltage-capacity curve of the capture and release process, indicating an early-stage energy consumption of 143.7 kJ per mole of CO_2 captured and 13.6 kJ per mole of CO_2 released, respectively. The area of the flow field and the carbon paper electrode was 25 cm^2. The liquid sorbent was composed of 10 ml 0.25 M 6MCIId in DMF with 0.5 M NaTFSI as the supporting salt. On the opposite side, a Fc tank was used to balance the charge, which was filled with 20 ml 0.25 M Fc in DMF with 0.5 M NaTFSI as the supporting salt and 12.5 mM 6MCIId to mitigate sorbent crossover.

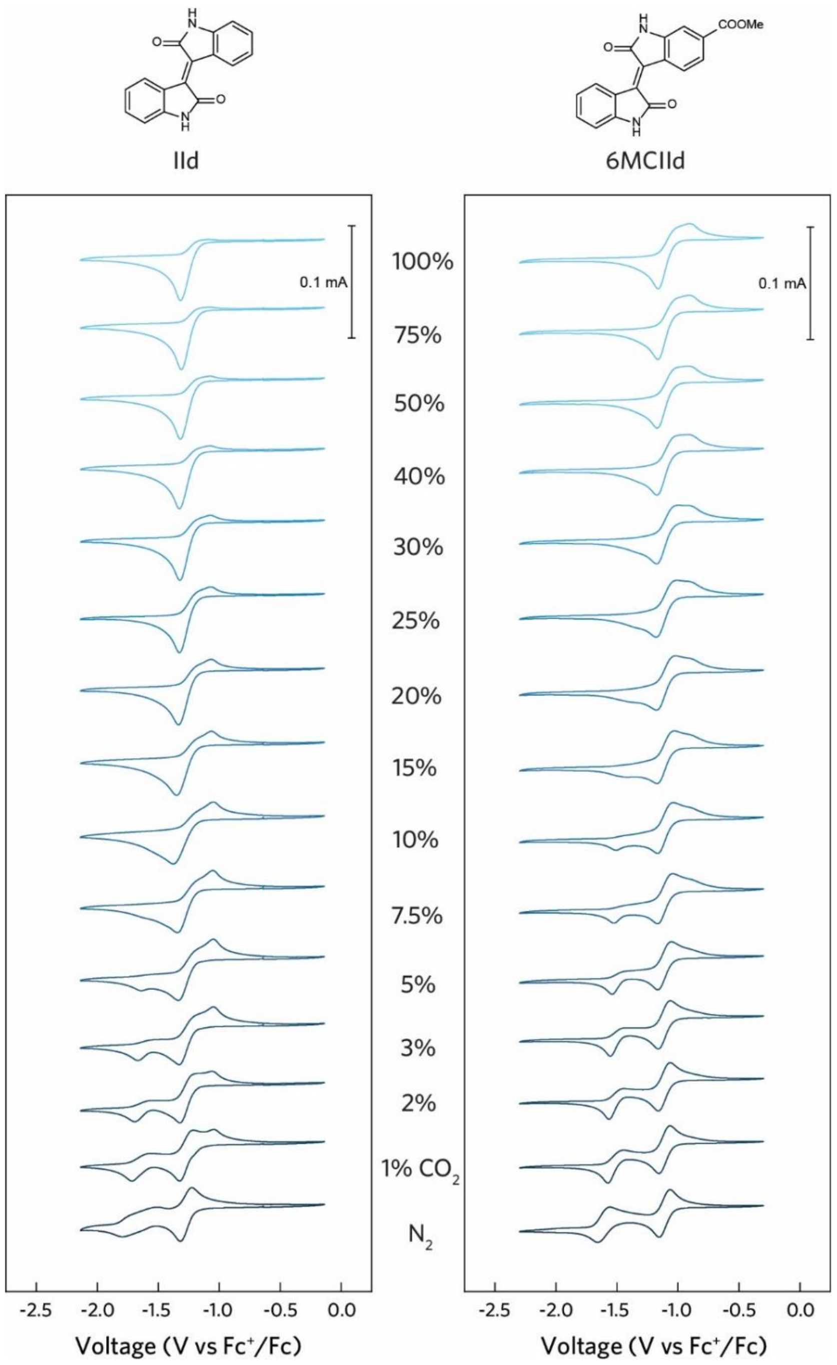

Supplementary Fig. 39 | CV of IId and 6MCIId under different concentrations of CO_2. **a**, IId. **b**, 6MCIId. The CV curves were recorded using 2.5 mM compound in anhydrous DMF with 0.1 M NBu_4PF_6 at a scan rate of −50 mV s^{-1}.

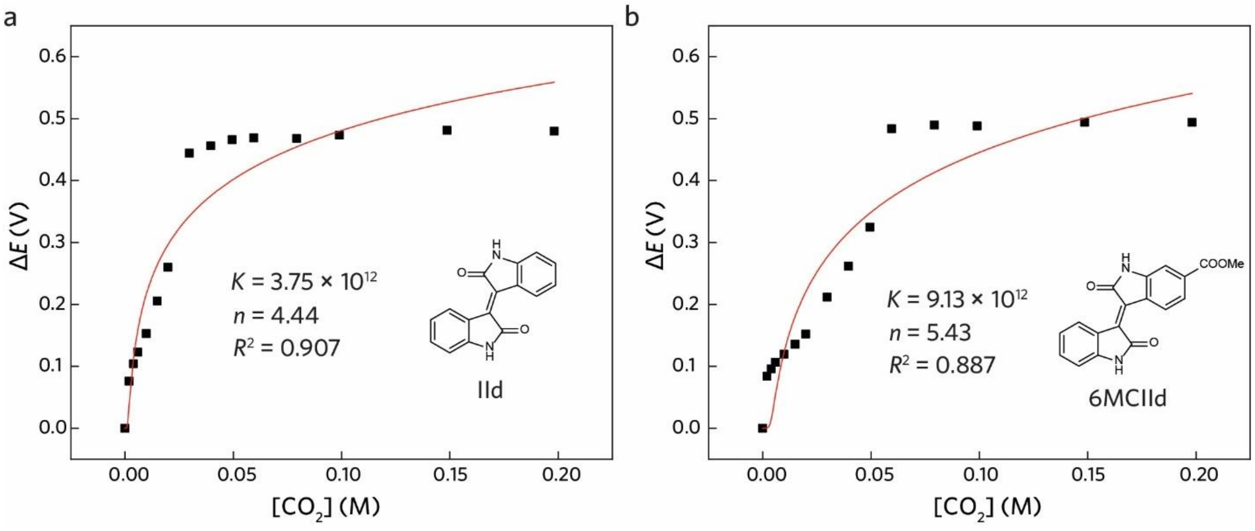

Supplementary Fig. 40 | Shifts in second reduction peak potentials ($\Delta E_{peak}(2)$) in the presence of various concentrations of CO_2 calculated using data from Response Fig. 1, and corresponding fitted values of n and CO_2 binding constant K. **a**, IId. **b**, 6MCIId.

Supplementary Tables

Supplementary Table 1 | A summary of existing redox-active quinone sorbents with their redox potentials (V vs. Fc^+/Fc) and CO_2 binding constants in DMF[1].

**Quinoids**		$E_{1/2}$ **(1)**[A] **in** CO_2 **(V)**	$E_{1/2}$ **(2)**[B] **in** N_2 **(V)**	$E_{1/2}$ **(2) in** CO_2 **(V)**	$\log K_{CO_2}$
AQ-O-C3H7	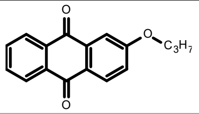	−1.55	−2.2	−1.55	11.70
AQ	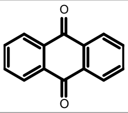	−1.4	−2.15	−1.4	13.39
AQ-Cl	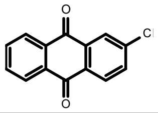	−1.25	−2	−1.88	2.73
AQ-COO-C3H7	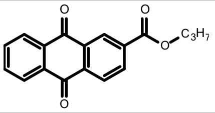	−1.29	−2	−1.89	2.56
BQ	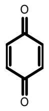	−0.95	−1.98	−0.95	18.12
DBQ	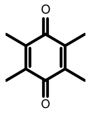	−1.39	−2.39	−1.39	17.61
TBQ	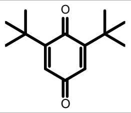	−1.25	−2.29	−1.25	18.29
BQ-Cl2	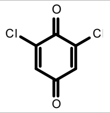	−0.7	−1.66	−1.59	1.86
BQ-Cl4	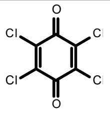	−0.48	−1.42	−1.38	1.28
p-NQ	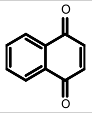	−1.23	−2.06	−1.23	14.74
p-NQ-Cl2	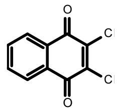	−0.9	−1.75	−1.63	2.73

[A] The first half-wave potential. [B] The second half-wave potential cathodic to the first one.

Supplementary Table 2 | Summary of key parameters of isoindigo sorbents including their redox properties, ^{1}H NMR, and optical band gap.

	**Substituents**	**$E_{1/2}$ in N_2 (V)[A]**	**$E_{1/2}$ in CO_2 (V)[A]**	**$\Delta E_{peak}(2)$[B] (V)**	**K_{CO_2} (M^{-1})**	**log K_{CO_2}**	**^{1}H NMR (H^a, $H^{a'}$) (ppm)**	**^{1}H NMR (H^b, $H^{b'}$) (ppm)**	**Optical band gap (eV)**
6CIIdNa	6-COONa	-1.33, -1.49	-1.14	0.301	6.11E+05	5.79	9.05, 8.93	10.84	2.03
IId	-	-1.29, -1.79	-1.21	0.511	2.19E+09	9.34	9.06	10.89	2.04
55DMIId	5,5'-OMe	-1.29, -1.76	-1.2	0.494	1.13E+09	9.05	8.85	10.69	1.78
NNDEHIId	*N*,*N*'-2-ethylhexyl	-1.23, -1.80	-1.16, -1.45	0.350	4.19E+06	6.62	9.1	N.A.	2.00
6BIId	6-Br	-1.21, -1.75	-1.14	0.538	6.25E+09	9.8	9.04, 9.00	10.95, 11.06	2.05
5BIId	5-Br	-1.19, -1.67	-1.11	0.494	1.12E+09	9.05	9.07, 9.31	10.96, 11.05	2.04
6AIIdSer	6-methyl serinate	-1.18, -1.60	-1.08	0.46	2.57E+08	8.41	9.07, 9.11	10.94, 11.12	2.04
6AIIdGly	6-methyl glycinate	-1.17, -1.62	-1.08	0.469	4.30E+08	8.63	9.08, 9.12	10.94, 11.12	2.04
55DFIId	5,5'-F	-1.14, -1.69	-1.13	0.547	9.13E+09	9.96	9.00	11.02	1.97
66DBIId	6,6'-Br	-1.14, -1.67	-1.12	0.525	3.88E+04	9.59	8.99	11.1	2.06
6MCIId	6-COOMe	-1.12, -1.62	-1.09	0.484	7.78E+08	8.89	9.07, 9.15	10.94, 11.08	2.03
6B6AIIdSer	6-Br-6'-methyl serinate	-1.11, -1.55	-1.11	0.475	5.41E+08	8.73	9.02, 9.11	11.09, 11.16	2.03
5566TFIId	5,5',6,6'-F	-1.10, -1.68	-1.1	0.570	2.17E+10	10.3	9.21	11.2	2.02
55DBIId	5,5-Br	-1.09, -1.61	-1.09	0.510	2.16E+09	9.33	9.32	11.11	1.99
6CIId	6-COOH	-1.09, -1.35	-1.06, -1.18	0.129	7.50E+02	2.87	9.07, 9.13	10.93, 11.05	2.01
5NIId	5-NO_2	-1.08, -1.57	-1.08	0.484	7.63E+08	8.88	9.08, 10.12	11.03, 11.63	2.07
5M5NIId	5-OMe-5'-NO_2	-1.08, -1.55	-1.08	0.470	4.45E+08	8.65	8.83, 10.14	10.84, 11.61	1.71
NNDPr66DBIId	*N*,*N*'-Pr-6,6'-Br	-1.07, -1.61	-1.08, -1.34	0.247	7.48E+04	4.48	9.05	N.A.	2.02
6B6MCIId	6-Br-6-COOMe	-1.05, -1.55	-1.05	0.486	8.34E+08	8.92	9.02, 9.14	11.09, 11.12	2.02
66DMCIId	6,6'-COOMe	-0.97, -1.41	-0.91	0.435	1.17E+08	8.07	9.17	11.18	2.02
5N6MCIId	5-NO_2-6-COOMe	-0.93, -1.40	-0.94, -1.15	0.232	4.15E+04	4.62	9.15, 10.10	11.22, 11.68	2.08

[A]Fc^+/Fc is used as the internal reference. [B]$\Delta E_{peak}(2)$ was calculated based on the difference between the second reduction peak potentials ($IId^{\bullet-}$/IId^{2-}) in CO_2 and N_2.

Supplementary Table 3 | Reaction rate constant for the first CO_2 binding using different isoindigo sorbents. Isoindigo (5 mM) in DMF using 0.1 M NBu_4PF_6 as the supporting salt.

**Isoindigo sorbent**	$k_{bimolecular}$ **(M^{-1} s^{-1})**	k_f **(s^{-1}) (20% CO_2)**
6MCIId	22.45	0.889
6AIIdGly	19.85	0.786
66DBIID	18.64	0.738
NNDPr66DBIId	3.31	0.131

Supplementary Table 4 | Comparison between the DFT-simulated 1st electron-transfer redox potentials (V vs. Fc^+/Fc) and experimental 1st half-wave potentials in N_2 (V vs. Fc^+/Fc) of various isoindigos.

**Isoindigo sorbent**	**Theoretical (V)**	**Experimental (V)**
IId	−1.102	−1.29
5BIId	−1.100	−1.19
66DBIId	−1.058	−1.14
55DBIId	−1.006	−1.09

Supplementary Table 5 | DFT-calculated CO_2 binding energies of $IId^{\bullet-}$ (E_b1) and $[IId\text{-}CO_2]^{\bullet-}$ (E_b2).

	E_b1 (eV)	E_b2 (eV)	Theoretical $\log K_{CO_2}$[A]	Experimental $\log K_{CO_2}$
IId	0.08	0.50	9.18	9.34
55DMIId	0.08	0.49	9.03	9.05
55DBIId	0.20	0.43	9.98	9.33
5BIId	0.23	0.50	11.56	9.05
66DBIId	0.20	0.45	10.29	9.59
NNDPr66DBIId	0.07	0.35	6.65	4.48

[A]Theoretical $\log K_{CO_2}$ was calculated using the equation, $\log K_{CO_2} = \frac{E_b1+E_b2}{RT\ln 10}$, where R is the ideal gas constant and T is the room temperature of 298 K.

Supplementary Table 6 | Solubilities of various isoindigos in DMF.

Isoindigo sorbent	Solubility in DMF (mM)
IId	230
55DBIId	6.3
66DMCIId	<2.5
6MCIId	515
6AIIdGly	606
6AIIdSer	830
6B6AIIdSer	568

Supplementary Methods

Materials and Methods

Unless otherwise stated, all the chemicals were purchased from commercial sources and used without further purification.

Nuclear Magnetic Resonance Spectroscopy (NMR)

NMR spectra were collected on a Bruker AMX400 (400 MHz) spectrometer. Chemical shifts were reported in parts per million (ppm). Residual solvent peak was used as an internal reference.

Low-Resolution Electrospray Ionisation Mass Spectroscopy (Low-Res ESI-MS)

Low-res ESI-MS was recorded on a Thermo Scientific LCQ Fleet Ion trap Mass spectrometer.

High-Resolution ESI Quadrupole Time of Flight (Q-TOF) MS

Analysis was performed using the Bruker compact Q-TOF-MS in negative ion mode using the ESI ionization source. Tuning and mass calibration was performed prior to running client samples, using 10 mM sodium formate. Mass spectra for the sample were collected from m/z 50-3000, at a rate of one spectrum per sec over a span of 30 seconds.

Ultraviolet-visible (UV-vis) Spectroscopy

UV-vis absorption spectra were recorded on a Thermo Scientific Genesys 10S UV-vis spectrophotometer.

Synthesis of isoindigos

General synthetic procedure for isoindigos:

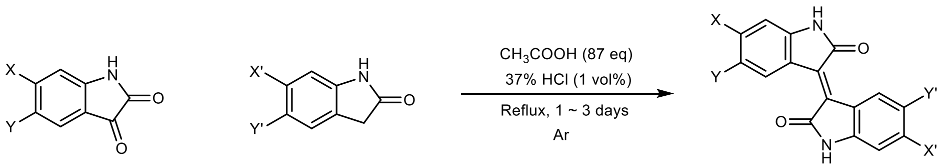

Supplementary Fig. 41 | Synthesis of isoindigos.

To a mixture of isatin (1.106 ~20 mmol, 1.0 equiv) and 2-oxindole (1.106~20 mmol, 1.0 equiv) in acetic acid (~87 equiv) was added 37% HCl solution (1 vol% of acetic acid). The suspension was heated at reflux for 1 to 3 days under Ar atmosphere before being cooled to room temperature (most reactions were completed in 1 day except for those substrates with strong electron-withdrawing groups which required further reaction time). The mixture was filtered and washed with copious water, ethanol, and ethyl acetate (EA). The product was dried in the vacuum oven at 60 °C for 15 h to afford the desired isoindigos in 50~97% yield.

5,5'-Dimethoxylisoindigo (55DMIId):

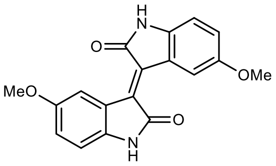

Supplementary Fig. 42 | Chemical structure of 55DMIId.

The target was synthesised by the general procedure starting with 5-methoxyisatin (209 mg, 1.18 mmol) and 5-methoxy-2-oxindole (192.6 mg, 1.18 mmol) in a day to afford the desired product (324 mg, 85%). ^{1}H NMR (400 MHz, DMSO) δ 10.69 (s, 2H), 8.85 (d, *J* = 2.6 Hz, 2H), 6.97 (dd, *J* = 8.5, 2.7 Hz, 2H), 6.75 (d, *J* = 8.5 Hz, 2H), 3.73 (s, 6H).

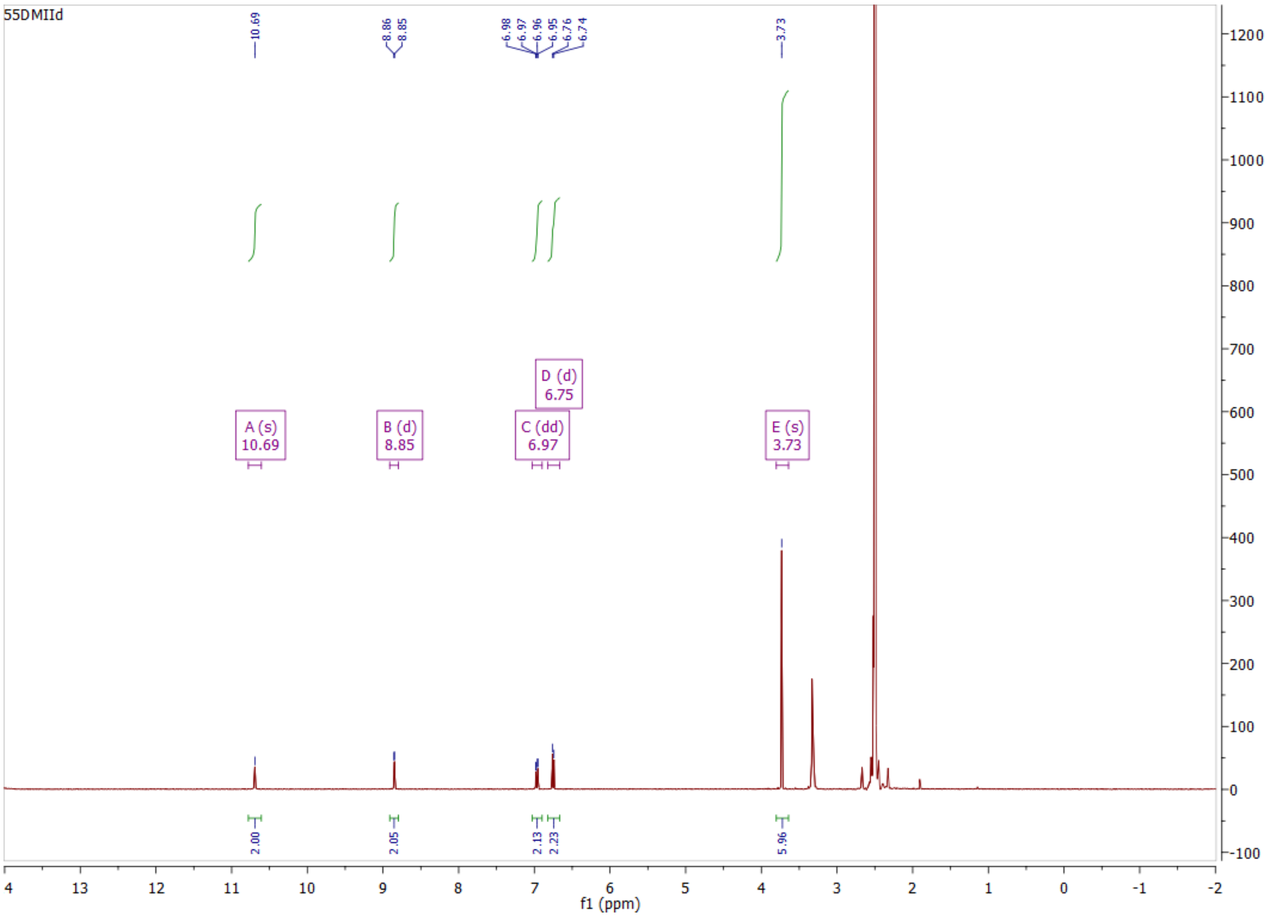

Supplementary Fig. 43 | ^{1}H NMR spectra of 55DMIId.

Isoindigo (IId):

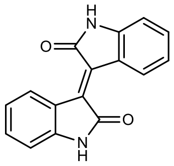

Supplementary Fig. 44 | Chemical structure of IId.

The target was synthesised by the general procedure starting with isatin (2.94 g, 20 mmol) and 2-oxindole (2.66 g, 20 mmol) in a day to afford the desired product (5.01 g, 96%). ^{1}H NMR (400 MHz, DMSO) δ 10.89 (s, 2H), 9.06 (d, *J* = 7.9 Hz, 2H), 7.34 (t, *J* = 7.6 Hz, 2H), 6.96 (t, *J* = 7.8 Hz, 2H), 6.84 (d, *J* = 7.7 Hz, 2H). ^{13}C NMR (101 MHz, DMSO) δ 168.92, 144.03, 133.27, 132.54, 129.25, 121.65, 121.07, 109.46.

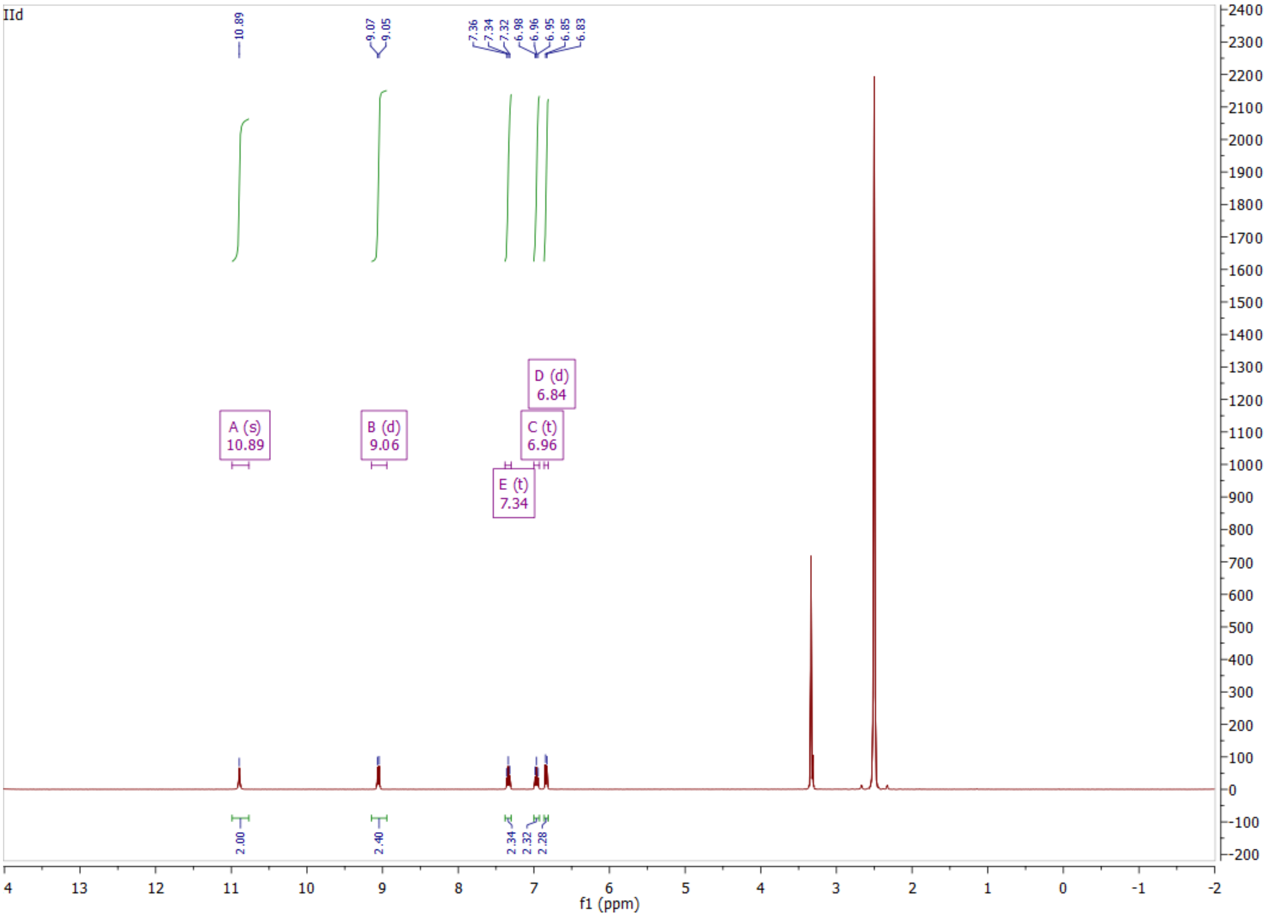

Supplementary Fig. 45 | ^{1}H NMR spectra of IId.

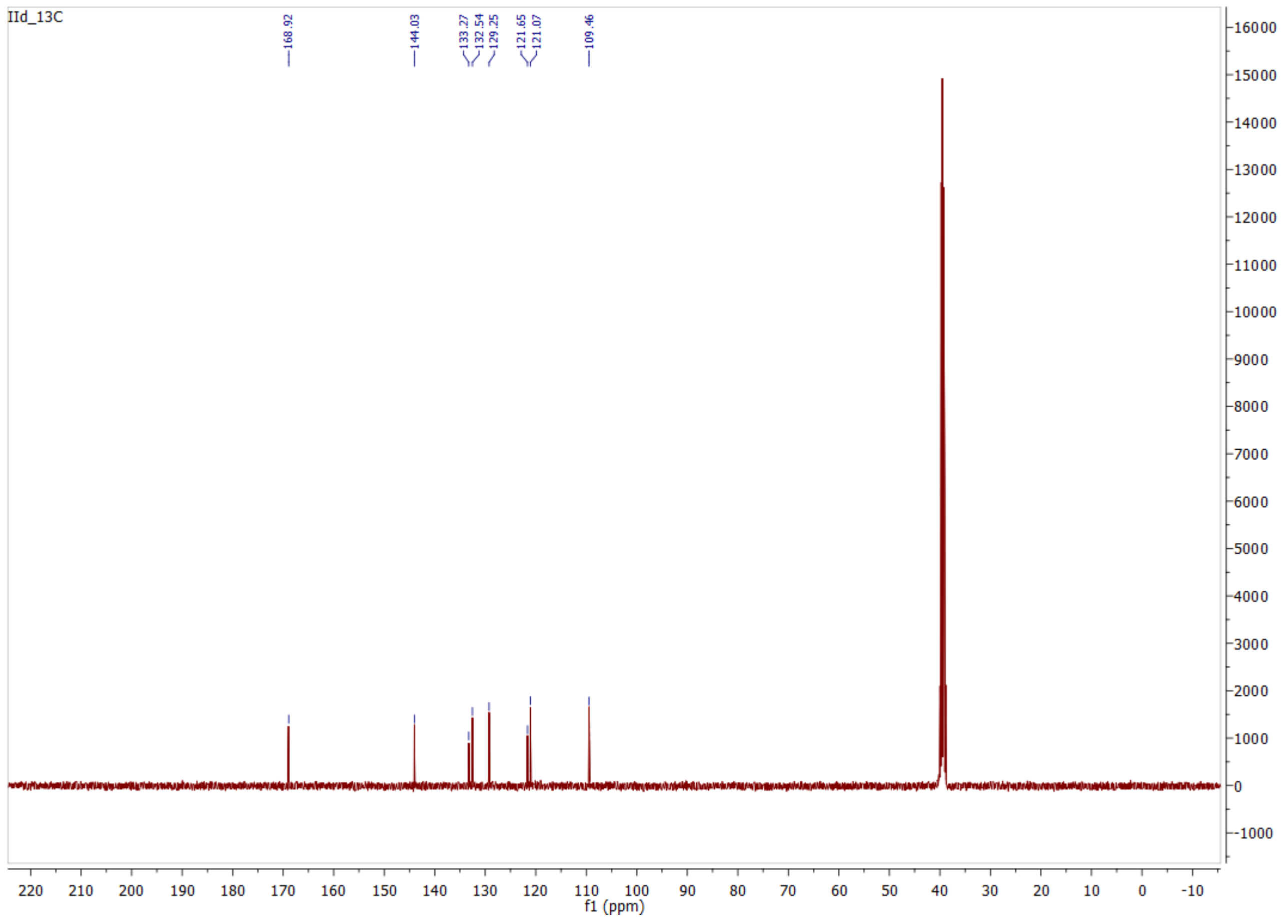

Supplementary Fig. 46 | ^{13}C NMR spectra of IId.

5,5'-Difluoroisoindigo (55DFIId):

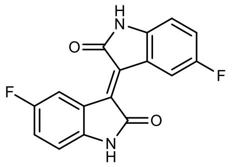

Supplementary Fig. 47 | Chemical structure of 55DFIId.

The target was synthesised by the general procedure starting with 5-fluoroisatin (195 mg, 1.18 mmol) and 5-fluoro-2-oxindole (178.3 mg, 1.18 mmol) in a day to afford the desired product (177 mg, 50%). ^{1}H NMR (400 MHz, DMSO) δ 11.02 (s, 2H), 9.00 (dd, *J* = 11.4, 2.7 Hz, 2H), 7.25 (td, *J* = 8.7, 2.8 Hz, 2H), 6.85 (dd, *J* = 8.6, 4.9 Hz, 2H).

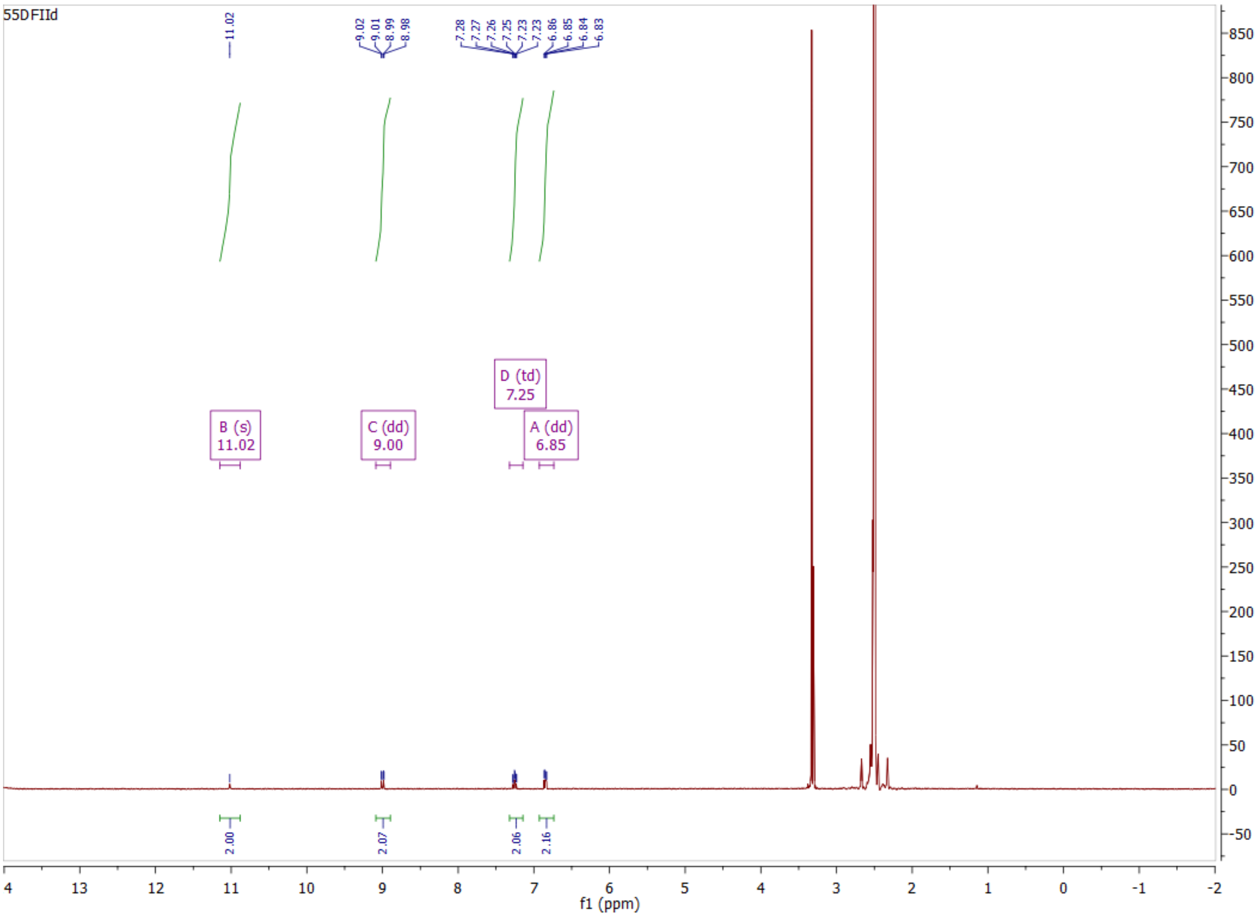

Supplementary Fig. 48 | ^{1}H NMR spectra of 55DFIId.

5-Bromoisoindigo (5BIId):

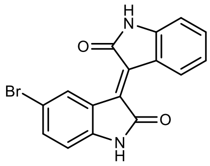

Supplementary Fig. 49 | Chemical structure of 5BIId.

The target was synthesised by the general procedure starting with isatin (323.7 mg, 2.2 mmol) and 5-bromo-2-oxindole (466.5 mg, 2.2 mmol) in a day to afford the desired product (691 mg, 92%). ^{1}H NMR (400 MHz, DMSO) δ 11.05 (s, 1H), 10.96 (s, 1H), 9.31 (d, *J* = 1.8 Hz, 1H), 9.07 (d, *J* = 8.0 Hz, 1H), 7.52 (dd, *J* = 8.3, 2.0 Hz, 1H), 7.37 (t, *J* = 7.6 Hz, 1H), 6.98 (t, *J* = 7.8 Hz, 1H), 6.83 (dd, *J* = 16.1, 8.0 Hz, 2H).

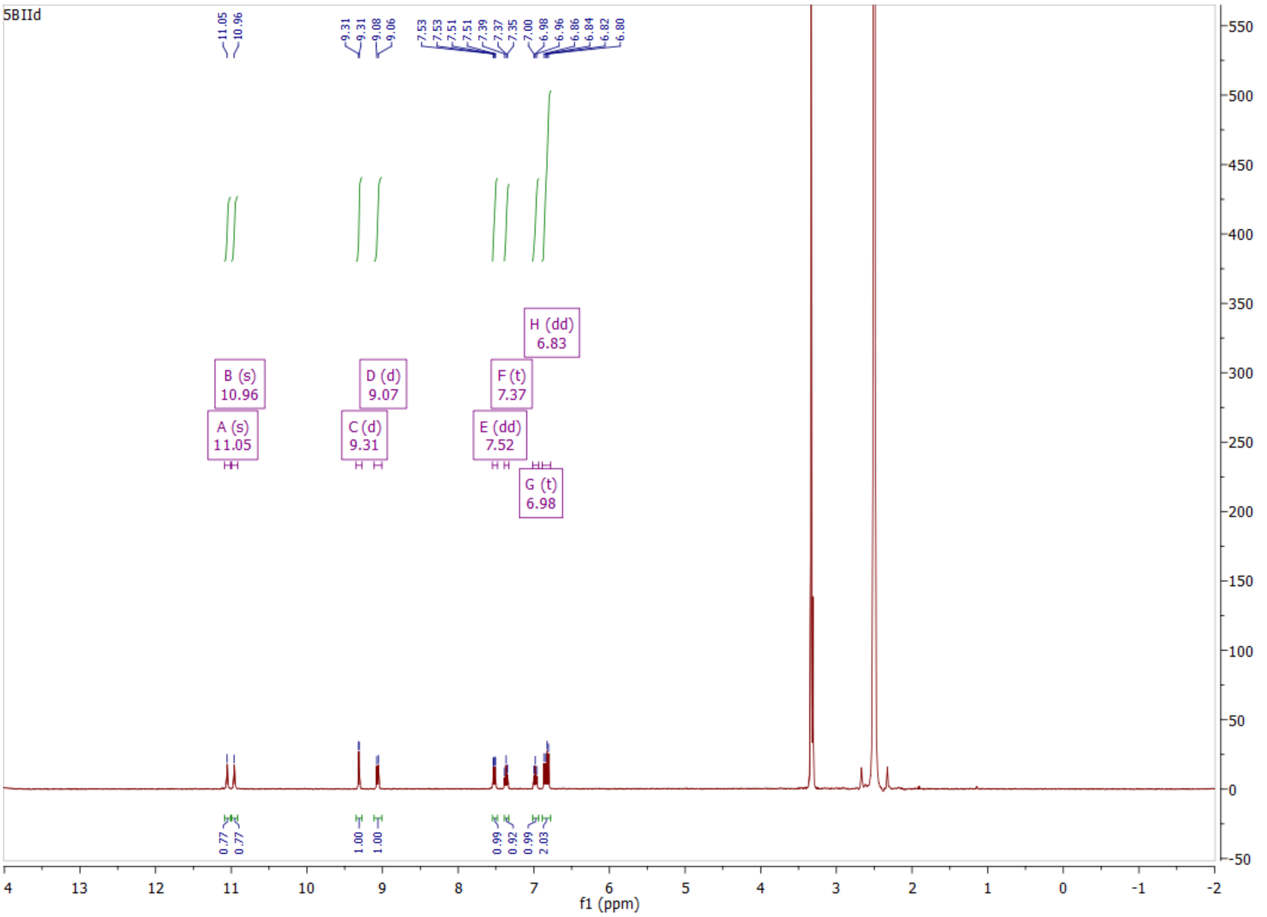

Supplementary Fig. 50 | ^{1}H NMR spectra of 5BIId.

6-Bromoisoindigo (6BIId):

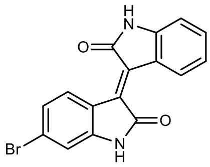

Supplementary Fig. 51 | Chemical structure of 6BIId.

The target was synthesised by the general procedure starting with 6-bromoisatin (250 mg, 1.106 mmol) and 2-oxindole (147 mg, 1.106 mmol) in a day to afford the desired product (261 mg, 69%). ^{1}H NMR (400 MHz, DMSO) δ 11.06 (s, 1H), 10.95 (s, 1H), 9.04 (d, *J* = 7.2 Hz, 1H), 9.00 (d, *J* = 8.9 Hz, 1H), 7.36 (s, 1H), 7.18 (d, *J* = 7.1 Hz, 1H), 6.98 (d, *J* = 8.6 Hz, 2H), 6.84 (d, *J* = 7.5 Hz, 1H).

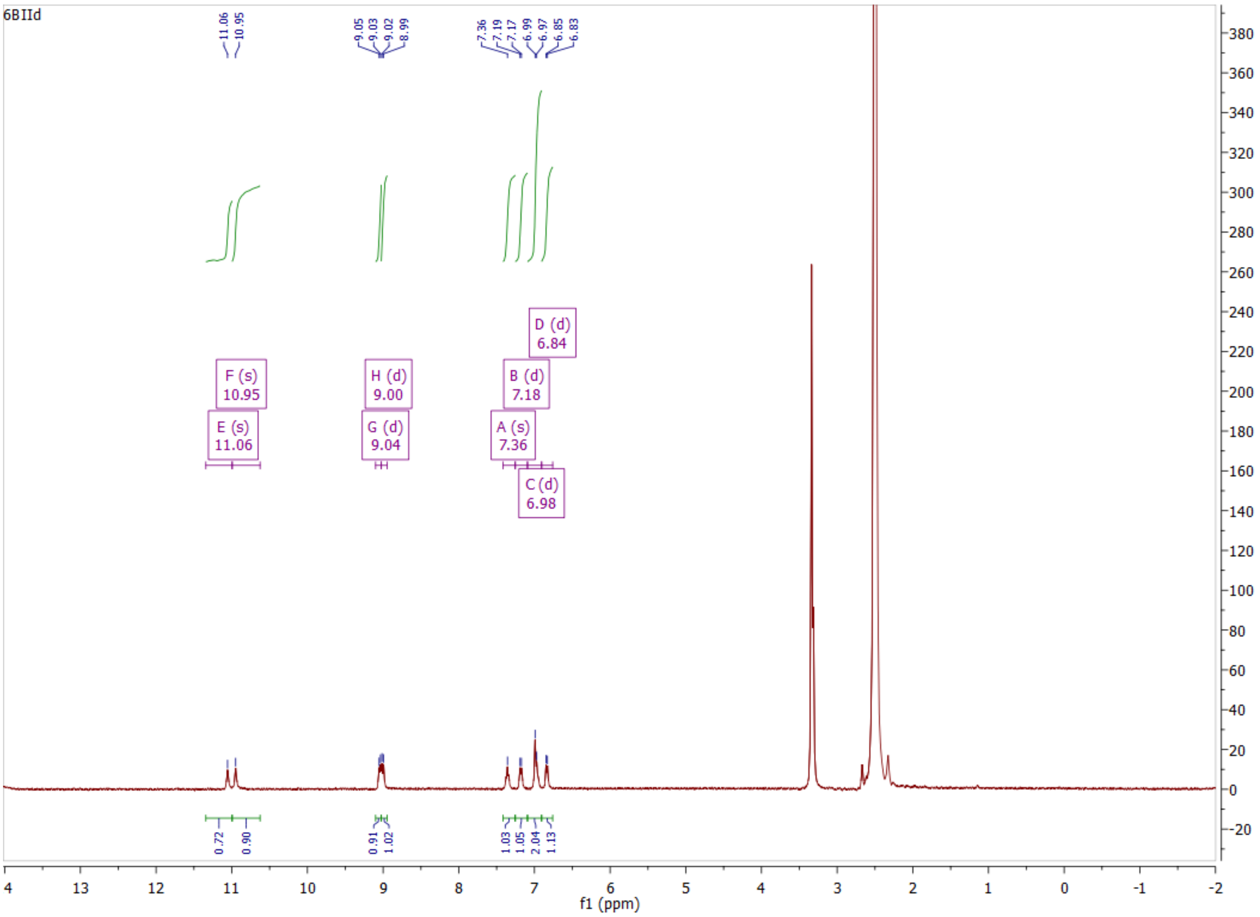

Supplementary Fig. 52 | ^{1}H NMR spectra of 6BIId.

6,6’-Dibromoisoindigo (66DBIId):

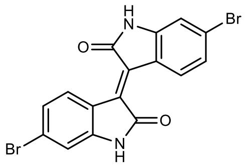

Supplementary Fig. 53 | Chemical structure of 66DBIId.

The target was synthesised by the general procedure starting with 6-bromoisatin (533 mg, 2.36 mmol) and 6-bromo-2-oxindole (500 mg, 2.36 mmol) in a day to afford the desired product (776 mg, 78%). ^{1}H NMR (400 MHz, DMSO) δ 11.10 (s, 2H), 8.99 (d, *J* = 8.7 Hz, 2H), 7.18 (dd, *J* = 8.7, 2.0 Hz, 2H), 6.99 (d, *J* = 2.0 Hz, 2H).

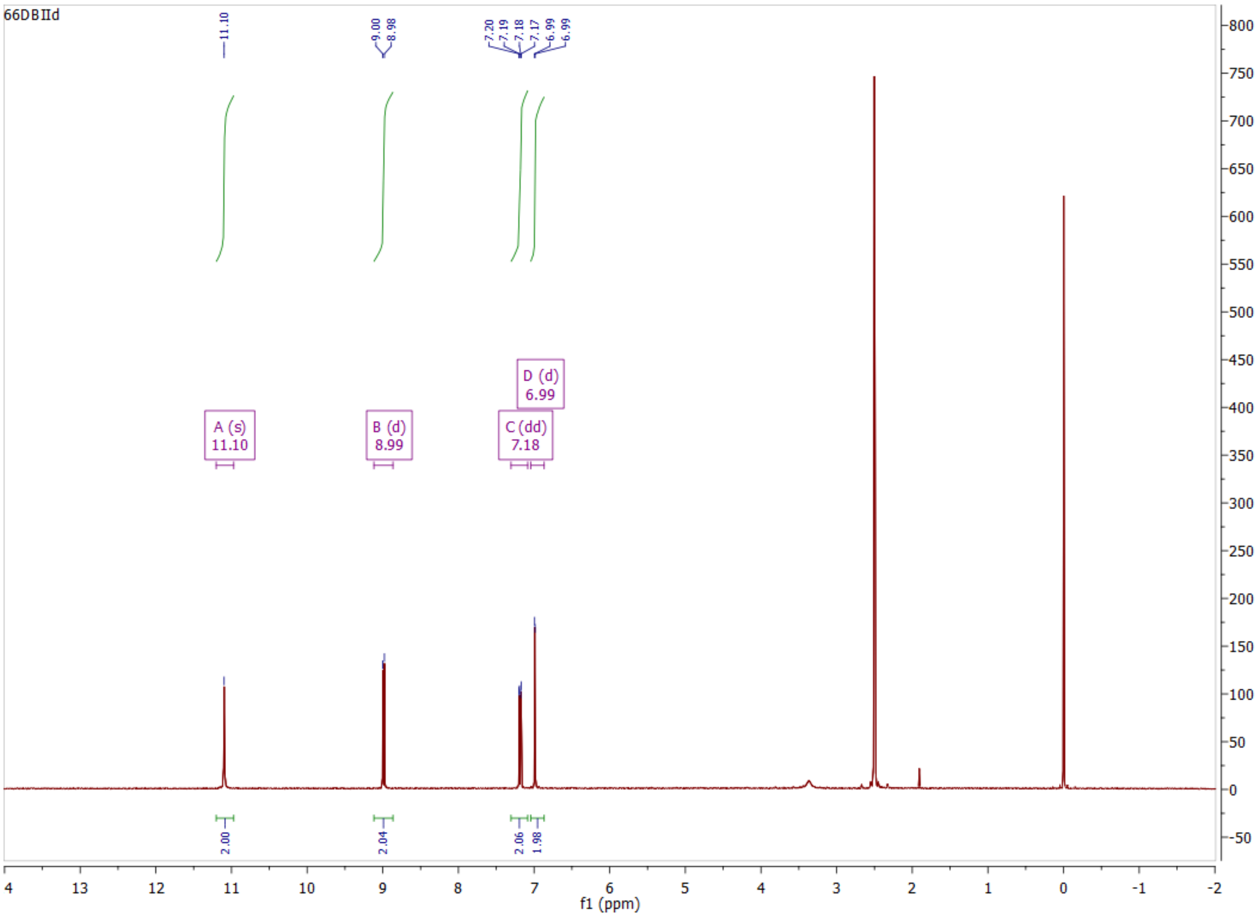

Supplementary Fig. 54 | ^{1}H NMR spectra of 66DBIId.

5,5'-Dibromoisoindigo (55DBIId):

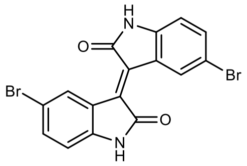

Supplementary Fig. 55 | Chemical structure of 55DBIId.

The target was synthesised by the general procedure starting with 5-bromoisatin (2.132 g, 9.44 mmol) and 5-bromo-2-oxindole (2 g, 9.44 mmol) in a day to afford the desired product (3.87, 97%). ^{1}H NMR (400 MHz, DMSO) δ 11.11 (s, 2H), 9.32 (d, *J* = 2.0 Hz, 2H), 7.55 (dd, *J* = 8.3, 2.1 Hz, 2H), 6.83 (d, *J* = 8.3 Hz, 2H).

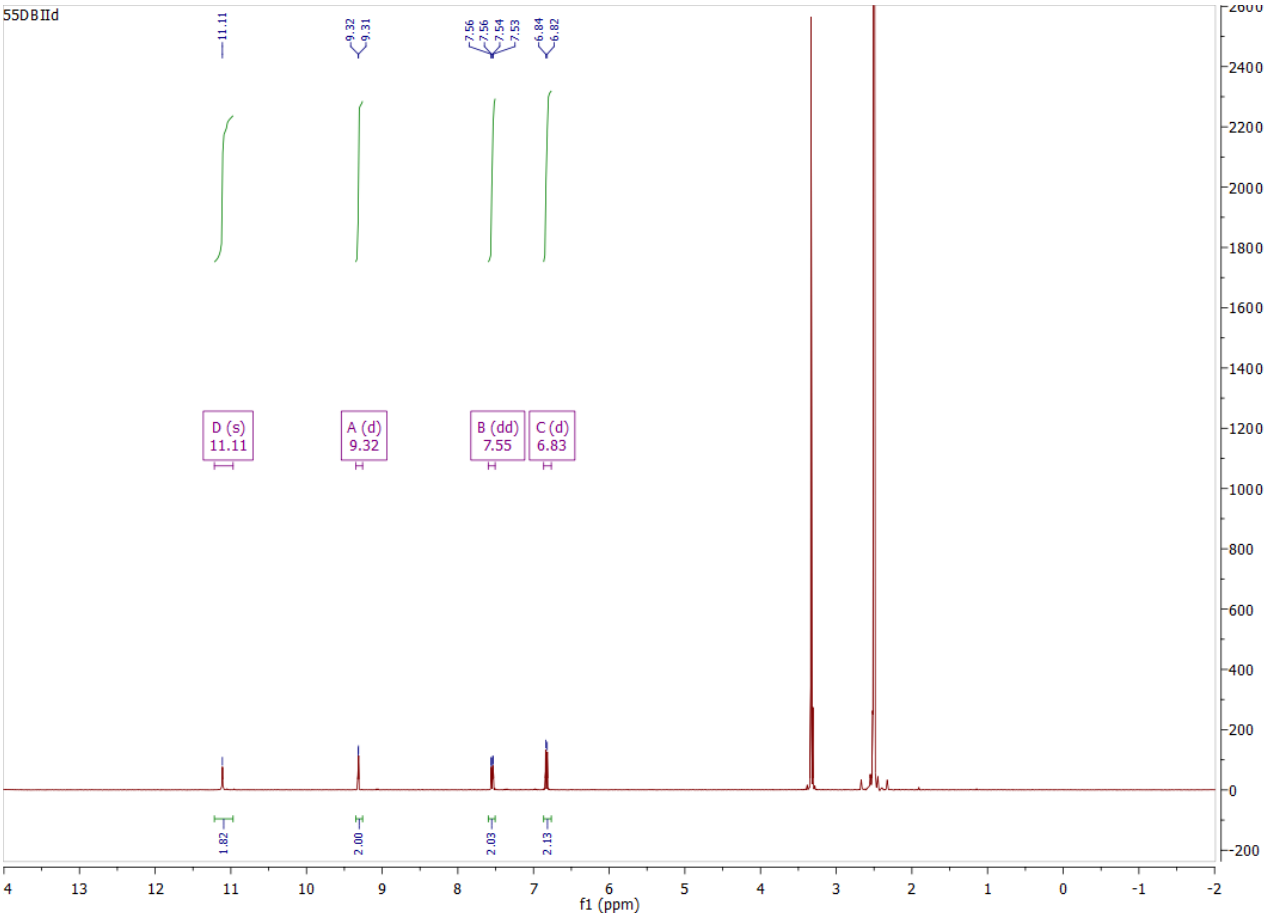

Supplementary Fig. 56 | ^{1}H NMR spectra of 55DBIId.

5-Nitroisoindigo (5NIId):

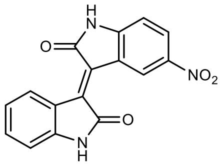

Supplementary Fig. 57 | Chemical structure of 5NIId.

The target was synthesised by the general procedure starting with 5-nitroisatin (227 mg, 1.18 mmol) and 2-oxindole (157.1 mg, 1.18 mmol) in three days to afford the desired product (339 mg, 93%). ^{1}H NMR (400 MHz, DMSO) δ 11.63 (s, 1H), 11.03 (s, 1H), 10.12 (d, *J* = 2.3 Hz, 1H), 9.08 (d, *J* = 8.1 Hz, 1H), 8.29 (dd, *J* = 8.7, 2.4 Hz, 1H), 7.40 (t, *J* = 7.6 Hz, 1H), 7.02 (dd, *J* = 15.7, 8.0 Hz, 2H), 6.88 (d, *J* = 7.6 Hz, 1H).

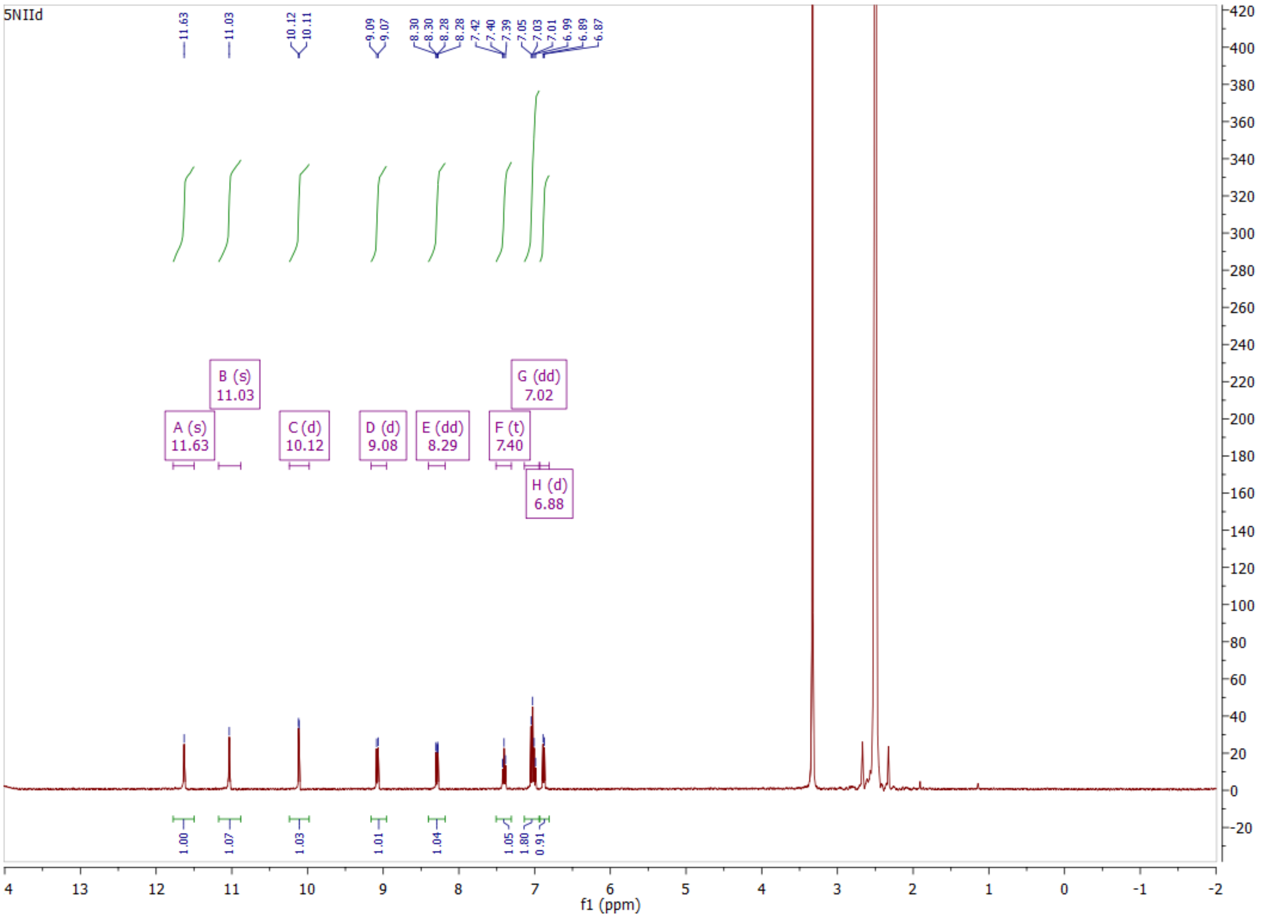

Supplementary Fig. 58 | ^{1}H NMR spectra of 5NIId.

5-Methoxyl-5’-nitroisoindigo (5M5NIId):

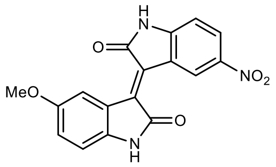

Supplementary Fig. 59 | Chemical structure of 5M5NIId.

The target was synthesised by the general procedure starting with 5-nitroisatin (227 mg, 1.18 mmol) and 5-methoxy-2-oxindole (192.6 mg, 1.18 mmol) in three days to afford the desired product (309 mg, 78%). ^{1}H NMR (400 MHz, DMSO) δ 11.61 (s, 1H), 10.84 (s, 1H), 10.14 (d, *J* = 2.3 Hz, 1H), 8.83 (d, *J* = 2.6 Hz, 1H), 8.29 (dd, *J* = 8.7, 2.4 Hz, 1H), 7.08 – 6.98 (m, 2H), 6.79 (d, *J* = 8.5 Hz, 1H), 3.75 (s, 3H). ^{13}C NMR (101 MHz, DMSO) δ 169.23, 168.89, 154.08, 149.26, 141.68, 138.81, 136.55, 130.89, 128.25, 124.98, 121.91, 121.39, 119.63, 115.68, 110.10, 109.64, 55.49. Low-res ESI-MS (m/z): $[M-H]^-$ calcd. for $C_{17}H_{10}N_3O_5$, 336; found, 336.

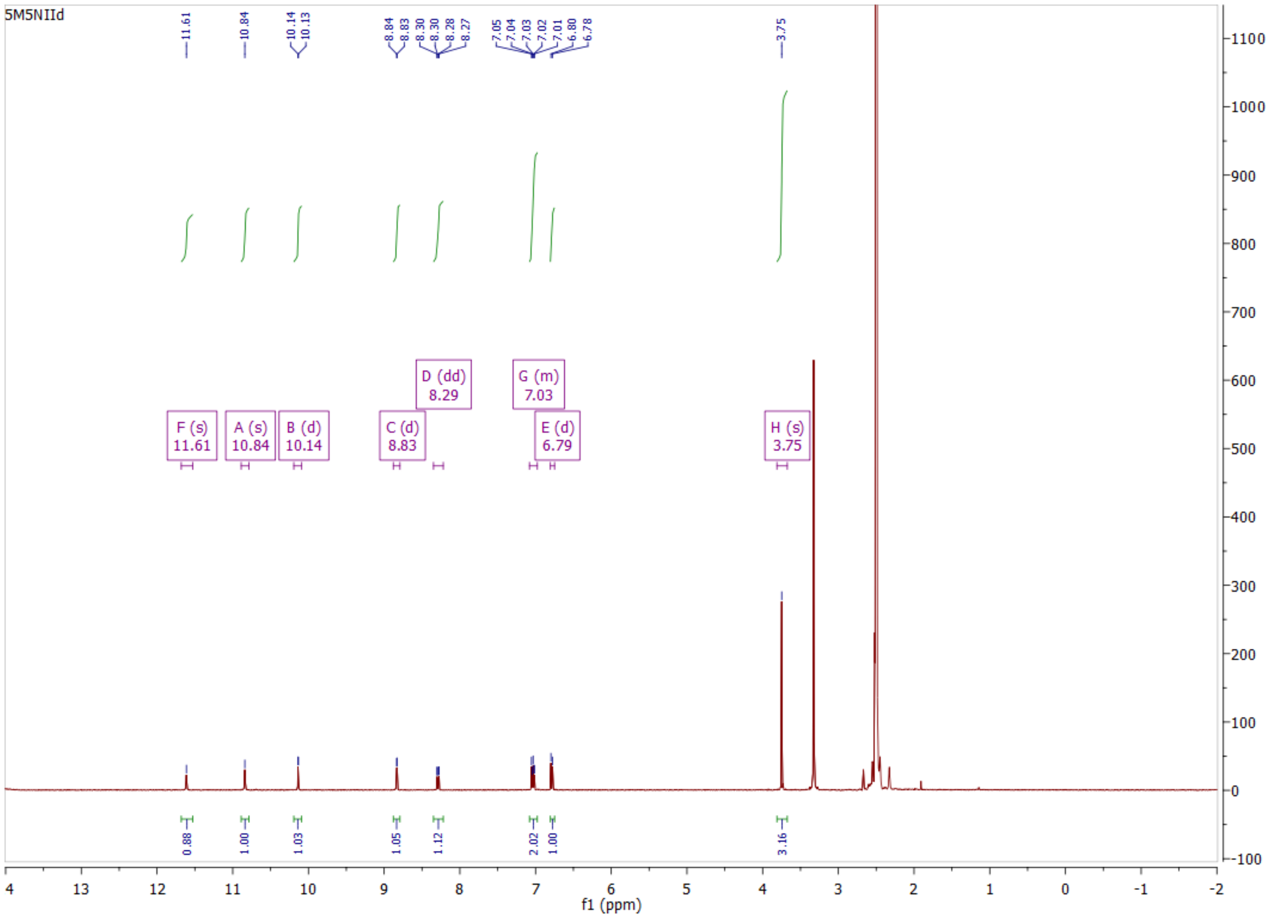

Supplementary Fig. 60 | ^{1}H NMR spectra of 5M5NIId.

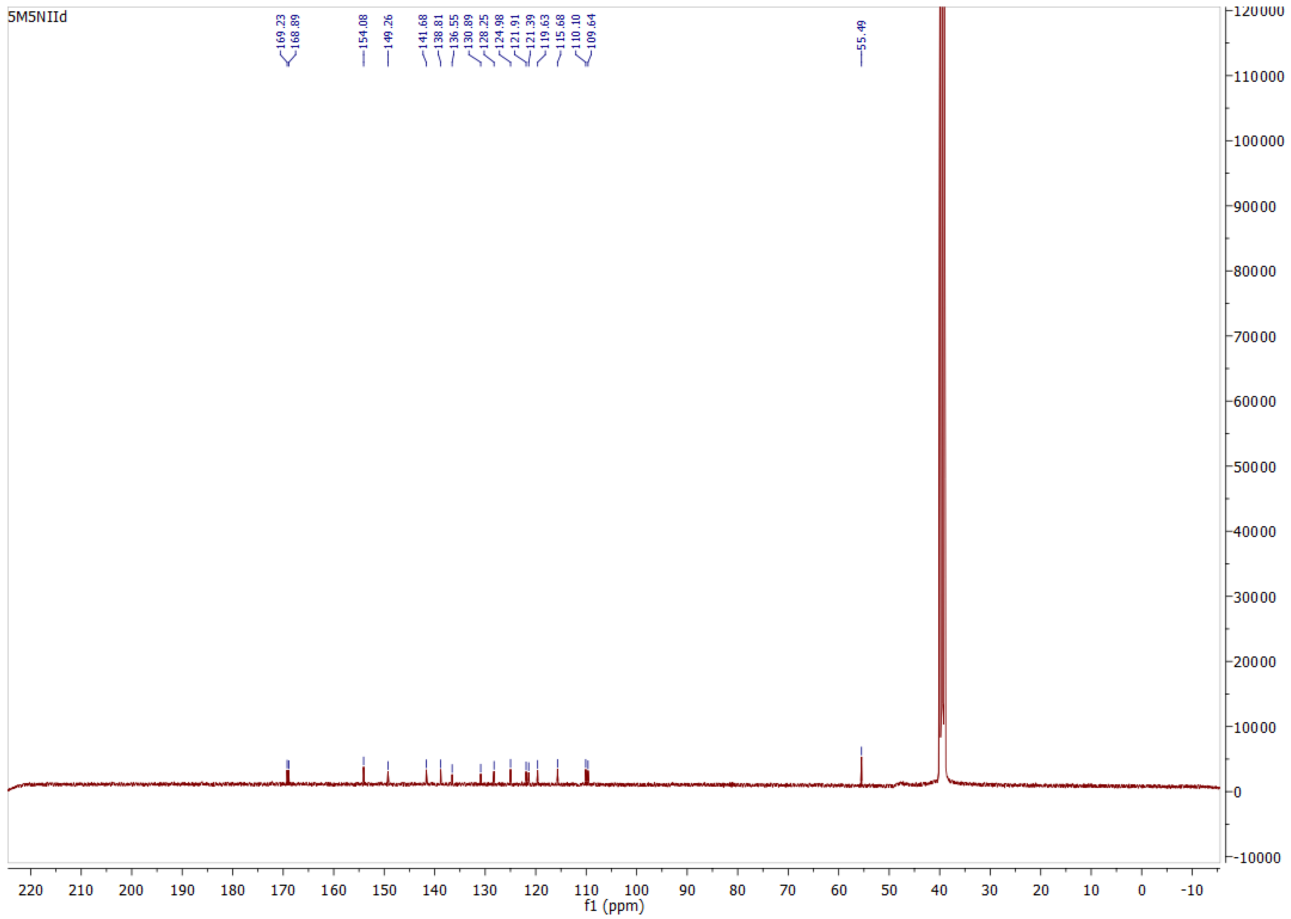

Supplementary Fig. 61 | ^{13}C NMR spectra of 5M5NIId.

Methyl isoindigo-6-carboxylate (6MCIId):

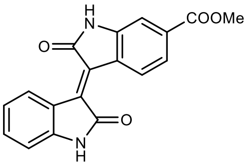

Supplementary Fig. 62 | Chemical structure of 6MCIId.

The target was synthesised by the general procedure starting with isatin (1.47 g, 10 mmol) and methyl 2-oxindole-6-carboxylate (1.91 g, 10 mmol) in a day to afford the desired product (2.93 g, 92%). ^{1}H NMR (400 MHz, DMSO) δ 11.08 (s, 1H), 10.94 (s, 1H), 9.15 (d, *J* = 8.4 Hz, 1H), 9.07 (d, *J* = 8.0 Hz, 1H), 7.57 (dd, *J* = 8.4, 1.7 Hz, 1H), 7.38 (td, *J* = 7.7, 1.2 Hz, 1H), 7.34 (d, *J* = 1.6 Hz, 1H), 7.02 – 6.94 (m, 1H), 6.85 (d, *J* = 7.3 Hz, 1H), 3.87 (s, 3H). ^{13}C NMR (101 MHz, DMSO) δ 168.68, 168.62, 165.57, 144.70, 143.91, 135.73, 133.47, 131.95, 131.79, 129.83, 129.06, 125.68, 121.98, 121.51, 121.28, 109.69, 109.29, 52.29. Low-res ESI-MS (m/z): $[M-H]^-$ calcd. for $C_{18}H_{11}N_2O_4$, 319; found, 319.

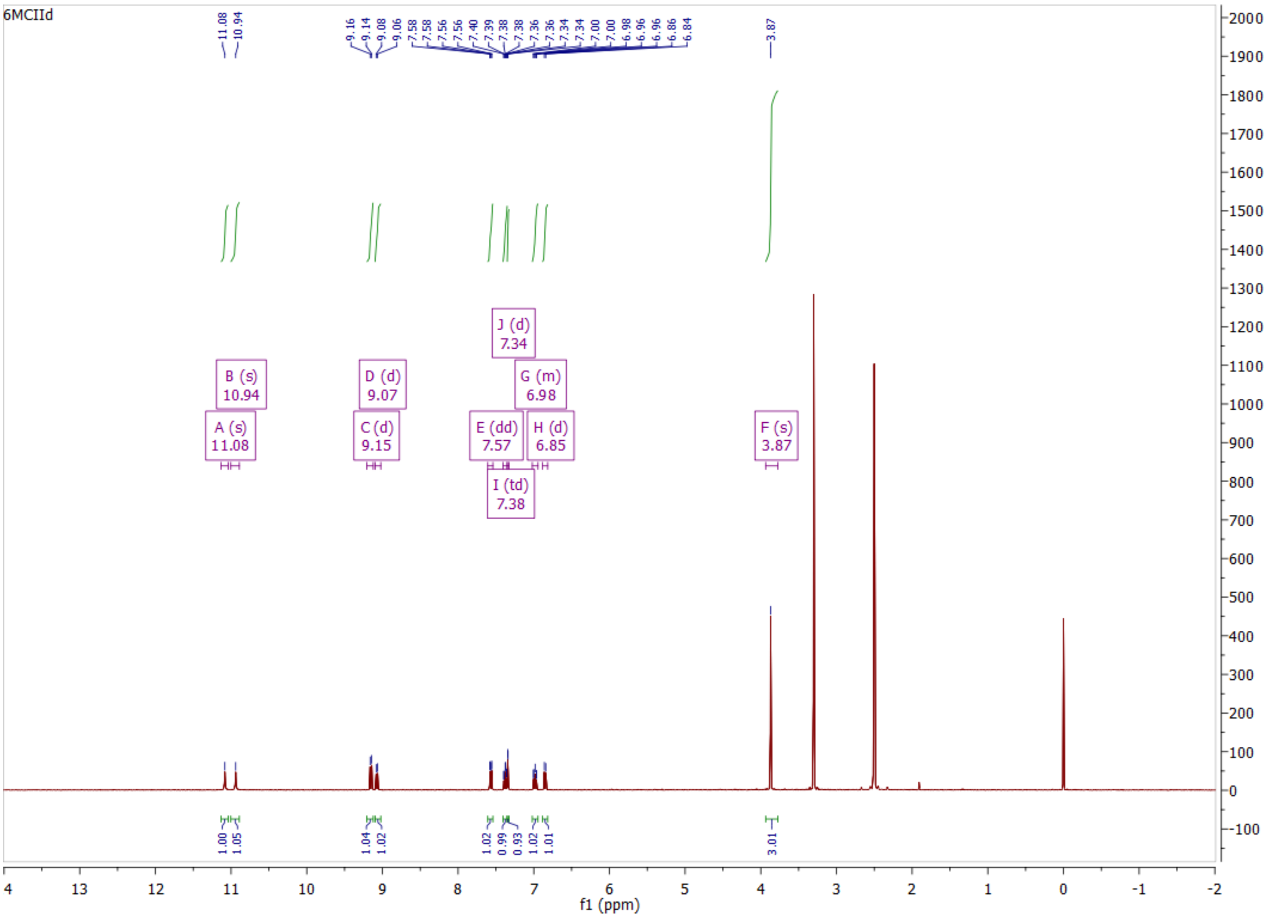

Supplementary Fig. 63 | ^{1}H NMR spectra of 6MCIId.

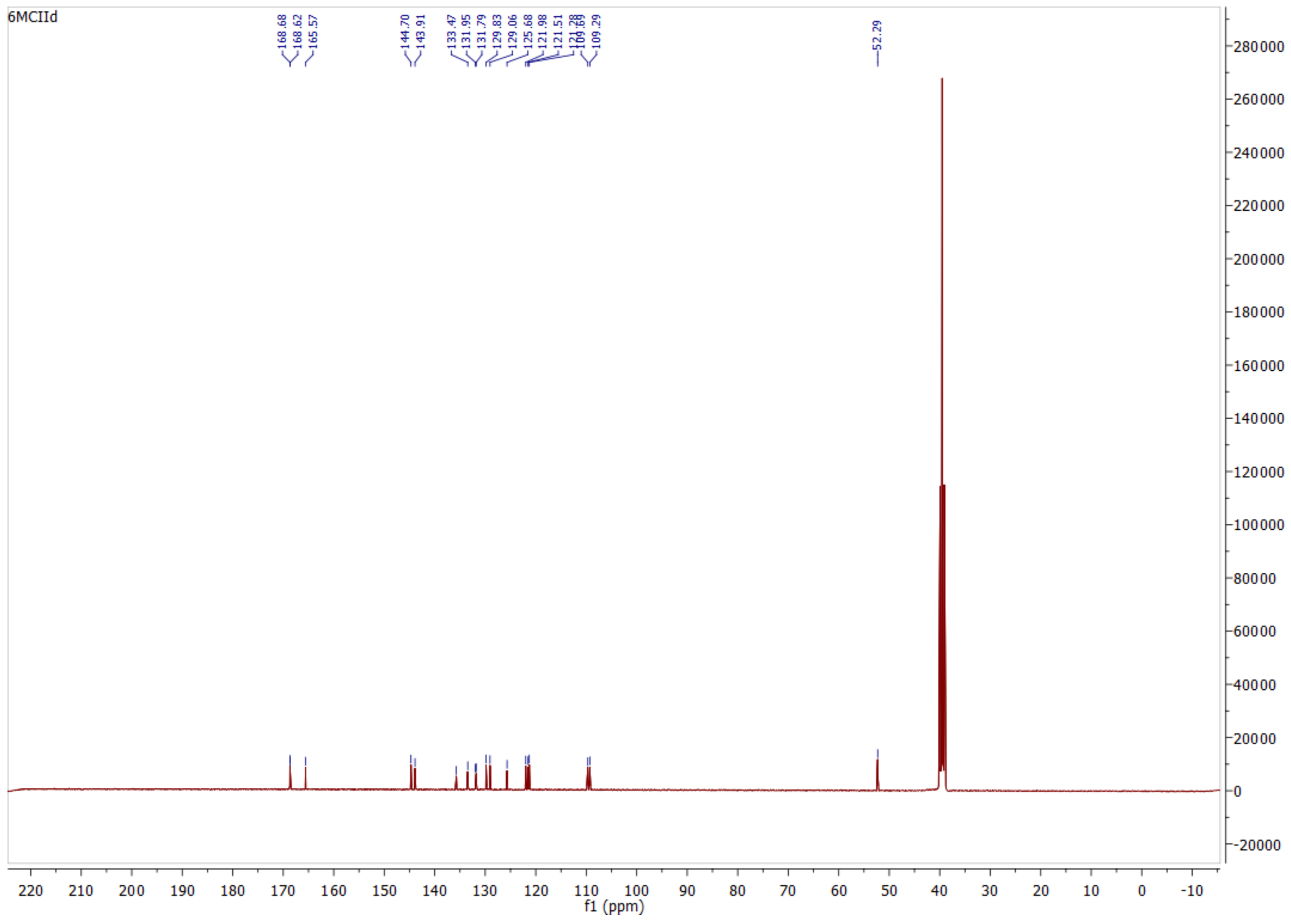

Supplementary Fig. 64 | ^{13}C NMR spectra of 6MCIId.

Methyl 6-bromoisoindigo-6’-carboxylate (6B6MCIId):

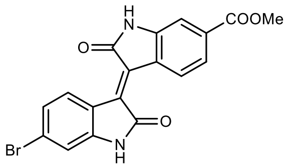

Supplementary Fig. 65 | Chemical structure of 6B6MCIId.

The target was synthesised by the general procedure starting with 6-bromoisatin (1.13 g, 5 mmol) and methyl 2-oxindole-6-carboxylate (956 mg, 5 mmol) in a day to afford the desired product (1.74 g, 87%). ^{1}H NMR (400 MHz, DMSO) δ 11.12 (s, 1H), 11.09 (s, 1H), 9.14 (d, J = 8.4 Hz, 1H), 9.02 (d, J = 8.7 Hz, 1H), 7.57 (dd, J = 8.4, 1.7 Hz, 1H), 7.33 (d, J = 1.6 Hz, 1H), 7.20 (dd, J = 8.7, 2.0 Hz, 1H), 7.01 (d, J = 2.0 Hz, 1H), 3.87 (s, 3H). ^{13}C NMR (101 MHz, DMSO) δ 168.66, 168.52, 165.54, 145.89, 144.11, 134.37, 132.46, 132.24, 131.25, 129.22, 126.30, 125.56, 124.01, 122.09, 120.63, 112.41, 109.40, 52.34. Low-res ESI-MS (m/z): $[M-H]^{-}$ calcd. for $C_{18}H_{10}BrN_2O_4$, 397; found, 397.

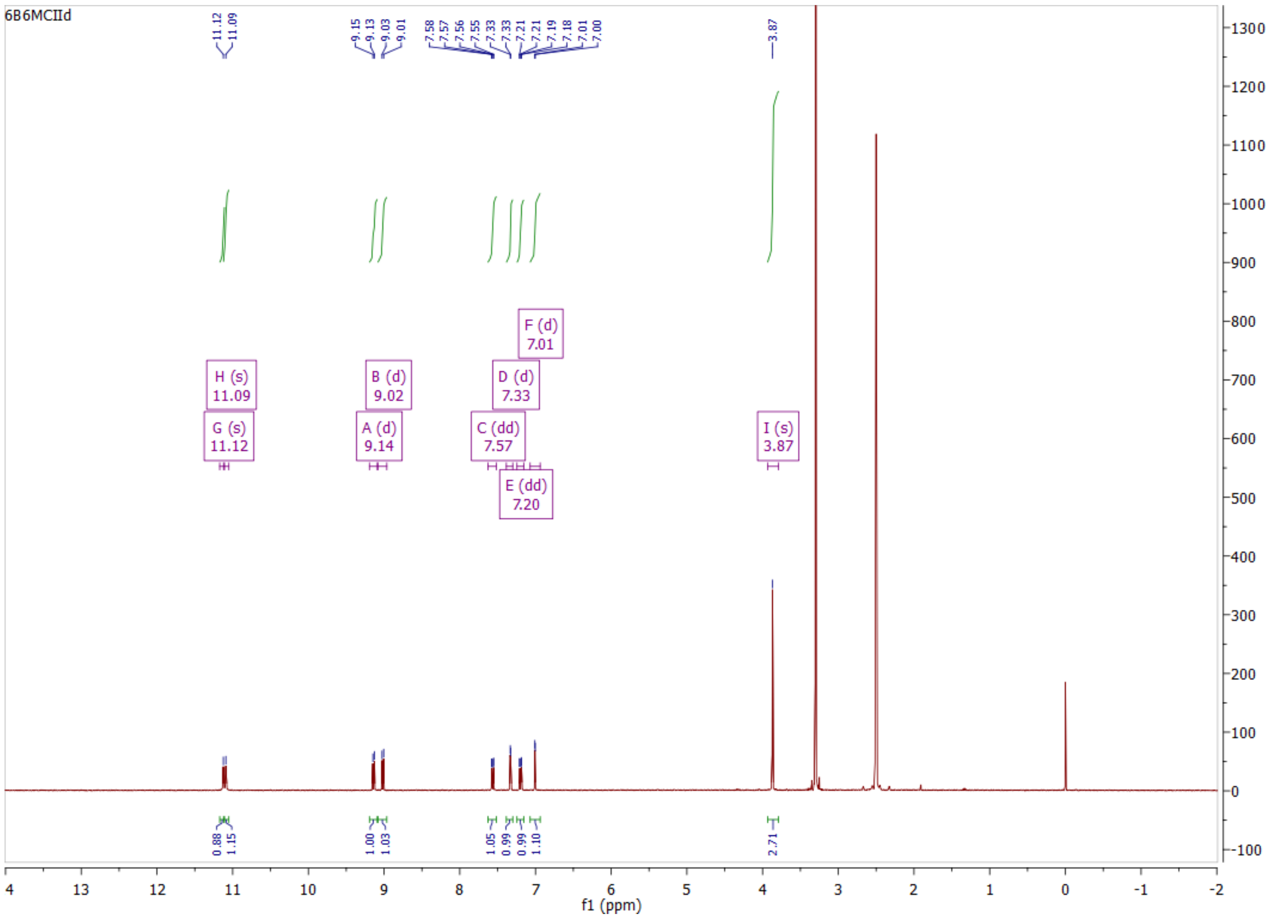

Supplementary Fig. 66 | ^{1}H NMR spectra of 6B6MCIId.

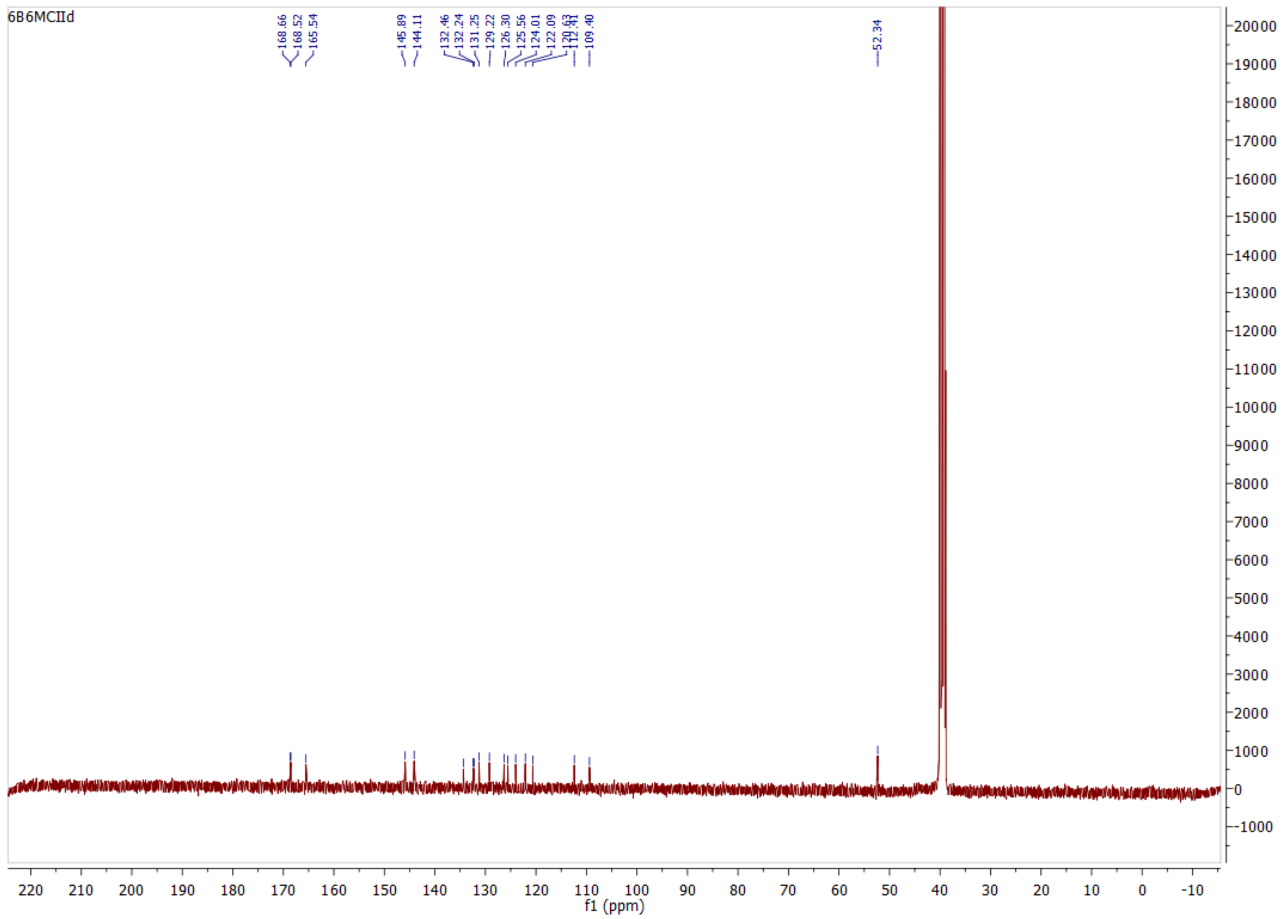

Supplementary Fig. 67 | ^{13}C NMR spectra of 6B6MCIId.

5,5',6,6'-Tetrafluoroisoindigo (5566TFIId):

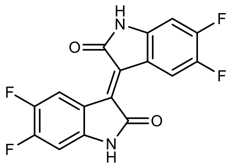

Supplementary Fig. 68 | Chemical structure of 5566TFIId.

The target was synthesised by the general procedure starting with 5,6-difluoroindoline-2,3-dione (366.2 mg, 2 mmol) and 5,6-difluoroindolin-2-one (338.3 mg, 2 mmol) in three days to afford the desired product (345 mg, 52%). ^{1}H NMR (400 MHz, DMSO) δ 11.20 (s, 2H), 9.21 (dd, *J* = 13.3, 8.7 Hz, 2H), 6.90 (dd, *J* = 10.2, 7.1 Hz, 2H). HRMS (m/z): [M−H]$^{-}$ calcd. for $C_{16}H_{5}F_{4}N_{2}O_{2}$, 333.0293; found, 333.0291.

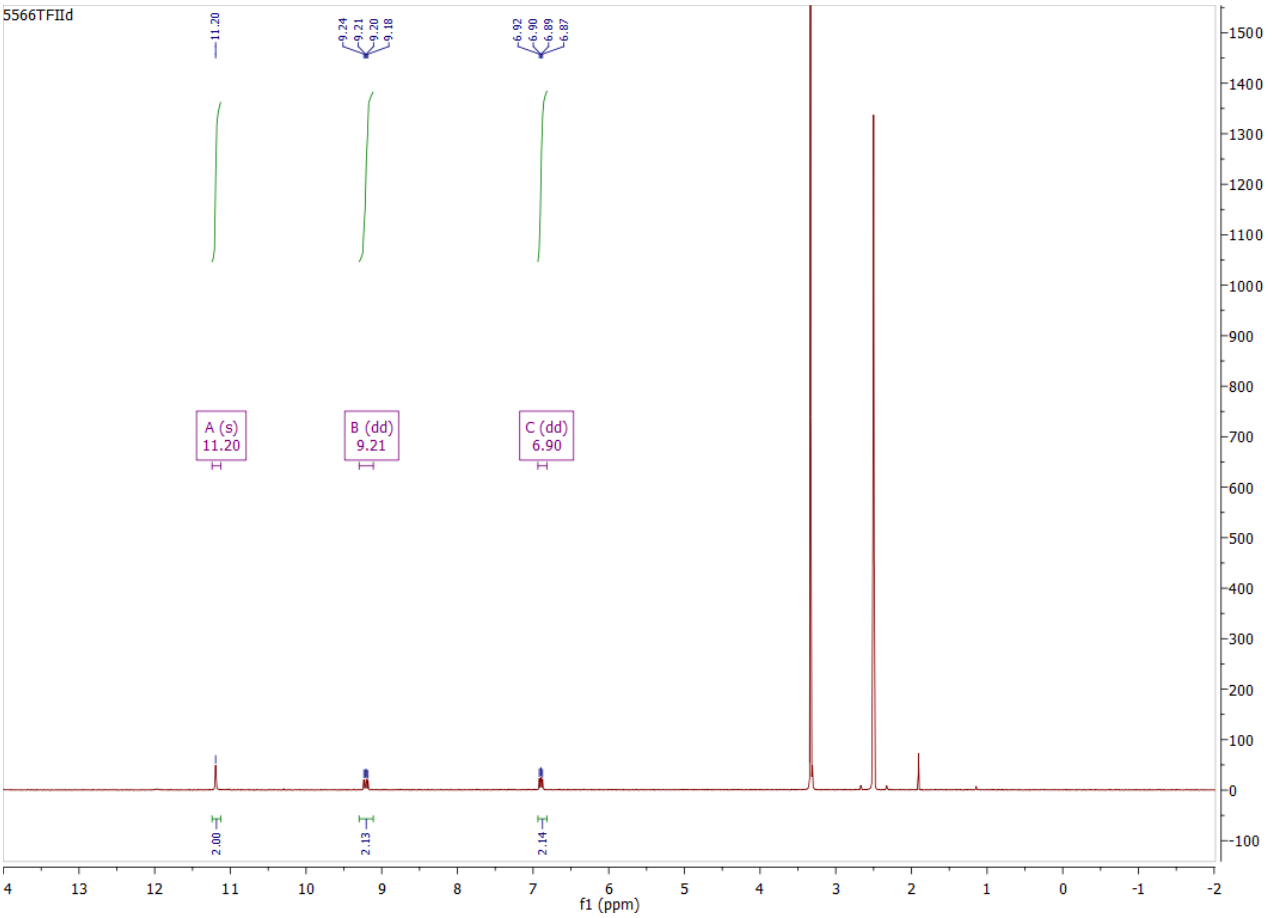

Supplementary Fig. 69 | ^{1}H NMR spectra of 5566TFIId.

Methyl-5-nitroisoindigo-6-carboxylate (5N6MCIId):

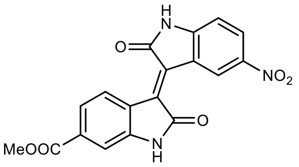

Supplementary Fig. 70 | Chemical structure of 5N6MCIId.

The target was synthesised by the general procedure starting with 5-nitroisatin (960.7 mg, 5 mmol) and methyl 2-oxindole-6-carboxylate (956 mg, 5 mmol) in three days to afford the desired product (1.77 g, 97%). ^{1}H NMR (400 MHz, DMSO) δ 11.68 (s, 1H), 11.22 (s, 1H), 10.10 (s, 1H), 9.15 (d, J = 8.4 Hz, 1H), 8.30 (dd, J = 8.7, 2.1 Hz, 1H), 7.57 (d, J = 8.5 Hz, 1H), 7.33 (s, 1H), 7.03 (d, J = 8.7 Hz, 1H), 3.87 (s, 3H). HRMS (m/z): $[M-H]^-$ calcd. for $C_{18}H_{10}N_3O_6$, 364.0575; found, 364.0574.

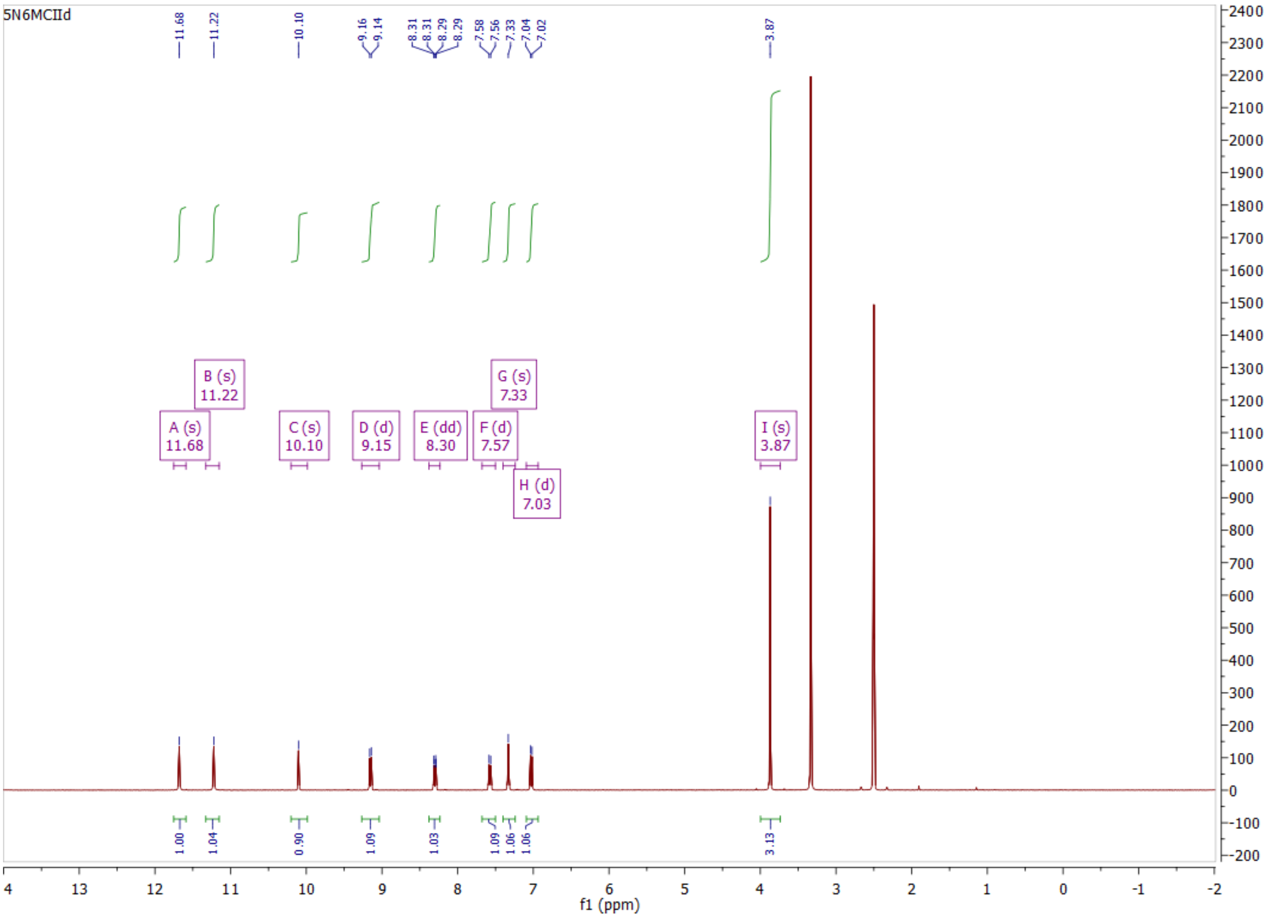

Supplementary Fig. 71 | ^{1}H NMR spectra of 5N6MCIId.

Dimethyl isoindigo-6,6'-dicarboxylate (66DMCIId):

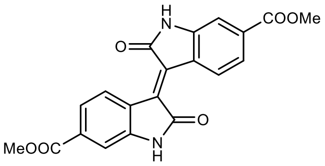

Supplementary Fig. 72 | Chemical structure of 66DMCIId.

The target was synthesised by the general procedure starting with methyl isatin-6-carboxylate (615.5 mg, 3 mmol) and methyl 2-oxindole-6-carboxylate (573.6 mg, 3 mmol) in three days to afford the desired product (1.1 g, 97%). ^{1}H NMR (400 MHz, DMSO) δ 11.18 (s, 2H), 9.17 (d, J = 8.2 Hz, 2H), 7.58 (d, J = 7.9 Hz, 2H), 7.33 (s, 2H), 3.87 (s, 6H). HRMS (m/z): [M−H]$^{-}$ calcd. for $C_{20}H_{13}N_2O_6$, 377.0779; found, 377.0785.

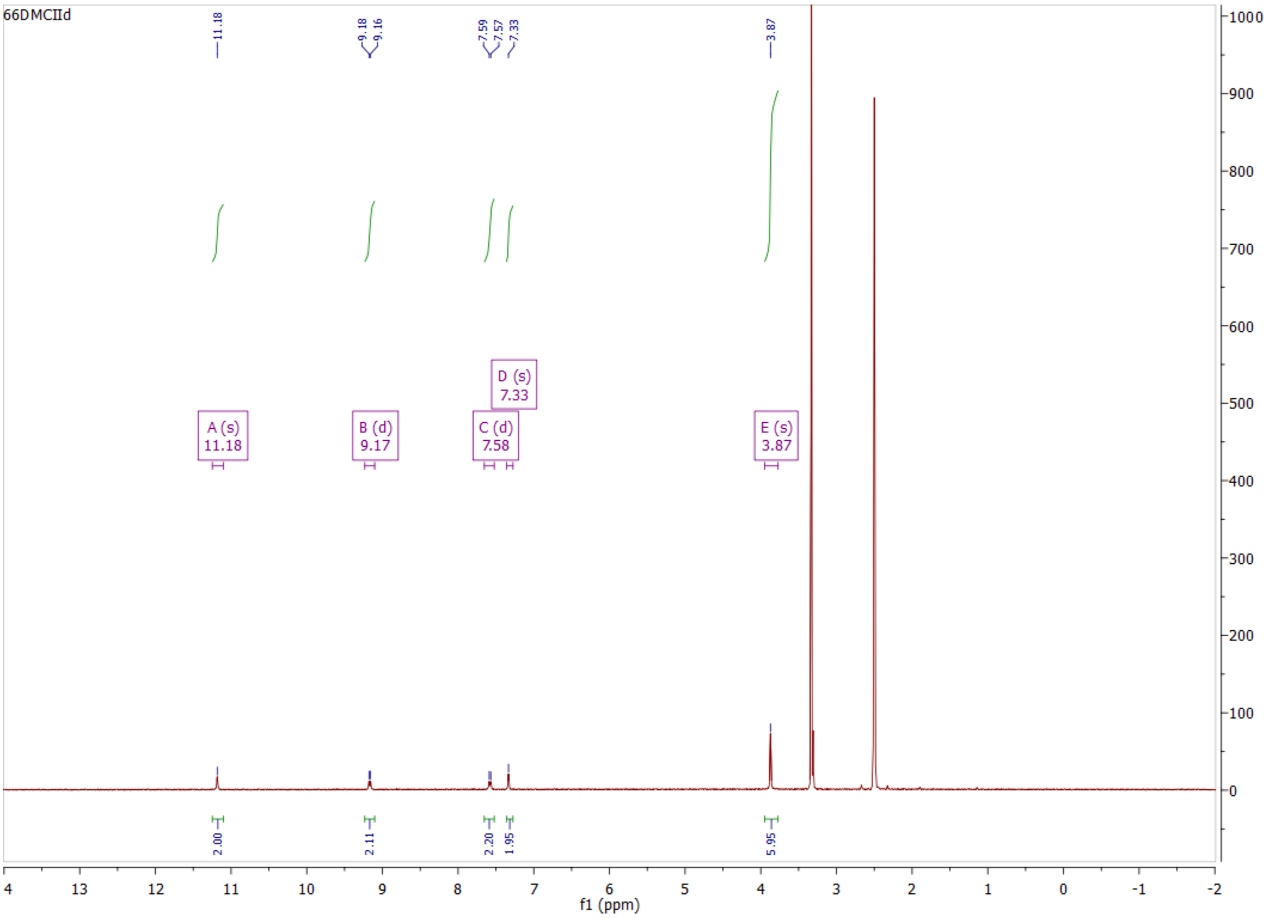

Supplementary Fig. 73 | ^{1}H NMR spectra of 66DMCIId.

2-Oxoindoline-6-carboxylic acid:

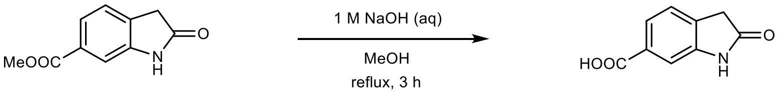

Supplementary Fig. 74 | Synthesis of 2-oxoindoline-6-carboxylic acid.

Methyl 2-oxindole-6-carboxylate (2 g, 10.4 mmol) was suspended in a mixture of methanol (20 ml) and 1 M NaOH (aq, 21 ml). The mixture was heated at reflux for 3 hours and allowed to cool down to room temperature. The solution was neutralised with 1 M HCl (aq) at 0 °C and a beige colour precipitate was formed. The precipitate was collected by filtration and washed with water and methanol. The crude product was dried in vacuo at 60 °C to give a beige solid (1.8 g, 97%), which was used in the next step without further purification. ^{1}H NMR (400 MHz, DMSO) δ 12.85 (s, 1H), 10.49 (s, 1H), 7.56 (d, J = 7.6 Hz, 1H), 7.33-7.30 (2H), 3.55 (s, 2H).

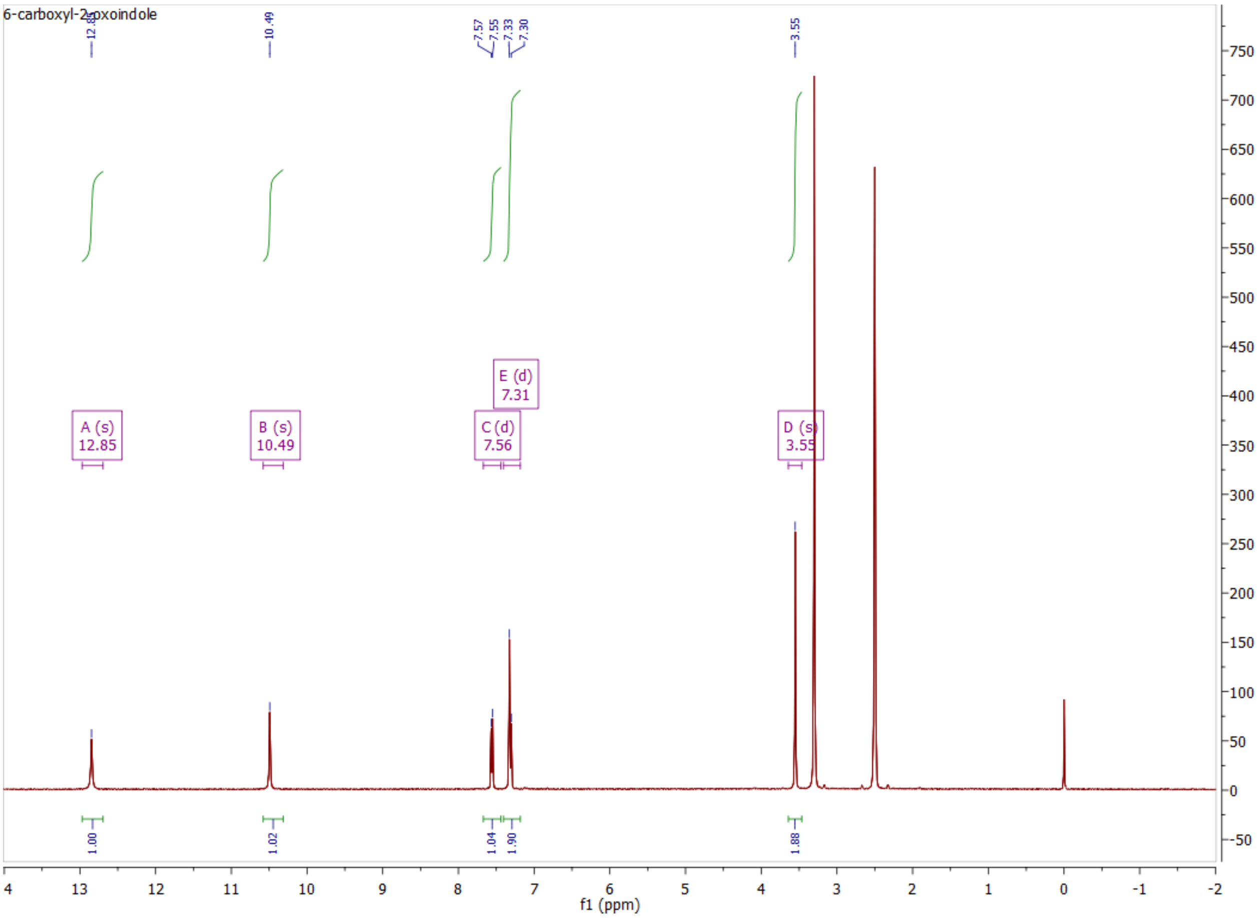

Supplementary Fig. 75 | ^{1}H NMR spectra of 2-oxoindoline-6-carboxylic acid.

Isoindigo-6-carboxylic acid (6CIId):

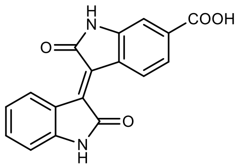

Supplementary Fig. 76 | Chemical structure of 6CIId.

The target was synthesised by the general procedure starting with isatin (1.47 g, 10 mmol) and 2-oxindoline-6-carboxylic acid (1.77 g, 10 mmol) in a day to afford the desired product (2.55 g, 83%). ^{1}H NMR (400 MHz, DMSO) δ 13.10 (s, 1H), 11.05 (s, 1H), 10.93 (s, 1H), 9.13 (d, *J* = 8.4 Hz, 1H), 9.07 (d, *J* = 8.1 Hz, 1H), 7.55 (dd, *J* = 8.4, 1.7 Hz, 1H), 7.46 – 7.23 (m, 2H), 7.04 – 6.93 (m, 1H), 6.85 (d, *J* = 7.2 Hz, 1H). ^{13}C NMR (101 MHz, DMSO) δ 168.74, 168.72, 166.64, 144.64, 143.92, 135.45, 133.42, 132.06, 129.75, 128.98, 125.36, 122.19, 121.54, 121.29, 109.70, 109.57, 104.49. Low-res ESI-MS (m/z): $[M-H]^-$ calcd. for $C_{17}H_9N_2O_4$, 305; found, 305.

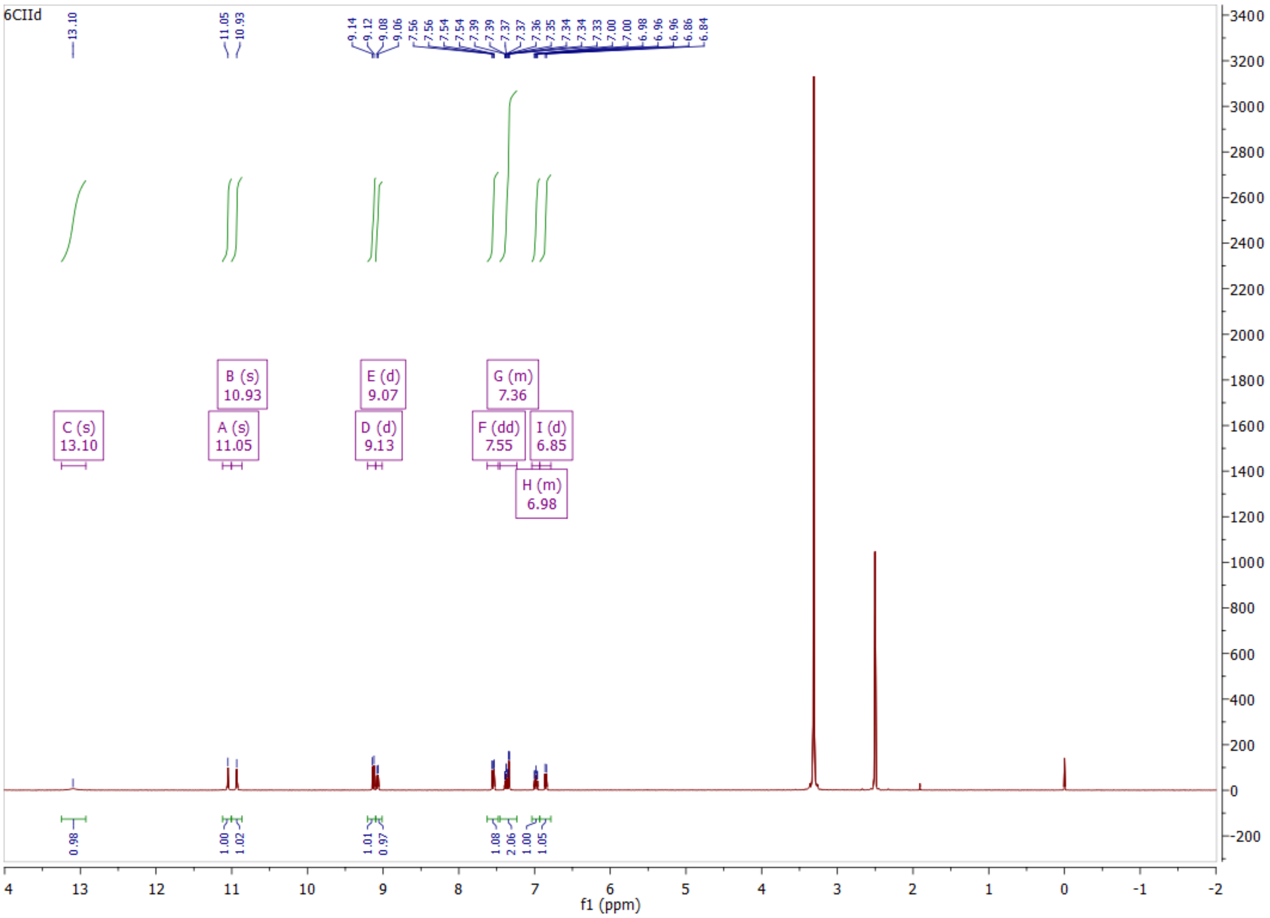

Supplementary Fig. 77 | ^{1}H NMR spectra of 6CIId.

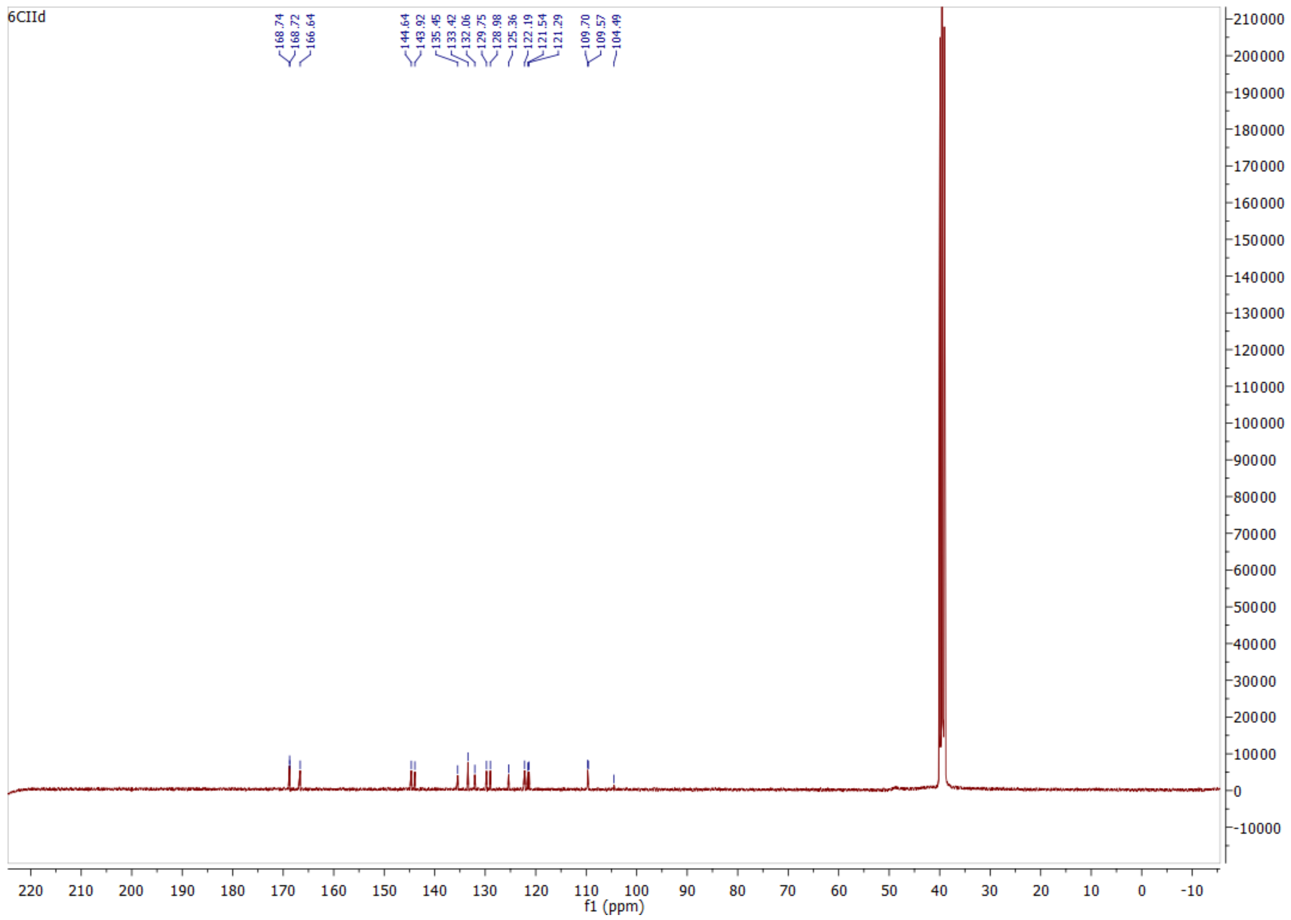

Supplementary Fig. 78 | ^{13}C NMR spectra of 6CIId.

6-Bromoisoindigo-6’-carboxylic acid (6B6CIId):

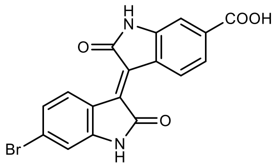

Supplementary Fig. 79 | Chemical structure of 6B6CIId.

The target was synthesised by the general procedure starting with 6-bromoisatin (2.26 g, 10 mmol) and 2-oxindoline-6-carboxylic acid (1.77 g, 10 mmol) in a day to afford the desired product (3.52 g, 91%). ^{1}H NMR (400 MHz, DMSO) δ 13.15 (s, 1H), 11.11 (s, 1H), 11.10 (s, 1H), 9.11 (d, J = 8.4 Hz, 1H), 9.01 (d, J = 8.7 Hz, 1H), 7.54 (dd, J = 8.4, 1.6 Hz, 1H), 7.31 (d, J = 1.3 Hz, 1H), 7.18 (dd, J = 8.7, 2.0 Hz, 1H), 6.98 (d, J = 1.9 Hz, 1H). Low-res ESI-MS (m/z): $[M-H]^-$ calcd. for $C_{17}H_8BrN_2O_4$, 383; found, 383.

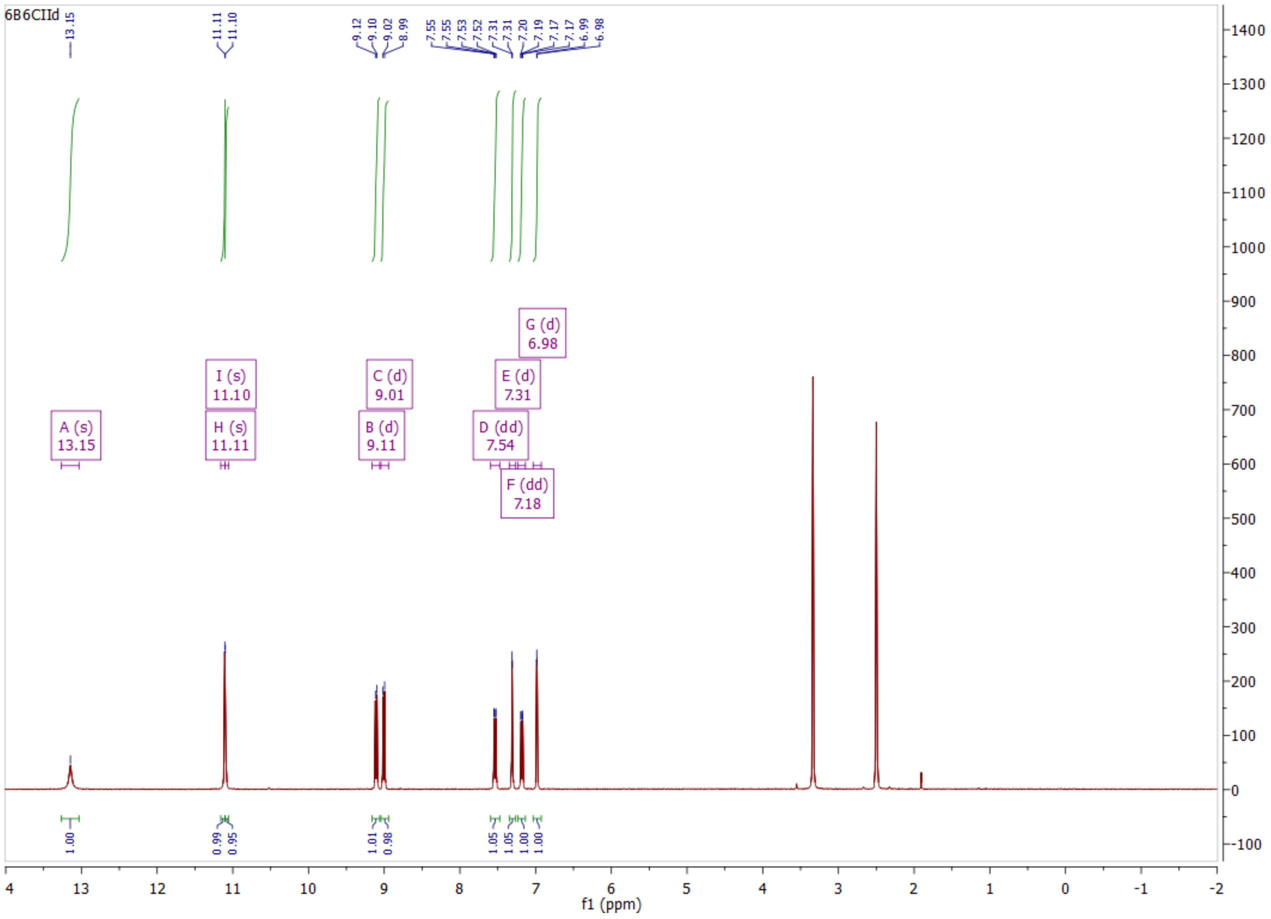

Supplementary Fig. 80 | ^{1}H NMR spectra of 6B6CIId.

Sodium isoindigo-6-carboxylate (6CIIdNa):

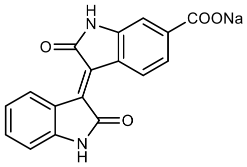

Supplementary Fig. 81 | Chemical structure of 6CIIdNa.

6CIId (306.3 mg, 1 mmol) was added portionwise to a solution of NaOH (0.2 M, 5 ml) in methanol at 0 °C. The mixture was stirred for another 15 min at 0 °C before it was concentrated in vacuo to dryness to afford a brown solid (328 mg, >99%). ^{1}H NMR (400 MHz, DMSO) δ 10.84 (s, 2H), 9.05 (d, *J* = 8.1 Hz, 1H), 8.93 (d, *J* = 8.3 Hz, 1H), 7.40 (d, *J* = 8.3 Hz, 1H), 7.35 – 7.24 (m, 2H), 6.95 (t, *J* = 7.9 Hz, 1H), 6.83 (d, *J* = 7.8 Hz, 1H). Low-res ESI-MS (m/z): $[M-H]^-$ calcd. for $C_{17}H_9N_2O_4$, 305; found, 305.

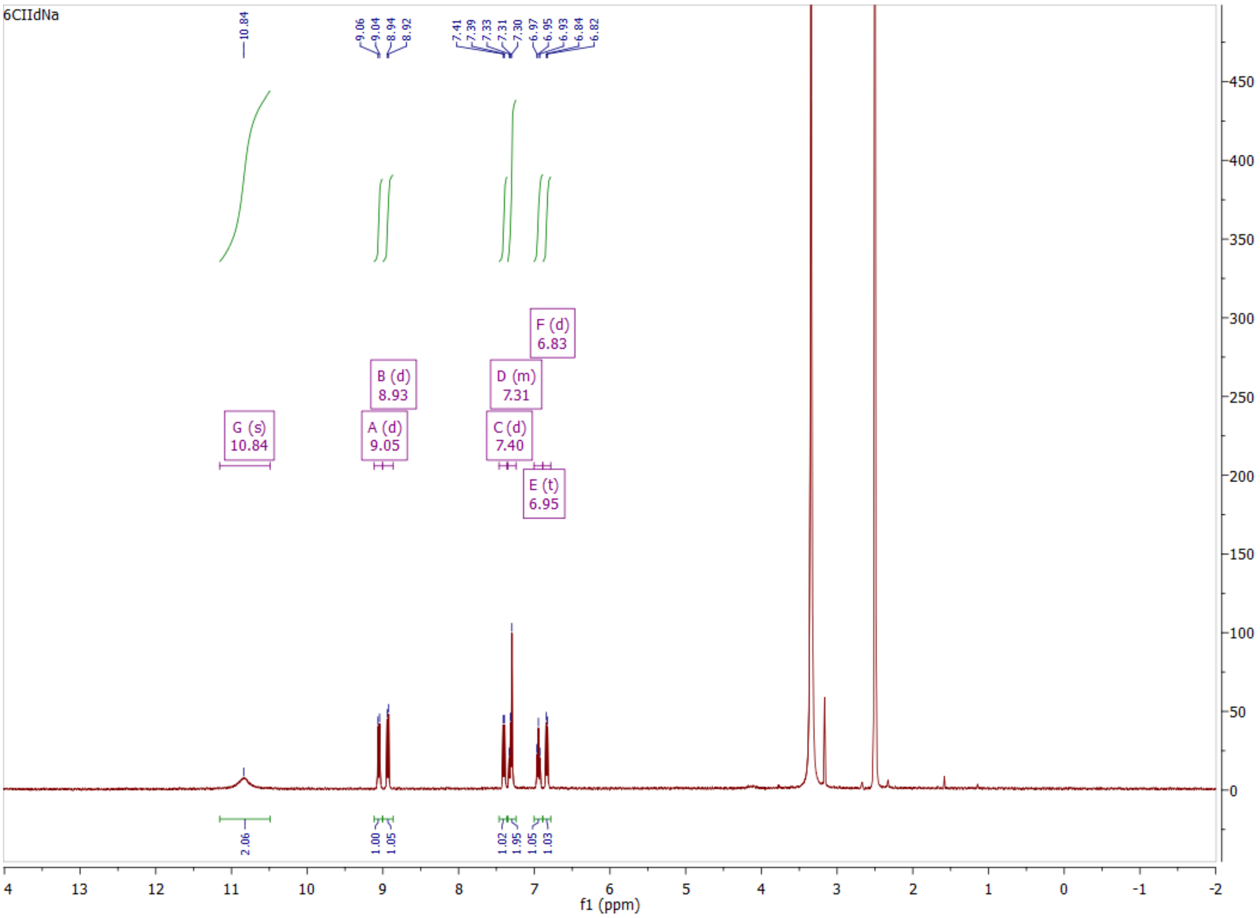

Supplementary Fig. 82 | ^{1}H NMR spectra of 6CIIdNa.

Methyl isoindigo-6-carbonylglycinate (6AIIdGly):

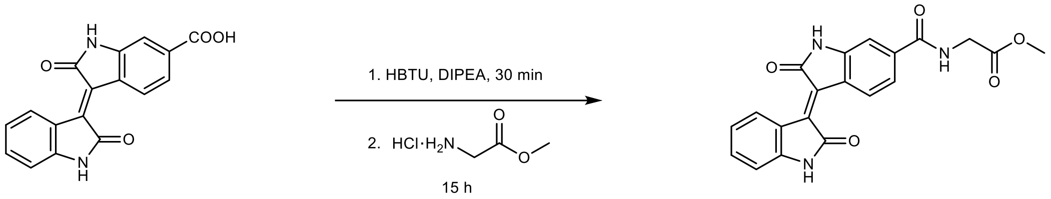

Supplementary Fig. 83 | Synthesis of 6AIIdGly.

To a mixture of 6CIId (1.23 g, 4 mmol) and 2-(1*H*-benzotriazol-1-yl)-1,1,3,3-tetramethyluronium hexafluorophosphate (HBTU) (1.64 g, 4.32 mmol) in anhydrous DMF (25 ml) was added *N*,*N*-diisopropylethylamine (DIPEA) (12 mmol, 2.1 ml) dropwisely. The mixture was stirred in Ar for 30 min before glycine methyl ester hydrochloride (540 mg, 4.32 mmol) in DMF (5 ml) was added. The reaction was monitored by TLC and stirred for another 15 h at room temperature. Upon completion of the reaction, the dark solution was poured onto a saturated $NaHCO_3$ aqueous solution (300 ml). The precipitate was collected by filtration, washed repeatedly with saturated $NaHCO_3$ (aq), water, 0.2 M HCl (aq), cold methanol and dichloromethane, and dried in vacuo to give a dark red solid. The crude product was recrystallised from ethanol to afford a dark red powder (1.284 g, 85%). ^{1}H NMR (400 MHz, DMSO) δ 11.12 (s, 1H), 10.94 (s, 1H), 9.18 – 8.98 (m, 3H), 7.47 (dd, *J* = 8.4, 1.6 Hz, 1H), 7.37 (td, *J* = 7.7, 0.9 Hz, 1H), 7.29 (d, *J* = 1.4 Hz, 1H), 7.03 – 6.94 (m, 1H), 6.85 (d, *J* = 7.6 Hz, 1H), 4.02 (d, *J* = 5.8 Hz, 2H), 3.67 (s, 3H). ^{13}C NMR (101 MHz, DMSO) δ 170.26, 168.91, 168.81, 165.89, 144.50, 144.01, 136.57, 134.87, 133.22, 132.20, 129.65, 128.93, 124.24, 121.57, 121.27, 119.94, 109.67, 108.17, 51.75, 41.24. Low-res ESI-MS (m/z): $[M-H]^-$ calcd. for $C_{20}H_{14}N_3O_5$, 376; found, 376.

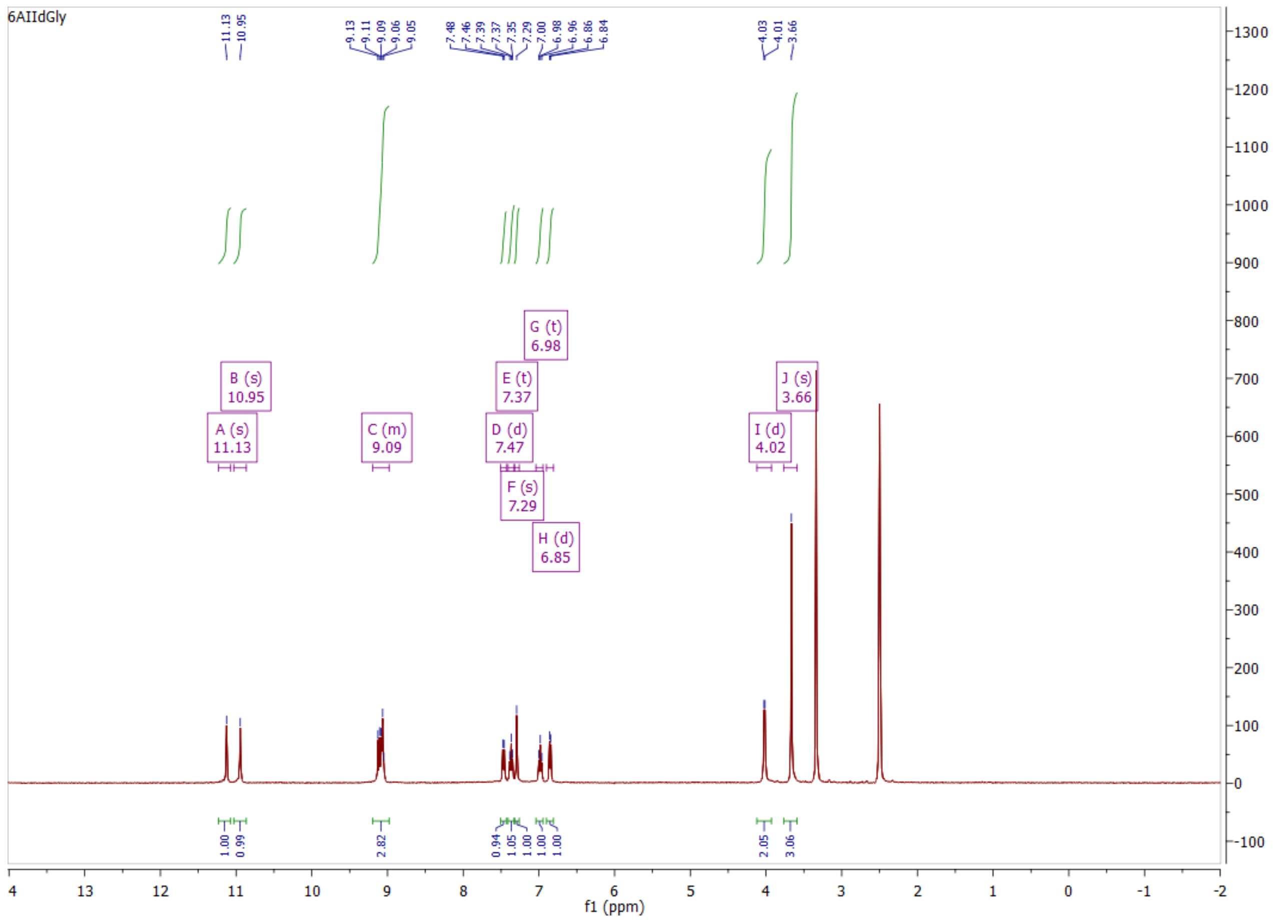

Supplementary Fig. 84 | ^{1}H NMR spectra of 6AIIdGly.

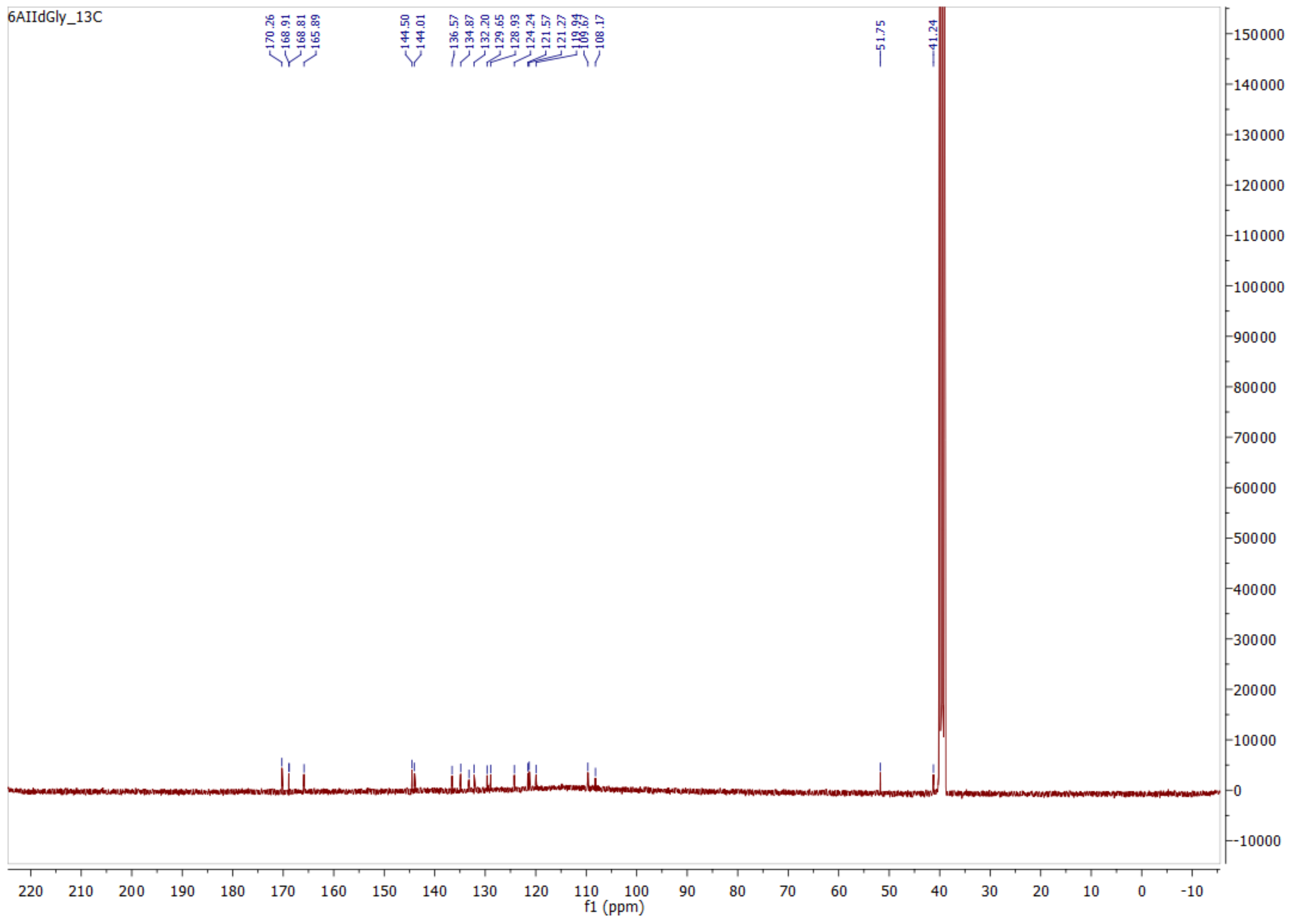

Supplementary Fig. 85 | ^{13}C NMR spectra of 6AIIdGly.

Methyl isoindigo-6-carbonylserinate (6AIIdSer):

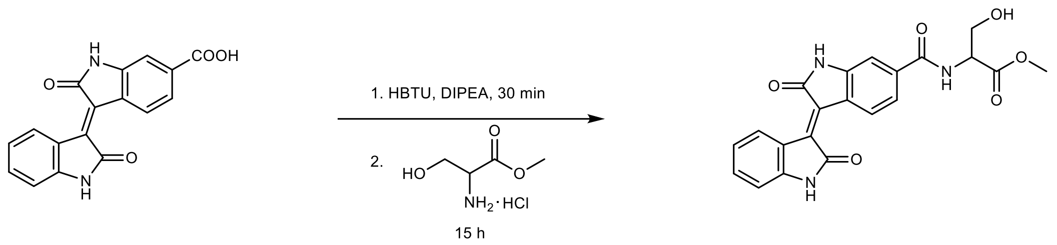

Supplementary Fig. 86 | Synthesis of 6AIIdSer.

To a mixture of 6CIId (1.85 g, 6 mmol) and 2-(1*H*-benzotriazol-1-yl)-1,1,3,3-tetramethyluronium hexafluorophosphate (HBTU) (2.46 g, 6.48 mmol) in anhydrous DMF (30 ml) was added *N*,*N*-diisopropylethylamine (DIPEA) (3.15 ml, 18 mmol) dropwisely. The mixture was stirred in Ar for 30 min before L-serine methyl ester hydrochloride (1.16 g, 6.48 mmol) in DMF (5 ml) was added. The reaction was monitored by TLC and stirred for another 15 h at room temperature. Upon completion of the reaction, the dark solution was poured onto a saturated $NaHCO_3$ aqueous solution (300 ml). The precipitate was collected by filtration, washed repeatedly with saturated $NaHCO_3$ (aq), water, 0.2 M HCl (aq), cold methanol and dichloromethane, and dried in vacuo to give a dark red solid. The crude product was recrystallised from ethanol to afford a dark red powder (1.39 g, 57%). ^{1}H NMR (400 MHz, DMSO) δ 11.12 (s, 1H), 10.93 (s, 1H), 9.12 (d, *J* = 8.4 Hz, 1H), 9.07 (d, *J* = 7.9 Hz, 1H), 8.70 (d, *J* = 7.3 Hz, 1H), 7.49 (dd, *J* = 8.5, 1.4 Hz, 1H), 7.37 (t, *J* = 7.2 Hz, 1H), 7.30 (s, 1H), 6.98 (t, *J* = 7.6 Hz, 1H), 6.85 (d, *J* = 7.6 Hz, 1H), 5.09 (t, *J* = 6.1 Hz, 1H), 4.54 (dd, *J* = 12.6, 5.5 Hz, 1H), 3.80 (t, *J* = 5.7 Hz, 2H), 3.66 (s, 3H). ^{13}C NMR (101 MHz, DMSO) δ 170.98, 168.98, 168.85, 165.90, 144.54, 143.96, 136.75, 134.90, 133.29, 132.27, 129.68, 128.89, 124.25, 121.61, 121.33, 120.28, 109.73, 108.38, 60.96, 55.74, 51.95. Low-res ESI-MS (m/z): $[M-H]^-$ calcd. for $C_{21}H_{16}N_3O_6$, 406; found, 406.

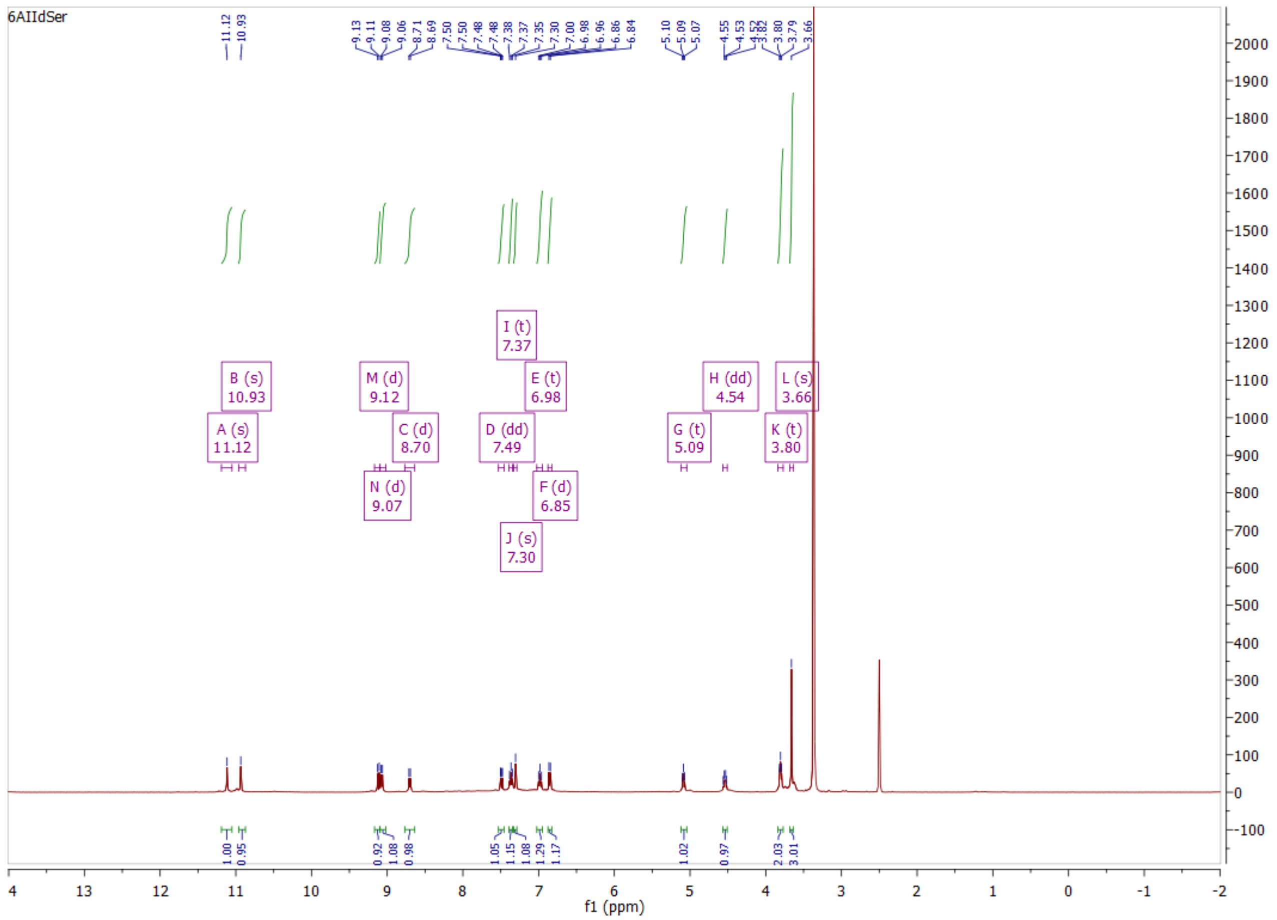

Supplementary Fig. 87 | ^{1}H NMR spectra of 6AIIdSer.

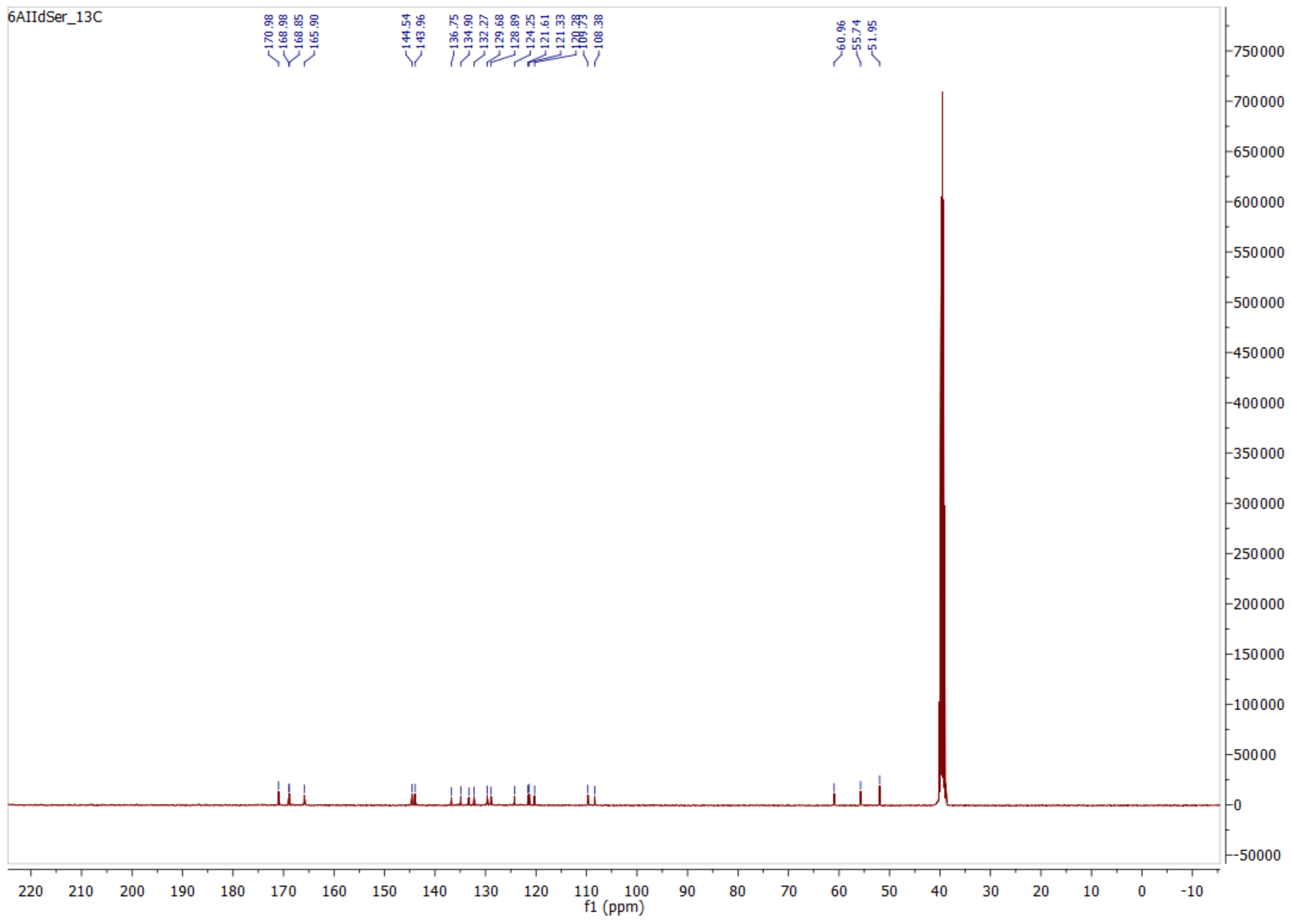

Supplementary Fig. 88 | ^{13}C NMR spectra of 6AIIdSer.

Methyl 6-bromoisoindigo-6'-carbonylserinate (6B6AIIdSer):

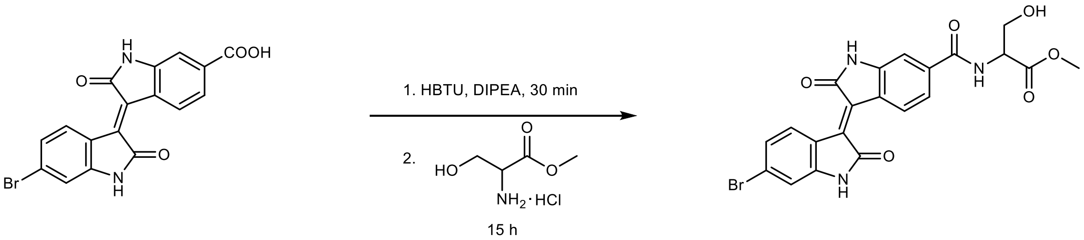

Supplementary Fig. 89 | Synthesis of 6B6AIIdSer.

To a mixture of 6B6CIId (2.31 g, 6 mmol) and 2-(1*H*-benzotriazol-1-yl)-1,1,3,3-tetramethyluronium hexafluorophosphate (HBTU) (2.46 g, 6.48 mmol) in anhydrous DMF (30 ml) was added *N*,*N*-diisopropylethylamine (DIPEA) (3.15 ml, 18 mmol) dropwisely. The mixture was stirred in Ar for 30 min before L-serine methyl ester hydrochloride (1.16 g, 6.48 mmol) in DMF (5 ml) was added. The reaction was monitored by TLC and stirred for another 15 h at room temperature. Upon completion of the reaction, the dark solution was poured onto a saturated $NaHCO_3$ aqueous solution (300 ml). The precipitate was collected by filtration, washed repeatedly with saturated $NaHCO_3$ (aq), water, 0.2 M HCl (aq), cold methanol and dichloromethane, and dried in vacuo to give the pure solid product in dark red (2.14 g, 73%). ^{1}H NMR (400 MHz, DMSO) δ 11.16 (s, 1H), 11.09 (s, 1H), 9.11 (d, *J* = 8.4 Hz, 1H), 9.02 (d, *J* = 8.7 Hz, 1H), 8.71 (d, *J* = 7.4 Hz, 1H), 7.49 (dd, *J* = 8.4, 1.6 Hz, 1H), 7.30 (d, *J* = 1.4 Hz, 1H), 7.20 (dd, *J* = 8.6, 2.0 Hz, 1H), 7.01 (d, *J* = 1.9 Hz, 1H), 5.07 (t, *J* = 6.1 Hz, 1H), 4.53 (dd, *J* = 12.7, 5.5 Hz, 1H), 3.81 (s, 2H), 3.66 (s, 3H). ^{13}C NMR (101 MHz, DMSO) δ 170.92, 168.97, 168.66, 165.82, 145.71, 144.12, 137.01, 133.48, 132.90, 131.10, 129.02, 125.97, 124.11, 123.98, 120.72, 120.35, 112.38, 108.46, 60.92, 55.70, 51.90. Low-res ESI-MS (m/z): $[M-H]^-$ calcd. for $C_{21}H_{16}N_3O_6$, 484; found, 484.

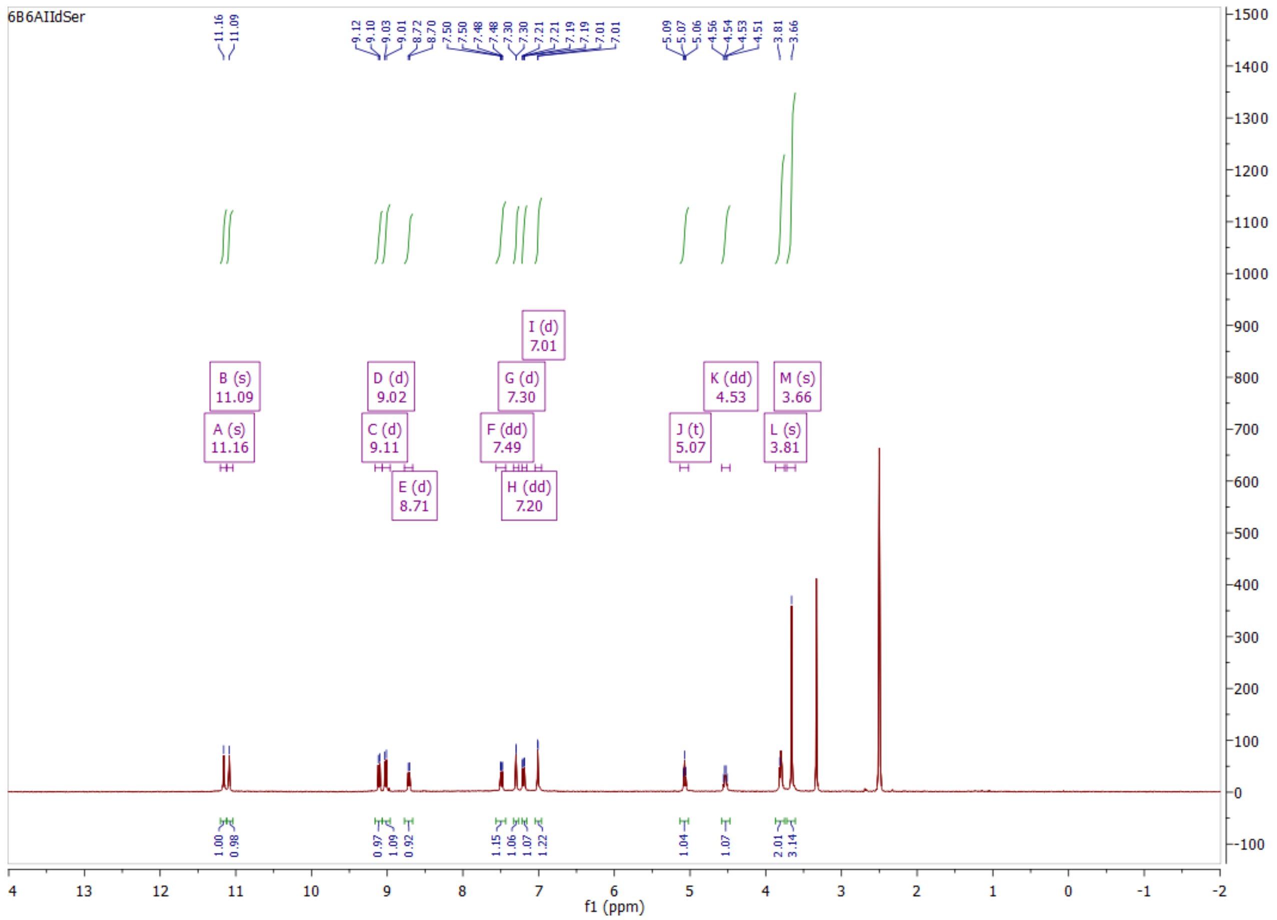

Supplementary Fig. 90 | ^{1}H NMR spectra of 6B6AIIdSer.

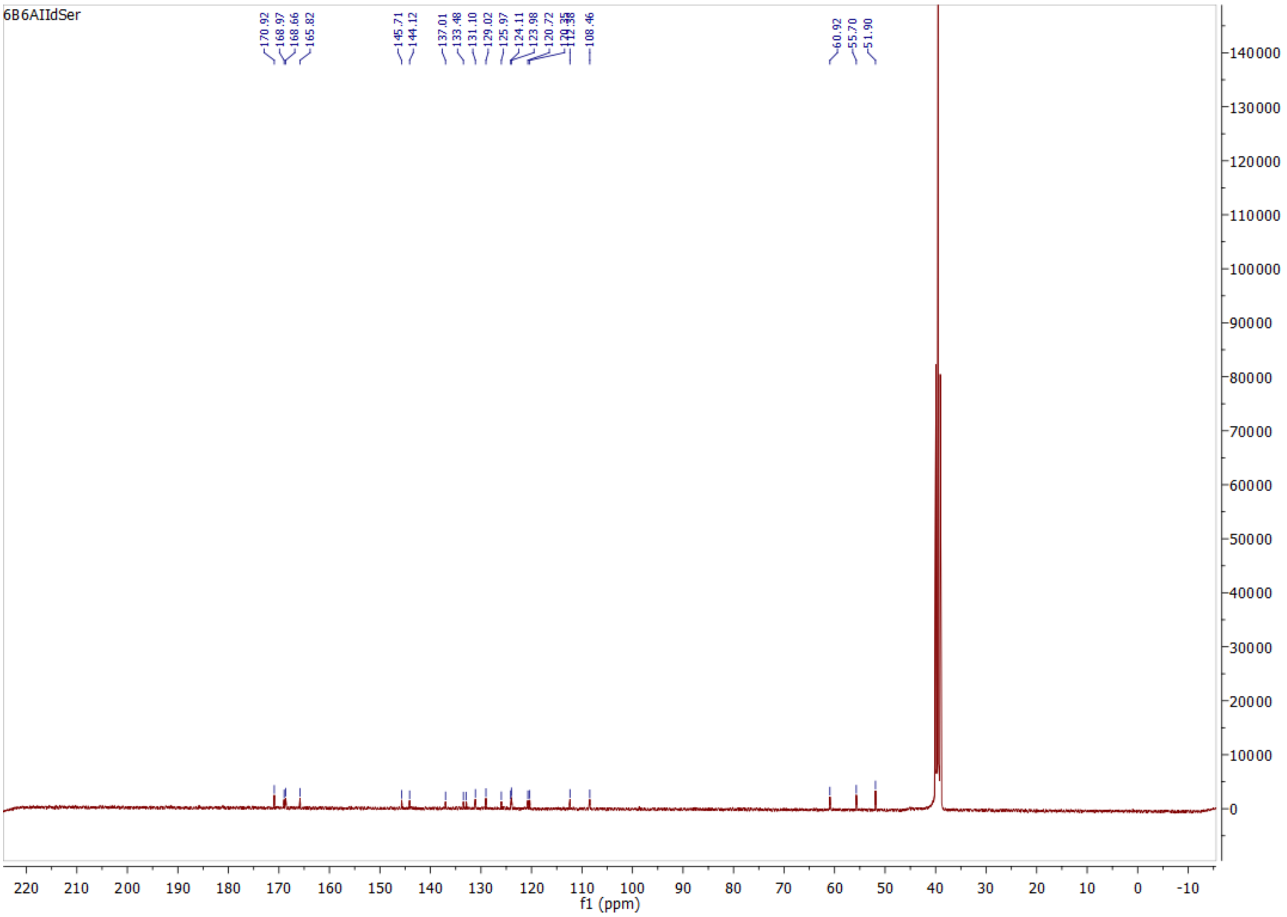

Supplementary Fig. 91 | ^{13}C NMR spectra of 6B6AIIdSer.

_N,N'_-Dipropyl-6,6'-dibromoisoindigo (NNDPr66DBIId):

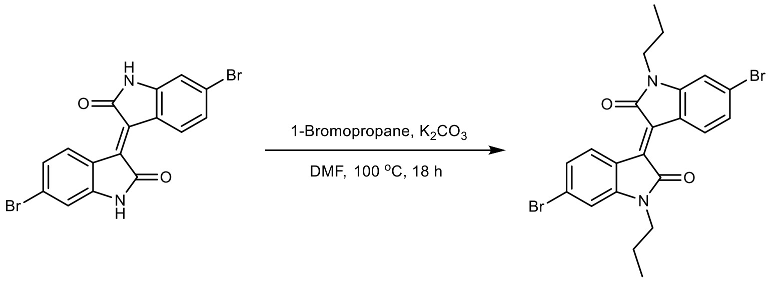

Supplementary Fig. 92 | Synthesis of NNDPr66DBIId.

To a mixture of 66DBIId (158 mg, 0.377 mmol) and K_2CO_3 (261 mg, 1.89 mmol) in DMF (5 ml) under Ar was added propyl bromide (0.2 ml). The mixture was heated at 100 °C under Ar for 18 h. The reaction mixture was cooled and evaporated to dryness in vacuo. The crude solid was partition between dichloromethane and water. The organic phase was dried over Na_2SO_4, filtered, and dried in vacuo. The crude product was then purified by column chromatography on silica gel (230 ~ 400 mesh, hexane/CH_2Cl_2 =1:3) to give a red colour solid (104 mg, 55%). ^{1}H NMR (400 MHz, DMSO) δ 9.05 (d, J = 8.6 Hz, 2H), 7.41 (d, J = 1.9 Hz, 2H), 7.25 (dd, J = 8.6, 1.9 Hz, 2H), 3.74 (t, J = 7.0 Hz, 4H), 1.62 (dd, J = 14.6, 7.6 Hz, 4H), 0.90 (t, J = 7.4 Hz, 6H).

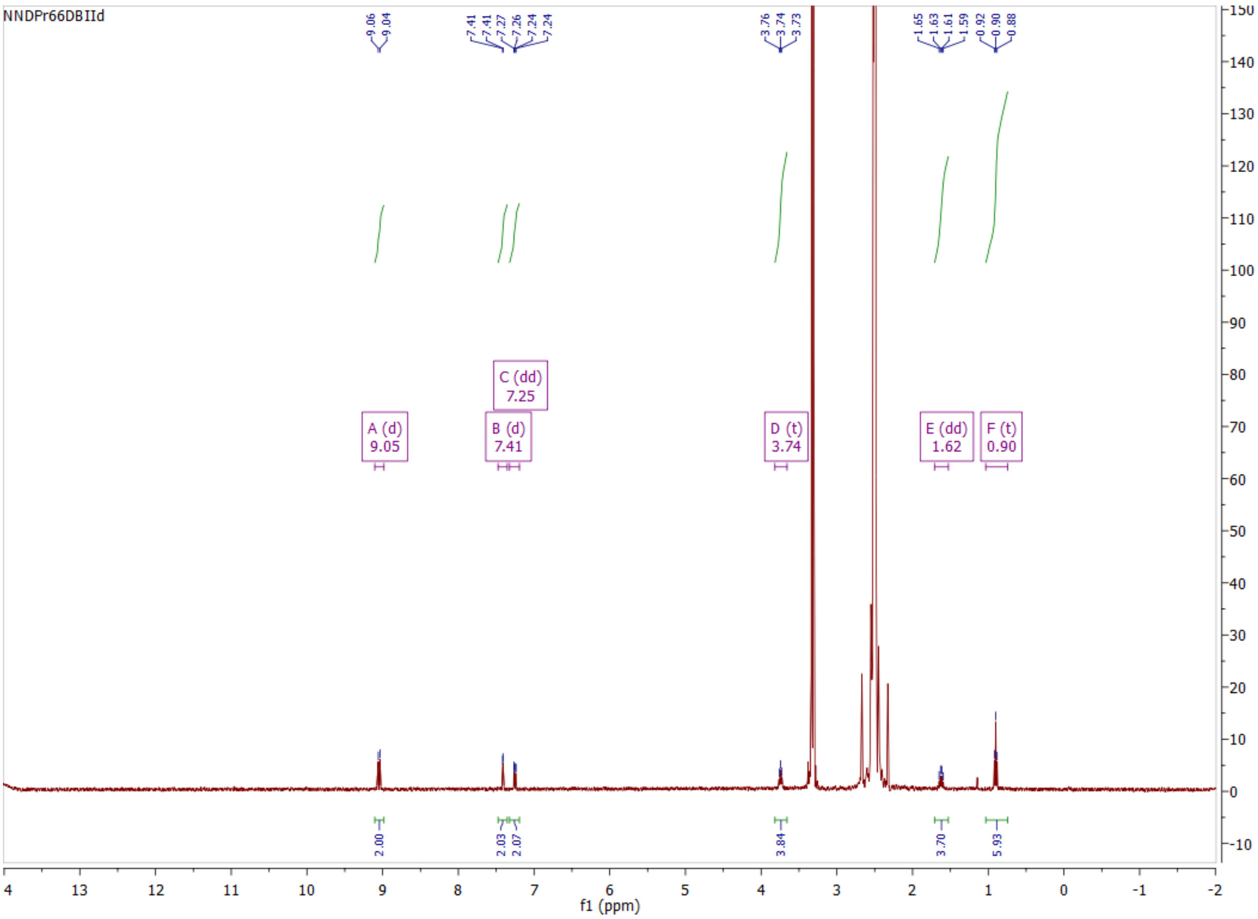

Supplementary Fig. 93 | ^{1}H NMR spectra of NNDPr66DBIId.

N,N'-Di-(2-ethylhexyl)-isoindigo (NNDEHIId):

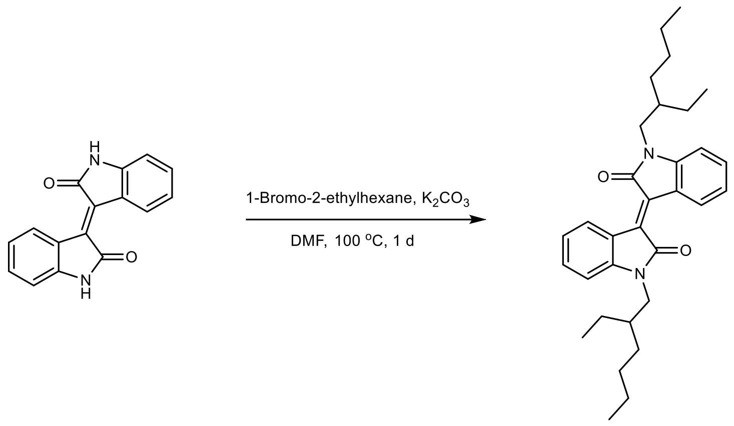

Supplementary Fig. 94 | Synthesis of NNDEHIId.

To a mixture of isoindigo (2.62 g, 10 mmol) and K_2CO_3 (6.9 g, 50 mmol) in DMF (100 ml) was added 1-bromo-2-ethylhexane (3.8 ml, 22 mmol) under Ar atmosphere. The mixture was heated at 100 °C for 1 day. After the reaction was cooled to room temperature, the mixture was poured onto 1 l of water and a dark red precipitate was formed. The solid was filtered, washed with a copious amount of water and cold methanol, and dried in vacuo. The crude product was purified by column chromatography on silica gel (230~400 mesh, hexane/CH_2Cl_2 = 1:1) to give a dark red solid (1.58 g, 32%). ^{1}H NMR (400 MHz, DMSO) δ 9.10 (d, *J* = 7.6 Hz, 2H), 7.44 (t, *J* = 7.7 Hz, 2H), 7.03 (dd, *J* = 17.1, 8.0 Hz, 4H), 3.66 (d, *J* = 7.6 Hz, 4H), 2.40 (d, *J* = 10.6 Hz, 4H), 1.82 (s, 2H), 1.44 – 1.07 (m, 14H), 0.86 (dt, *J* = 13.8, 7.2 Hz, 10H).

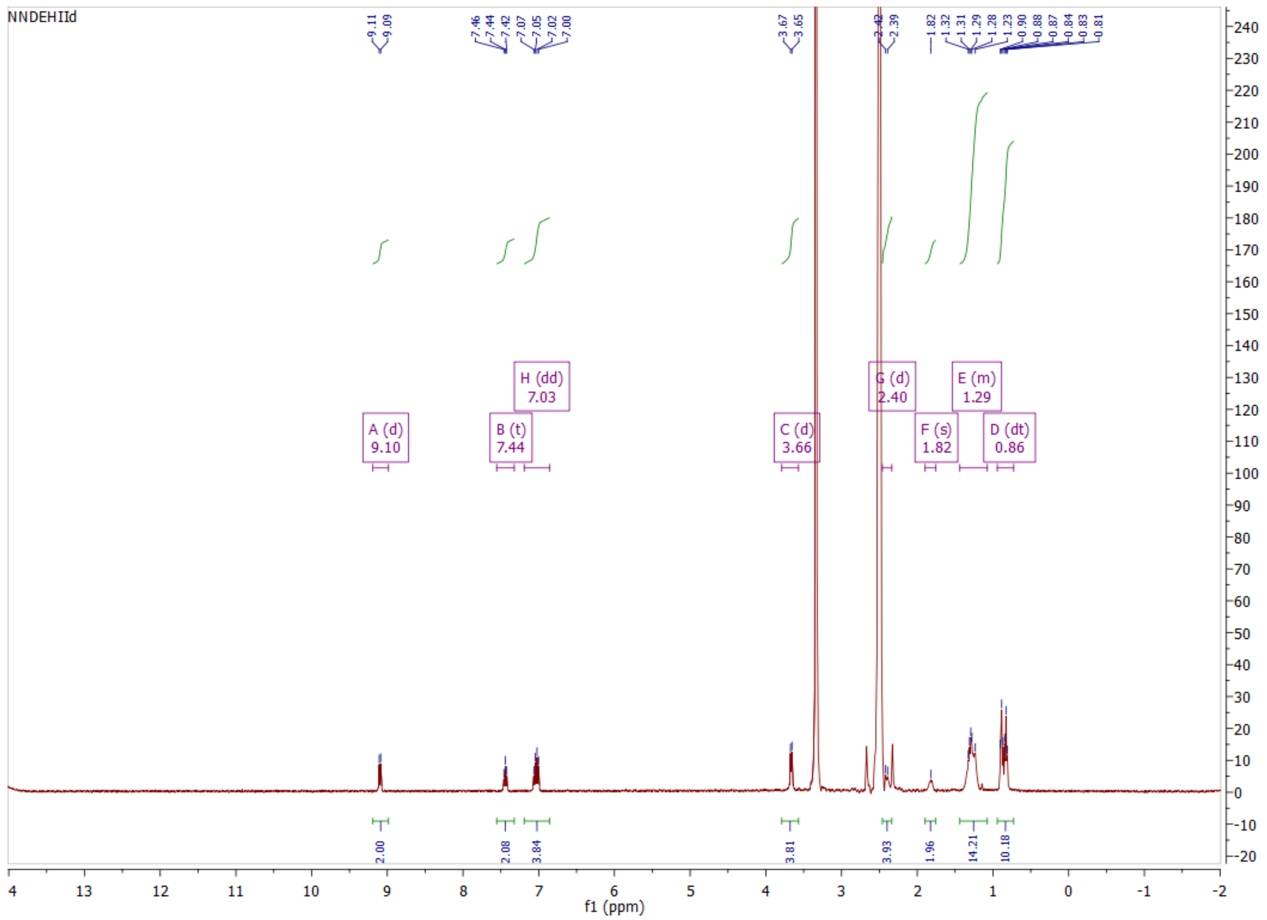

Supplementary Fig. 95 | ^{1}H NMR spectra of NNDEHIId.

Bulk electrolysis

IId^{2-} and IId-CO_2 adduct were synthesised by constant current bulk electrolysis of IId in DMSO-d_6 under N_2 or CO_2 atmosphere. The setup consisted of a three-neck round-bottom flask for the working, counter, and reference electrodes, respectively. Carbon felt (CT GF030; Fuel Cell Store) was used as the working electrode and a silver wire was used as the reference electrode. The counter electrode was separated from the isoindigo solution with a fritted electrode chamber (MR-1196; Bioanalytical Systems). A piece of carbon felt coated with $LiFePO_4$ (50 mg) was used as the counter electrode, which was immersed in a neat electrolyte (1 ml) without isoindigo. $LiClO_4$ (250 mM) in DMSO-d_6 was used as the electrolyte. In a standard condition, IId (26.2 mg, 0.1 mmol) was stirred and reduced at a constant current of 1.5 mA in DMSO-d_6 (2.5 ml, 250 mM $LiClO_4$) under continuous N_2 or CO_2 bubbling. The product was used for NMR analysis without further purification.

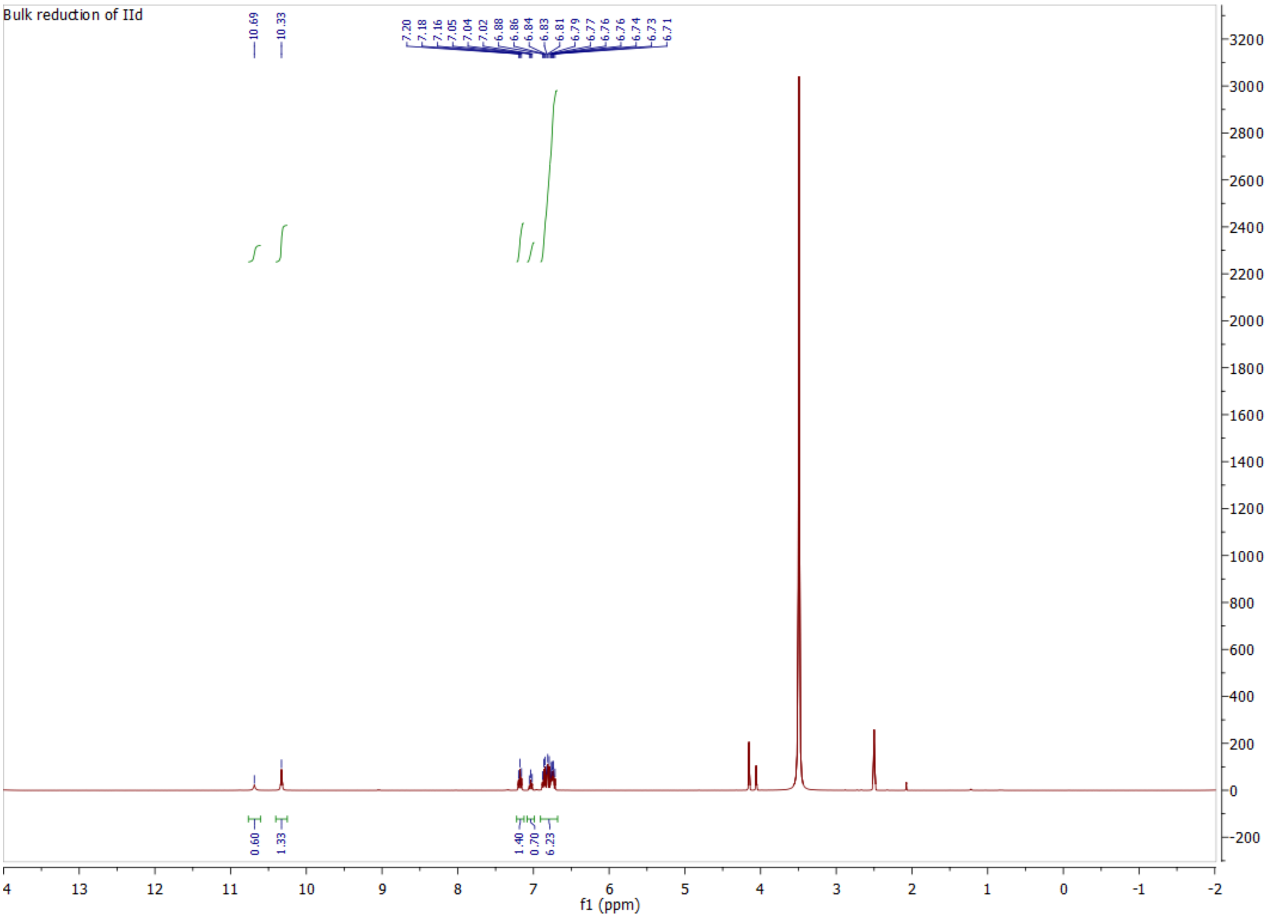

Supplementary Fig. 96 | ^{1}H NMR spectra of the crude solution after bulk reduction of IId.

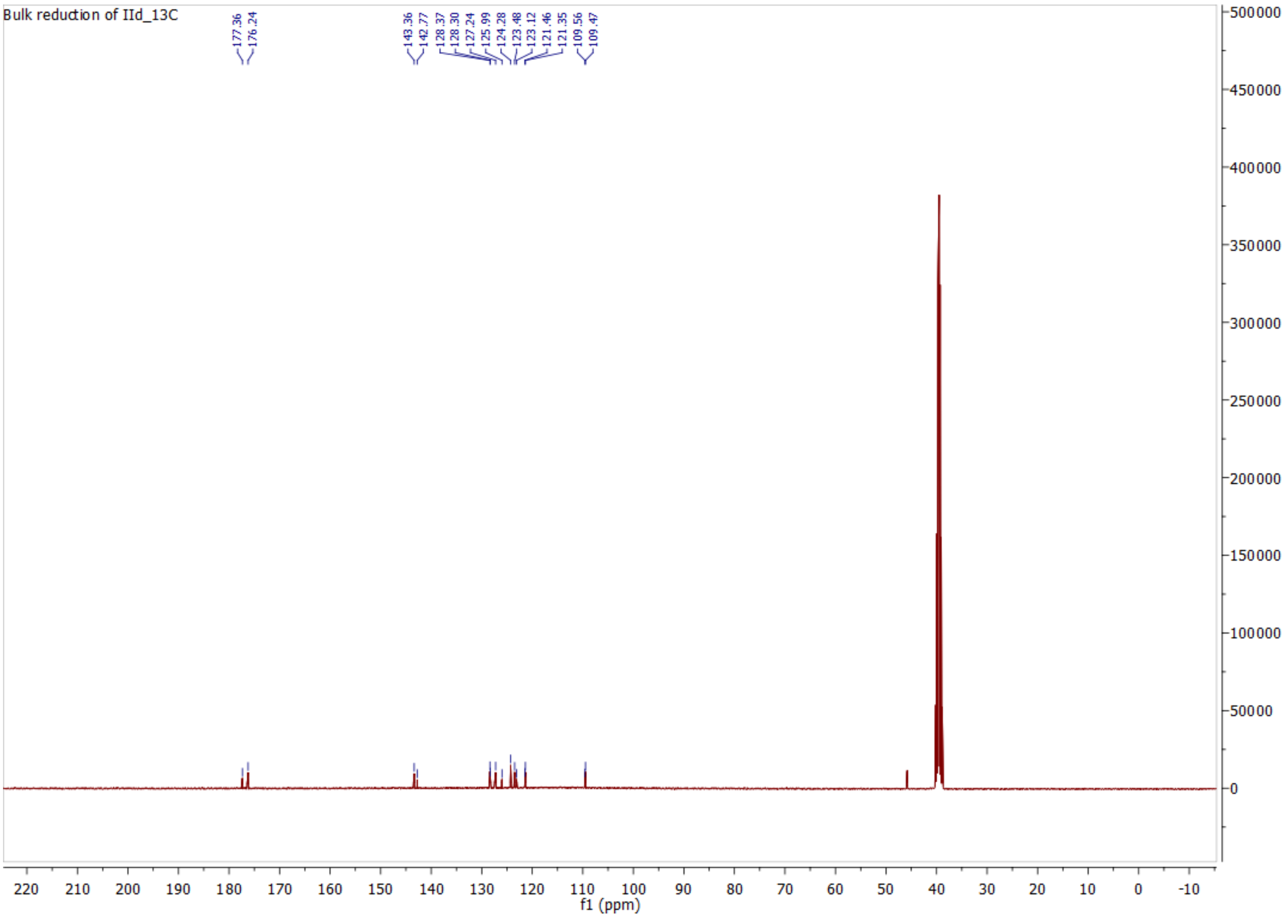

Supplementary Fig. 97 | ^{13}C NMR spectra of the crude solution after bulk reduction of IId.

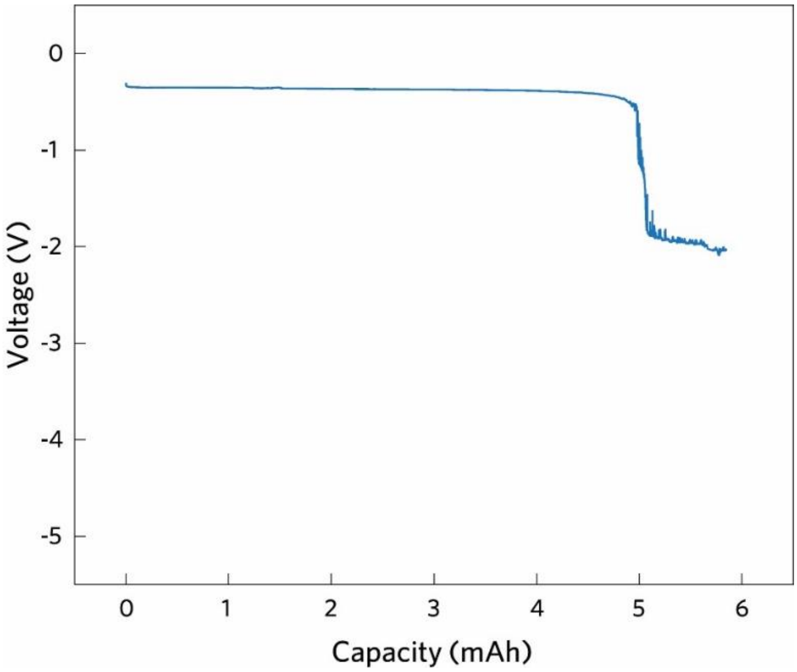

Supplementary Fig. 98 | Potential-capacity curve of the bulk electrolytic reduction of IId.

Nafion membrane pretreatment

The Nafion membranes (Nafion 115; Ionpower) were pretreated using the following method[2]. The membrane was boiled in 3% hydrogen peroxide for 1 h. The membranes were then boiled in 0.25 M sulfuric acid solution for 1 h and cleaned in boiling deionised water for 30 min (twice). Subsequently, the membranes were boiled in 0.25 M sodium hydroxide solution for 1 h and cleaned in boiling deionised water for 30 min (twice). Finally, the membranes were dried under vacuum at 80 °C for at least 1 d and stored in anhydrous DMF.

DFT calculations

The Gaussian16 code[3] was used in the DFT calculations with the B3LYP functional[4,5] and the 6-31++g(d,p) basis set[6-8]. The conductor-like polarizable continuum model (CPCM) was used to simulate the solvation effect of DMF solvent at room temperature[9]. In order to simulate the processes in solvated environment, free energies excluding translational contributions at 298 K are calculated. Benchmark of calculated redox potentials of the first and second electron transfer steps against measured potentials and comparison of functionals can be found in our previous work[2]. Vibrational frequencies were calculated for optimized structures by determining the second derivative of the energy with respect to the nuclear coordinates and then transforming to mass-weighted coordinates.

The equilibrium redox potential of the Lewis base sorbents (abbreviated as B) was calculated as $(G(B_{red}) - G(B_{ox}))/(-|e|)$, where $G(B_{red})$ and $G(B_{ox})$ are the Gibbs free energies of the oxidised and reduced base, respectively. The CO_2 binding energy (E_b) was calculated as $E_b = G_{CO2@B} - G_B - G_{CO2}$, where the first term on the right side of the equation is the Gibbs free energy of CO_2 adsorbed on the sorbent molecule, the second and third terms are the Gibbs energies of the isolated B (any charge state) and CO_2, respectively.

Cell culture

NIH3T3/GFP fibroblast cell line was kindly provided by Dr. Yun Chen at Johns Hopkins University and cultured in Dulbecco’s Modified Eagle’s Medium (DMEM; Gibco) supplemented with 10% fetal bovine serum (FBS; Hyclone) and 1% penicillin/streptomycin (Pen/Strep; Gibco). U2OS.EGFP cells were kindly provided by Dr. J Keith Joung at Massachusetts General Hospital and cultured in DMEM supplemented with 10% FBS, 2 mM GlutaMAX (Gibco), and 1% Pen/Strep. MCF10A epithelial cell line was kindly provided by Dr. Konstantinos Konstantopoulos at Johns Hopkins University and cultured in DMEM/F-12 (1:1) supplemented with 5% New Zealand horse serum (Gibco), 20 ng ml^{-1} EGF (PeproTech), 0.5 μg ml^{-1} hydrocortisone (Sigma Aldrich), 100 ng ml^{-1} cholera toxin (Sigma Aldrich), 10 μg ml^{-1} insulin (Gibco), and 1% Pen/Strep. All cells were cultured in a humidified incubator at 5% CO_2 and 37 °C. All cell lines were tested negative for mycoplasma.

Cytotoxicity assay and cell morphology

Cell cytotoxicity was examined by alamarBlue HS Cell Viability Reagent (Invitrogen) according to manufacturer’s protocol[10,11]. Briefly, cells were plated in 96 wellplates (Falcon) at a density of 20,000 per well for overnight. Testing chemicals dissolved in DMF were sterilised by passing through 0.2 μm DMSO-safe Acrodisc syringe filters (Pall Laboratory) and then added into culture medium at predetermined concentrations. All testing conditions contained 1% DMF as the solvent background. The cells were incubated with the chemicals for 48 hours, washed once with Dulbecco′s Phosphate Buffered Saline (DPBS; Gibco), and then incubated with fresh culture medium containing alamarBlue reagent for 4 hours in a humidified incubator at 37 °C and 5% CO_2. Absorbance was measured at 570

nm by a SpectraMax microplate reader, with 600 nm as a reference wavelength. The cell viability was calculated by relative absorbance to control groups. Data were presented as mean ± standard deviation (SD). The dose-response curves were fitted using a four-parameter logistic function to calculate the cytotoxic median lethal concentration (LC_{50}). Cell morphology was monitored throughout the assay and images were acquired with an EVOS cell imaging system (Thermo Fisher).

Supplementary Notes

Supplementary Note 1 | Characterisations of the intramolecular hydrogen bonding

To confirm the formation of intramolecular hydrogen bonding, we conducted Variable-Temperature (VT) ^{1}H NMR experiments by heating the crude solution of **IId** after bulk electrolysis from 20 to 90 °C (**Supplementary Fig. 3**). As the temperature increases, the characteristic peaks at 10.33 and 10.69 ppm broaden and shift upfield, which is consistent with the fact that hydrogen bonding weakens at elevated temperatures. At 90 °C, the peaks almost vanish due to the fast proton exchange, suggesting the breaking of intramolecular hydrogen bonding. Interestingly, when the temperature is cooled back to 30 °C, the NMR spectrum shows the intact **IId-CO_2** adduct, indicating that our structural design, by harnessing intramolecular hydrogen bonding, can effectively stabilise the EMCC intermediate. Besides, the reversible variation of peak integration at 7.18 and 7.04 ppm at different temperatures suggest the presence of rotational isomers. Furthermore, 2D ^{1}H-^{13}C Heteronuclear Single Quantum Coherence (HSQC) NMR was performed to fully understand the assignment of each NMR peak (**Supplementary Fig. 4**). The disappearance of peaks at 10.69 and 10.33 ppm in the 1D spectrum confirms their origination from the amide protons. The aromatic protons and carbons are resolved by the cross peaks in the 2D spectrum. Based on these results, a full assignment of the NMR peaks is given in **Supplementary Fig. 2**.

In addition to NMR, we recorded the Fourier transform infrared (FT-IR) spectra of the crude solution after bulk electrolysis (**Supplementary Fig. 5**). Compared to **IId**, the IR band assigned to C=O stretching (1699 cm^{-1}) vanished, and a new band appeared at 1716 cm^{-1} in **IId-CO_2**, supporting the formation of the carbonate bond. Moreover, the IR band of free carboxylate ion should be more red-shifted (~1600 cm^{-1}) than 1716 cm^{-1} due to the resonance effect. Therefore, the band at 1716 cm^{-1} suggests the formation of carboxylic acid-like species, corroborating the presence of intramolecular hydrogen bonding between the carbonate oxygen and amide hydrogen. Although the structures of rotational isomers are not fully confirmed due to the lack of single crystal structures, our NMR and FT-IR results strongly support the existence of the proposed intramolecular hydrogen bonding.

Supplementary Note 2 | Modular synthesis of isoindigos

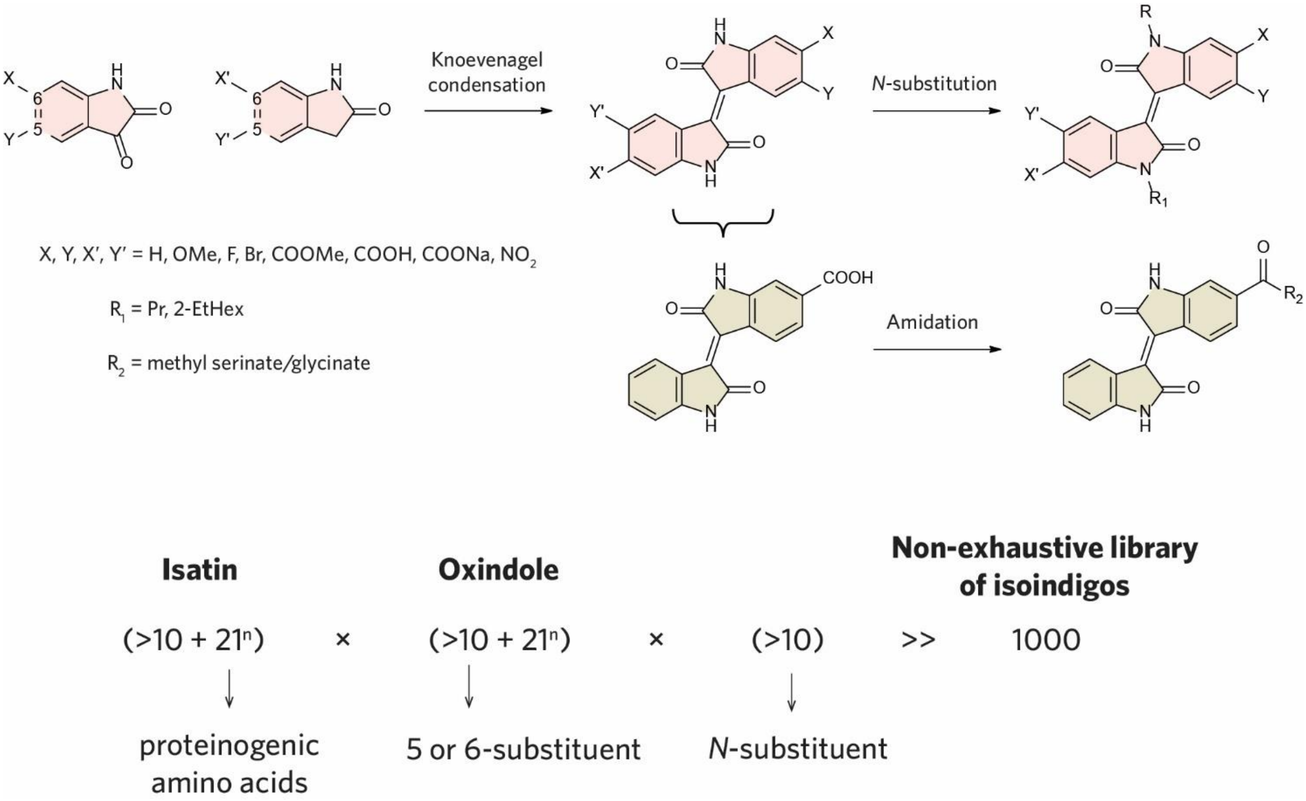

Supplementary Fig. 99 | Modular design and synthesis of isoindigo-based redox-active CO_2 carriers.

Through Knoevenagel condensation between 2-oxindoles and isatins, electron-donating or withdrawing groups (EDGs or EWGs) can be easily encoded into the aromatic systems of isoindigos at 5 or 6-position (**Supplementary Fig. 99**). The *N*-substituent groups can be introduced subsequently via S_N2 reactions. This ease in the synthesis and modification of isoindigo moieties allows us to quickly build a vast library of EMCC sorbent candidates with 21 examples in total, where the redox potential, CO_2 binding constant, and solubility can be easily tuned via molecular engineering. Specifically, we explored a variety of substituents including electron-donating methoxy and electron-withdrawing fluoro, bromo, carboxyl, methyl carboxylate, amide, and nitro groups at 5 or 6-position of isoindigo to study the electronic and steric impact on their redox potentials and CO_2 affinities. Besides, *N*-substituents with aliphatic chains have also been probed to verify the role of intramolecular hydrogen bonding in CO_2 complexation. This design gives rise to a non-exhaustive library of isoindigos.

Supplementary Note 3 | CO_2 binding constant and reaction rate constant

The CO_2 binding constant (K_{CO_2}) was calculated by Eq 1:

$$K_{CO_2} = \frac{\exp\left(\frac{F}{RT}\Delta E_{peak}(2)\right)-1}{[CO_2]} \quad \text{(Eq 1)}$$

where F is the Faraday constant, R is the ideal gas constant, T is the temperature, $\Delta E_{peak}(2)$ is the difference between the second reduction CV peak potentials ($IId^{\bullet-}/IId^{2-}$) in CO_2 and N_2 atmospheres (**Supplementary Fig. 7** and **8**), and $[CO_2]$ is the concentration of dissolved CO_2 (0.198 M in DMF under 100% CO_2 headspace at 298 K).

In electrochemistry, the *Cottrell equation* describes the current response (i) when the potential is a step function in time (t):

$$i = nAFC_0^*\left(\frac{D}{\pi t}\right)^{\frac{1}{2}} \quad \text{(Eq 2)}$$

where i is current, n is the number of electrons transferred per molecule, A is the area of the electrode, C_0^* is the bulk concentration of the redox-active isoindigo molecule, D is the diffusion coefficient, and t is time. The expected current for a diffusion limited electrochemical process following an ECE (electron transfer, chemical reaction, electron transfer) mechanism at a plane electrode under diffusion-controlled conditions is:

$$i = AFC_0^*\left(\frac{D}{\pi t}\right)^{\frac{1}{2}}[n_1 + n_2(1 - e^{-k_f t})] \quad \text{(Eq 3)}$$

where n_1 and n_2 are the number of electrons transferred in the first and second step, respectively, and k_f is the forward reaction rate constant of the chemical reaction. Here, to calculate k_f after the first electron transfer, n_2 and n_1 are set to 1. Eq 3 then becomes:

$$i = AFC_0^*\left(\frac{D}{\pi t}\right)^{\frac{1}{2}}(2 - e^{-k_f t}) \quad \text{(Eq 4)}$$

The integrated Cottrell equation relates total charge Q with time for chronoamperometry:

$$Q = 2nAFD^{\frac{1}{2}}C_0^*\pi^{-\frac{1}{2}}t^{\frac{1}{2}} \quad \text{(Eq 5)}$$

Then, the constant k can be calculated, here k is assumed constant:

$$k = nAFD^{\frac{1}{2}}C_0^*\pi^{-\frac{1}{2}} \quad \text{(Eq 6)}$$

where n is the number of electrons reduced per molecule.

To calculate k_f, the constant k is calculated using Eq 5 using potentiostatic measurements. Plugging in Eq 6, and rearranging Eq 4 gives:

$$k_f\, t = -\ln\left(2 - \frac{it^{\frac{1}{2}}}{k}\right) \quad \text{(Eq 7)}$$

The data for determining k_f is shown in **Supplementary Figs. 16 to 19** using 5 mM sorbent molecules in 0.1 M NBu_4PF_6 DMF electrolyte. The value k was determined using data under N_2; k_f was obtained under 1 bar 20% CO_2 (0.0396 M CO_2 in solution). The bimolecular reaction rate constant $k_{bimolecular}$ was determined using Eq 8 and summarised in **Supplementary Table 2**.

$$k_{bimolecular} = k_f / [CO_2] \quad \text{(Eq 8)}$$

where $[CO_2]$ = 0.0396 M.

Supplementary Note 4 | CO_2 binding behaviours of 5N6MCIId and 6CIIdNa

We further designed an isoindigo with extremely strong EWG substituents to test the tolerance of the intramolecular hydrogen bonding (***b***) for promoting CO_2 binding. 5N6MCIId, with a nitro substitution at 5-position in one ring and a methyl carboxylate substitution at 6'-position in the other ring, exhibits the most anodically shifted reduction potential under N_2 (−0.93 V vs. Fc^+/Fc) among all isoindigos in this work. Due to the non-symmetrical structure in 5N6MCIId, the strong electron-withdrawing carboxylate and nitro group can work synergistically to frustrate the CO_2 binding in one oxindole ring. The carboxylate group withdraws the electron density from the O atom within the ring connecting it, while the nitro group in the adjacent ring creates steric hindrance and dipole-dipole repulsion to inhibit CO_2 binding (**Supplementary Fig. 21**). In addition, the methyl carboxylate group is also a hydrogen bonding acceptor which may disrupt the intermolecular hydrogen bonding (***b***) between the amide hydrogen and the CO_2 complexed. As a result, the molecule displays a weaker binding with CO_2 compared to the other *N*-unsubstituted isoindigos with a $\log K_{CO_2}$ of 4.62. Nevertheless, this value is qualified for CO_2 capture from flue gas, and the reductive potential required for completing two-electron transfer is −1.15 V vs. Fc^+/Fc, meaning it still can circumvent the oxygen-related side reactions during the EMCC process.

In addition to EDG and EWG substituents, we also study the effect of ionic substituents on the isoindigo moiety. We synthesised an isoindigo (6CIIdNa) bearing sodium carboxylate group at 6-position (Fig. 3). Despite the electron-withdrawing effect of the substituent, 6CIIdNa displays a reduction potential at −1.33 V vs. Fc^+/Fc under N_2 and a CO_2 binding constant of 5.79. The cathodically shifted potential can be explained by the electrostatic repulsion between the negatively charged electrode surface and the anionic 6CIIdNa which creates an energy barrier to reduce the molecule to species with more negative charges. The decreased CO_2 binding affinity is probably caused by the disruption of the intermolecular hydrogen bonding (***b***) between the amide hydrogen and the CO_2 complexed, since the carboxylate anion is a strong hydrogen bonding acceptor.

Supplementary Note 5 | Degradation rate of EMCC prototype based on 6B6AIIdSer

To mitigate the crossover problem, the CO_2 adduct was not fully oxidised back to the neutral sorbent in each CO_2 capture-release cycle on purpose, causing a major loss of the electrochemical capacity. Other sources of decay may originate from many different factors, such as crossover of the redox-active electrode molecules, decomposition of the electrolyte or carbon paper, or degradation of the CO_2 carrier. For 6B6AIIdSer (**Supplementary Fig. 34**), we utilise the energy capacity of the sorbent tank as a reference to estimate the degradation rate using the following equation,

$$r_{\mathrm{d}} = \left(\frac{Q_0 - Q_n}{n \cdot Q_0}\right) * 100\% \qquad \text{(Eq 9)}$$

where r_{d} is the degradation rate, Q_0 is the initial energy capacity of the sorbent tank, Q_n is the last-cycle energy capacity of the sorbent tank, and *n* is the cycle number.

Using –1.3 V as the cut-off potential, the energy capacity of the flow cell decreased to 7 mAh in the last cycle tested. The initial energy capacity of 6B6AIIdSer (0.2 M, 5 ml) is 53.6 mAh. Therefore, the estimated degradation rate is ~2%. Noticeably, the actual degradation rate should be much less if the CO_2 adduct was fully oxidised in each CO_2 capture-release cycle.

Supplementary Note 6 | An alternative method to estimate the CO_2 binding constant

As an alternative method, we also performed CV measurements of isoindigos at different CO_2 concentrations (**Supplementary Fig. 39**). The relationship between $\Delta E_{peak}(2)$ and CO_2 concentration is fitted according to the following equation to determine the CO_2 binding constant:

$$\Delta E_{\mathrm{peak}}(2) = \frac{RT}{zF} \ln\left(1 + K[\mathrm{CO_2}]^n\right) \quad \text{(Eq 10)}$$

where *F* is the Faraday constant, *R* is the ideal gas constant, *T* is the temperature, *z* is the number of electron transferred, $\Delta E_{peak}(2)$ is the difference between the second reduction peak potentials ($IId^{\bullet -}/IId^{2-}$) in CO_2 and N_2 atmospheres, $[CO_2]$ is the concentration of dissolved CO_2 (0.198 M in DMF under 100% CO_2 headspace at 298 K), *n* is the number of binding sites, and *K* is the CO_2 binding constant.

As shown in **Supplementary Fig. 40**, curve fitting gives rise to similar *K* values on the order of 10^{12} for IId and 6MCIId. However, the fitting quality was unsatisfactory (relatively low R^2 values and unreasonable number of binding sites >2), likely due to the limited CO_2 complexation kinetics under low CO_2 concentrations that complicated the thermodynamic analysis.

Supplementary References

1. Simeon, F. *et al.* Electrochemical and molecular assessment of quinones as CO_2-binding redox molecules for carbon capture. *J. Phys. Chem. C* **126**, 1389-1399 (2022).
2. Li, X., Zhao, X., Liu, Y., Hatton, T. A. & Liu, Y. Redox-tunable Lewis bases for electrochemical carbon dioxide capture. *Nat. Energy* **7**, 1065-1075 (2022).
3. Gaussian 16 Rev. C.01 (Wallingford, CT, 2016).
4. Becke, A. D. Density-functional thermochemistry. III. The role of exact exchange. *J. Chem. Phys.* **98**, 5648-5652 (1993).
5. Lee, C., Yang, W. & Parr, R. G. Development of the Colle-Salvetti correlation-energy formula into a functional of the electron density. *Phys. Rev. B* **37**, 785-789 (1988).
6. Clark, T., Chandrasekhar, J., Spitznagel, G. W. & Schleyer, P. V. R. Efficient diffuse function-augmented basis sets for anion calculations. III. The 3-21+G basis set for first-row elements, Li-F. *J. Comput. Chem.* **4**, 294-301 (1983).
7. Hariharan, P. C. & Pople, J. A. The influence of polarization functions on molecular orbital hydrogenation energies. *Theor. Chim. Acta* **28**, 213-222 (1973).
8. Hehre, W. J., Ditchfield, R. & Pople, J. A. Self-consistent molecular orbital methods. XII. Further extensions of gaussian-type basis sets for use in molecular orbital studies of organic molecules. *J. Chem. Phys.* **56**, 2257-2261 (1972).
9. Barone, V. & Cossi, M. Quantum calculation of molecular energies and energy gradients in solution by a conductor solvent model. *J. Phys. Chem. A* **102**, 1995-2001 (1998).
10. Rampersad, S. N. Multiple applications of alamar blue as an indicator of metabolic function and cellular health in cell viability bioassays. *Sensors* **12**, 12347-12360 (2012).
11. Kumar, P., Nagarajan, A. & Uchil, P. D. Analysis of cell viability by the alamarBlue assay. *Cold Spring Harb. Protoc.* **6**, 462-464 (2018).
