# Supplementary material for: Redox-tunable isoindigos for electrochemically mediated carbon capture
Source: Nat Commun. 2024 Feb 8;15:1175. doi: 10.1038/s41467-024-45410-z (PMC10853560; doi:10.1038/s41467-024-45410-z)
Supplement: Supplementary file 4 — Source Data [file 41467_2024_45410_MOESM4_ESM.zip › Source data/HRMS.pptx]

## Slide 1
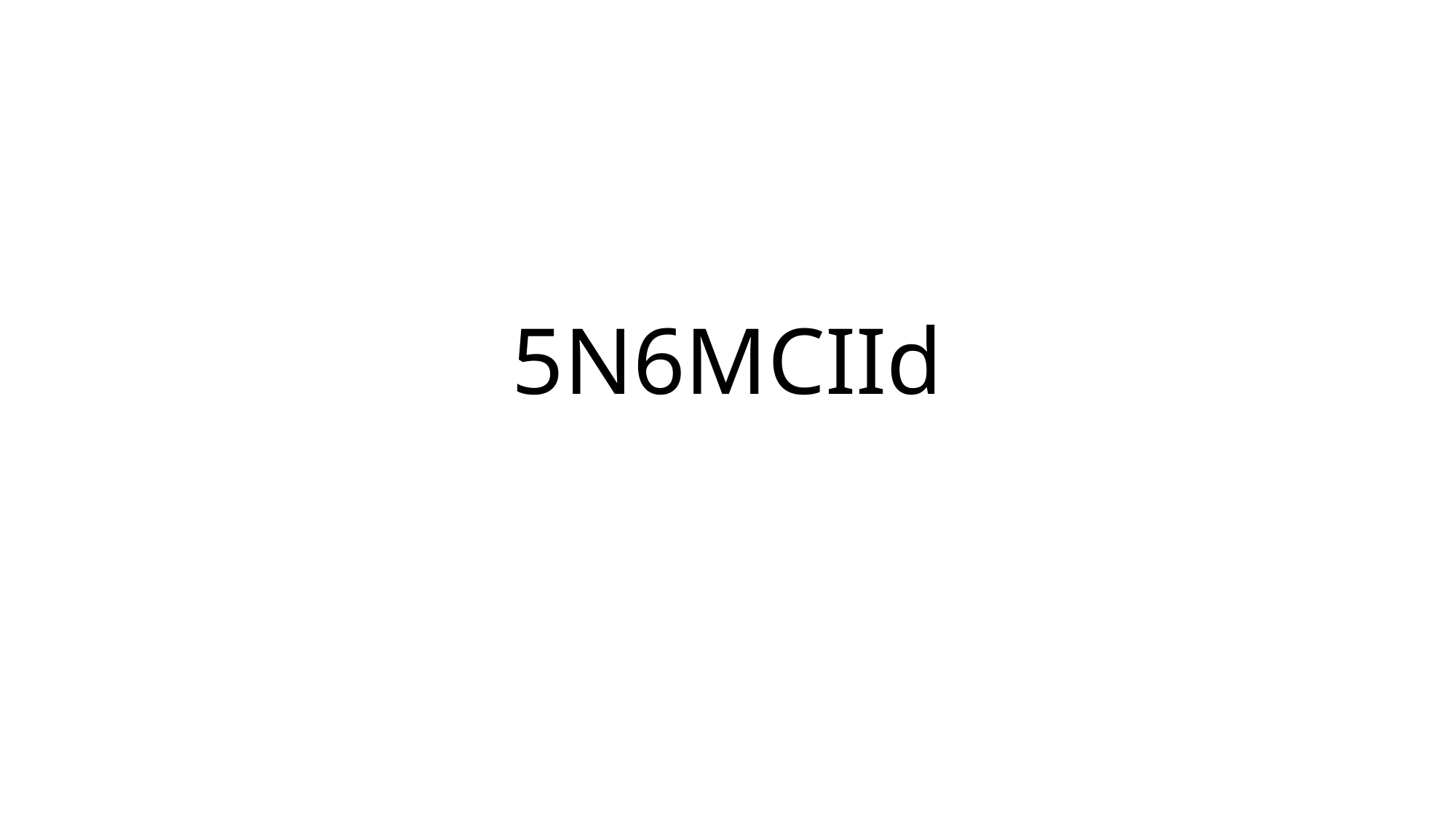

# 5N6MCIId

## Slide 2
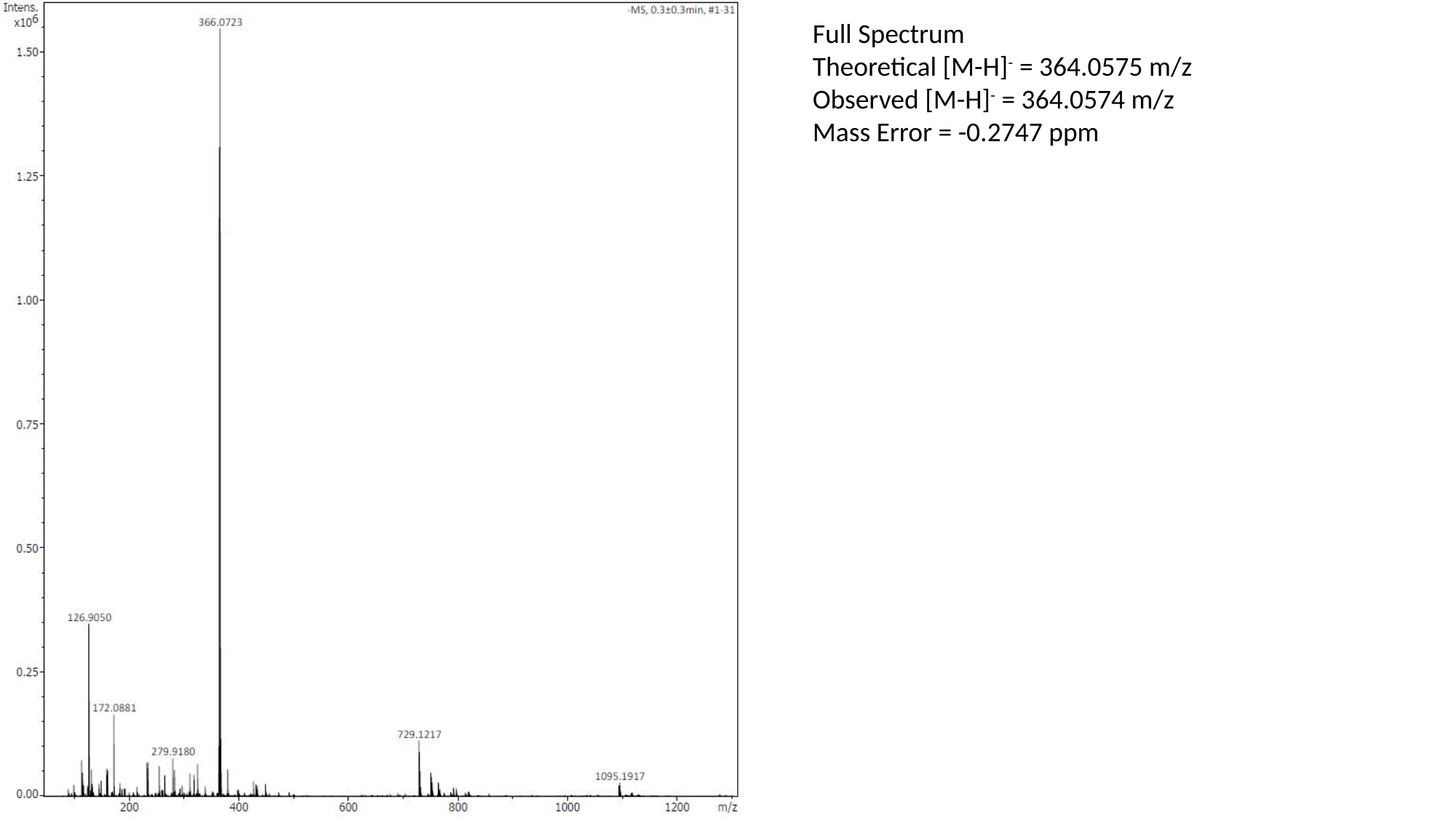

Full Spectrum
Theoretical [M-H]- = 364.0575 m/z
Observed [M-H]- = 364.0574 m/z
Mass Error = -0.2747 ppm

## Slide 3
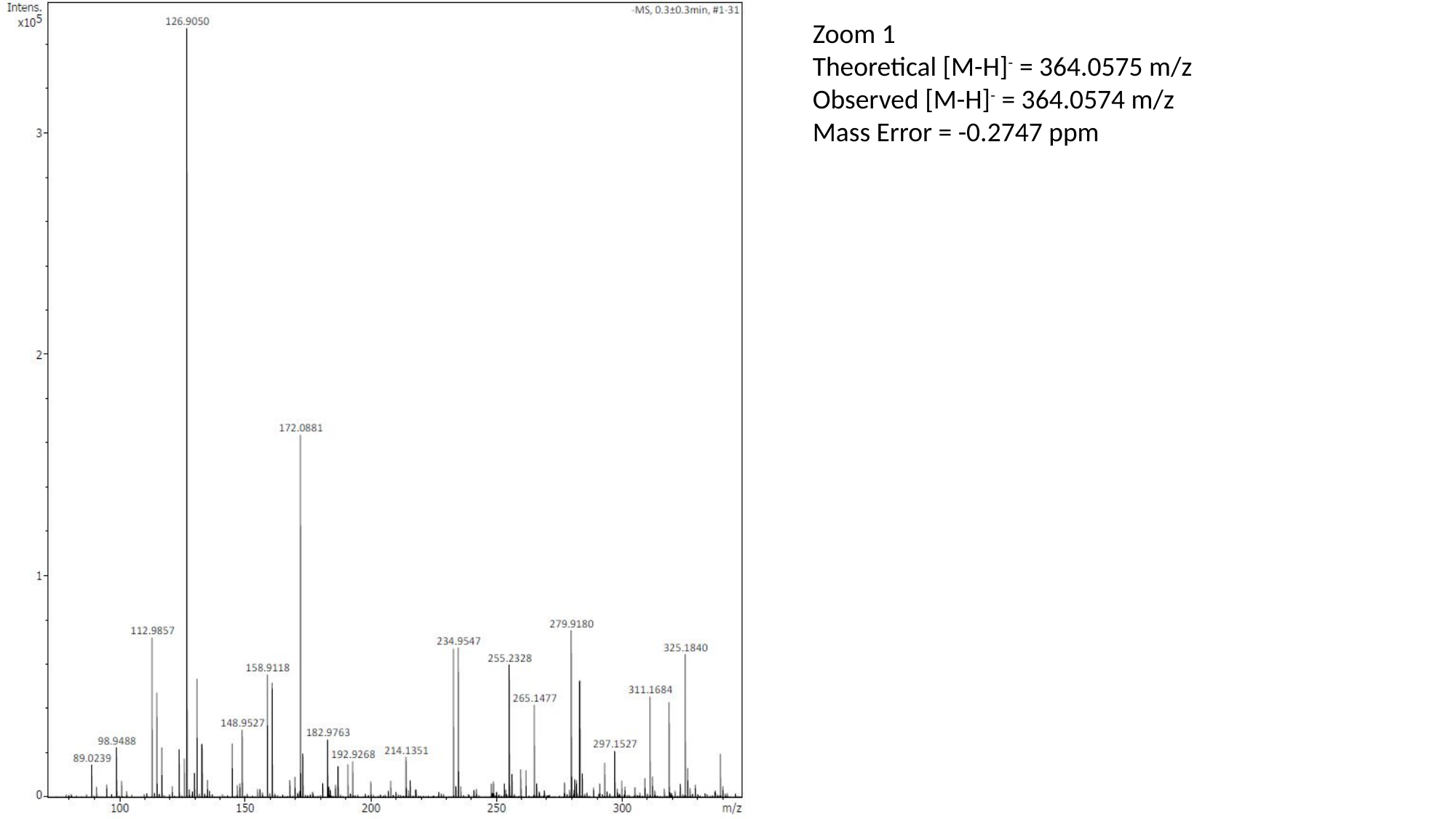

Zoom 1
Theoretical [M-H]- = 364.0575 m/z
Observed [M-H]- = 364.0574 m/z
Mass Error = -0.2747 ppm

## Slide 4
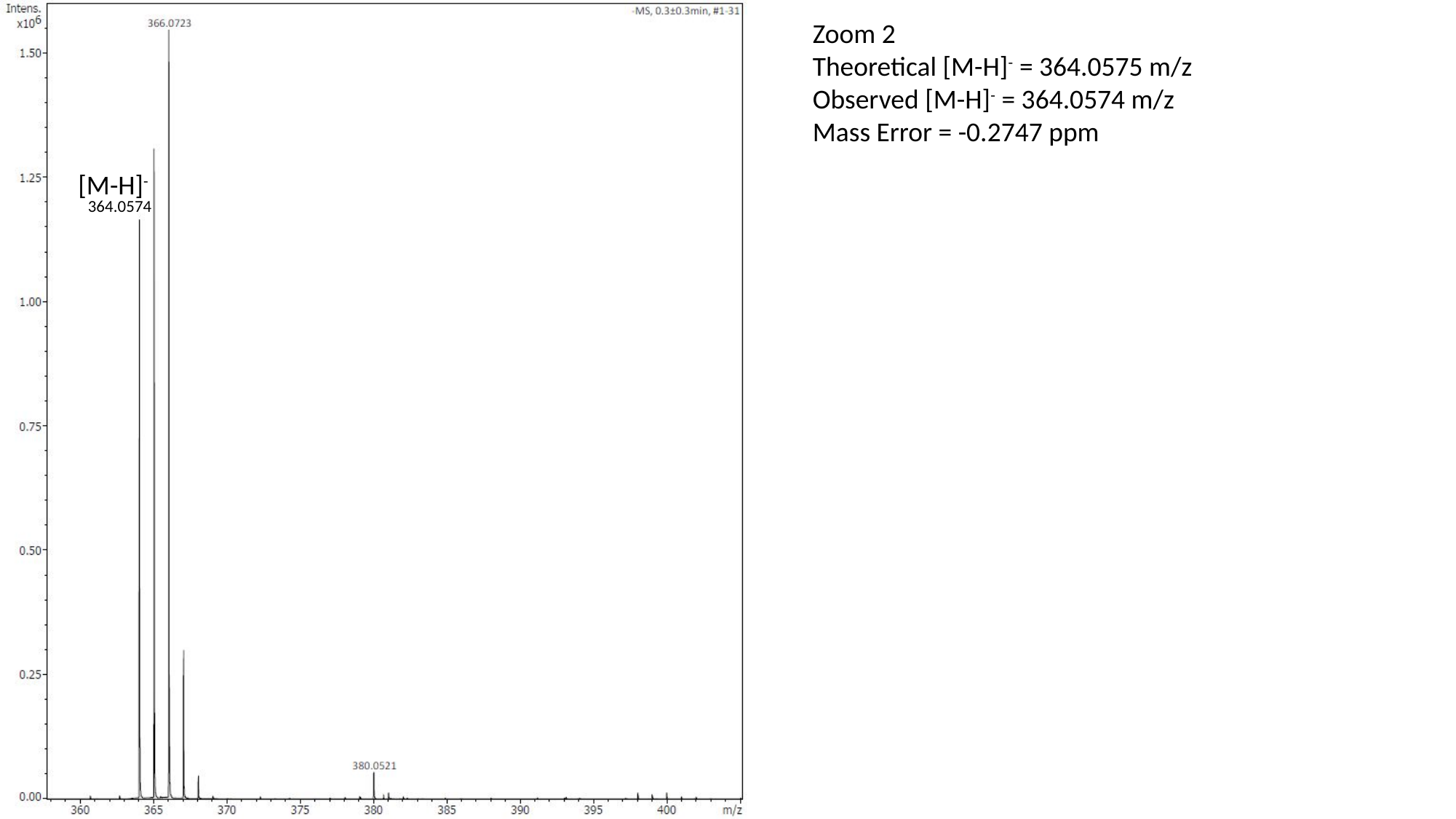

Zoom 2
Theoretical [M-H]- = 364.0575 m/z
Observed [M-H]- = 364.0574 m/z
Mass Error = -0.2747 ppm
[M-H]-
364.0574

## Slide 5
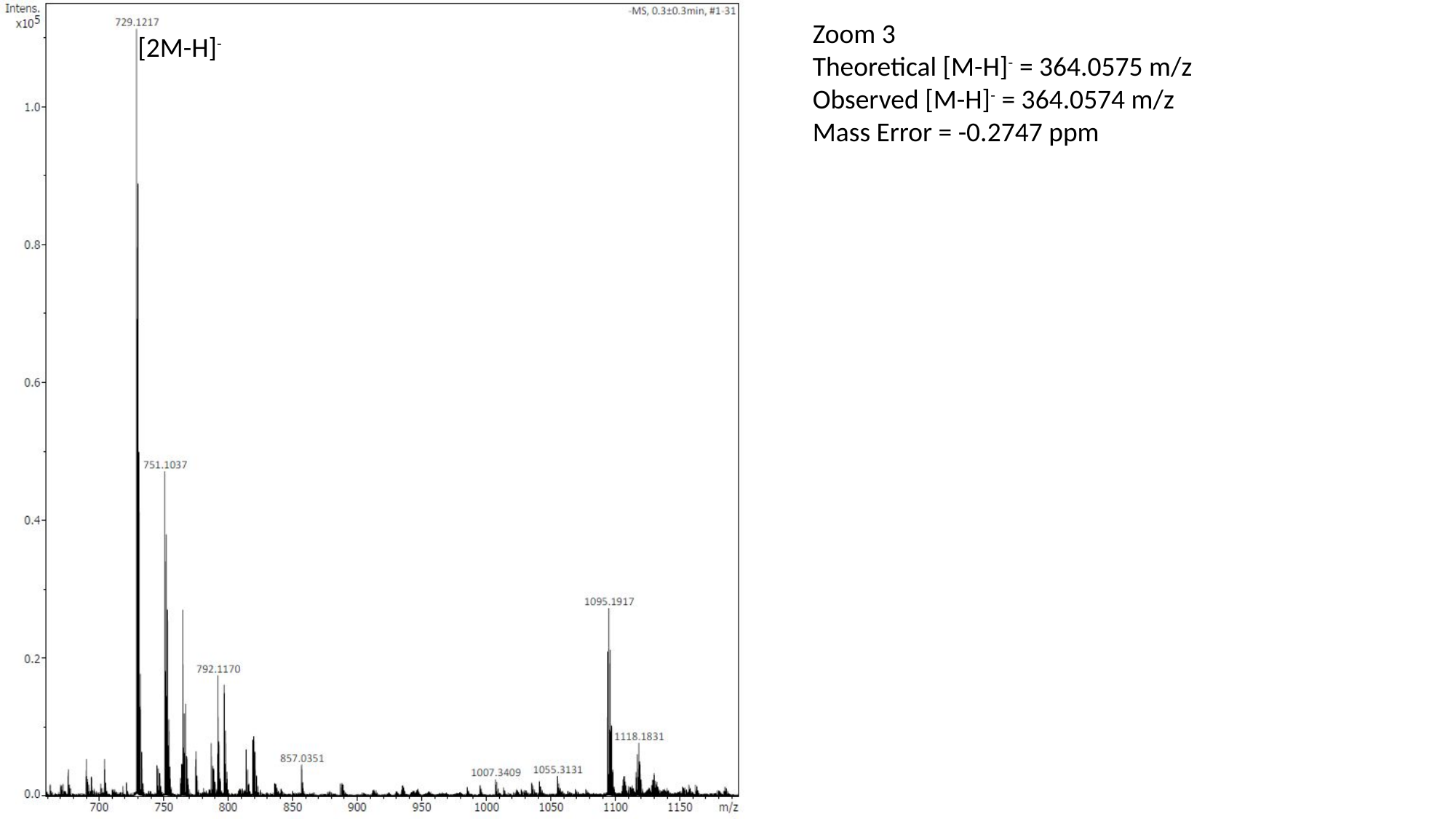

Zoom 3
Theoretical [M-H]- = 364.0575 m/z
Observed [M-H]- = 364.0574 m/z
Mass Error = -0.2747 ppm
[2M-H]-

## Slide 6
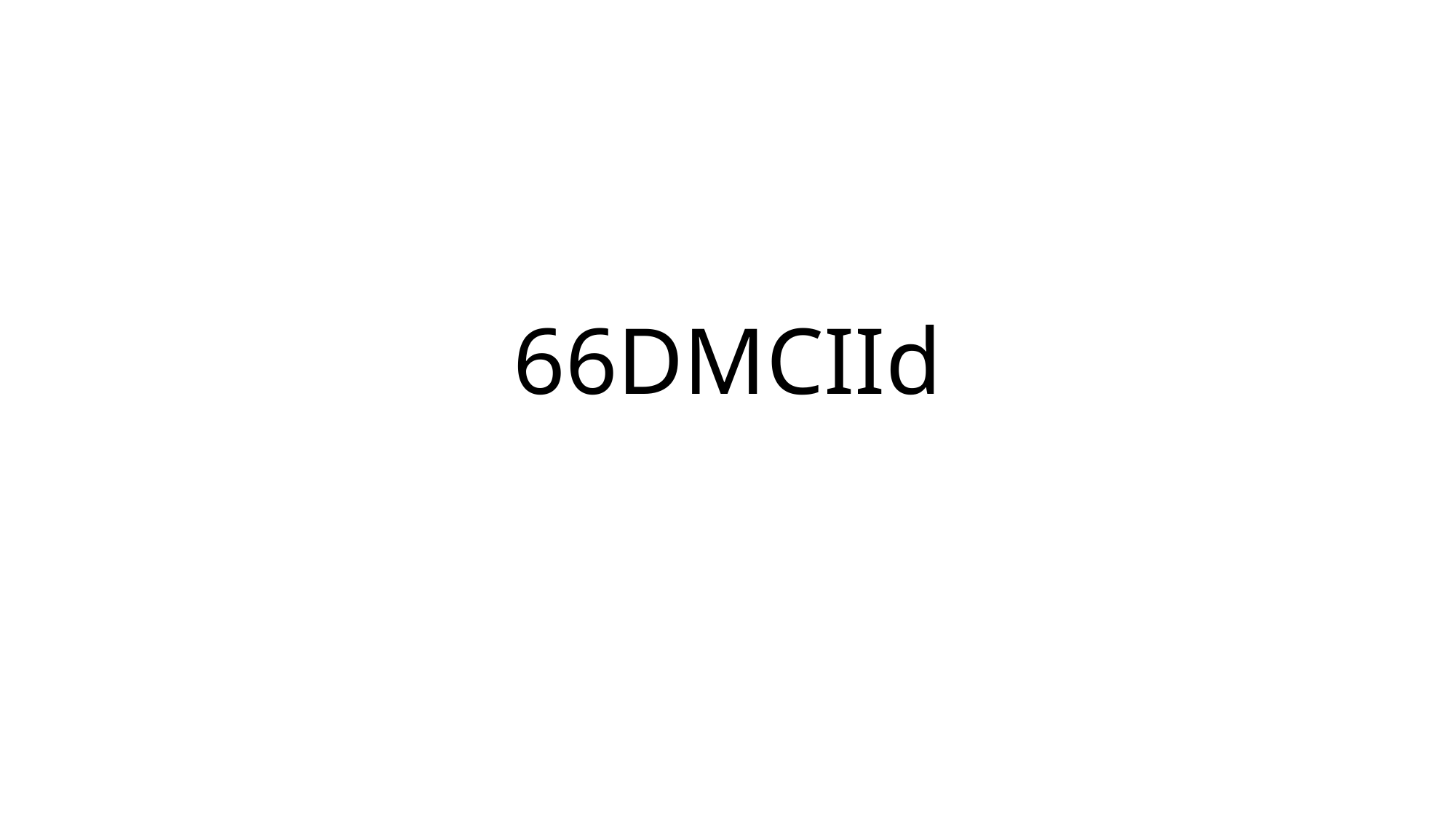

# 66DMCIId

## Slide 7
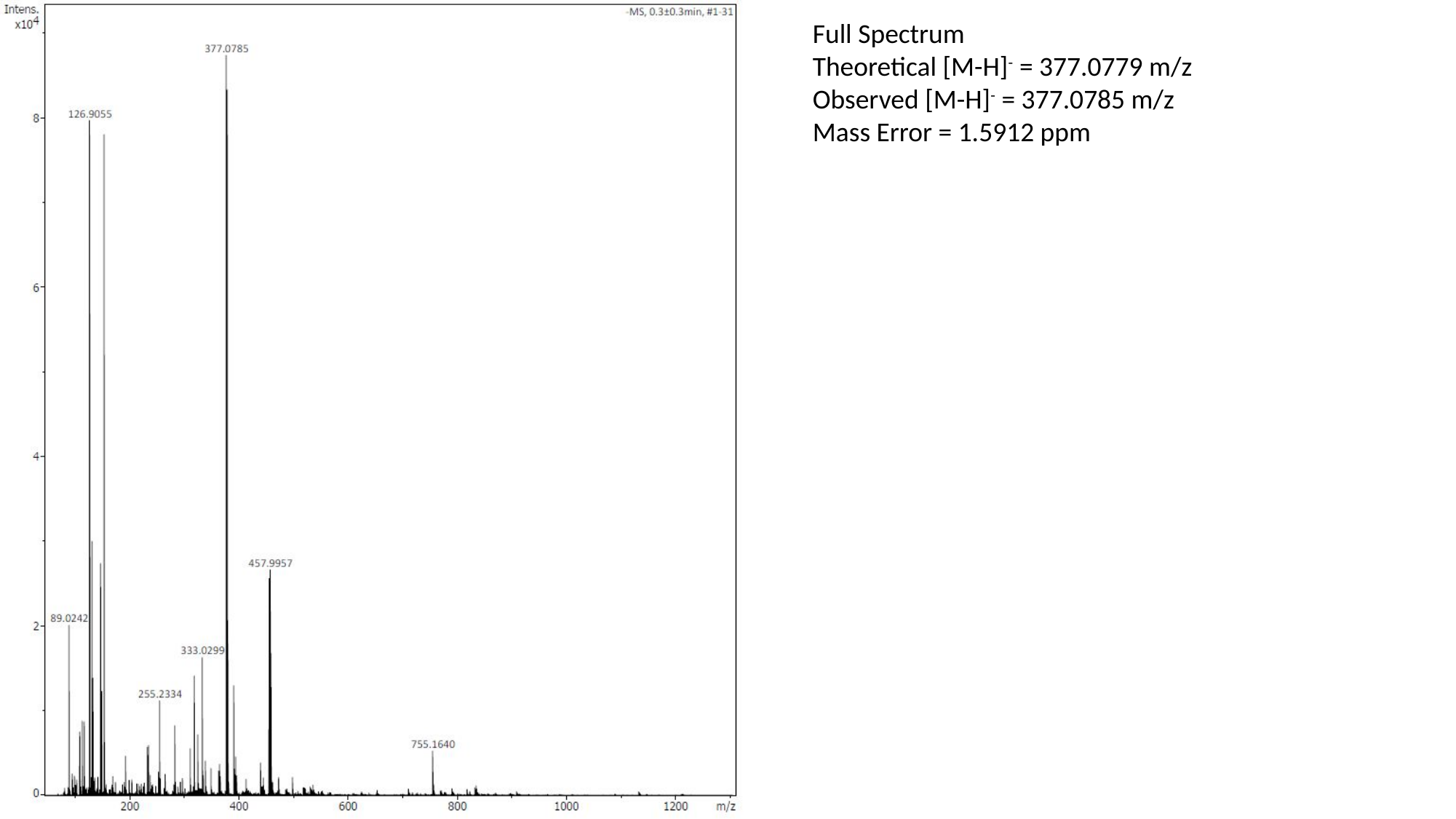

Full Spectrum
Theoretical [M-H]- = 377.0779 m/z
Observed [M-H]- = 377.0785 m/z
Mass Error = 1.5912 ppm

## Slide 8
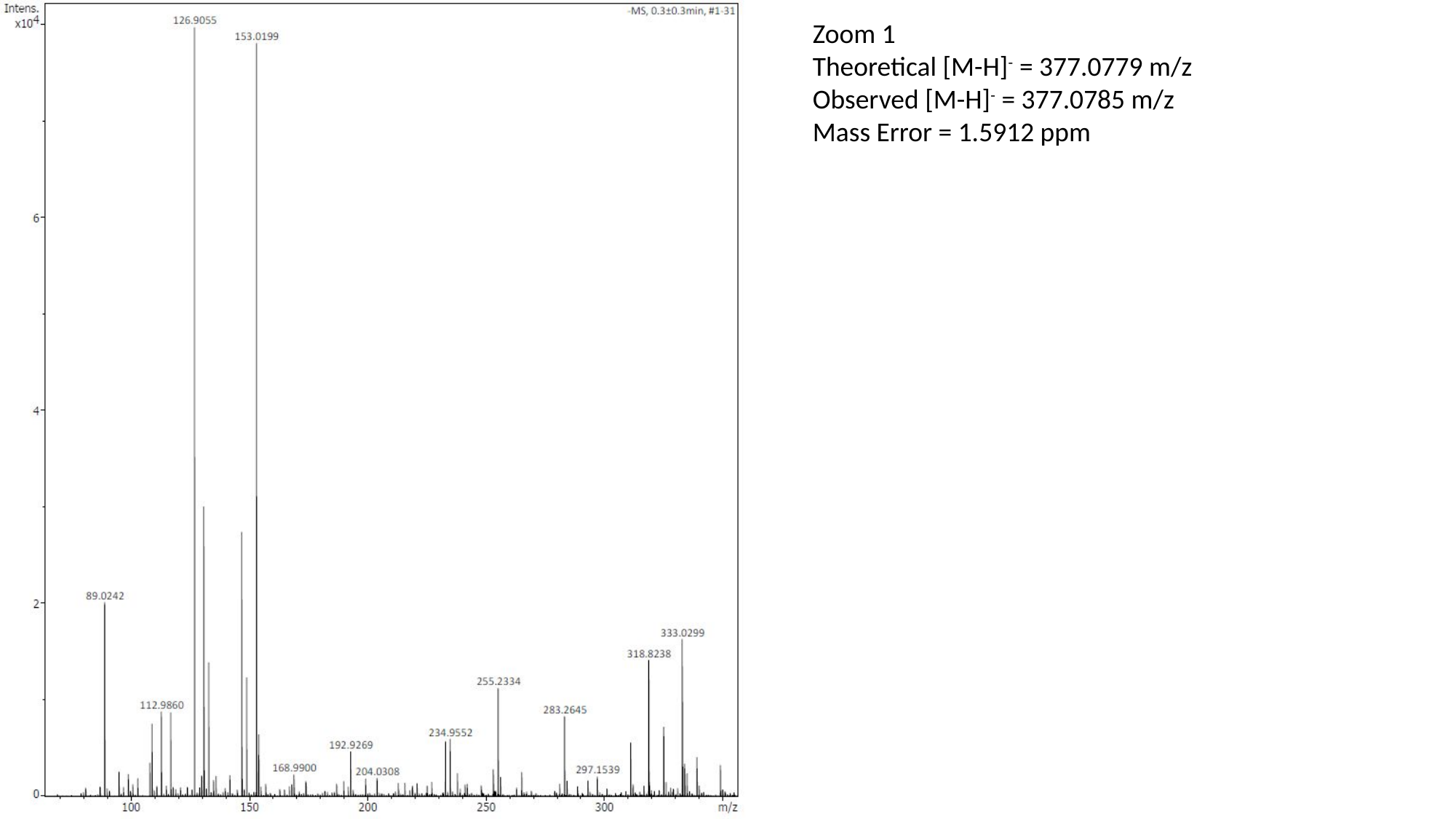

Zoom 1
Theoretical [M-H]- = 377.0779 m/z
Observed [M-H]- = 377.0785 m/z
Mass Error = 1.5912 ppm

## Slide 9
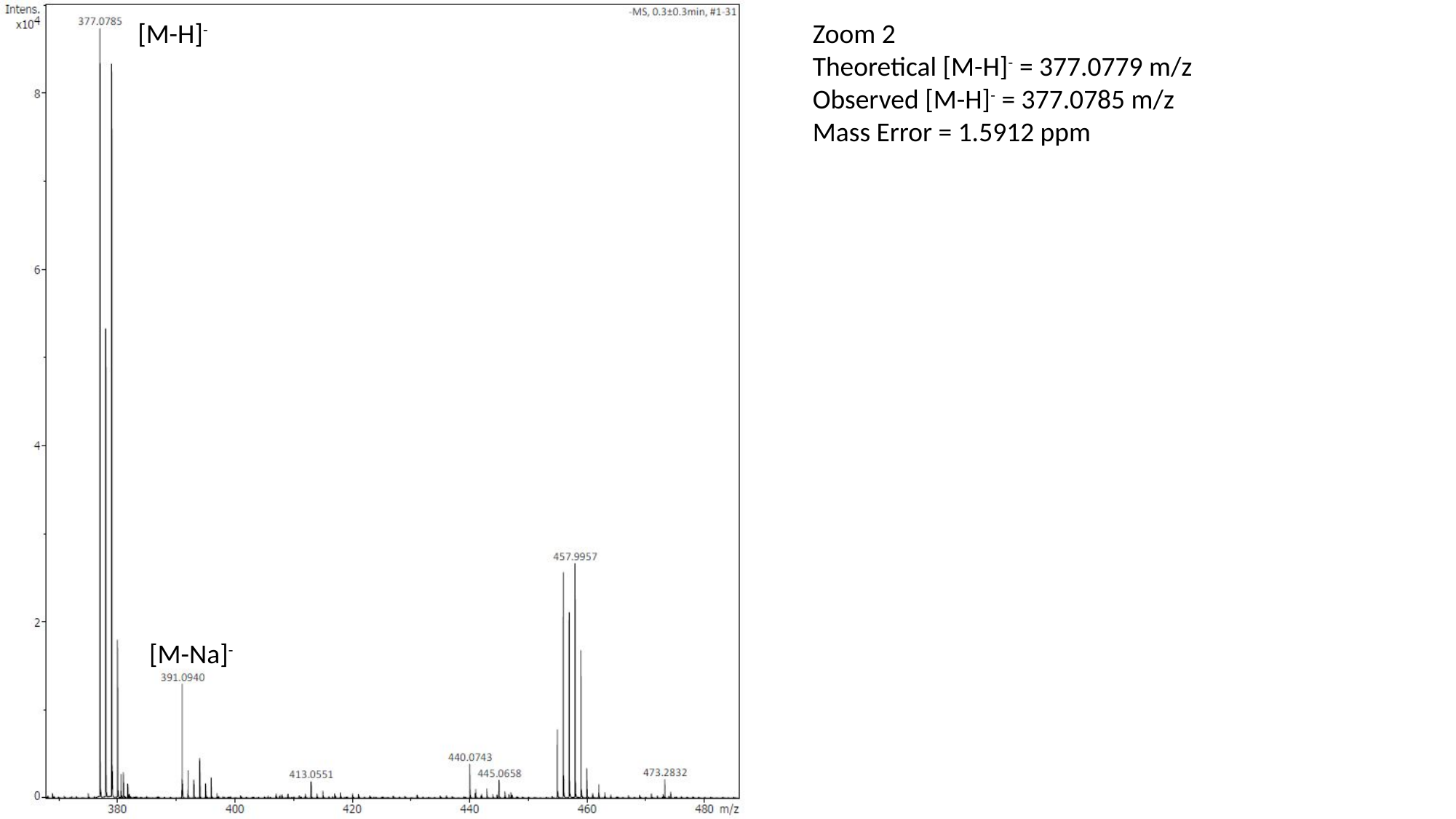

[M-H]-
Zoom 2
Theoretical [M-H]- = 377.0779 m/z
Observed [M-H]- = 377.0785 m/z
Mass Error = 1.5912 ppm
[M-Na]-

## Slide 10
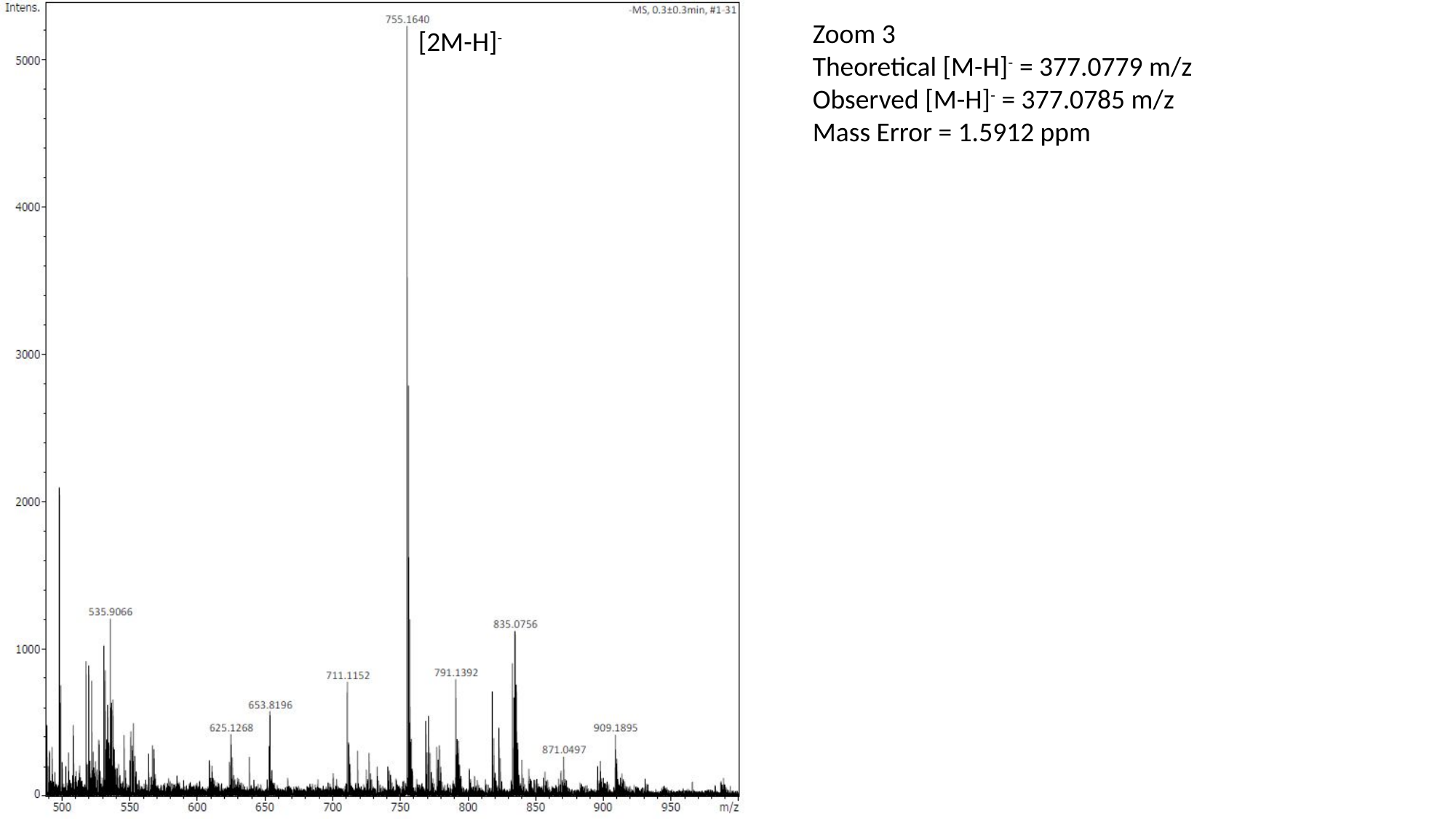

Zoom 3
Theoretical [M-H]- = 377.0779 m/z
Observed [M-H]- = 377.0785 m/z
Mass Error = 1.5912 ppm
[2M-H]-

## Slide 11
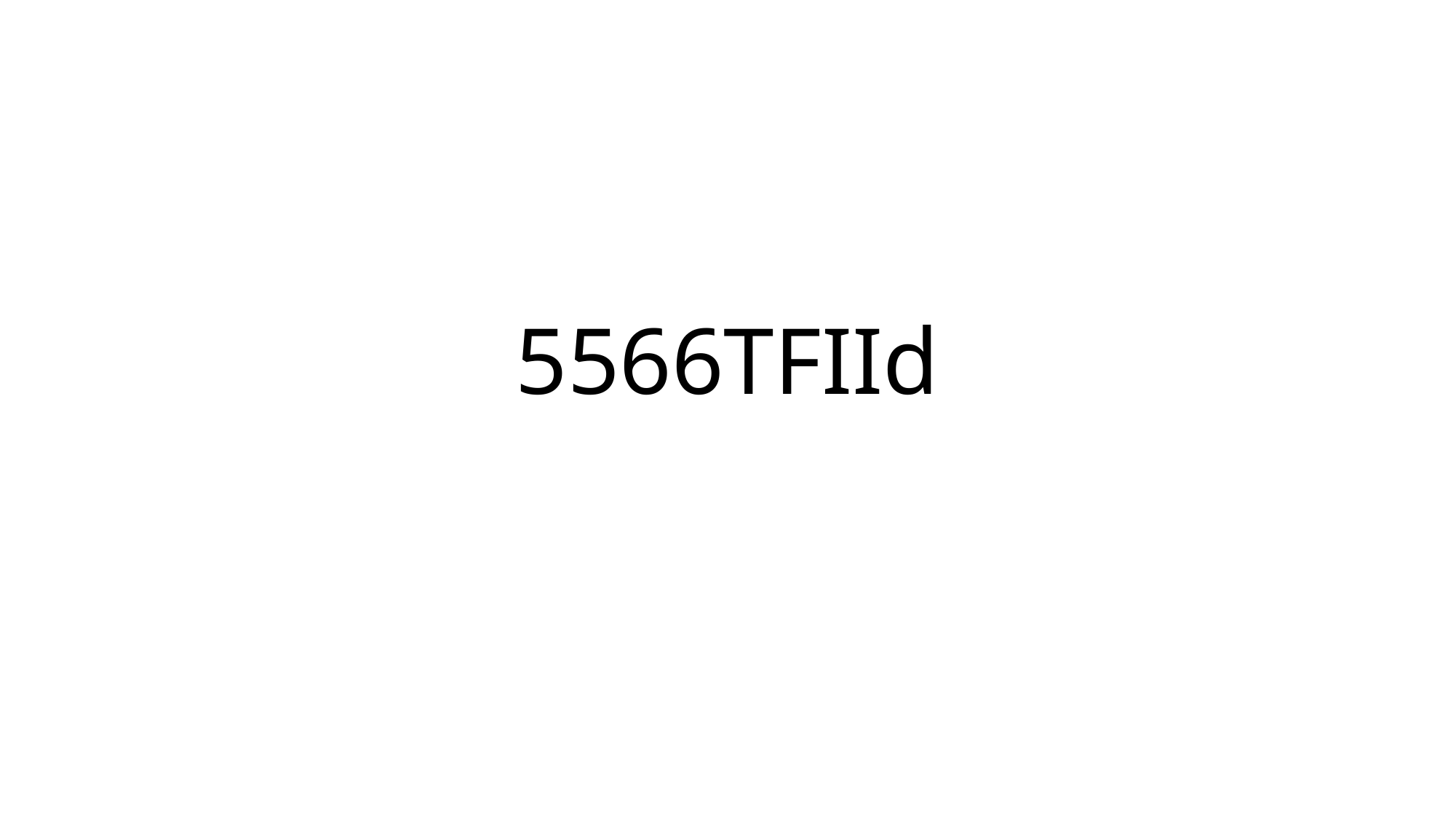

# 5566TFIId

## Slide 12
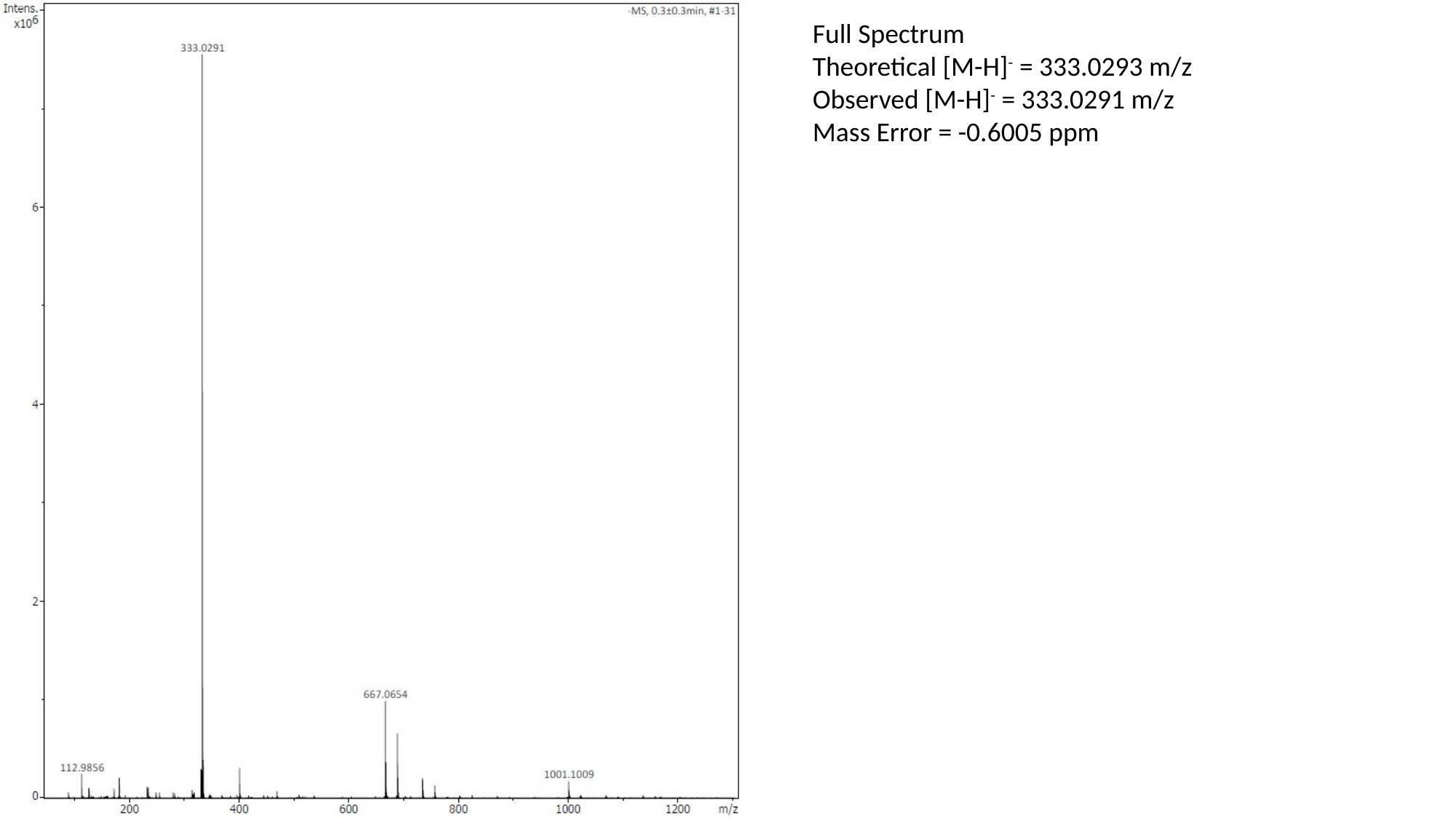

Full Spectrum
Theoretical [M-H]- = 333.0293 m/z
Observed [M-H]- = 333.0291 m/z
Mass Error = -0.6005 ppm

## Slide 13
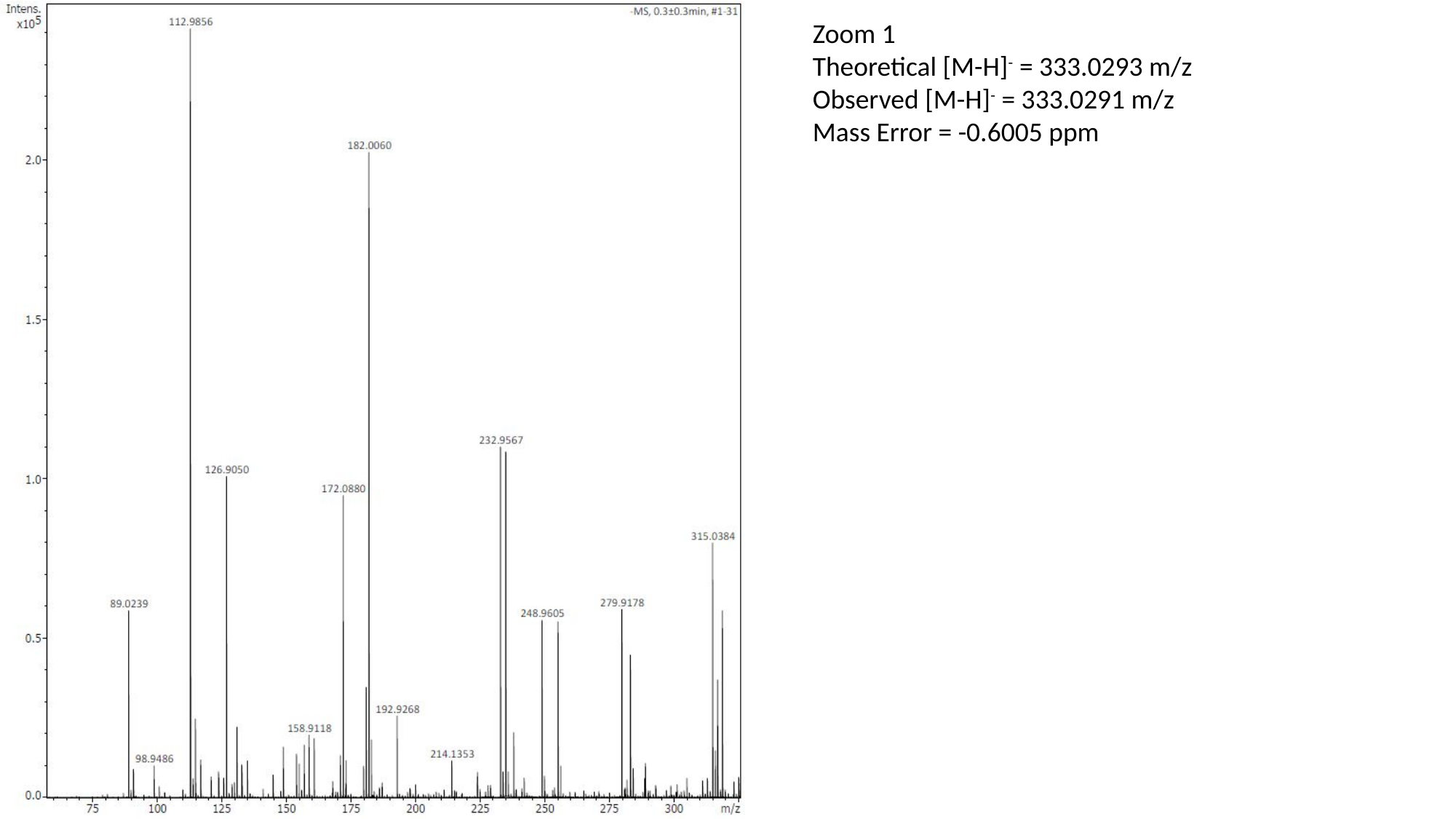

Zoom 1
Theoretical [M-H]- = 333.0293 m/z
Observed [M-H]- = 333.0291 m/z
Mass Error = -0.6005 ppm

## Slide 14
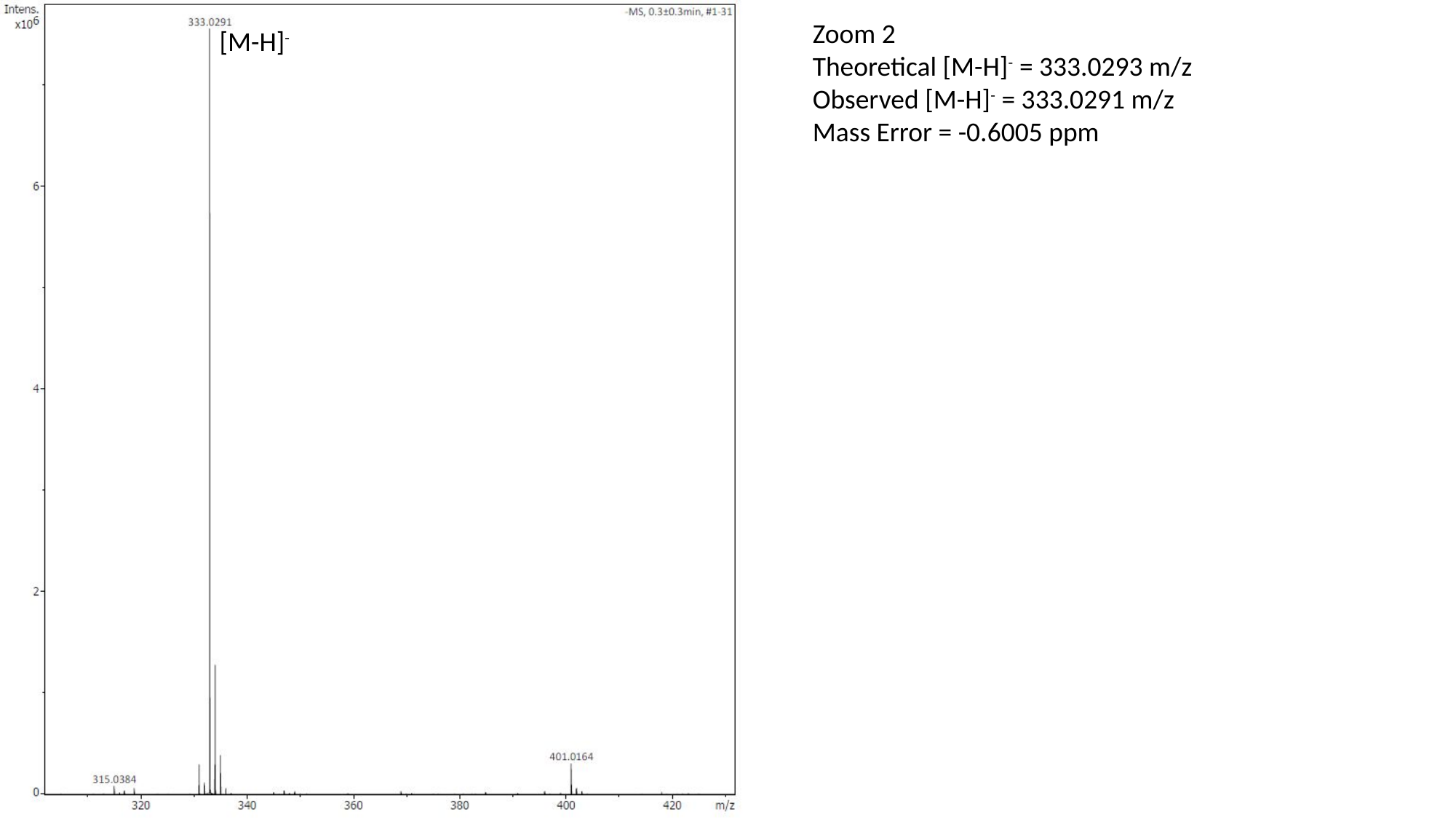

Zoom 2
Theoretical [M-H]- = 333.0293 m/z
Observed [M-H]- = 333.0291 m/z
Mass Error = -0.6005 ppm
[M-H]-

## Slide 15
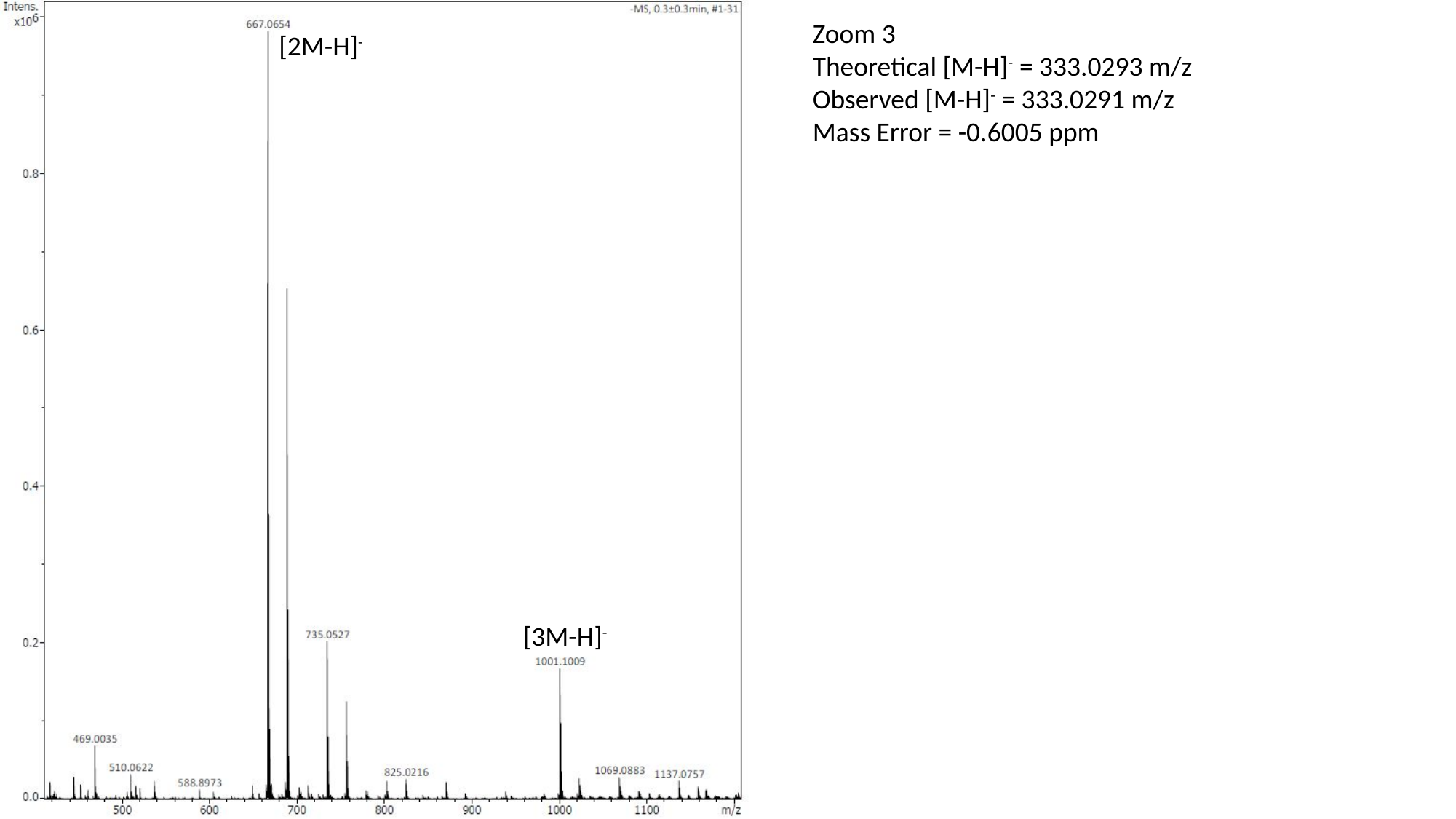

Zoom 3
Theoretical [M-H]- = 333.0293 m/z
Observed [M-H]- = 333.0291 m/z
Mass Error = -0.6005 ppm
[2M-H]-
[3M-H]-
